# FastANI, Mash and Dashing equally differentiate between *Klebsiella* species

**DOI:** 10.7717/peerj.13784

**Published:** 2022-07-21

**Authors:** Julie E. Hernández-Salmerón, Gabriel Moreno-Hagelsieb

**Affiliations:** Department of Biology, Wilfrid Laurier University, Waterloo, Ontario, Canada

**Keywords:** Sketching algorithms, FastANI, Hierarchical clustering, Genome comparison, Klebsiella

## Abstract

Bacteria of the genus *Klebsiella* are among the most important multi-drug resistant human pathogens, though they have been isolated from a variety of environments. The importance and ubiquity of these organisms call for quick and accurate methods for their classification. Average Nucleotide Identity (ANI) is becoming a standard for species delimitation based on whole genome sequence comparison. However, much faster genome comparison tools have been appearing in the literature. In this study we tested the quality of different approaches for genome-based species delineation against ANI. To this end, we compared 1,189 *Klebsiella* genomes using measures calculated with Mash, Dashing, and DNA compositional signatures, all of which run in a fraction of the time required to obtain ANI. Receiver Operating Characteristic (ROC) curve analyses showed equal quality in species discrimination for ANI, Mash and Dashing, with Area Under the Curve (AUC) values above 0.99, followed by DNA signatures (AUC: 0.96). Accordingly, groups obtained at optimized cutoffs largely agree with species designation, with ANI, Mash and Dashing producing 15 species-level groups. DNA signatures broke the dataset into more than 30 groups. Testing Mash to map species after adding draft genomes to the dataset also showed excellent results (AUC above 0.99), producing a total of 26 *Klebsiella* species-level groups. The ecological niches of *Klebsiella* strains were found to neither be related to species delimitation, nor to protein functional content, suggesting that a single *Klebsiella* species can have a wide repertoire of ecological functions.

## Introduction

Multi-drug resistant bacteria represent a global threat to human health. Strains of the *Klebsiella* genus are among the most common antibiotic resistant human pathogens, causing as much as 50% mortality in infected neonatal, elderly and immunocompromised patients (Podschun & Ullmann, 1998; Xu, Sun & Ma, 2017). *Klebsiella* are considered ubiquitous in the environment (*i.e*., water, soil and plants), commonly found in the mucous surfaces of mammals, and as insect symbionts (Pinto-tomás et al., 2009; Martínez-Romero et al., 2015; Davis & Price, 2016). Imprecise detection methods affect the identification of potentially pathogenic strains (Podder et al., 2014; Rodríguez-Medina et al., 2019). Particularly, nearly identical *K. pneumoniae* strains, isolated from disparate sources, have been found to be almost as virulent as strains of a clinical origin (Struve & Krogfelt, 2004; Huang et al., 2016; Dantur et al., 2018). This often leads to classification difficulties and taxonomic biases (Long et al., 2017), highlighting the need for faster and accurate techniques for the identification of *Klebsiella* isolates from environmental sources with potential to infect people.

To date, techniques to classify *de novo* sequenced *Klebsiella* genomes at the species level are still under development (Garza-Ramos et al., 2015; Long et al., 2017; Hennart et al., 2022). Some methods include the use of PCR-based probes based on genetic markers, such as the Multi Locus Sequence Typing (MLST) methods selected to identify *K. variicola* strains (Garza-Ramos et al., 2015; Barrios-Camacho et al., 2019); hierarchical clustering tools to classify *K. pneumoniae*, associated with antibiotic resistance to *β*-lactamase compounds (Berrazeg et al., 2013); and pan-genomic analysis to redefine subspecies of *K. pneumoniae* as new species of this genus (Caputo et al., 2015). These techniques aimed for the identification of particular species, but the current amount of genomic data allows for the extensive study of all available information at the whole genomic level.

Currently, the most common measure to delimit species using genome sequences seems to be the Average Nucleotide Identity (ANI), which has been used to revise the taxonomy of prokaryotes (Goris et al., 2007; Richter & Rosselló-Móra, 2009; Jain et al., 2018). ANI is an homology-dependent measure derived from pairwise alignments calculated by a number of algorithms, such as blastn (Altschul et al., 1997), and mummer (Kurtz et al., 2004). However, to improve the speed for analyzing large amounts of sequence data, other methods have been developed. FastANI uses an alignment-free mapping algorithm (Mashmap) implemented to approximate ANI calculations in a range of 80–100% identity (Jain et al., 2018). Mash is based on the construction of MinHash sketches, derived from samples of small oligonucleotides (normally 21 bp long), and transformed into hashes that can be efficiently computed and compared (Ondov et al., 2016). Dashing is another program using a computer transformation of oligonucleotides, hyperloglog sketches, to improve speed and produce results similar to those of Mash (Baker & Langmead, 2019). Methods based on compositional analyses, based on oligonucleotides no more than 4 bp long, have also been used to group genomes (Richter & Rosselló-Móra, 2009; Moreno-Hagelsieb et al., 2013; Hernández-González, Moreno-Hagelsieb & Olmedo-Álvarez, 2018).

The present study aims to test the accuracy and efficiency of different methods in grouping *Klebsiella* genomes into their annotated species, as well as to determine if their genetic content is associated with the environment of isolation.

## Methods

### Genomic data

A total of 13,574 genome sequences of *Klebsiella* were downloaded from NCBI’s RefSeq database (Haft et al., 2018) in December 2021. Of these, only 1,189 were complete, or closed, genomes (Table S1). This study focused mainly on the complete genomes dataset. The sources of isolation were compiled from the information available in both RefSeq and PATRIC (Davis et al., 2020). Identification of type strains relied on the descriptions found in the RefSeq gbff files (Table S1).

### Pairwise distances

To calculate Average Nucleotide Identity (ANI), we used FastANI v1.32 (Jain et al., 2018), which calculates a close approximation to the original ANI implementation (Goris et al., 2007), which was based on pairwise alignments produced with blastn (Altschul et al., 1997). Other tools used for comparing genome sequences included Mash v2.2.2 (Ondov et al., 2016), Dashing v1.0 (Baker & Langmead, 2019), and DNA compositional signatures (Campbell, Mrázek & Karlin, 1999). We ran FastANI with a fragment length of 1,020 bp to better approximate the original ANI calculations (Goris et al., 2007). For Mash, we used the default k-mer length of 21 nt, besides a sketch size of 5,000, rather than the default of 1,000, since increasing the sketch size should improve the accuracy of the obtained dissimilarities (Ondov et al., 2016). For Dashing, we tested three measures: mash, full-mash and, the default, Jaccard. We selected a sketch size option of 2^14^ (-S 14), because this size was found to be optimal for estimating Jaccard similarities by the authors of the program (Baker & Langmead, 2019). We also used a k-mer length of 21 to make the results more comparable to those of Mash, given that our initial tests found that Dashing, ran with its default k-mer length of 31, produced results for fewer pairwise comparisons than Mash. The options used for each program are shown in Table 1. DNA signatures were calculated using an *ad hoc* program, written in perl. We obtained DNA signatures for di, tri, and tetra nucleotides as reported previously (Moreno-Hagelsieb et al., 2013).

**Table 1:** Commands used to run each program.

fastANI ––ql [queries list] ––rl [refs list] ––fragLen 1020
mash sketch -s 5000 -o [mshfile] [infile]
mash triangle -E [mshfiles produced above]
dashing cmp -k 21 -S 14 -T -O jaccard.matrix [infiles]
dashing cmp -k 21 -S 14 -T -O full-mash.matrix ––full-mash-dist [infiles]
dashing cmp -k 21 -S 14 -T -O mash.matrix ––mash-dist [infiles]

The pairwise values obtained were transformed into distances when appropriate. Accordingly, ANI values, consisting of percent identities, were transformed into dissimilarities by subtracting them from 100, then dividing the results by 100; Jaccard indexes, obtained with Dashing, were subtracted from 1. All Mash values represent dissimilarities and, thus, did not require transformation. DNA signatures were compared using *δ* (Campbell, Mrázek & Karlin, 1999), consisting on Manhattan distances divided by the length of the signature vector (Campbell, Mrázek & Karlin, 1999; Moreno-Hagelsieb et al., 2013).

### Clustering analysis and optimal cut points

**Figure 1:**
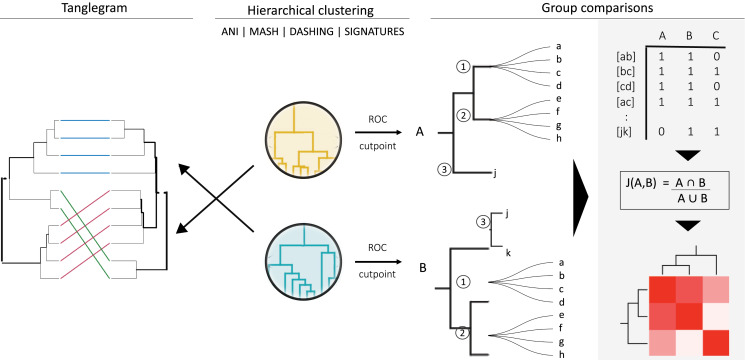
General strategy for program and group comparisons. Hierarchical clustering was performed first (center). Clustering was followed by pairwise comparisons of all clusters obtained. Cut points specific for each dataset were calculated by optimizing F1 scores. The hierarchies were cut at the thresholds obtained. The members (a, b, c, *etc*.) of the resulting groups (nodes 1, 2 and 3) were rearranged into linked pairs and compared.

To divide *Klebsiella* strains into species-level groups, we first performed hierarchical clustering based on the distances obtained with each method described above (Fig. 1). These clusters were produced using hclust and the divisive method (diana), implemented in R (R Core Team, 2021). Representative clusters were plotted using ggtree (Yu, 2020) and ggtreeExtra (Xu et al., 2021).

To evaluate performance and obtain optimum cutoff values for each of the programs tested, we calculated Receiver Operating Characteristic (ROC) curves (Swets, 1988). The positive dataset consisted of pairs of genomes assigned to the same species, while the negative dataset consisted of pairs of genomes in different species, but same genus. Only genomes annotated at the species level were used for these evaluations. Both ROC analyses and optimal cutoff points were estimated with the cutpointr R package (Thiele & Hirschfeld, 2021). We optimized for a maximum F1 score ${({[2 \times TP]} /{[2 \times TP + FP + FN]} )}$. These evaluations assumed that most of the genomes downloaded from RefSeq were correctly classified into species.

To avoid biases in ROC curves and optimal cutoff analyses due to over-representation of *K. pneumoniae* strains, we produced 15 randomized samples of 62 genomes of *K. pneumoniae*. Each sample along with the rest of the complete genomes made up 15 testing datasets.

### Comparing results

Hierarchical clusters were compared by calculating their Baker’s Gamma Index (Baker, 1974), rank correlations between the points, or thresholds, where pairs of items combine in the compared dendrograms. The similarities were visualized using entanglement plots enhanced using the “step1side” untangle method. To produce such plots and calculate Baker’s Gamma Indexes, we used the dendextend package in R (Galili, 2015). The more similar the clusters are to each other, the lower the entanglement value, which ranges between 0 and 1, and the larger their Baker’s Gamma Index, which ranges between −1 and 1, with zero representing null correlations.

To compare the groups resulting from cutting hierarchical clusters at optimal cutoff thresholds, we reorganized each group into pairs of genomes belonging to the same group. The similarities between assigned pairs by each program were compared in terms of shared genome pairs (Fig. 1). An *ad hoc* program was written in perl to analyse the species composition of the groups produced.

### Domain content

Protein domains were obtained by comparing the proteins encoded by all genomes analysed, using mmseqs2 (Steinegger & Söding, 2017), against the appropriately formatted Pfam (Finn et al., 2015) and CDD databases (Marchler-Bauer et al., 2017). An *ad hoc* perl script was written to gather the domain results into a single table to compare genome-to-genome domain content using Jaccard distances as implemented in the philentropy package in R (Drost, 2018). The results were used to perform divisive hierarchical clustering for an overall view of domain content similarities.

## Results

### FastANI, Mash and Dashing classify *Klebsiella* into almost identical groups

A total of 1,189 complete genome sequences labeled as *Klebsiella* were downloaded from the RefSeq database (Haft et al., 2018). As of December 2021, this dataset contained 1,168 strains mapped to 10 named species, and 21 strains without a species designation (Table S1).

**Figure 2:**
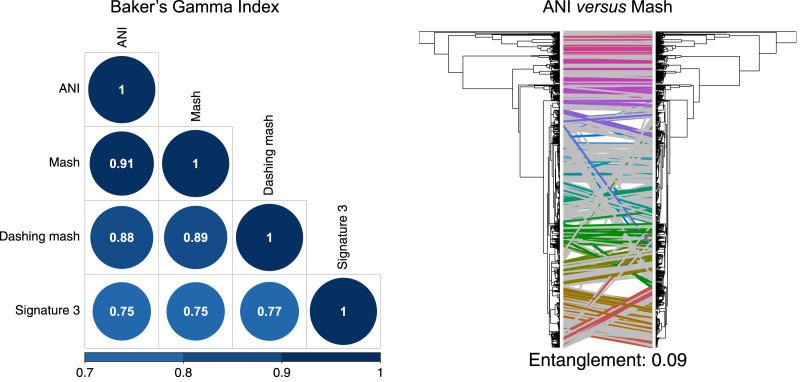
Comparing clusters. Left: Baker’s Gamma Indexes show that hierarchies produced with the different sketching algorithms (Mash and Dashing) produced the most similar hierarchies to that produced with FastANI. Right: Mash produced the hierarchy most similar to that produced by FastANI.

To produce hierarchical clusters with the complete genome dataset (Fig. 2), we used distances calculated with FastANI, Mash and DNA signatures, plus three available in Dashing (mash, full-mash and Jaccard). All hierarchies were compared using Baker’s gamma correlations (Baker, 1974), and their similarities visualized using Entanglement plots. Mash produced the most similar cluster to that produced by FastANI (Fig. 2). Two Dashing calculated distances, mash and full-mash, had a Baker’s Gamma Index of 1.00, while Dashing’s Jaccard distances had a Baker’s Gamma Index of 0.96 with the other Dashing distances (Fig. S1). Therefore our illustrations used Dashing mash as a representative of all Dashing results.

**Figure 3:**
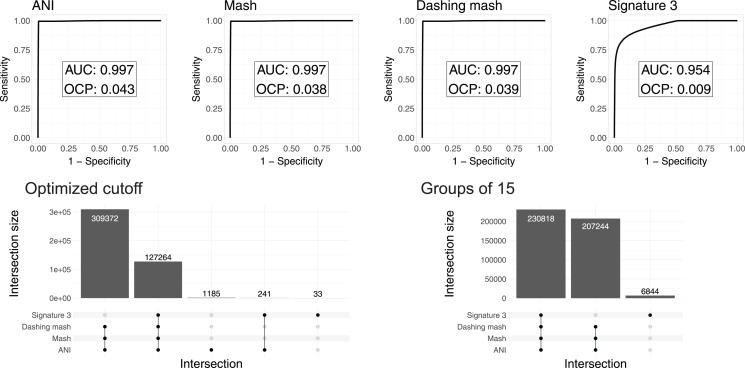
ROC curve analyses. Top: the area under the curve (AUC) suggests almost perfect species delineation for FastANI, Mash and Dashing mash, with lower performance for DNA signatures. Bottom, left: the UpSet plot compares the genome pairs grouped together after pruning the dendrograms at the specific optimal cut points (OCP) obtained for each program. FastANI, Mash and Dashing shared almost all of the grouped pairs. Bottom, right: after using cutoff to obtain 15 groups with all distances, FastANI, Mash and Dashing produced identical groups.

To test and compare the accuracy of the methods in species assignment, we performed Received Operating Characteristic (ROC) analyses (Swets, 1988). ROC curves for FastANI, Mash and all Dashing distances resulted in the same Area Under the Curve (AUC) of 0.997 (Fig. 3, Fig. S1), which suggests that the distances produced by all three programs are close to perfection in distinguishing genomes from the same species from those in the same genus but different species. DNA signatures had lower, but respectable, AUC values above 0.95 (Fig. 3, Fig. S1).

To ensure that the AUC values were not the result of *K. pneumoniae* over-representation, many of which have very low distances, we produced 15 subsampling datasets randomly assigning 62 *K. pneumoniae* to the complete dataset. The quality of the programs in differentiating species showed the same high value of AUC 0.98 for all 15 subsamples using FastANI, Mash and Dashing mash.

To compare results from cutting the clusters into species-level groups, we obtained cut points optimizing the F1 score. After cutting the hierarchies using these cutoffs, we obtained 15 groups from the complete genome dataset with FastANI, 18 with Mash and Dashing, and more than 30 with DNA Signatures. To determine whether they contained similar groups, we checked each pair of genomes found within each group. Set intersection analyses showed that FastANI, mash, and all Dashing distances, produced almost identical groups (Fig. 3, Fig. S1), while DNA signatures produced more evident differences. A closer look showed that the groups produced by Mash and Dashing were identical, which should be expected given that they use very similar strategies and formulas. Distances calculated with FastANI resulted in 1,426 pairwise assignments not found by the sketching programs (Mash and Dashing), which amounts to 0.3% of the 436,636 pairwise designations shared by FastANI and the sketching programs.

To try and match the FastANI results, we found cutoff values to obtain 15 groups with the sketching algorithms. In all cases, the resulting groups were identical to those obtained with FastANI. Similarly, finding a threshold to obtain 18 groups with FastANI resulted in identical groups as those obtained with the sketching algorithms at their optimal cut points as estimated by the cutpointr analyses. As explained above, in pairwise terms, the 15 and 18 groups share most of their group assignments on a per genome pair basis. Thus, the conflict was minimal. However, the groups resulting from matching group numbers between programs were identical deserves further explanation. The ROC curves and derived optimal cutoff values were performed with tables containing same-species and same-genus genome pairs, while the grouping was performed on genomes gathered into hierarchical clusters. Thus, the differences can be attributed to differences in grouping strategies.

**Figure 4:**
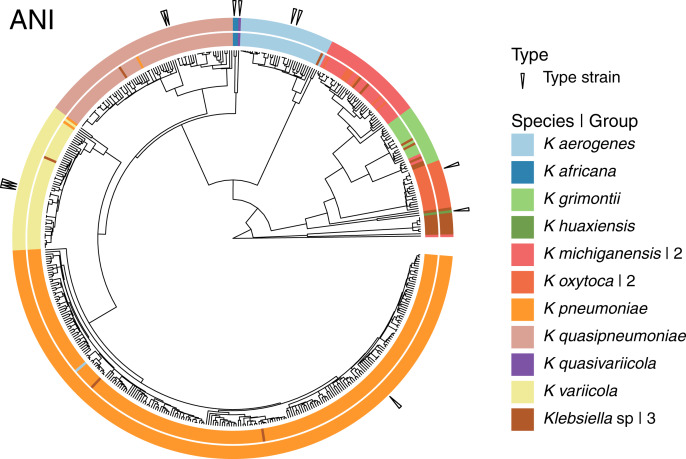
Species annotation *vs* groups. The 15 groups produced by cutting the sketching and FastANI hierarchies (outer circle) coincide well with species annotation (inner circle), fixing the few apparently mislabeled genomes. The numbers to the right indicate number of groups sharing the species name. Three groups remained without a species name. To improve the illustration, the confirmed *K. pneumoniae* dataset was reduced by keeping representatives from clusters obtained at a Mash distance of 0.005.

Given that the original dataset contained 10 named species, we continued the analyses using the 15 groups results (Fig. 4). Three of these 15 groups were composed entirely of *Klebsiella* strains with no species designation. Among the remaining 12 groups; two were formed by the species represented by single genomes, *K. huaxiensis* and *K. quasivariicola*, both type strains of their respective species. Another group was formed by the single species represented by two genomes, *K. africana*, one of them, 200023, described as a type strain of the species. The remaining nine groups, loosely ordered by the number of genomes included, from largest to smallest, were (Fig. 4):

The *K. pneumoniae* group. This group was the largest and contained 927 of the 930 *K. pneumoniae* strains, combined with one of the 35 *K. aerogenes* strains, and two unspecified *Klebsiella* strains. This suggests that the *K. aerogenes* strain, NCTC9644, is a misidentified *K. pneumoniae*. This group contained the *K. pneumoniae* type strain FDAARGOS775.

The *K. quasipneumoniae* group. This group gathered all 74 *K. quasipneumoniae* strains, one of the *K. pneumoniae* strains missing in the group above, and one unspecified strain, suggesting that the *K. pneumoniae* strain, KAM260, was a *K. quasipneumoniae* isolate. The *K. quasipneumoniae* type strains 01A030T and FDAARGOS1503 were part of this group.

The *K. variicola* group. This group contained all 53 *K. variicola* strains, the last two missing *K. pneumoniae* strains, and one unspecified strain. Therefore, these two *K. pneumoniae* strains, YH43 and INF022-sc-2279895, might be *K. variicola* isolates. Three of the members of this group were *K. variicola* type strains: DSM15968, F2R9 and F2R9T.

The *K. aerogenes* group. This group contained all of the remaining 34 *K. aerogenes* strains, forming a clean, single-species, group with one unspecified strain. Two members of the group, FDAARGOS1442 and KCTC2190, were type strains.

The two *K. michiganensis* groups. One of these groups was formed by 29 of the 32 *K. michiganensis* strains, seven of the 25 *K. oxytoca* strains and four unspecified ones, suggesting that the seven *K. oxytoca* strains were misidentified *K. michiganensis*. One more *K. michiganensis* strain, RC10, formed a group of its own.

The *K. grimontii* group. This group contained all 15 *K. grimontii* strains, the two missing *K. michiganensis* strains, and five unspecified ones, suggesting that the *K. michiganensis* strains, B106 and Sb-24, were misidentified *K. grimontii* isolates.

The two *K. oxytoca* groups. Finally, of the remaining 18 *K. oxytoca* strains, 17 formed a clean, single-species group and one formed a group by itself, reinforcing the suggestion above that the seven *K. oxytoca* strains mentioned above were *K. michiganensis* isolates.

The presence two single strain groups sharing species names with larger groups, namely one of *K. michiganensis* and one of *K. oxytoca*, suggests that a different, less stringent, threshold might join them with their corresponding, largest, groups. However, as observed in the hierarchy (Fig. 4), the isolated *K. michiganensis* strain is the outermost outlier in the cluster. Though the isolated *K. oxytoca* strain is not as clearly separated from the rest of the largest *K. oxytoca* group, no threshold put them together without joining otherwise clean groups. These results suggest that the separated *K. oxytoca* and *K. michiganensis* strains might either constitute *Klebsiella* species other than those represented in the complete genome dataset, or altogether mislabeled strains.

### Neither species, nor protein content, seem related to isolation sources

**Figure 5:**
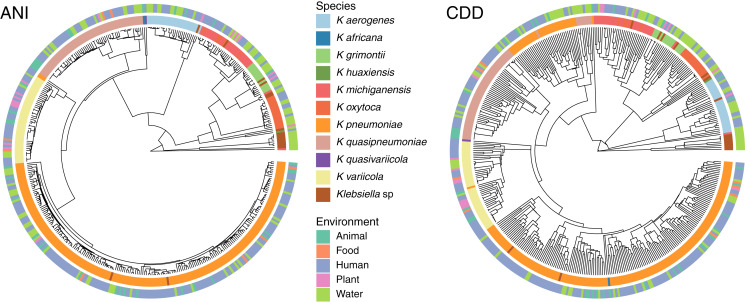
Domain content and isolation source. Isolation source neither follows species, nor domain content. For illustration purposes, the hierarchy plot used a reduced *K. pneumoniae* dataset filtered at a Mash distance cutoff of 0.005. Only genomes with isolation source annotations in PATRIC (Davis et al., 2020) are shown.

To determine the relationship between genetic traits and the source of isolation in clustering species, we clustered the complete genomes of *Klebsiella* according to their domain content, and mapped the different hosts and environments from which they were isolated into the hierarchies (Fig. 5). The variety of hosts and source information available for the complete genomes was binned into five environments: animals, food, human, plants and water. While domain content was similar to the FastANI cluster (Baker’s Gamma Index: ANI *vs.* CDD = 0.87; ANI *vs.* Pfam = 0.85; CDD *vs.* Pfam = 0.93), the environment of isolation did not appear to have an effect on the grouping of *Klebsiella* strains (Fig. 5).

No matter where the strains were isolated from, they kept a clear species-specific cohesion, particularly those isolated from a clinical environment. In this regard, some authors have argued that intraspecific ecological and genetic interactions may constrain the diversification within a species (Cohan, 2019). Thus, environmental microbes isolated from different sources tend to display a genetic continuum, such as *K. variicola*, which was isolated from almost all environments, yet it appears as a single cluster.

### After adding draft genomes, Mash divides *Klebsiella* genomes into 26 species

**Figure 6:**
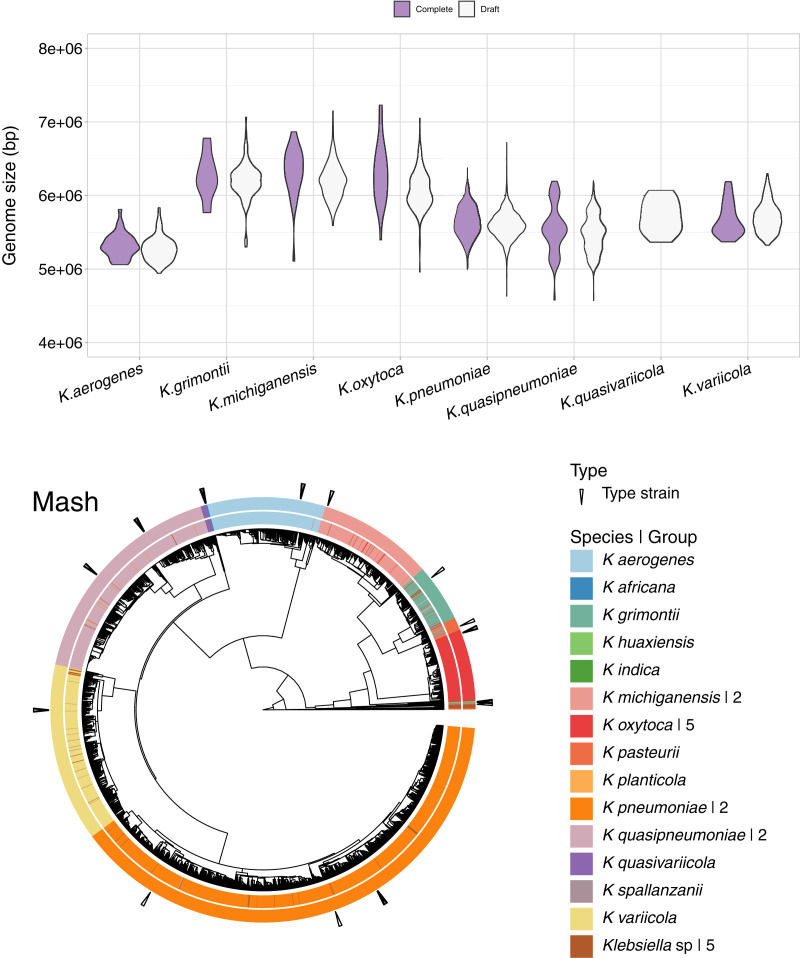
Draft genomes analysis. Top: genomic length of complete and draft genomes of *Klebsiella* species. The genome length appeared similar regardless the type of assembly. *K. oxytoca*, *K. michiganensis* and *K. grimontii* show the largest genomes with over 7 Mb, while *K. quasipneumoniae* has smaller genomes ranging from 4.5 to 6 Mbp. Bottom: Mash clustering for complete and draft genomes show similar results to those of obtained with the complete genomes alone. The numbers to theright indicate number of groups sharing the species name. For illustration purposes, the hierarchy plotused a reduced *K. pneumoniae* dataset filtered at a Mash distance cutoff of 0.005.

In addition to the 1,189 complete *Klebsiella* genomes, we obtained a total of 12,385 draft genomes of different assembly status/categories: Chromosome, Scaffold and Contig (Table S1). Genome lengths appeared to be similar between sequences belonging to the same species, regardless of assembly status (Fig. 6). *K. oxytoca*, *K. michiganensis* and *K. grimontii* showed the largest genomes with over 7 Mbp, while strains of *K. aerogenes* had the smallest genomes ranging from 4.5 to 6 Mbp.

Since Mash and Dashing produced the most similar results to FastANI, we decided to test Mash with all types of genome assemblies. The results remained excellent, with an AUC of 0.999. The cut point value obtained was the same 0.038 as the one obtained for the complete genomes alone. Using this cutoff, we obtained 28 species-level groups of *Klebsiella*, while a cutoff of 0.043 reduced the obtained groups to 26 (Fig. 6, Table S1). The difference consisted on the fusion of two *K. aerogenes* groups, containing each 328 and 23 genomes, into one; plus the incorporation of a singled out *K. quasivariicola*, strain Q2548, into a group containing all three available *K. africana* strains. Since the dataset contains a total of 13,574 genome sequences, an improvement in the position of 24 genomes might be considered modest, suggesting, again, slight differences when optimizing cutoffs based on pairwise relationships, compared to hierarchical clustering.

Of the 26 groups obtained at the 0.043 cutoff, five were composed exclusively of unspecified *Klebsiella* genomes. One group contained the only representative of its species, *K. planticola*. The rest, sorted from largest to smallest, also named after their most abundant species annotation, were (Fig. 6).

Two of *K. pneumoniae*. The first group, by far, the largest, contained 11,219 strains, around 83% of the 13,574 *Klebsiella* genomes analysed. Of these, 11,194 were appropriately labeled, one was the *K. aerogenes* strain, NCTC9644, found in the complete genome analysis above, and the remaining 24 were unspecified strains. Six of the strains in the group were *K. pneumoniae* type strains. The other group consisted of a single, highly divergent, strain, *K. pneumoniae* 4300STDY6470518.

One of *K. variicola*. This group was composed of 502 appropriately named genomes, three labeled *K. pneumoniae*, and 28 unspecified strains. Three of the members of the group were *K. variicola* type strains.

Two of *K. quasipneumoniae*. The first group consisted of 433 appropriately named strains, plus eight named *K. pneumoniae* and six unspecified ones. Two of the members of the group were *K. quasipneumoniae* type strains. The second group contained 209 appropriately named strains, one labeled as *K. pneumoniae*, strain KPN1344, and two unspecified strains. Four members of the second group were *K. quasipneumoniae* type strains. Accordingly, a relaxed cutoff of 0.05 joined these two groups together.

One of *K. aerogenes*, composed, as explained above, of 352 genomes. A total of 351 of these appropriately named, plus one unspecified strain: *Klebsiella* sp A52. Two of these genomes belonged to *K. aerogenes* type strains: KCTC2190 and FDAARGOS1442.

Two of *K. michiganensis*. The largest of these groups was composed of 320 appropriately labeled strains, two of them type strains: DSM25444 and CCUG66515; 13 labeled as *K. oxytoca*, adding six to those clustering with *K. michiganensis* in the complete genome analyses presented above; and nine unspecified genomes. The second group consisted of the single, highly divergent *K. michiganensis* strain, RC10, identified in the complete genome analyses above.

Five of *K. oxytoca*. All of these groups were clean, with the largest one containing 207 appropriately named strains, three of them type strains; plus three unspecified ones. The remaining groups contained five appropriately labeled strains in total. A cutoff of 0.05 reduced these groups to four.

One of *K. grimontii*, composed of 133 appropriately named genomes, 18 labeled as *K. michiganensis*, thus adding 16 strains to the ones appearing in the whole genome analysis above. Other members of the group were seven apparently mislabeled *K. oxytoca* strains. The group also included 13 unspecified strains. The group contained one type strain: *K. grimontii* 06D021.

One of *K. pasteurii*. This was a somewhat controversial group. The group contained a total of 38 genomes, five of them unspecified. Of the remaining genomes, 14 were labeled *K. pasteurii*, one of them a type strain: *K. pasteurii* SB6412. Of the other members of the group, 11 were named *K. michiganensis*, and eight *K. grimontii*. The type strain indicated the group to be appropriately named. However, the comparable proportion of apparently inappropriately named strains suggested otherwise. Accordingly, a relaxed cutoff of 0.05 joined this group with the *K. grimontii* group. Though we chose the 0.043 threshold to present these results, because it left a *K. pasteurii* group in the list, these analyses suggested that the *K. pasteurii* and *K. grimontii* groups belonged together, and that a threshold higher than 0.043 should be chosen for delimiting *Klebsiella* species.

One of *K. quasivariicola*. This was a clean group composed of 16 appropriately named genomes, one of them a type strain: *K. quasivariicola* KPN1705.

One of *K. africana*. This group was composed, as explained above, of three appropriate labeled strains, plus one labeled as *K. quasivariicola* Q2548. Two of the members of the group were *K. africana* type strains: 200023 and SB5857.

One of *K. spallanzanii*. This was also a clean group containing a type strain: *K. spallanzanii* SB6411.

One of *K. huaxiensis*, composed of three appropriately labeled genomes, one of them a type strain: *K. huaxiensis* WCHKl090001.

Finally, one of *K. indica*, composed of just two genomes, one of them the species type strain, TOUT106; the other one unspecified, *Klebsiella* sp 2680.

## Discussion

With the advances in sequencing technologies, it becomes essential to develop and validate efficient tools to handle large amounts of genomic data. ANI has been used worldwide since the authors found that a threshold of 95% mirrored the 70% DNA-DNA hybridization threshold recommended for species delimitation (Wayne et al., 1987), with the advantage of being applicable across any sequenced prokaryotic species (Richter & Rosselló-Móra, 2009). However, it is computationally intensive to calculate ANI, since these values are based on whole genome comparisons (Zhou et al., 2020). Apart from this, a recent revision has argued against the accuracy of the threshold proposed by the authors as a universal microbial species delineation, substantial sampling and species redundancy was found to alter the results and found no evidence of a universal genetic boundary among microbial species currently annotated in the NCBI taxonomy (Murray, Gao & Wu, 2021).

While recent developed programs have tried to deal with speed issues, better knowledge of the accuracy of their results is needed before they can be widely adopted to assign species. Our results show that Mash and Dashing produce the same results as FastANI. The optimized threshold for Mash, 0.038, closely corresponds with a previous analysis in *Escherichia coli*, where the authors reported a cutoff of 0.037 (Abram et al., 2021). As for Dashing, we could not find any tests in a specific bacterial species, apart from the experiments performed when the program was released (Baker & Langmead, 2019). However, since we obtained the same species groups as FastANI and Mash, we anticipate it may provide reliable results with highly improved computational efficiency. DNA signatures still offer a good approximation to the results produced by FastANI and Mash, with an AUC of 0.954. Little attention has been given to compositional methods, even though they have demonstrated various applications apart from clustering species (Richter & Rosselló-Móra, 2009), such as identification of exogenous DNA through horizontal transfer events, pathogenicity islands and bacteriophages (Bohlin, 2011).

Our study was conducted using all the genome sequences labeled as *Klebsiella* strains in the RefSeq database as of December 2021. This genus contained a variety of species with diverse ecological functions. The most studied, *K. pneumoniae*, is considered the third worldwide leading pathogen for deaths associated to resistance, after *E. coli* and *Staphylococcus aureus* (Murray et al., 2022). The genus also includes other important opportunistic human pathogens, such as *K. aerogenes*, *K. variicola* and *K. oxytoca* (Tomulescu et al., 2021). Members of the last two species also display plant-growth promoting activities such as nitrogen-fixation and production of relevant compounds with biotechnological applications (Tomulescu et al., 2021). The most recently described species, *K. huaxiensis*, *K. grimontii* and *K. africanensis* have been recovered from cattle and human feces/urine (Passet & Brisse, 2018; Hu et al., 2019; Rodrigues et al., 2019). The ubiquity of *Klebsiella* strains in natural environments, as well as their underestimated virulence potential, has posed a challenge to the proper identification and classification of *Klebsiella* species (Barrios-Camacho et al., 2019; Rodríguez-Medina et al., 2019). It has been estimated that between 2.5% and 10% of *K. pneumoniae* isolates are misidentified *K. variicola* strains (Rosenblueth et al., 2004; Fontana et al., 2019), which has led to fatal consequences for patients (Seki et al., 2013). This is an example of the significant implications that misidentification can have in epidemiological studies, which highlights the importance of a swift and adequate molecular identification that combines the appropriate computational tools and phenotypic approaches.

We extended our analysis to test data with less quality processed sequences since the majority of available genomes in public databases are unfinished. As of December 2021, only 7% (24,259/343,140) of the bacterial genomes in the RefSeq database were complete. We found that both sketching programs, Mash and Dashing, performed well for draft genomes without even altering the cutoff for the complete genomes analyzed. Mash has been previously reported to also perform well in whole metagenomic comparisons (Dong et al., 2020) and to be very useful in fungal taxonomy (Gostinčar, 2020), along with many other promising applications (Zhou et al., 2020).

## Conclusions

Our results revealed that Mash and Dashing resolve *Klebsiella* species as accurately as FastANI. The isolation source was not found to be related to the species or domain content. Therefore, only sequence similarities seem to define species boundaries so far. Further research on diverse bacterial species should be performed to have a broader perspective of the reliability and performance of these programs.

## Supplemental Information

10.7717/peerj.13784/supp-1 Supplemental Information 1 Hierarchical clustering comparisons calculated using the Baker’s Gamma Index, AUC and OCP calculated for each of the programs: FastANI, Mash Dashing and DNA Signatures with di, tri and tetra-nucleotides. The cluster comparisons show higher similarities between ANI and Mash clustering, followed by Dashing mash and full-mash. The Optimized cutoff values for all programs conclude such similarities in species assignation between ANI, Mash and Dashing.

10.7717/peerj.13784/supp-2 Supplemental Information 2 Overall genomic information of the *Klebsiella* complete and draft datasets. The table shows the identification numbers of all genomes used in this study per assembly type. Type strains of all the species analyzed are included. All the genomic sequences were retrieved from the NCBI RefSeq.

## Additional Information and Declarations

### Data Availability

The following information was supplied regarding data availability: The raw data is available at GitHub: https://github.com/Computational-conSequences/Klebsiella-sorting.
