# Supplementary figures and images for: FastANI, Mash and Dashing equally differentiate between Klebsiella species
Source: PeerJ. 2022 Jul 21;10:e13784. doi: 10.7717/peerj.13784 (PMC9308963; doi:10.7717/peerj.13784)

## Baker's Gamma Index

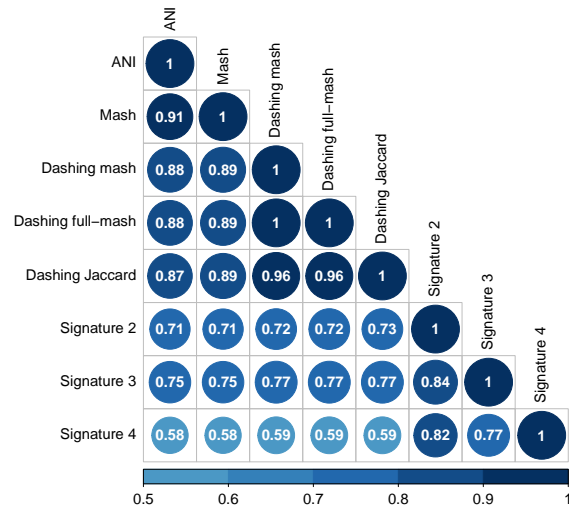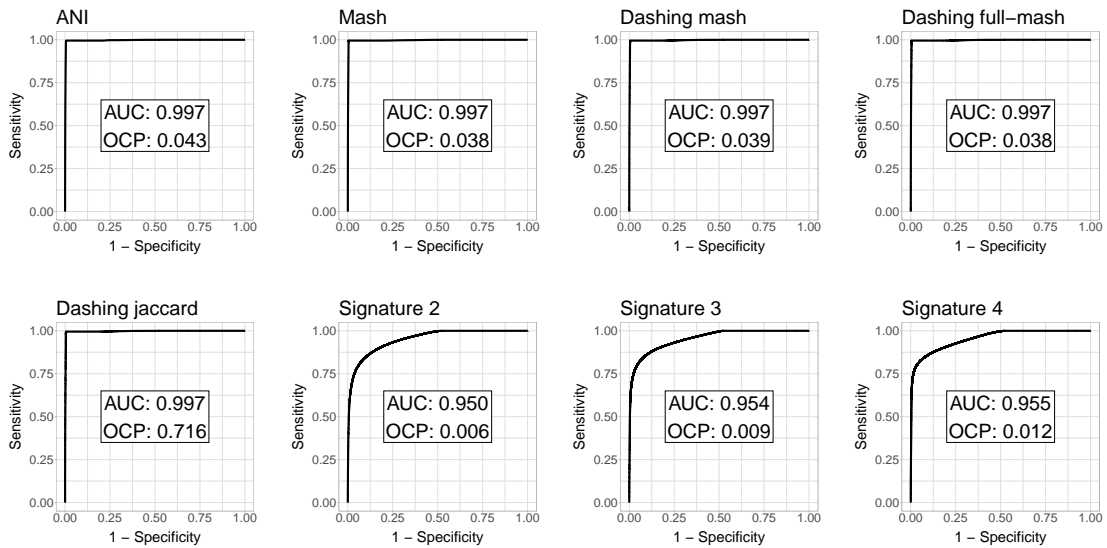

## Optimized cutoff groups

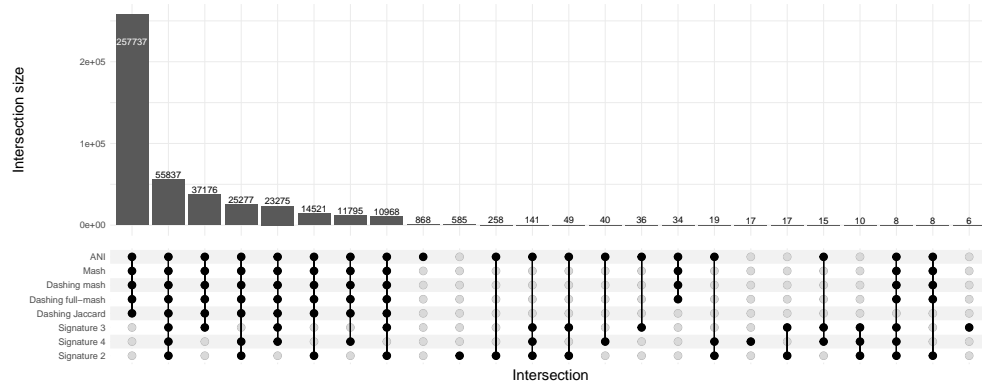

Figure S1.

Supplement: Supplemental Information 1 — The cluster comparisons show higher similarities between ANI and Mash clustering, followed by Dashing mash and full-mash. The Optimized cutoff values for all programs conclude such similarities in species assignation between ANI, Mash and Dashing. [file peerj-10-13784-s001.pdf]
