# Supplementary material for: FastANI, Mash and Dashing equally differentiate between Klebsiella species
Source: PeerJ. 2022 Jul 21;10:e13784. doi: 10.7717/peerj.13784 (PMC9308963; doi:10.7717/peerj.13784)
Supplement: Supplemental Information 2 — The table shows the identification numbers of all genomes used in this study per assembly type. Type strains of all the species analyzed are included. All the genomic sequences were retrieved from the NCBI RefSeq. [file peerj-10-13784-s002.docx]

Genome ID Assembly Status Strain Mash Groups 0.038 Mash Groups 0.043 Mash Groups 0.05 Type

GCF_000009885 Complete Klebsiella pneumoniae pneumoniae NTUH-K2044 Klebsiella pneumoniae 1 Klebsiella pneumoniae 1 Klebsiella pneumoniae 1 NA

GCF_000016305 Complete Klebsiella pneumoniae pneumoniae MGH78578 ATCC700721 Klebsiella pneumoniae 1 Klebsiella pneumoniae 1 Klebsiella pneumoniae 1 NA

GCF_000019565 Complete Klebsiella variicola 342 Klebsiella variicola Klebsiella variicola Klebsiella variicola NA

GCF_000025465 Complete Klebsiella variicola At-22 Klebsiella variicola Klebsiella variicola Klebsiella variicola NA

GCF_000163075 Scaffold Klebsiella variicola 1 55 Klebsiella variicola Klebsiella variicola Klebsiella variicola NA

GCF_000163455 Scaffold Klebsiella pneumoniae rhinoscleromatis ATCC13884 Klebsiella pneumoniae 1 Klebsiella pneumoniae 1 Klebsiella pneumoniae 1 Type strain

GCF_000195655 Scaffold Klebsiella sp MS92-3 Klebsiella pneumoniae 1 Klebsiella pneumoniae 1 Klebsiella pneumoniae 1 NA

GCF_000215745 Complete Klebsiella aerogenes KCTC2190 Klebsiella aerogenes 1 Klebsiella aerogenes Klebsiella aerogenes Type strain

GCF_000219945 Contig Klebsiella pneumoniae JH1 Klebsiella pneumoniae 1 Klebsiella pneumoniae 1 Klebsiella pneumoniae 1 NA

GCF_000219965 Contig Klebsiella pneumoniae 1162281 Klebsiella pneumoniae 1 Klebsiella pneumoniae 1 Klebsiella pneumoniae 1 NA

GCF_000220485 Complete Klebsiella pneumoniae KCTC2242 Klebsiella pneumoniae 1 Klebsiella pneumoniae 1 Klebsiella pneumoniae 1 NA

GCF_000238715 Scaffold Klebsiella sp 4 1 44FAA Klebsiella pneumoniae 1 Klebsiella pneumoniae 1 Klebsiella pneumoniae 1 NA

GCF_000240185 Complete Klebsiella pneumoniae pneumoniae HS11286 Klebsiella pneumoniae 1 Klebsiella pneumoniae 1 Klebsiella pneumoniae 1 NA

GCF_000240325 Complete Klebsiella michiganensis KCTC1686 Klebsiella michiganensis 1 Klebsiella michiganensis 1 Klebsiella michiganensis 1 NA

GCF_000247835 Scaffold Klebsiella michiganensis 10-5242 Klebsiella michiganensis 1 Klebsiella michiganensis 1 Klebsiella michiganensis 1 NA

GCF_000247855 Scaffold Klebsiella oxytoca 10-5243 Klebsiella oxytoca 1 Klebsiella oxytoca 1 Klebsiella oxytoca 1 NA

GCF_000247875 Scaffold Klebsiella oxytoca 10-5245 Klebsiella oxytoca 1 Klebsiella oxytoca 1 Klebsiella oxytoca 1 NA

GCF_000247915 Scaffold Klebsiella michiganensis 10-5250 Klebsiella pasteurii Klebsiella pasteurii Klebsiella grimontii NA

GCF_000252915 Contig Klebsiella oxytoca 11492-1 Klebsiella oxytoca 1 Klebsiella oxytoca 1 Klebsiella oxytoca 1 NA

GCF_000255975 Scaffold Klebsiella pneumoniae pneumoniae LCT-KP214 Klebsiella pneumoniae 1 Klebsiella pneumoniae 1 Klebsiella pneumoniae 1 NA

GCF_000263095 Contig Klebsiella pneumoniae pneumoniae LZ Klebsiella pneumoniae 1 Klebsiella pneumoniae 1 Klebsiella pneumoniae 1 NA

GCF_000276705 Complete Klebsiella michiganensis E718 Klebsiella michiganensis 1 Klebsiella michiganensis 1 Klebsiella michiganensis 1 NA

GCF_000281255 Contig Klebsiella pneumoniae pneumoniae KPNIH2 Klebsiella pneumoniae 1 Klebsiella pneumoniae 1 Klebsiella pneumoniae 1 NA

GCF_000281275 Contig Klebsiella pneumoniae pneumoniae KPNIH5 Klebsiella pneumoniae 1 Klebsiella pneumoniae 1 Klebsiella pneumoniae 1 NA

GCF_000281295 Contig Klebsiella pneumoniae pneumoniae KPNIH6 Klebsiella pneumoniae 1 Klebsiella pneumoniae 1 Klebsiella pneumoniae 1 NA

GCF_000281315 Contig Klebsiella pneumoniae pneumoniae KPNIH9 Klebsiella pneumoniae 1 Klebsiella pneumoniae 1 Klebsiella pneumoniae 1 NA

GCF_000281335 Contig Klebsiella pneumoniae pneumoniae KPNIH12 Klebsiella pneumoniae 1 Klebsiella pneumoniae 1 Klebsiella pneumoniae 1 NA

GCF_000281355 Contig Klebsiella pneumoniae pneumoniae KPNIH17 Klebsiella pneumoniae 1 Klebsiella pneumoniae 1 Klebsiella pneumoniae 1 NA

GCF_000281375 Contig Klebsiella pneumoniae pneumoniae KPNIH20 Klebsiella pneumoniae 1 Klebsiella pneumoniae 1 Klebsiella pneumoniae 1 NA

GCF_000281395 Contig Klebsiella pneumoniae pneumoniae KPNIH4 Klebsiella pneumoniae 1 Klebsiella pneumoniae 1 Klebsiella pneumoniae 1 NA

GCF_000281415 Contig Klebsiella pneumoniae pneumoniae KPNIH7 Klebsiella pneumoniae 1 Klebsiella pneumoniae 1 Klebsiella pneumoniae 1 NA

GCF_000281435 Complete Klebsiella pneumoniae pneumoniae KPNIH10 Klebsiella pneumoniae 1 Klebsiella pneumoniae 1 Klebsiella pneumoniae 1 NA

GCF_000281455 Contig Klebsiella pneumoniae pneumoniae KPNIH14 Klebsiella pneumoniae 1 Klebsiella pneumoniae 1 Klebsiella pneumoniae 1 NA

GCF_000281475 Contig Klebsiella pneumoniae pneumoniae KPNIH18 Klebsiella pneumoniae 1 Klebsiella pneumoniae 1 Klebsiella pneumoniae 1 NA

GCF_000281495 Contig Klebsiella pneumoniae pneumoniae KPNIH21 Klebsiella pneumoniae 1 Klebsiella pneumoniae 1 Klebsiella pneumoniae 1 NA

GCF_000281515 Contig Klebsiella pneumoniae pneumoniae KPNIH22 Klebsiella pneumoniae 1 Klebsiella pneumoniae 1 Klebsiella pneumoniae 1 NA

GCF_000281535 Complete Klebsiella pneumoniae pneumoniae KPNIH1 Klebsiella pneumoniae 1 Klebsiella pneumoniae 1 Klebsiella pneumoniae 1 NA

GCF_000281555 Contig Klebsiella pneumoniae pneumoniae KPNIH8 Klebsiella pneumoniae 1 Klebsiella pneumoniae 1 Klebsiella pneumoniae 1 NA

GCF_000281575 Contig Klebsiella pneumoniae pneumoniae KPNIH11 Klebsiella pneumoniae 1 Klebsiella pneumoniae 1 Klebsiella pneumoniae 1 NA

GCF_000281595 Contig Klebsiella pneumoniae pneumoniae KPNIH16 Klebsiella pneumoniae 1 Klebsiella pneumoniae 1 Klebsiella pneumoniae 1 NA

GCF_000281635 Contig Klebsiella pneumoniae pneumoniae KPNIH23 Klebsiella pneumoniae 1 Klebsiella pneumoniae 1 Klebsiella pneumoniae 1 NA

GCF_000281755 Contig Klebsiella pneumoniae pneumoniae DSM30104 JCM1662 NBRC14940 Klebsiella pneumoniae 1 Klebsiella pneumoniae 1 Klebsiella pneumoniae 1 Type strain

GCF_000283455 Contig Klebsiella pneumoniae pneumoniae ST258-490 Klebsiella pneumoniae 1 Klebsiella pneumoniae 1 Klebsiella pneumoniae 1 NA

GCF_000293135 Contig Klebsiella sp OBRC7 Klebsiella michiganensis 1 Klebsiella michiganensis 1 Klebsiella michiganensis 1 NA

GCF_000294365 Complete Klebsiella pneumoniae pneumoniae 1084 Klebsiella pneumoniae 1 Klebsiella pneumoniae 1 Klebsiella pneumoniae 1 NA

GCF_000300655 Scaffold Klebsiella pneumoniae pneumoniae WGLW1 Klebsiella pneumoniae 1 Klebsiella pneumoniae 1 Klebsiella pneumoniae 1 NA

GCF_000300675 Scaffold Klebsiella pneumoniae pneumoniae WGLW2 Klebsiella pneumoniae 1 Klebsiella pneumoniae 1 Klebsiella pneumoniae 1 NA

GCF_000300735 Scaffold Klebsiella pneumoniae pneumoniae KpO3210 Klebsiella pneumoniae 1 Klebsiella pneumoniae 1 Klebsiella pneumoniae 1 NA

GCF_000300835 Scaffold Klebsiella pneumoniae pneumoniae KpQ3 Klebsiella pneumoniae 1 Klebsiella pneumoniae 1 Klebsiella pneumoniae 1 NA

GCF_000300935 Scaffold Klebsiella pneumoniae pneumoniae WGLW3 Klebsiella pneumoniae 1 Klebsiella pneumoniae 1 Klebsiella pneumoniae 1 NA

GCF_000300955 Scaffold Klebsiella pneumoniae pneumoniae WGLW5 Klebsiella pneumoniae 1 Klebsiella pneumoniae 1 Klebsiella pneumoniae 1 NA

GCF_000309505 Contig Klebsiella pneumoniae pneumoniae ST512-K30BO Klebsiella pneumoniae 1 Klebsiella pneumoniae 1 Klebsiella pneumoniae 1 NA

GCF_000313365 Contig Klebsiella pneumoniae pneumoniae ST258-K28BO Klebsiella pneumoniae 1 Klebsiella pneumoniae 1 Klebsiella pneumoniae 1 NA

GCF_000313465 Contig Klebsiella pneumoniae pneumoniae ST258-K26BO Klebsiella pneumoniae 1 Klebsiella pneumoniae 1 Klebsiella pneumoniae 1 NA

GCF_000315385 Scaffold Klebsiella pneumoniae pneumoniae Ecl8 Klebsiella pneumoniae 1 Klebsiella pneumoniae 1 Klebsiella pneumoniae 1 NA

GCF_000316245 Contig Klebsiella pneumoniae VA360 Klebsiella pneumoniae 1 Klebsiella pneumoniae 1 Klebsiella pneumoniae 1 NA

GCF_000316265 Contig Klebsiella pneumoniae JHCK1 Klebsiella pneumoniae 1 Klebsiella pneumoniae 1 Klebsiella pneumoniae 1 NA

GCF_000333535 Scaffold Klebsiella planticola B43 Klebsiella planticola Klebsiella planticola Klebsiella planticola NA

GCF_000334515 Complete Klebsiella aerogenes EA1509E Klebsiella aerogenes 1 Klebsiella aerogenes Klebsiella aerogenes NA

GCF_000338255 Contig Klebsiella pneumoniae hvKP1 Klebsiella pneumoniae 1 Klebsiella pneumoniae 1 Klebsiella pneumoniae 1 NA

GCF_000349245 Contig Klebsiella quasipneumoniae 700603 Klebsiella quasipneumoniae 1 Klebsiella quasipneumoniae 1 Klebsiella quasipneumoniae NA

GCF_000349265 Contig Klebsiella pneumoniae ATCC BAA1705 Klebsiella pneumoniae 1 Klebsiella pneumoniae 1 Klebsiella pneumoniae 1 NA

GCF_000364385 Complete Klebsiella pneumoniae ATCC BAA2146 Klebsiella pneumoniae 1 Klebsiella pneumoniae 1 Klebsiella pneumoniae 1 NA

GCF_000383335 Scaffold Klebsiella aerogenes FGI35 Klebsiella aerogenes 1 Klebsiella aerogenes Klebsiella aerogenes NA

GCF_000398905 Scaffold Klebsiella sp KTE92 Klebsiella variicola Klebsiella variicola Klebsiella variicola NA

GCF_000401195 Contig Klebsiella pneumoniae UHKPC23 Klebsiella pneumoniae 1 Klebsiella pneumoniae 1 Klebsiella pneumoniae 1 NA

GCF_000406405 Contig Klebsiella pneumoniae UHKPC40 Klebsiella pneumoniae 1 Klebsiella pneumoniae 1 Klebsiella pneumoniae 1 NA

GCF_000406425 Contig Klebsiella pneumoniae UHKPC09 Klebsiella pneumoniae 1 Klebsiella pneumoniae 1 Klebsiella pneumoniae 1 NA

GCF_000406445 Contig Klebsiella pneumoniae UHKPC01 Klebsiella pneumoniae 1 Klebsiella pneumoniae 1 Klebsiella pneumoniae 1 NA

GCF_000406465 Contig Klebsiella pneumoniae UHKPC27 Klebsiella pneumoniae 1 Klebsiella pneumoniae 1 Klebsiella pneumoniae 1 NA

GCF_000406485 Contig Klebsiella pneumoniae 361 1301 Klebsiella pneumoniae 1 Klebsiella pneumoniae 1 Klebsiella pneumoniae 1 NA

GCF_000406525 Contig Klebsiella pneumoniae UHKPC81 Klebsiella pneumoniae 1 Klebsiella pneumoniae 1 Klebsiella pneumoniae 1 NA

GCF_000406545 Contig Klebsiella pneumoniae VAKPC297 Klebsiella pneumoniae 1 Klebsiella pneumoniae 1 Klebsiella pneumoniae 1 NA

GCF_000406565 Contig Klebsiella pneumoniae VAKPC280 Klebsiella pneumoniae 1 Klebsiella pneumoniae 1 Klebsiella pneumoniae 1 NA

GCF_000406585 Contig Klebsiella pneumoniae VAKPC270 Klebsiella pneumoniae 1 Klebsiella pneumoniae 1 Klebsiella pneumoniae 1 NA

GCF_000406605 Contig Klebsiella pneumoniae UHKPC24 Klebsiella pneumoniae 1 Klebsiella pneumoniae 1 Klebsiella pneumoniae 1 NA

GCF_000406625 Contig Klebsiella pneumoniae VAKPC276 Klebsiella pneumoniae 1 Klebsiella pneumoniae 1 Klebsiella pneumoniae 1 NA

GCF_000406645 Contig Klebsiella pneumoniae VAKPC309 Klebsiella pneumoniae 1 Klebsiella pneumoniae 1 Klebsiella pneumoniae 1 NA

GCF_000406665 Contig Klebsiella pneumoniae UHKPC26 Klebsiella pneumoniae 1 Klebsiella pneumoniae 1 Klebsiella pneumoniae 1 NA

GCF_000406685 Contig Klebsiella pneumoniae UHKPC22 Klebsiella pneumoniae 1 Klebsiella pneumoniae 1 Klebsiella pneumoniae 1 NA

GCF_000406705 Contig Klebsiella pneumoniae UHKPC04 Klebsiella pneumoniae 1 Klebsiella pneumoniae 1 Klebsiella pneumoniae 1 NA

GCF_000406725 Contig Klebsiella pneumoniae VAKPC252 Klebsiella pneumoniae 1 Klebsiella pneumoniae 1 Klebsiella pneumoniae 1 NA

GCF_000406745 Contig Klebsiella pneumoniae 440 1540 Klebsiella pneumoniae 1 Klebsiella pneumoniae 1 Klebsiella pneumoniae 1 NA

GCF_000406765 Complete Klebsiella pneumoniae 500 1420 Klebsiella pneumoniae 1 Klebsiella pneumoniae 1 Klebsiella pneumoniae 1 NA

GCF_000406825 Contig Klebsiella pneumoniae VAKPC254 Klebsiella pneumoniae 1 Klebsiella pneumoniae 1 Klebsiella pneumoniae 1 NA

GCF_000406845 Contig Klebsiella pneumoniae VAKPC269 Klebsiella pneumoniae 1 Klebsiella pneumoniae 1 Klebsiella pneumoniae 1 NA

GCF_000406865 Contig Klebsiella pneumoniae 540 1460 Klebsiella pneumoniae 1 Klebsiella pneumoniae 1 Klebsiella pneumoniae 1 NA

GCF_000406885 Contig Klebsiella pneumoniae 646 1568 Klebsiella pneumoniae 1 Klebsiella pneumoniae 1 Klebsiella pneumoniae 1 NA

GCF_000408965 Contig Klebsiella pneumoniae UHKPC05 Klebsiella pneumoniae 1 Klebsiella pneumoniae 1 Klebsiella pneumoniae 1 NA

GCF_000408985 Contig Klebsiella pneumoniae UHKPC52 Klebsiella pneumoniae 1 Klebsiella pneumoniae 1 Klebsiella pneumoniae 1 NA

GCF_000409005 Contig Klebsiella pneumoniae UHKPC45 Klebsiella pneumoniae 1 Klebsiella pneumoniae 1 Klebsiella pneumoniae 1 NA

GCF_000409025 Contig Klebsiella pneumoniae VAKPC278 Klebsiella pneumoniae 1 Klebsiella pneumoniae 1 Klebsiella pneumoniae 1 NA

GCF_000409045 Contig Klebsiella pneumoniae UHKPC29 Klebsiella pneumoniae 1 Klebsiella pneumoniae 1 Klebsiella pneumoniae 1 NA

GCF_000409065 Contig Klebsiella pneumoniae DMC0526 Klebsiella pneumoniae 1 Klebsiella pneumoniae 1 Klebsiella pneumoniae 1 NA

GCF_000409085 Contig Klebsiella pneumoniae UHKPC48 Klebsiella pneumoniae 1 Klebsiella pneumoniae 1 Klebsiella pneumoniae 1 NA

GCF_000409105 Contig Klebsiella pneumoniae UHKPC32 Klebsiella pneumoniae 1 Klebsiella pneumoniae 1 Klebsiella pneumoniae 1 NA

GCF_000409125 Contig Klebsiella pneumoniae UHKPC57 Klebsiella pneumoniae 1 Klebsiella pneumoniae 1 Klebsiella pneumoniae 1 NA

GCF_000409715 Contig Klebsiella pneumoniae ATCC25955 Klebsiella pneumoniae 1 Klebsiella pneumoniae 1 Klebsiella pneumoniae 1 NA

GCF_000409735 Contig Klebsiella pneumoniae G5-2 Klebsiella pneumoniae 1 Klebsiella pneumoniae 1 Klebsiella pneumoniae 1 NA

GCF_000412575 Contig Klebsiella pneumoniae pneumoniae CIP52145 B5055 Klebsiella pneumoniae 1 Klebsiella pneumoniae 1 Klebsiella pneumoniae 1 NA

GCF_000417045 Contig Klebsiella pneumoniae UHKPC59 Klebsiella pneumoniae 1 Klebsiella pneumoniae 1 Klebsiella pneumoniae 1 NA

GCF_000417065 Contig Klebsiella pneumoniae UHKPC61 Klebsiella pneumoniae 1 Klebsiella pneumoniae 1 Klebsiella pneumoniae 1 NA

GCF_000417105 Contig Klebsiella pneumoniae UHKPC28 Klebsiella pneumoniae 1 Klebsiella pneumoniae 1 Klebsiella pneumoniae 1 NA

GCF_000417125 Contig Klebsiella pneumoniae UHKPC47 Klebsiella pneumoniae 1 Klebsiella pneumoniae 1 Klebsiella pneumoniae 1 NA

GCF_000417145 Contig Klebsiella pneumoniae UHKPC02 Klebsiella pneumoniae 1 Klebsiella pneumoniae 1 Klebsiella pneumoniae 1 NA

GCF_000417165 Contig Klebsiella pneumoniae UHKPC31 Klebsiella pneumoniae 1 Klebsiella pneumoniae 1 Klebsiella pneumoniae 1 NA

GCF_000417185 Contig Klebsiella pneumoniae UHKPC06 Klebsiella pneumoniae 1 Klebsiella pneumoniae 1 Klebsiella pneumoniae 1 NA

GCF_000417205 Contig Klebsiella pneumoniae DMC1316 Klebsiella pneumoniae 1 Klebsiella pneumoniae 1 Klebsiella pneumoniae 1 NA

GCF_000417225 Complete Klebsiella pneumoniae DMC1097 Klebsiella pneumoniae 1 Klebsiella pneumoniae 1 Klebsiella pneumoniae 1 NA

GCF_000417245 Contig Klebsiella pneumoniae UHKPC18 Klebsiella pneumoniae 1 Klebsiella pneumoniae 1 Klebsiella pneumoniae 1 NA

GCF_000417265 Complete Klebsiella pneumoniae UHKPC07 Klebsiella pneumoniae 1 Klebsiella pneumoniae 1 Klebsiella pneumoniae 1 NA

GCF_000417305 Contig Klebsiella pneumoniae UHKPC69 Klebsiella pneumoniae 1 Klebsiella pneumoniae 1 Klebsiella pneumoniae 1 NA

GCF_000417325 Contig Klebsiella pneumoniae UHKPC77 Klebsiella pneumoniae 1 Klebsiella pneumoniae 1 Klebsiella pneumoniae 1 NA

GCF_000417345 Contig Klebsiella pneumoniae UHKPC96 Klebsiella pneumoniae 1 Klebsiella pneumoniae 1 Klebsiella pneumoniae 1 NA

GCF_000417365 Contig Klebsiella pneumoniae 160 1080 Klebsiella pneumoniae 1 Klebsiella pneumoniae 1 Klebsiella pneumoniae 1 NA

GCF_000417385 Contig Klebsiella pneumoniae 120 1020 Klebsiella pneumoniae 1 Klebsiella pneumoniae 1 Klebsiella pneumoniae 1 NA

GCF_000417405 Contig Klebsiella pneumoniae 140 1040 Klebsiella pneumoniae 1 Klebsiella pneumoniae 1 Klebsiella pneumoniae 1 NA

GCF_000417425 Contig Klebsiella pneumoniae 280 1220 Klebsiella pneumoniae 1 Klebsiella pneumoniae 1 Klebsiella pneumoniae 1 NA

GCF_000417445 Contig Klebsiella pneumoniae UHKPC67 Klebsiella pneumoniae 1 Klebsiella pneumoniae 1 Klebsiella pneumoniae 1 NA

GCF_000417465 Contig Klebsiella pneumoniae UHKPC179 Klebsiella pneumoniae 1 Klebsiella pneumoniae 1 Klebsiella pneumoniae 1 NA

GCF_000417485 Contig Klebsiella pneumoniae DMC0799 Klebsiella pneumoniae 1 Klebsiella pneumoniae 1 Klebsiella pneumoniae 1 NA

GCF_000417545 Contig Klebsiella pneumoniae UHKPC17 Klebsiella pneumoniae 1 Klebsiella pneumoniae 1 Klebsiella pneumoniae 1 NA

GCF_000427015 Contig Klebsiella michiganensis SA2 Klebsiella grimontii Klebsiella grimontii Klebsiella grimontii NA

GCF_000439165 Contig Klebsiella pneumoniae pneumoniae MP14 Klebsiella pneumoniae 1 Klebsiella pneumoniae 1 Klebsiella pneumoniae 1 NA

GCF_000439185 Scaffold Klebsiella pneumoniae pneumoniae UKKV901664 Klebsiella pneumoniae 1 Klebsiella pneumoniae 1 Klebsiella pneumoniae 1 NA

GCF_000441835 Scaffold Klebsiella pneumoniae LCT-KP182 Klebsiella pneumoniae 1 Klebsiella pneumoniae 1 Klebsiella pneumoniae 1 NA

GCF_000441855 Scaffold Klebsiella pneumoniae LCT-KP289 Klebsiella pneumoniae 1 Klebsiella pneumoniae 1 Klebsiella pneumoniae 1 NA

GCF_000444855 Contig Klebsiella pneumoniae EGD-HP19-C Klebsiella pneumoniae 1 Klebsiella pneumoniae 1 Klebsiella pneumoniae 1 NA

GCF_000465975 Complete Klebsiella pneumoniae KP1 Klebsiella pneumoniae 1 Klebsiella pneumoniae 1 Klebsiella pneumoniae 1 NA

GCF_000474015 Complete Klebsiella pneumoniae CG43 Klebsiella pneumoniae 1 Klebsiella pneumoniae 1 Klebsiella pneumoniae 1 NA

GCF_000474635 Contig Klebsiella pneumoniae 303K Klebsiella pneumoniae 1 Klebsiella pneumoniae 1 Klebsiella pneumoniae 1 NA

GCF_000474865 Contig Klebsiella pneumoniae BIDMC18C Klebsiella pneumoniae 1 Klebsiella pneumoniae 1 Klebsiella pneumoniae 1 NA

GCF_000474885 Contig Klebsiella pneumoniae BIDMC12C Klebsiella pneumoniae 1 Klebsiella pneumoniae 1 Klebsiella pneumoniae 1 NA

GCF_000474905 Contig Klebsiella pneumoniae BIDMC16 Klebsiella pneumoniae 1 Klebsiella pneumoniae 1 Klebsiella pneumoniae 1 NA

GCF_000485755 Scaffold Klebsiella pneumoniae 909957 Klebsiella pneumoniae 1 Klebsiella pneumoniae 1 Klebsiella pneumoniae 1 NA

GCF_000492195 Scaffold Klebsiella pneumoniae BIDMC41 Klebsiella pneumoniae 1 Klebsiella pneumoniae 1 Klebsiella pneumoniae 1 NA

GCF_000492215 Scaffold Klebsiella pneumoniae BIDMC40 Klebsiella pneumoniae 1 Klebsiella pneumoniae 1 Klebsiella pneumoniae 1 NA

GCF_000492295 Scaffold Klebsiella pneumoniae BIDMC36 Klebsiella pneumoniae 1 Klebsiella pneumoniae 1 Klebsiella pneumoniae 1 NA

GCF_000492315 Scaffold Klebsiella pneumoniae BIDMC25 Klebsiella pneumoniae 1 Klebsiella pneumoniae 1 Klebsiella pneumoniae 1 NA

GCF_000492335 Scaffold Klebsiella pneumoniae BIDMC24 Klebsiella pneumoniae 1 Klebsiella pneumoniae 1 Klebsiella pneumoniae 1 NA

GCF_000492355 Scaffold Klebsiella pneumoniae BIDMC23 Klebsiella pneumoniae 1 Klebsiella pneumoniae 1 Klebsiella pneumoniae 1 NA

GCF_000492375 Scaffold Klebsiella pneumoniae BIDMC22 Klebsiella pneumoniae 1 Klebsiella pneumoniae 1 Klebsiella pneumoniae 1 NA

GCF_000492395 Scaffold Klebsiella pneumoniae BIDMC21 Klebsiella pneumoniae 1 Klebsiella pneumoniae 1 Klebsiella pneumoniae 1 NA

GCF_000492415 Scaffold Klebsiella quasipneumoniae quasipneumoniae UCICRE14 Klebsiella quasipneumoniae 2 Klebsiella quasipneumoniae 2 Klebsiella quasipneumoniae NA

GCF_000492475 Scaffold Klebsiella variicola UCICRE10 Klebsiella variicola Klebsiella variicola Klebsiella variicola NA

GCF_000492515 Scaffold Klebsiella pneumoniae UCICRE8 Klebsiella pneumoniae 1 Klebsiella pneumoniae 1 Klebsiella pneumoniae 1 NA

GCF_000492535 Scaffold Klebsiella pneumoniae UCICRE7 Klebsiella pneumoniae 1 Klebsiella pneumoniae 1 Klebsiella pneumoniae 1 NA

GCF_000492555 Scaffold Klebsiella pneumoniae UCICRE6 Klebsiella pneumoniae 1 Klebsiella pneumoniae 1 Klebsiella pneumoniae 1 NA

GCF_000492595 Scaffold Klebsiella pneumoniae UCICRE4 Klebsiella pneumoniae 1 Klebsiella pneumoniae 1 Klebsiella pneumoniae 1 NA

GCF_000492635 Scaffold Klebsiella pneumoniae UCICRE2 Klebsiella pneumoniae 1 Klebsiella pneumoniae 1 Klebsiella pneumoniae 1 NA

GCF_000492695 Scaffold Klebsiella pneumoniae BWH30 Klebsiella pneumoniae 1 Klebsiella pneumoniae 1 Klebsiella pneumoniae 1 NA

GCF_000492735 Scaffold Klebsiella pneumoniae BWH28 Klebsiella pneumoniae 1 Klebsiella pneumoniae 1 Klebsiella pneumoniae 1 NA

GCF_000492755 Scaffold Klebsiella pneumoniae MGH48 Klebsiella pneumoniae 1 Klebsiella pneumoniae 1 Klebsiella pneumoniae 1 NA

GCF_000492775 Scaffold Klebsiella pneumoniae MGH46 Klebsiella pneumoniae 1 Klebsiella pneumoniae 1 Klebsiella pneumoniae 1 NA

GCF_000492795 Scaffold Klebsiella quasipneumoniae similipneumoniae MGH44 Klebsiella quasipneumoniae 1 Klebsiella quasipneumoniae 1 Klebsiella quasipneumoniae NA

GCF_000492815 Scaffold Klebsiella oxytoca MGH42 Klebsiella oxytoca 1 Klebsiella oxytoca 1 Klebsiella oxytoca 1 NA

GCF_000492835 Scaffold Klebsiella variicola MGH40 Klebsiella variicola Klebsiella variicola Klebsiella variicola NA

GCF_000492875 Scaffold Klebsiella pneumoniae MGH36 Klebsiella pneumoniae 1 Klebsiella pneumoniae 1 Klebsiella pneumoniae 1 NA

GCF_000492915 Scaffold Klebsiella pneumoniae MGH32 Klebsiella pneumoniae 1 Klebsiella pneumoniae 1 Klebsiella pneumoniae 1 NA

GCF_000492935 Scaffold Klebsiella pneumoniae MGH30 Klebsiella pneumoniae 1 Klebsiella pneumoniae 1 Klebsiella pneumoniae 1 NA

GCF_000492955 Scaffold Klebsiella oxytoca MGH28 Klebsiella oxytoca 1 Klebsiella oxytoca 1 Klebsiella oxytoca 1 NA

GCF_000493075 Scaffold Klebsiella pneumoniae MGH21 Klebsiella pneumoniae 1 Klebsiella pneumoniae 1 Klebsiella pneumoniae 1 NA

GCF_000493095 Scaffold Klebsiella variicola MGH20 Klebsiella variicola Klebsiella variicola Klebsiella variicola NA

GCF_000493115 Scaffold Klebsiella pneumoniae MGH19 Klebsiella pneumoniae 1 Klebsiella pneumoniae 1 Klebsiella pneumoniae 1 NA

GCF_000493135 Scaffold Klebsiella pneumoniae MGH18 Klebsiella pneumoniae 1 Klebsiella pneumoniae 1 Klebsiella pneumoniae 1 NA

GCF_000493155 Scaffold Klebsiella pneumoniae MGH17 Klebsiella pneumoniae 1 Klebsiella pneumoniae 1 Klebsiella pneumoniae 1 NA

GCF_000495875 Scaffold Klebsiella pneumoniae pneumoniae KKBO1 Klebsiella pneumoniae 1 Klebsiella pneumoniae 1 Klebsiella pneumoniae 1 NA

GCF_000495895 Scaffold Klebsiella pneumoniae pneumoniae KKBO4 Klebsiella pneumoniae 1 Klebsiella pneumoniae 1 Klebsiella pneumoniae 1 NA

GCF_000498775 Contig Klebsiella pneumoniae LAU-KP1 Klebsiella pneumoniae 1 Klebsiella pneumoniae 1 Klebsiella pneumoniae 1 NA

GCF_000499865 Contig Klebsiella pneumoniae pneumoniae KpQ15 Klebsiella pneumoniae 1 Klebsiella pneumoniae 1 Klebsiella pneumoniae 1 NA

GCF_000499885 Contig Klebsiella pneumoniae pneumoniae KpQ24 Klebsiella pneumoniae 1 Klebsiella pneumoniae 1 Klebsiella pneumoniae 1 NA

GCF_000507385 Contig Klebsiella oxytoca BRL6-2 Klebsiella oxytoca 1 Klebsiella oxytoca 1 Klebsiella oxytoca 1 NA

GCF_000512165 Complete Klebsiella pneumoniae pneumoniae Kp13 Klebsiella pneumoniae 1 Klebsiella pneumoniae 1 Klebsiella pneumoniae 1 NA

GCF_000521985 Scaffold Klebsiella pneumoniae BIDMC31 Klebsiella pneumoniae 1 Klebsiella pneumoniae 1 Klebsiella pneumoniae 1 NA

GCF_000523395 Contig Klebsiella sp 10982 Klebsiella quasivariicola 1 Klebsiella quasivariicola Klebsiella quasivariicola NA

GCF_000524315 Contig Klebsiella sp AS10 Klebsiella michiganensis 1 Klebsiella michiganensis 1 Klebsiella michiganensis 1 NA

GCF_000527215 Contig Klebsiella oxytoca KA2 Klebsiella oxytoca 1 Klebsiella oxytoca 1 Klebsiella oxytoca 1 NA

GCF_000527235 Contig Klebsiella oxytoca OK1 Klebsiella oxytoca 1 Klebsiella oxytoca 1 Klebsiella oxytoca 1 NA

GCF_000529645 Scaffold Klebsiella pneumoniae pneumoniae SA1 Klebsiella pneumoniae 1 Klebsiella pneumoniae 1 Klebsiella pneumoniae 1 NA

GCF_000529705 Scaffold Klebsiella pneumoniae pneumoniae BJ1-GA Klebsiella pneumoniae 1 Klebsiella pneumoniae 1 Klebsiella pneumoniae 1 NA

GCF_000529775 Scaffold Klebsiella pneumoniae pneumoniae T69 Klebsiella pneumoniae 1 Klebsiella pneumoniae 1 Klebsiella pneumoniae 1 NA

GCF_000534075 Scaffold Klebsiella aerogenes UCI48 Klebsiella aerogenes 1 Klebsiella aerogenes Klebsiella aerogenes NA

GCF_000534095 Scaffold Klebsiella aerogenes UCI47 Klebsiella aerogenes 1 Klebsiella aerogenes Klebsiella aerogenes NA

GCF_000534115 Scaffold Klebsiella aerogenes UCI46 Klebsiella aerogenes 1 Klebsiella aerogenes Klebsiella aerogenes NA

GCF_000534135 Scaffold Klebsiella aerogenes UCI45 Klebsiella aerogenes 1 Klebsiella aerogenes Klebsiella aerogenes NA

GCF_000534235 Scaffold Klebsiella aerogenes UCI28 Klebsiella aerogenes 1 Klebsiella aerogenes Klebsiella aerogenes NA

GCF_000534255 Scaffold Klebsiella aerogenes UCI27 Klebsiella aerogenes 1 Klebsiella aerogenes Klebsiella aerogenes NA

GCF_000534315 Scaffold Klebsiella aerogenes UCI16 Klebsiella aerogenes 1 Klebsiella aerogenes Klebsiella aerogenes NA

GCF_000534335 Scaffold Klebsiella aerogenes UCI15 Klebsiella aerogenes 1 Klebsiella aerogenes Klebsiella aerogenes NA

GCF_000565135 Contig Klebsiella pneumoniae NB60 Klebsiella pneumoniae 1 Klebsiella pneumoniae 1 Klebsiella pneumoniae 1 NA

GCF_000566725 Scaffold Klebsiella pneumoniae UCI44 Klebsiella pneumoniae 1 Klebsiella pneumoniae 1 Klebsiella pneumoniae 1 NA

GCF_000566745 Scaffold Klebsiella pneumoniae UCI43 Klebsiella pneumoniae 1 Klebsiella pneumoniae 1 Klebsiella pneumoniae 1 NA

GCF_000566765 Scaffold Klebsiella pneumoniae UCI42 Klebsiella pneumoniae 1 Klebsiella pneumoniae 1 Klebsiella pneumoniae 1 NA

GCF_000566785 Scaffold Klebsiella pneumoniae UCI41 Klebsiella pneumoniae 1 Klebsiella pneumoniae 1 Klebsiella pneumoniae 1 NA

GCF_000566805 Scaffold Klebsiella pneumoniae UCI38 Klebsiella pneumoniae 1 Klebsiella pneumoniae 1 Klebsiella pneumoniae 1 NA

GCF_000566825 Scaffold Klebsiella pneumoniae UCI37 Klebsiella pneumoniae 1 Klebsiella pneumoniae 1 Klebsiella pneumoniae 1 NA

GCF_000566845 Scaffold Klebsiella pneumoniae UCI34 Klebsiella pneumoniae 1 Klebsiella pneumoniae 1 Klebsiella pneumoniae 1 NA

GCF_000566865 Scaffold Klebsiella pneumoniae UCI33 Klebsiella pneumoniae 1 Klebsiella pneumoniae 1 Klebsiella pneumoniae 1 NA

GCF_000566885 Scaffold Klebsiella pneumoniae UCI26 Klebsiella pneumoniae 1 Klebsiella pneumoniae 1 Klebsiella pneumoniae 1 NA

GCF_000566905 Scaffold Klebsiella pneumoniae UCI25 Klebsiella pneumoniae 1 Klebsiella pneumoniae 1 Klebsiella pneumoniae 1 NA

GCF_000566925 Scaffold Klebsiella pneumoniae UCI22 Klebsiella pneumoniae 1 Klebsiella pneumoniae 1 Klebsiella pneumoniae 1 NA

GCF_000566945 Scaffold Klebsiella pneumoniae UCI21 Klebsiella pneumoniae 1 Klebsiella pneumoniae 1 Klebsiella pneumoniae 1 NA

GCF_000566965 Scaffold Klebsiella pneumoniae UCI20 Klebsiella pneumoniae 1 Klebsiella pneumoniae 1 Klebsiella pneumoniae 1 NA

GCF_000566985 Scaffold Klebsiella pneumoniae UCI19 Klebsiella pneumoniae 1 Klebsiella pneumoniae 1 Klebsiella pneumoniae 1 NA

GCF_000567005 Scaffold Klebsiella variicola UCI18 Klebsiella variicola Klebsiella variicola Klebsiella variicola NA

GCF_000567025 Scaffold Klebsiella pneumoniae BIDMC45 Klebsiella pneumoniae 1 Klebsiella pneumoniae 1 Klebsiella pneumoniae 1 NA

GCF_000567045 Scaffold Klebsiella pneumoniae BIDMC53 Klebsiella pneumoniae 1 Klebsiella pneumoniae 1 Klebsiella pneumoniae 1 NA

GCF_000567065 Scaffold Klebsiella pneumoniae BIDMC52 Klebsiella pneumoniae 1 Klebsiella pneumoniae 1 Klebsiella pneumoniae 1 NA

GCF_000567085 Scaffold Klebsiella pneumoniae BIDMC51 Klebsiella pneumoniae 1 Klebsiella pneumoniae 1 Klebsiella pneumoniae 1 NA

GCF_000567105 Scaffold Klebsiella pneumoniae BIDMC48 Klebsiella pneumoniae 1 Klebsiella pneumoniae 1 Klebsiella pneumoniae 1 NA

GCF_000567125 Scaffold Klebsiella pneumoniae BIDMC47 Klebsiella pneumoniae 1 Klebsiella pneumoniae 1 Klebsiella pneumoniae 1 NA

GCF_000567145 Scaffold Klebsiella pneumoniae BIDMC46b Klebsiella pneumoniae 1 Klebsiella pneumoniae 1 Klebsiella pneumoniae 1 NA

GCF_000567165 Scaffold Klebsiella pneumoniae BIDMC46a Klebsiella pneumoniae 1 Klebsiella pneumoniae 1 Klebsiella pneumoniae 1 NA

GCF_000567185 Scaffold Klebsiella pneumoniae BIDMC42b Klebsiella pneumoniae 1 Klebsiella pneumoniae 1 Klebsiella pneumoniae 1 NA

GCF_000567205 Scaffold Klebsiella pneumoniae BIDMC42a Klebsiella pneumoniae 1 Klebsiella pneumoniae 1 Klebsiella pneumoniae 1 NA

GCF_000567225 Contig Klebsiella pneumoniae BIDMC35 Klebsiella pneumoniae 1 Klebsiella pneumoniae 1 Klebsiella pneumoniae 1 NA

GCF_000567245 Scaffold Klebsiella pneumoniae BIDMC34 Klebsiella pneumoniae 1 Klebsiella pneumoniae 1 Klebsiella pneumoniae 1 NA

GCF_000567265 Scaffold Klebsiella pneumoniae BIDMC32 Klebsiella pneumoniae 1 Klebsiella pneumoniae 1 Klebsiella pneumoniae 1 NA

GCF_000567285 Scaffold Klebsiella pneumoniae BIDMC18D Klebsiella pneumoniae 1 Klebsiella pneumoniae 1 Klebsiella pneumoniae 1 NA

GCF_000567305 Scaffold Klebsiella pneumoniae BIDMC18A Klebsiella pneumoniae 1 Klebsiella pneumoniae 1 Klebsiella pneumoniae 1 NA

GCF_000567325 Scaffold Klebsiella pneumoniae BIDMC14 Klebsiella pneumoniae 1 Klebsiella pneumoniae 1 Klebsiella pneumoniae 1 NA

GCF_000567345 Scaffold Klebsiella pneumoniae BIDMC13 Klebsiella pneumoniae 1 Klebsiella pneumoniae 1 Klebsiella pneumoniae 1 NA

GCF_000567365 Scaffold Klebsiella pneumoniae BIDMC12B Klebsiella pneumoniae 1 Klebsiella pneumoniae 1 Klebsiella pneumoniae 1 NA

GCF_000567385 Scaffold Klebsiella pneumoniae BIDMC12A Klebsiella pneumoniae 1 Klebsiella pneumoniae 1 Klebsiella pneumoniae 1 NA

GCF_000567405 Scaffold Klebsiella pneumoniae BIDMC11 Klebsiella pneumoniae 1 Klebsiella pneumoniae 1 Klebsiella pneumoniae 1 NA

GCF_000567425 Scaffold Klebsiella pneumoniae BIDMC7B Klebsiella pneumoniae 1 Klebsiella pneumoniae 1 Klebsiella pneumoniae 1 NA

GCF_000567445 Scaffold Klebsiella pneumoniae BIDMC5 Klebsiella pneumoniae 1 Klebsiella pneumoniae 1 Klebsiella pneumoniae 1 NA

GCF_000567465 Scaffold Klebsiella pneumoniae BIDMC4 Klebsiella pneumoniae 1 Klebsiella pneumoniae 1 Klebsiella pneumoniae 1 NA

GCF_000567485 Scaffold Klebsiella pneumoniae BIDMC2A Klebsiella pneumoniae 1 Klebsiella pneumoniae 1 Klebsiella pneumoniae 1 NA

GCF_000567505 Scaffold Klebsiella pneumoniae BIDMC1 Klebsiella pneumoniae 1 Klebsiella pneumoniae 1 Klebsiella pneumoniae 1 NA

GCF_000567525 Scaffold Klebsiella pneumoniae UCICRE1 Klebsiella pneumoniae 1 Klebsiella pneumoniae 1 Klebsiella pneumoniae 1 NA

GCF_000567545 Scaffold Klebsiella pneumoniae BWH41 Klebsiella pneumoniae 1 Klebsiella pneumoniae 1 Klebsiella pneumoniae 1 NA

GCF_000567565 Scaffold Klebsiella pneumoniae BWH36 Klebsiella pneumoniae 1 Klebsiella pneumoniae 1 Klebsiella pneumoniae 1 NA

GCF_000567585 Scaffold Klebsiella pneumoniae BWH22 Klebsiella pneumoniae 1 Klebsiella pneumoniae 1 Klebsiella pneumoniae 1 NA

GCF_000567605 Scaffold Klebsiella pneumoniae BWH15 Klebsiella pneumoniae 1 Klebsiella pneumoniae 1 Klebsiella pneumoniae 1 NA

GCF_000567625 Scaffold Klebsiella pneumoniae BWH2 Klebsiella pneumoniae 1 Klebsiella pneumoniae 1 Klebsiella pneumoniae 1 NA

GCF_000567645 Scaffold Klebsiella pneumoniae MGH47 Klebsiella pneumoniae 1 Klebsiella pneumoniae 1 Klebsiella pneumoniae 1 NA

GCF_000567665 Scaffold Klebsiella pneumoniae MGH45 Klebsiella pneumoniae 1 Klebsiella pneumoniae 1 Klebsiella pneumoniae 1 NA

GCF_000567685 Scaffold Klebsiella pneumoniae MGH43 Klebsiella pneumoniae 1 Klebsiella pneumoniae 1 Klebsiella pneumoniae 1 NA

GCF_000567705 Scaffold Klebsiella michiganensis MGH41 Klebsiella michiganensis 1 Klebsiella michiganensis 1 Klebsiella michiganensis 1 NA

GCF_000567745 Scaffold Klebsiella pneumoniae MGH35 Klebsiella pneumoniae 1 Klebsiella pneumoniae 1 Klebsiella pneumoniae 1 NA

GCF_000567765 Contig Klebsiella pneumoniae MGH31 Klebsiella pneumoniae 1 Klebsiella pneumoniae 1 Klebsiella pneumoniae 1 NA

GCF_000567785 Scaffold Klebsiella pneumoniae MGH29 Klebsiella pneumoniae 1 Klebsiella pneumoniae 1 Klebsiella pneumoniae 1 NA

GCF_000567805 Scaffold Klebsiella michiganensis MGH27 Klebsiella michiganensis 1 Klebsiella michiganensis 1 Klebsiella michiganensis 1 NA

GCF_000567825 Scaffold Klebsiella pneumoniae BIDMC7A Klebsiella pneumoniae 1 Klebsiella pneumoniae 1 Klebsiella pneumoniae 1 NA

GCF_000567845 Scaffold Klebsiella pneumoniae UCICRE13 Klebsiella pneumoniae 1 Klebsiella pneumoniae 1 Klebsiella pneumoniae 1 NA

GCF_000585875 Contig Klebsiella pneumoniae KP3-S Klebsiella pneumoniae 1 Klebsiella pneumoniae 1 Klebsiella pneumoniae 1 NA

GCF_000585895 Contig Klebsiella pneumoniae KP4-R Klebsiella pneumoniae 1 Klebsiella pneumoniae 1 Klebsiella pneumoniae 1 NA

GCF_000585915 Contig Klebsiella pneumoniae KP2-R Klebsiella pneumoniae 1 Klebsiella pneumoniae 1 Klebsiella pneumoniae 1 NA

GCF_000585935 Contig Klebsiella pneumoniae KP5-R Klebsiella pneumoniae 1 Klebsiella pneumoniae 1 Klebsiella pneumoniae 1 NA

GCF_000585955 Contig Klebsiella pneumoniae KP1-I Klebsiella pneumoniae 1 Klebsiella pneumoniae 1 Klebsiella pneumoniae 1 NA

GCF_000597905 Complete Klebsiella pneumoniae 30684/NJST258 2 Klebsiella pneumoniae 1 Klebsiella pneumoniae 1 Klebsiella pneumoniae 1 NA

GCF_000598005 Complete Klebsiella pneumoniae 30660/NJST258 1 Klebsiella pneumoniae 1 Klebsiella pneumoniae 1 Klebsiella pneumoniae 1 NA

GCF_000599905 Contig Klebsiella pneumoniae Kb677 Klebsiella pneumoniae 1 Klebsiella pneumoniae 1 Klebsiella pneumoniae 1 NA

GCF_000599925 Contig Klebsiella pneumoniae Kb140 Klebsiella pneumoniae 1 Klebsiella pneumoniae 1 Klebsiella pneumoniae 1 NA

GCF_000607265 Scaffold Klebsiella oxytoca G54 Klebsiella oxytoca 1 Klebsiella oxytoca 1 Klebsiella oxytoca 1 NA

GCF_000613225 Contig Klebsiella quasipneumoniae similipneumoniae 07A044 Klebsiella quasipneumoniae 1 Klebsiella quasipneumoniae 1 Klebsiella quasipneumoniae Type strain

GCF_000632415 Complete Klebsiella michiganensis HKOPL1 Klebsiella michiganensis 1 Klebsiella michiganensis 1 Klebsiella michiganensis 1 NA

GCF_000633235 Contig Klebsiella michiganensis H1g Klebsiella michiganensis 1 Klebsiella michiganensis 1 Klebsiella michiganensis 1 NA

GCF_000633735 Scaffold Klebsiella pneumoniae CHS60 Klebsiella pneumoniae 1 Klebsiella pneumoniae 1 Klebsiella pneumoniae 1 NA

GCF_000633755 Scaffold Klebsiella pneumoniae CHS47 Klebsiella pneumoniae 1 Klebsiella pneumoniae 1 Klebsiella pneumoniae 1 NA

GCF_000633775 Scaffold Klebsiella pneumoniae CHS42 Klebsiella pneumoniae 1 Klebsiella pneumoniae 1 Klebsiella pneumoniae 1 NA

GCF_000688155 Contig Klebsiella pneumoniae MGH63 Klebsiella pneumoniae 1 Klebsiella pneumoniae 1 Klebsiella pneumoniae 1 NA

GCF_000688175 Contig Klebsiella pneumoniae UCI64 Klebsiella pneumoniae 1 Klebsiella pneumoniae 1 Klebsiella pneumoniae 1 NA

GCF_000688195 Contig Klebsiella pneumoniae UCI60 Klebsiella pneumoniae 1 Klebsiella pneumoniae 1 Klebsiella pneumoniae 1 NA

GCF_000688215 Contig Klebsiella pneumoniae MGH75 Klebsiella pneumoniae 1 Klebsiella pneumoniae 1 Klebsiella pneumoniae 1 NA

GCF_000688535 Contig Klebsiella pneumoniae CHS21 Klebsiella pneumoniae 1 Klebsiella pneumoniae 1 Klebsiella pneumoniae 1 NA

GCF_000688555 Contig Klebsiella pneumoniae CHS16 Klebsiella pneumoniae 1 Klebsiella pneumoniae 1 Klebsiella pneumoniae 1 NA

GCF_000688635 Contig Klebsiella pneumoniae CHS08 Klebsiella pneumoniae 1 Klebsiella pneumoniae 1 Klebsiella pneumoniae 1 NA

GCF_000692155 Scaffold Klebsiella aerogenes MGH61 Klebsiella aerogenes 1 Klebsiella aerogenes Klebsiella aerogenes NA

GCF_000692175 Scaffold Klebsiella aerogenes MGH62 Klebsiella aerogenes 1 Klebsiella aerogenes Klebsiella aerogenes NA

GCF_000692195 Scaffold Klebsiella aerogenes MGH77 Klebsiella aerogenes 1 Klebsiella aerogenes Klebsiella aerogenes NA

GCF_000692215 Scaffold Klebsiella aerogenes MGH78 Klebsiella aerogenes 1 Klebsiella aerogenes Klebsiella aerogenes NA

GCF_000692915 Scaffold Klebsiella pneumoniae BIDMC18B Klebsiella pneumoniae 1 Klebsiella pneumoniae 1 Klebsiella pneumoniae 1 NA

GCF_000692935 Scaffold Klebsiella pneumoniae BIDMC54 Klebsiella pneumoniae 1 Klebsiella pneumoniae 1 Klebsiella pneumoniae 1 NA

GCF_000692955 Scaffold Klebsiella pneumoniae BIDMC55 Klebsiella pneumoniae 1 Klebsiella pneumoniae 1 Klebsiella pneumoniae 1 NA

GCF_000692975 Scaffold Klebsiella pneumoniae BIDMC60 Klebsiella pneumoniae 1 Klebsiella pneumoniae 1 Klebsiella pneumoniae 1 NA

GCF_000692995 Scaffold Klebsiella variicola BIDMC61 Klebsiella variicola Klebsiella variicola Klebsiella variicola NA

GCF_000693015 Scaffold Klebsiella pneumoniae BIDMC68 Klebsiella pneumoniae 1 Klebsiella pneumoniae 1 Klebsiella pneumoniae 1 NA

GCF_000693035 Scaffold Klebsiella pneumoniae BIDMC69 Klebsiella pneumoniae 1 Klebsiella pneumoniae 1 Klebsiella pneumoniae 1 NA

GCF_000693055 Scaffold Klebsiella pneumoniae BWH45 Klebsiella pneumoniae 1 Klebsiella pneumoniae 1 Klebsiella pneumoniae 1 NA

GCF_000693075 Scaffold Klebsiella pneumoniae BWH46 Klebsiella pneumoniae 1 Klebsiella pneumoniae 1 Klebsiella pneumoniae 1 NA

GCF_000693095 Scaffold Klebsiella pneumoniae BWH47 Klebsiella pneumoniae 1 Klebsiella pneumoniae 1 Klebsiella pneumoniae 1 NA

GCF_000693115 Scaffold Klebsiella pneumoniae BWH48 Klebsiella pneumoniae 1 Klebsiella pneumoniae 1 Klebsiella pneumoniae 1 NA

GCF_000693135 Scaffold Klebsiella pneumoniae CHS02 Klebsiella pneumoniae 1 Klebsiella pneumoniae 1 Klebsiella pneumoniae 1 NA

GCF_000693155 Scaffold Klebsiella pneumoniae CHS03 Klebsiella pneumoniae 1 Klebsiella pneumoniae 1 Klebsiella pneumoniae 1 NA

GCF_000693175 Scaffold Klebsiella pneumoniae CHS05 Klebsiella pneumoniae 1 Klebsiella pneumoniae 1 Klebsiella pneumoniae 1 NA

GCF_000693195 Scaffold Klebsiella pneumoniae CHS06 Klebsiella pneumoniae 1 Klebsiella pneumoniae 1 Klebsiella pneumoniae 1 NA

GCF_000693215 Scaffold Klebsiella pneumoniae CHS07 Klebsiella pneumoniae 1 Klebsiella pneumoniae 1 Klebsiella pneumoniae 1 NA

GCF_000693235 Scaffold Klebsiella pneumoniae CHS09 Klebsiella pneumoniae 1 Klebsiella pneumoniae 1 Klebsiella pneumoniae 1 NA

GCF_000693255 Scaffold Klebsiella pneumoniae CHS11 Klebsiella pneumoniae 1 Klebsiella pneumoniae 1 Klebsiella pneumoniae 1 NA

GCF_000693275 Scaffold Klebsiella pneumoniae CHS12 Klebsiella pneumoniae 1 Klebsiella pneumoniae 1 Klebsiella pneumoniae 1 NA

GCF_000693295 Scaffold Klebsiella pneumoniae CHS13 Klebsiella pneumoniae 1 Klebsiella pneumoniae 1 Klebsiella pneumoniae 1 NA

GCF_000693315 Scaffold Klebsiella pneumoniae CHS14 Klebsiella pneumoniae 1 Klebsiella pneumoniae 1 Klebsiella pneumoniae 1 NA

GCF_000693335 Scaffold Klebsiella pneumoniae CHS17 Klebsiella pneumoniae 1 Klebsiella pneumoniae 1 Klebsiella pneumoniae 1 NA

GCF_000693355 Scaffold Klebsiella pneumoniae CHS18 Klebsiella pneumoniae 1 Klebsiella pneumoniae 1 Klebsiella pneumoniae 1 NA

GCF_000693375 Scaffold Klebsiella pneumoniae CHS19 Klebsiella pneumoniae 1 Klebsiella pneumoniae 1 Klebsiella pneumoniae 1 NA

GCF_000693395 Scaffold Klebsiella pneumoniae CHS20 Klebsiella pneumoniae 1 Klebsiella pneumoniae 1 Klebsiella pneumoniae 1 NA

GCF_000693415 Scaffold Klebsiella pneumoniae CHS22 Klebsiella pneumoniae 1 Klebsiella pneumoniae 1 Klebsiella pneumoniae 1 NA

GCF_000693435 Scaffold Klebsiella pneumoniae CHS23 Klebsiella pneumoniae 1 Klebsiella pneumoniae 1 Klebsiella pneumoniae 1 NA

GCF_000693455 Scaffold Klebsiella pneumoniae CHS24 Klebsiella pneumoniae 1 Klebsiella pneumoniae 1 Klebsiella pneumoniae 1 NA

GCF_000693475 Scaffold Klebsiella pneumoniae CHS25 Klebsiella pneumoniae 1 Klebsiella pneumoniae 1 Klebsiella pneumoniae 1 NA

GCF_000693495 Scaffold Klebsiella pneumoniae CHS26 Klebsiella pneumoniae 1 Klebsiella pneumoniae 1 Klebsiella pneumoniae 1 NA

GCF_000693515 Scaffold Klebsiella pneumoniae CHS27 Klebsiella pneumoniae 1 Klebsiella pneumoniae 1 Klebsiella pneumoniae 1 NA

GCF_000693535 Scaffold Klebsiella pneumoniae CHS28 Klebsiella pneumoniae 1 Klebsiella pneumoniae 1 Klebsiella pneumoniae 1 NA

GCF_000693555 Scaffold Klebsiella pneumoniae CHS29 Klebsiella pneumoniae 1 Klebsiella pneumoniae 1 Klebsiella pneumoniae 1 NA

GCF_000693575 Scaffold Klebsiella pneumoniae CHS30 Klebsiella pneumoniae 1 Klebsiella pneumoniae 1 Klebsiella pneumoniae 1 NA

GCF_000693595 Scaffold Klebsiella pneumoniae CHS31 Klebsiella pneumoniae 1 Klebsiella pneumoniae 1 Klebsiella pneumoniae 1 NA

GCF_000693615 Scaffold Klebsiella pneumoniae CHS32 Klebsiella pneumoniae 1 Klebsiella pneumoniae 1 Klebsiella pneumoniae 1 NA

GCF_000693635 Scaffold Klebsiella pneumoniae CHS33 Klebsiella pneumoniae 1 Klebsiella pneumoniae 1 Klebsiella pneumoniae 1 NA

GCF_000693655 Scaffold Klebsiella pneumoniae CHS34 Klebsiella pneumoniae 1 Klebsiella pneumoniae 1 Klebsiella pneumoniae 1 NA

GCF_000693675 Scaffold Klebsiella pneumoniae CHS35 Klebsiella pneumoniae 1 Klebsiella pneumoniae 1 Klebsiella pneumoniae 1 NA

GCF_000693695 Scaffold Klebsiella pneumoniae CHS36 Klebsiella pneumoniae 1 Klebsiella pneumoniae 1 Klebsiella pneumoniae 1 NA

GCF_000693715 Scaffold Klebsiella pneumoniae CHS37 Klebsiella pneumoniae 1 Klebsiella pneumoniae 1 Klebsiella pneumoniae 1 NA

GCF_000693735 Scaffold Klebsiella pneumoniae CHS38 Klebsiella pneumoniae 1 Klebsiella pneumoniae 1 Klebsiella pneumoniae 1 NA

GCF_000693755 Scaffold Klebsiella pneumoniae CHS39 Klebsiella pneumoniae 1 Klebsiella pneumoniae 1 Klebsiella pneumoniae 1 NA

GCF_000693775 Scaffold Klebsiella pneumoniae CHS40 Klebsiella pneumoniae 1 Klebsiella pneumoniae 1 Klebsiella pneumoniae 1 NA

GCF_000693795 Scaffold Klebsiella pneumoniae CHS41 Klebsiella pneumoniae 1 Klebsiella pneumoniae 1 Klebsiella pneumoniae 1 NA

GCF_000693815 Scaffold Klebsiella pneumoniae CHS43 Klebsiella pneumoniae 1 Klebsiella pneumoniae 1 Klebsiella pneumoniae 1 NA

GCF_000693835 Scaffold Klebsiella pneumoniae CHS44 Klebsiella pneumoniae 1 Klebsiella pneumoniae 1 Klebsiella pneumoniae 1 NA

GCF_000693855 Scaffold Klebsiella pneumoniae CHS45 Klebsiella pneumoniae 1 Klebsiella pneumoniae 1 Klebsiella pneumoniae 1 NA

GCF_000693875 Scaffold Klebsiella pneumoniae CHS46 Klebsiella pneumoniae 1 Klebsiella pneumoniae 1 Klebsiella pneumoniae 1 NA

GCF_000693895 Scaffold Klebsiella pneumoniae CHS48 Klebsiella pneumoniae 1 Klebsiella pneumoniae 1 Klebsiella pneumoniae 1 NA

GCF_000693915 Scaffold Klebsiella pneumoniae CHS49 Klebsiella pneumoniae 1 Klebsiella pneumoniae 1 Klebsiella pneumoniae 1 NA

GCF_000693935 Scaffold Klebsiella pneumoniae CHS50 Klebsiella pneumoniae 1 Klebsiella pneumoniae 1 Klebsiella pneumoniae 1 NA

GCF_000693955 Scaffold Klebsiella pneumoniae CHS51 Klebsiella pneumoniae 1 Klebsiella pneumoniae 1 Klebsiella pneumoniae 1 NA

GCF_000693975 Scaffold Klebsiella pneumoniae CHS52 Klebsiella pneumoniae 1 Klebsiella pneumoniae 1 Klebsiella pneumoniae 1 NA

GCF_000693995 Scaffold Klebsiella pneumoniae CHS53 Klebsiella pneumoniae 1 Klebsiella pneumoniae 1 Klebsiella pneumoniae 1 NA

GCF_000694015 Scaffold Klebsiella pneumoniae CHS54 Klebsiella pneumoniae 1 Klebsiella pneumoniae 1 Klebsiella pneumoniae 1 NA

GCF_000694035 Scaffold Klebsiella pneumoniae CHS55 Klebsiella pneumoniae 1 Klebsiella pneumoniae 1 Klebsiella pneumoniae 1 NA

GCF_000694055 Scaffold Klebsiella pneumoniae CHS56 Klebsiella pneumoniae 1 Klebsiella pneumoniae 1 Klebsiella pneumoniae 1 NA

GCF_000694075 Scaffold Klebsiella pneumoniae CHS57 Klebsiella pneumoniae 1 Klebsiella pneumoniae 1 Klebsiella pneumoniae 1 NA

GCF_000694095 Scaffold Klebsiella pneumoniae CHS58 Klebsiella pneumoniae 1 Klebsiella pneumoniae 1 Klebsiella pneumoniae 1 NA

GCF_000694115 Scaffold Klebsiella pneumoniae CHS59 Klebsiella pneumoniae 1 Klebsiella pneumoniae 1 Klebsiella pneumoniae 1 NA

GCF_000694135 Scaffold Klebsiella pneumoniae CHS61 Klebsiella pneumoniae 1 Klebsiella pneumoniae 1 Klebsiella pneumoniae 1 NA

GCF_000694155 Scaffold Klebsiella pneumoniae CHS62 Klebsiella pneumoniae 1 Klebsiella pneumoniae 1 Klebsiella pneumoniae 1 NA

GCF_000694175 Scaffold Klebsiella pneumoniae CHS63 Klebsiella pneumoniae 1 Klebsiella pneumoniae 1 Klebsiella pneumoniae 1 NA

GCF_000694195 Scaffold Klebsiella pneumoniae CHS64 Klebsiella pneumoniae 1 Klebsiella pneumoniae 1 Klebsiella pneumoniae 1 NA

GCF_000694215 Scaffold Klebsiella pneumoniae CHS65 Klebsiella pneumoniae 1 Klebsiella pneumoniae 1 Klebsiella pneumoniae 1 NA

GCF_000694235 Scaffold Klebsiella pneumoniae CHS66 Klebsiella pneumoniae 1 Klebsiella pneumoniae 1 Klebsiella pneumoniae 1 NA

GCF_000694255 Scaffold Klebsiella pneumoniae CHS67 Klebsiella pneumoniae 1 Klebsiella pneumoniae 1 Klebsiella pneumoniae 1 NA

GCF_000694275 Scaffold Klebsiella pneumoniae CHS70 Klebsiella pneumoniae 1 Klebsiella pneumoniae 1 Klebsiella pneumoniae 1 NA

GCF_000694295 Scaffold Klebsiella pneumoniae CHS71 Klebsiella pneumoniae 1 Klebsiella pneumoniae 1 Klebsiella pneumoniae 1 NA

GCF_000694315 Scaffold Klebsiella pneumoniae CHS72 Klebsiella pneumoniae 1 Klebsiella pneumoniae 1 Klebsiella pneumoniae 1 NA

GCF_000694335 Scaffold Klebsiella pneumoniae CHS73 Klebsiella pneumoniae 1 Klebsiella pneumoniae 1 Klebsiella pneumoniae 1 NA

GCF_000694355 Scaffold Klebsiella pneumoniae CHS74 Klebsiella pneumoniae 1 Klebsiella pneumoniae 1 Klebsiella pneumoniae 1 NA

GCF_000694375 Scaffold Klebsiella pneumoniae CHS75 Klebsiella pneumoniae 1 Klebsiella pneumoniae 1 Klebsiella pneumoniae 1 NA

GCF_000694395 Scaffold Klebsiella pneumoniae CHS76 Klebsiella pneumoniae 1 Klebsiella pneumoniae 1 Klebsiella pneumoniae 1 NA

GCF_000694415 Scaffold Klebsiella pneumoniae CHS80 Klebsiella pneumoniae 1 Klebsiella pneumoniae 1 Klebsiella pneumoniae 1 NA

GCF_000694435 Scaffold Klebsiella pneumoniae MGH51 Klebsiella pneumoniae 1 Klebsiella pneumoniae 1 Klebsiella pneumoniae 1 NA

GCF_000694455 Scaffold Klebsiella pneumoniae MGH52 Klebsiella pneumoniae 1 Klebsiella pneumoniae 1 Klebsiella pneumoniae 1 NA

GCF_000694475 Contig Klebsiella pneumoniae MGH59 Klebsiella pneumoniae 1 Klebsiella pneumoniae 1 Klebsiella pneumoniae 1 NA

GCF_000694495 Scaffold Klebsiella pneumoniae MGH60 Klebsiella pneumoniae 1 Klebsiella pneumoniae 1 Klebsiella pneumoniae 1 NA

GCF_000694515 Scaffold Klebsiella pneumoniae MGH64 Klebsiella pneumoniae 1 Klebsiella pneumoniae 1 Klebsiella pneumoniae 1 NA

GCF_000694535 Scaffold Klebsiella pneumoniae MGH65 Klebsiella pneumoniae 1 Klebsiella pneumoniae 1 Klebsiella pneumoniae 1 NA

GCF_000694555 Scaffold Klebsiella pneumoniae MGH66 Klebsiella pneumoniae 1 Klebsiella pneumoniae 1 Klebsiella pneumoniae 1 NA

GCF_000694575 Scaffold Klebsiella pneumoniae MGH67 Klebsiella pneumoniae 1 Klebsiella pneumoniae 1 Klebsiella pneumoniae 1 NA

GCF_000694595 Scaffold Klebsiella variicola MGH68 Klebsiella variicola Klebsiella variicola Klebsiella variicola NA

GCF_000694615 Scaffold Klebsiella pneumoniae MGH69 Klebsiella pneumoniae 1 Klebsiella pneumoniae 1 Klebsiella pneumoniae 1 NA

GCF_000694635 Scaffold Klebsiella pneumoniae MGH70 Klebsiella pneumoniae 1 Klebsiella pneumoniae 1 Klebsiella pneumoniae 1 NA

GCF_000694655 Scaffold Klebsiella pneumoniae MGH71 Klebsiella pneumoniae 1 Klebsiella pneumoniae 1 Klebsiella pneumoniae 1 NA

GCF_000694675 Scaffold Klebsiella pneumoniae MGH72 Klebsiella pneumoniae 1 Klebsiella pneumoniae 1 Klebsiella pneumoniae 1 NA

GCF_000694695 Scaffold Klebsiella pneumoniae MGH73 Klebsiella pneumoniae 1 Klebsiella pneumoniae 1 Klebsiella pneumoniae 1 NA

GCF_000694715 Scaffold Klebsiella pneumoniae MGH74 Klebsiella pneumoniae 1 Klebsiella pneumoniae 1 Klebsiella pneumoniae 1 NA

GCF_000694735 Scaffold Klebsiella variicola MGH76 Klebsiella variicola Klebsiella variicola Klebsiella variicola NA

GCF_000694755 Scaffold Klebsiella pneumoniae MGH79 Klebsiella pneumoniae 1 Klebsiella pneumoniae 1 Klebsiella pneumoniae 1 NA

GCF_000694775 Scaffold Klebsiella variicola MGH80 Klebsiella variicola Klebsiella variicola Klebsiella variicola NA

GCF_000694795 Scaffold Klebsiella pneumoniae UCI55 Klebsiella pneumoniae 1 Klebsiella pneumoniae 1 Klebsiella pneumoniae 1 NA

GCF_000694815 Scaffold Klebsiella pneumoniae UCI56 Klebsiella pneumoniae 1 Klebsiella pneumoniae 1 Klebsiella pneumoniae 1 NA

GCF_000694835 Scaffold Klebsiella pneumoniae UCI59 Klebsiella pneumoniae 1 Klebsiella pneumoniae 1 Klebsiella pneumoniae 1 NA

GCF_000694855 Scaffold Klebsiella pneumoniae UCI61 Klebsiella pneumoniae 1 Klebsiella pneumoniae 1 Klebsiella pneumoniae 1 NA

GCF_000694875 Scaffold Klebsiella pneumoniae UCI62 Klebsiella pneumoniae 1 Klebsiella pneumoniae 1 Klebsiella pneumoniae 1 NA

GCF_000694895 Scaffold Klebsiella pneumoniae UCI63 Klebsiella pneumoniae 1 Klebsiella pneumoniae 1 Klebsiella pneumoniae 1 NA

GCF_000694915 Scaffold Klebsiella pneumoniae UCI67 Klebsiella pneumoniae 1 Klebsiella pneumoniae 1 Klebsiella pneumoniae 1 NA

GCF_000694935 Scaffold Klebsiella pneumoniae UCI68 Klebsiella pneumoniae 1 Klebsiella pneumoniae 1 Klebsiella pneumoniae 1 NA

GCF_000695285 Contig Klebsiella pneumoniae pneumoniae KPB1 Klebsiella pneumoniae 1 Klebsiella pneumoniae 1 Klebsiella pneumoniae 1 NA

GCF_000695305 Contig Klebsiella pneumoniae pneumoniae KPB2 Klebsiella pneumoniae 1 Klebsiella pneumoniae 1 Klebsiella pneumoniae 1 NA

GCF_000695935 Complete Klebsiella pneumoniae pneumoniae KPNIH27 Klebsiella pneumoniae 1 Klebsiella pneumoniae 1 Klebsiella pneumoniae 1 NA

GCF_000699325 Contig Klebsiella pneumoniae 4541-2 Klebsiella pneumoniae 1 Klebsiella pneumoniae 1 Klebsiella pneumoniae 1 NA

GCF_000699345 Contig Klebsiella pneumoniae 11227-1 Klebsiella pneumoniae 1 Klebsiella pneumoniae 1 Klebsiella pneumoniae 1 NA

GCF_000699405 Contig Klebsiella pneumoniae 4006 Klebsiella pneumoniae 1 Klebsiella pneumoniae 1 Klebsiella pneumoniae 1 NA

GCF_000699425 Contig Klebsiella pneumoniae 7699 Klebsiella pneumoniae 1 Klebsiella pneumoniae 1 Klebsiella pneumoniae 1 NA

GCF_000706585 Scaffold Klebsiella pneumoniae UCI17 Klebsiella pneumoniae 1 Klebsiella pneumoniae 1 Klebsiella pneumoniae 1 NA

GCF_000706605 Scaffold Klebsiella pneumoniae BIDMC10 Klebsiella pneumoniae 1 Klebsiella pneumoniae 1 Klebsiella pneumoniae 1 NA

GCF_000707985 Scaffold Klebsiella pneumoniae BIDMC33B Klebsiella pneumoniae 1 Klebsiella pneumoniae 1 Klebsiella pneumoniae 1 NA

GCF_000710075 Contig Klebsiella pneumoniae 5422 Klebsiella pneumoniae 1 Klebsiella pneumoniae 1 Klebsiella pneumoniae 1 NA

GCF_000710095 Contig Klebsiella pneumoniae Top52 1721 Klebsiella pneumoniae 1 Klebsiella pneumoniae 1 Klebsiella pneumoniae 1 NA

GCF_000710805 Contig Klebsiella pneumoniae T2-1-2 Klebsiella pneumoniae 1 Klebsiella pneumoniae 1 Klebsiella pneumoniae 1 NA

GCF_000710855 Contig Klebsiella pneumoniae T2-1-1 Klebsiella pneumoniae 1 Klebsiella pneumoniae 1 Klebsiella pneumoniae 1 NA

GCF_000714655 Complete Klebsiella oxytoca KONIH1 Klebsiella michiganensis 1 Klebsiella michiganensis 1 Klebsiella michiganensis 1 NA

GCF_000714675 Complete Klebsiella pneumoniae pneumoniae KPNIH24 Klebsiella pneumoniae 1 Klebsiella pneumoniae 1 Klebsiella pneumoniae 1 NA

GCF_000717515 Complete Klebsiella pneumoniae pneumoniae KPR0928 Klebsiella pneumoniae 1 Klebsiella pneumoniae 1 Klebsiella pneumoniae 1 NA

GCF_000722485 Contig Klebsiella pneumoniae KP ST11 OXA48 Klebsiella pneumoniae 1 Klebsiella pneumoniae 1 Klebsiella pneumoniae 1 NA

GCF_000724525 Complete Klebsiella michiganensis M1 Klebsiella michiganensis 1 Klebsiella michiganensis 1 Klebsiella michiganensis 1 NA

GCF_000733255 Complete Klebsiella pneumoniae pneumoniae PittNDM01 Klebsiella pneumoniae 1 Klebsiella pneumoniae 1 Klebsiella pneumoniae 1 NA

GCF_000735215 Contig Klebsiella michiganensis R8A Klebsiella michiganensis 1 Klebsiella michiganensis 1 Klebsiella michiganensis 1 NA

GCF_000738865 Scaffold Klebsiella pneumoniae MRSN8157 Klebsiella pneumoniae 1 Klebsiella pneumoniae 1 Klebsiella pneumoniae 1 NA

GCF_000738875 Scaffold Klebsiella pneumoniae MRSN6920 Klebsiella pneumoniae 1 Klebsiella pneumoniae 1 Klebsiella pneumoniae 1 NA

GCF_000739495 Complete Klebsiella pneumoniae carbapenem-resistant blaNDM-1 Klebsiella pneumoniae 1 Klebsiella pneumoniae 1 Klebsiella pneumoniae 1 NA

GCF_000742135 Scaffold Klebsiella pneumoniae ATCC13883 Klebsiella pneumoniae 1 Klebsiella pneumoniae 1 Klebsiella pneumoniae 1 Type strain

GCF_000742755 Complete Klebsiella pneumoniae pneumoniae ATCC43816 KPPR1 Klebsiella pneumoniae 1 Klebsiella pneumoniae 1 Klebsiella pneumoniae 1 NA

GCF_000742915 Contig Klebsiella variicola BZ19 Klebsiella variicola Klebsiella variicola Klebsiella variicola NA

GCF_000747055 Contig Klebsiella pneumoniae 325 Klebsiella pneumoniae 1 Klebsiella pneumoniae 1 Klebsiella pneumoniae 1 NA

GCF_000751755 Contig Klebsiella quasipneumoniae quasipneumoniae 01A030 Klebsiella quasipneumoniae 2 Klebsiella quasipneumoniae 2 Klebsiella quasipneumoniae Type strain

GCF_000755545 Scaffold Klebsiella aerogenes CDC UA0804-01 Klebsiella aerogenes 1 Klebsiella aerogenes Klebsiella aerogenes NA

GCF_000755605 Scaffold Klebsiella pneumoniae KPPR1 Klebsiella pneumoniae 1 Klebsiella pneumoniae 1 Klebsiella pneumoniae 1 NA

GCF_000764615 Complete Klebsiella pneumoniae PMK1 Klebsiella pneumoniae 1 Klebsiella pneumoniae 1 Klebsiella pneumoniae 1 NA

GCF_000764635 Scaffold Klebsiella pneumoniae BAMC07-18 Klebsiella pneumoniae 1 Klebsiella pneumoniae 1 Klebsiella pneumoniae 1 NA

GCF_000766125 Contig Klebsiella pneumoniae 349 Klebsiella pneumoniae 1 Klebsiella pneumoniae 1 Klebsiella pneumoniae 1 NA

GCF_000773875 Contig Klebsiella pneumoniae MRSN1319 Klebsiella pneumoniae 1 Klebsiella pneumoniae 1 Klebsiella pneumoniae 1 NA

GCF_000775375 Complete Klebsiella pneumoniae pneumoniae KPNIH33 Klebsiella pneumoniae 1 Klebsiella pneumoniae 1 Klebsiella pneumoniae 1 NA

GCF_000775395 Complete Klebsiella pneumoniae pneumoniae KPNIH32 Klebsiella pneumoniae 1 Klebsiella pneumoniae 1 Klebsiella pneumoniae 1 NA

GCF_000775955 Complete Klebsiella pneumoniae XH209 Klebsiella pneumoniae 1 Klebsiella pneumoniae 1 Klebsiella pneumoniae 1 NA

GCF_000783515 Contig Klebsiella pneumoniae FDAARGOS84 Klebsiella pneumoniae 1 Klebsiella pneumoniae 1 Klebsiella pneumoniae 1 NA

GCF_000783895 Contig Klebsiella michiganensis FDAARGOS66 Klebsiella michiganensis 1 Klebsiella michiganensis 1 Klebsiella michiganensis 1 NA

GCF_000784945 Complete Klebsiella pneumoniae pneumoniae KPNIH29 Klebsiella pneumoniae 1 Klebsiella pneumoniae 1 Klebsiella pneumoniae 1 NA

GCF_000784985 Complete Klebsiella pneumoniae pneumoniae KPNIH30 Klebsiella pneumoniae 1 Klebsiella pneumoniae 1 Klebsiella pneumoniae 1 NA

GCF_000785005 Complete Klebsiella pneumoniae pneumoniae KPNIH31 Klebsiella pneumoniae 1 Klebsiella pneumoniae 1 Klebsiella pneumoniae 1 NA

GCF_000785625 Scaffold Klebsiella pneumoniae XDR Klebsiella pneumoniae 1 Klebsiella pneumoniae 1 Klebsiella pneumoniae 1 NA

GCF_000786375 Contig Klebsiella variicola 223/14 Klebsiella variicola Klebsiella variicola Klebsiella variicola NA

GCF_000787995 Scaffold Klebsiella pneumoniae 101488 Klebsiella pneumoniae 1 Klebsiella pneumoniae 1 Klebsiella pneumoniae 1 NA

GCF_000788005 Scaffold Klebsiella pneumoniae 101712 Klebsiella pneumoniae 1 Klebsiella pneumoniae 1 Klebsiella pneumoniae 1 NA

GCF_000788025 Scaffold Klebsiella pneumoniae 4640 Klebsiella pneumoniae 1 Klebsiella pneumoniae 1 Klebsiella pneumoniae 1 NA

GCF_000802765 Contig Klebsiella aerogenes ND17 Klebsiella aerogenes 1 Klebsiella aerogenes Klebsiella aerogenes NA

GCF_000805735 Contig Klebsiella pneumoniae CCBH13327 Klebsiella pneumoniae 1 Klebsiella pneumoniae 1 Klebsiella pneumoniae 1 NA

GCF_000807475 Contig Klebsiella pneumoniae 38544 Klebsiella pneumoniae 1 Klebsiella pneumoniae 1 Klebsiella pneumoniae 1 NA

GCF_000807495 Contig Klebsiella pneumoniae 38547 Klebsiella pneumoniae 1 Klebsiella pneumoniae 1 Klebsiella pneumoniae 1 NA

GCF_000807515 Contig Klebsiella pneumoniae 6234 Klebsiella pneumoniae 1 Klebsiella pneumoniae 1 Klebsiella pneumoniae 1 NA

GCF_000809125 Contig Klebsiella pneumoniae JT4 Klebsiella pneumoniae 1 Klebsiella pneumoniae 1 Klebsiella pneumoniae 1 NA

GCF_000812205 Complete Klebsiella variicola DX120E Klebsiella variicola Klebsiella variicola Klebsiella variicola NA

GCF_000812525 Contig Klebsiella pneumoniae GN8 Klebsiella pneumoniae 1 Klebsiella pneumoniae 1 Klebsiella pneumoniae 1 NA

GCF_000813205 Complete Klebsiella pneumoniae HK787 Klebsiella pneumoniae 1 Klebsiella pneumoniae 1 Klebsiella pneumoniae 1 NA

GCF_000814305 Complete Klebsiella pneumoniae 34618 Klebsiella pneumoniae 1 Klebsiella pneumoniae 1 Klebsiella pneumoniae 1 NA

GCF_000814805 Complete Klebsiella pneumoniae pneumoniae 1158 Klebsiella pneumoniae 1 Klebsiella pneumoniae 1 Klebsiella pneumoniae 1 NA

GCF_000817855 Scaffold Klebsiella pneumoniae ST147 Klebsiella pneumoniae 1 Klebsiella pneumoniae 1 Klebsiella pneumoniae 1 NA

GCF_000818615 Scaffold Klebsiella pneumoniae ST485 Klebsiella pneumoniae 1 Klebsiella pneumoniae 1 Klebsiella pneumoniae 1 NA

GCF_000826565 Contig Klebsiella variicola 801 Klebsiella variicola Klebsiella variicola Klebsiella variicola NA

GCF_000826605 Contig Klebsiella pneumoniae KPM nasey Klebsiella pneumoniae 1 Klebsiella pneumoniae 1 Klebsiella pneumoniae 1 NA

GCF_000826795 Scaffold Klebsiella pneumoniae UMNturkey9 Klebsiella pneumoniae 1 Klebsiella pneumoniae 1 Klebsiella pneumoniae 1 NA

GCF_000827665 Contig Klebsiella quasipneumoniae 1kgm Klebsiella quasipneumoniae 1 Klebsiella quasipneumoniae 1 Klebsiella quasipneumoniae NA

GCF_000828055 Complete Klebsiella variicola DSM15968 Klebsiella variicola Klebsiella variicola Klebsiella variicola Type strain

GCF_000941635 Complete Klebsiella variicola 8917 Klebsiella variicola Klebsiella variicola Klebsiella variicola NA

GCF_000942155 Contig Klebsiella variicola 1f2B Klebsiella variicola Klebsiella variicola Klebsiella variicola NA

GCF_000943095 Scaffold Klebsiella pneumoniae ST15 NDM1 Klebsiella pneumoniae 1 Klebsiella pneumoniae 1 Klebsiella pneumoniae 1 NA

GCF_000943135 Contig Klebsiella variicola 2b2A Klebsiella variicola Klebsiella variicola Klebsiella variicola NA

GCF_000943155 Scaffold Klebsiella pneumoniae novel ST outbreak lineage 2 Klebsiella pneumoniae 1 Klebsiella pneumoniae 1 Klebsiella pneumoniae 1 NA

GCF_000949515 Contig Klebsiella pneumoniae DSM2026 Klebsiella pneumoniae 1 Klebsiella pneumoniae 1 Klebsiella pneumoniae 1 NA

GCF_000956385 Contig Klebsiella pneumoniae CDPH3707 Klebsiella pneumoniae 1 Klebsiella pneumoniae 1 Klebsiella pneumoniae 1 NA

GCF_000956395 Contig Klebsiella pneumoniae CDPH3020 Klebsiella pneumoniae 1 Klebsiella pneumoniae 1 Klebsiella pneumoniae 1 NA

GCF_000956775 Contig Klebsiella aerogenes 33850 Klebsiella aerogenes 1 Klebsiella aerogenes Klebsiella aerogenes NA

GCF_000956895 Contig Klebsiella aerogenes 35007 Klebsiella aerogenes 2 Klebsiella aerogenes Klebsiella aerogenes NA

GCF_000957275 Contig Klebsiella aerogenes 42193 Klebsiella aerogenes 1 Klebsiella aerogenes Klebsiella aerogenes NA

GCF_000957485 Contig Klebsiella aerogenes 28944 Klebsiella aerogenes 1 Klebsiella aerogenes Klebsiella aerogenes NA

GCF_000957525 Contig Klebsiella aerogenes 32540 Klebsiella aerogenes 1 Klebsiella aerogenes Klebsiella aerogenes NA

GCF_000957705 Contig Klebsiella aerogenes 35003 Klebsiella aerogenes 1 Klebsiella aerogenes Klebsiella aerogenes NA

GCF_000957715 Contig Klebsiella aerogenes 35005 Klebsiella aerogenes 2 Klebsiella aerogenes Klebsiella aerogenes NA

GCF_000957725 Contig Klebsiella aerogenes 35006 Klebsiella aerogenes 2 Klebsiella aerogenes Klebsiella aerogenes NA

GCF_000958075 Contig Klebsiella aerogenes 35715 Klebsiella aerogenes 1 Klebsiella aerogenes Klebsiella aerogenes NA

GCF_000958305 Contig Klebsiella aerogenes 44247 Klebsiella aerogenes 1 Klebsiella aerogenes Klebsiella aerogenes NA

GCF_000958945 Scaffold Klebsiella pneumoniae CDPH3823 Klebsiella pneumoniae 1 Klebsiella pneumoniae 1 Klebsiella pneumoniae 1 NA

GCF_000958965 Scaffold Klebsiella pneumoniae CDPH5262 Klebsiella pneumoniae 1 Klebsiella pneumoniae 1 Klebsiella pneumoniae 1 NA

GCF_000963575 Complete Klebsiella michiganensis RC10 Klebsiella michiganensis 2 Klebsiella michiganensis 2 Klebsiella michiganensis 2 NA

GCF_000967845 Chromosome Klebsiella pneumoniae rhinoscleromatis SB3432 Klebsiella pneumoniae 1 Klebsiella pneumoniae 1 Klebsiella pneumoniae 1 NA

GCF_000968155 Complete Klebsiella pneumoniae Kp52145 Klebsiella pneumoniae 1 Klebsiella pneumoniae 1 Klebsiella pneumoniae 1 NA

GCF_000971075 Scaffold Klebsiella pneumoniae ST437 Klebsiella pneumoniae 1 Klebsiella pneumoniae 1 Klebsiella pneumoniae 1 NA

GCF_000981955 Contig Klebsiella pneumoniae MRSN2404 Klebsiella pneumoniae 1 Klebsiella pneumoniae 1 Klebsiella pneumoniae 1 NA

GCF_000982045 Contig Klebsiella pneumoniae MRSN6902 Klebsiella pneumoniae 1 Klebsiella pneumoniae 1 Klebsiella pneumoniae 1 NA

GCF_000982085 Contig Klebsiella pneumoniae HE12 Klebsiella pneumoniae 1 Klebsiella pneumoniae 1 Klebsiella pneumoniae 1 NA

GCF_000982205 Contig Klebsiella pneumoniae MRSN3852 Klebsiella pneumoniae 1 Klebsiella pneumoniae 1 Klebsiella pneumoniae 1 NA

GCF_000982215 Contig Klebsiella pneumoniae MRSN3562 Klebsiella pneumoniae 1 Klebsiella pneumoniae 1 Klebsiella pneumoniae 1 NA

GCF_000986855 Contig Klebsiella variicola CICC10011 Klebsiella variicola Klebsiella variicola Klebsiella variicola NA

GCF_001006265 Chromosome Klebsiella pneumoniae yzusk-4 Klebsiella pneumoniae 1 Klebsiella pneumoniae 1 Klebsiella pneumoniae 1 NA

GCF_001006545 Contig Klebsiella michiganensis CH2 Klebsiella michiganensis 1 Klebsiella michiganensis 1 Klebsiella michiganensis 1 NA

GCF_001006555 Contig Klebsiella aerogenes B3 Klebsiella aerogenes 1 Klebsiella aerogenes Klebsiella aerogenes NA

GCF_001006575 Contig Klebsiella variicola B1 Klebsiella variicola Klebsiella variicola Klebsiella variicola NA

GCF_001006625 Contig Klebsiella variicola CH4 Klebsiella variicola Klebsiella variicola Klebsiella variicola NA

GCF_001008725 Contig Klebsiella pneumoniae pneumoniae ST258 FL Klebsiella pneumoniae 1 Klebsiella pneumoniae 1 Klebsiella pneumoniae 1 NA

GCF_001011175 Contig Klebsiella aerogenes GN03019 Klebsiella aerogenes 1 Klebsiella aerogenes Klebsiella aerogenes NA

GCF_001011185 Contig Klebsiella aerogenes GN02173 Klebsiella aerogenes 1 Klebsiella aerogenes Klebsiella aerogenes NA

GCF_001011195 Contig Klebsiella aerogenes GN02286 Klebsiella aerogenes 1 Klebsiella aerogenes Klebsiella aerogenes NA

GCF_001011205 Contig Klebsiella aerogenes GN02326 Klebsiella aerogenes 1 Klebsiella aerogenes Klebsiella aerogenes NA

GCF_001011255 Contig Klebsiella aerogenes GN02278 Klebsiella aerogenes 1 Klebsiella aerogenes Klebsiella aerogenes NA

GCF_001011265 Contig Klebsiella aerogenes GN02355 Klebsiella aerogenes 1 Klebsiella aerogenes Klebsiella aerogenes NA

GCF_001011295 Contig Klebsiella aerogenes GN02384 Klebsiella aerogenes 1 Klebsiella aerogenes Klebsiella aerogenes NA

GCF_001011305 Contig Klebsiella aerogenes GN02420 Klebsiella aerogenes 1 Klebsiella aerogenes Klebsiella aerogenes NA

GCF_001011335 Contig Klebsiella aerogenes GN04690 Klebsiella aerogenes 1 Klebsiella aerogenes Klebsiella aerogenes NA

GCF_001011355 Contig Klebsiella aerogenes GN03543 Klebsiella aerogenes 1 Klebsiella aerogenes Klebsiella aerogenes NA

GCF_001011375 Contig Klebsiella aerogenes GN02154 Klebsiella aerogenes 1 Klebsiella aerogenes Klebsiella aerogenes NA

GCF_001011385 Contig Klebsiella aerogenes GN02079 Klebsiella aerogenes 1 Klebsiella aerogenes Klebsiella aerogenes NA

GCF_001011415 Contig Klebsiella aerogenes GN02126 Klebsiella aerogenes 1 Klebsiella aerogenes Klebsiella aerogenes NA

GCF_001011425 Contig Klebsiella aerogenes GN02464 Klebsiella aerogenes 1 Klebsiella aerogenes Klebsiella aerogenes NA

GCF_001011455 Contig Klebsiella aerogenes GN02329 Klebsiella aerogenes 1 Klebsiella aerogenes Klebsiella aerogenes NA

GCF_001011475 Contig Klebsiella aerogenes GN02499 Klebsiella aerogenes 1 Klebsiella aerogenes Klebsiella aerogenes NA

GCF_001011495 Contig Klebsiella aerogenes GN02509 Klebsiella aerogenes 2 Klebsiella aerogenes Klebsiella aerogenes NA

GCF_001011515 Contig Klebsiella aerogenes GN02694 Klebsiella aerogenes 2 Klebsiella aerogenes Klebsiella aerogenes NA

GCF_001011535 Contig Klebsiella aerogenes GN02525 Klebsiella aerogenes 1 Klebsiella aerogenes Klebsiella aerogenes NA

GCF_001011545 Contig Klebsiella aerogenes GN03688 Klebsiella aerogenes 1 Klebsiella aerogenes Klebsiella aerogenes NA

GCF_001011575 Contig Klebsiella aerogenes GN03927 Klebsiella aerogenes 1 Klebsiella aerogenes Klebsiella aerogenes NA

GCF_001011595 Contig Klebsiella aerogenes GN03959 Klebsiella aerogenes 1 Klebsiella aerogenes Klebsiella aerogenes NA

GCF_001011615 Contig Klebsiella aerogenes GN02770 Klebsiella aerogenes 1 Klebsiella aerogenes Klebsiella aerogenes NA

GCF_001011625 Contig Klebsiella aerogenes GN02761 Klebsiella aerogenes 2 Klebsiella aerogenes Klebsiella aerogenes NA

GCF_001011645 Contig Klebsiella aerogenes GN03732 Klebsiella aerogenes 1 Klebsiella aerogenes Klebsiella aerogenes NA

GCF_001011795 Contig Klebsiella aerogenes GN04835 Klebsiella aerogenes 2 Klebsiella aerogenes Klebsiella aerogenes NA

GCF_001011815 Contig Klebsiella aerogenes GN05253 Klebsiella aerogenes 1 Klebsiella aerogenes Klebsiella aerogenes NA

GCF_001011915 Contig Klebsiella aerogenes GN05224 Klebsiella aerogenes 1 Klebsiella aerogenes Klebsiella aerogenes NA

GCF_001011935 Contig Klebsiella aerogenes GN04794 Klebsiella aerogenes 1 Klebsiella aerogenes Klebsiella aerogenes NA

GCF_001021995 Complete Klebsiella aerogenes CAV1320 Klebsiella aerogenes 1 Klebsiella aerogenes Klebsiella aerogenes NA

GCF_001022035 Complete Klebsiella pneumoniae CAV1392 Klebsiella pneumoniae 1 Klebsiella pneumoniae 1 Klebsiella pneumoniae 1 NA

GCF_001022115 Complete Klebsiella oxytoca CAV1335 Klebsiella oxytoca 1 Klebsiella oxytoca 1 Klebsiella oxytoca 1 NA

GCF_001022175 Complete Klebsiella pneumoniae CAV1344 Klebsiella pneumoniae 1 Klebsiella pneumoniae 1 Klebsiella pneumoniae 1 NA

GCF_001022235 Complete Klebsiella pneumoniae CAV1596 Klebsiella pneumoniae 1 Klebsiella pneumoniae 1 Klebsiella pneumoniae 1 NA

GCF_001022295 Complete Klebsiella oxytoca CAV1099 Klebsiella oxytoca 1 Klebsiella oxytoca 1 Klebsiella oxytoca 1 NA

GCF_001028875 Contig Klebsiella michiganensis PHS892 Klebsiella michiganensis 1 Klebsiella michiganensis 1 Klebsiella michiganensis 1 NA

GCF_001028885 Contig Klebsiella michiganensis PHS890 Klebsiella michiganensis 1 Klebsiella michiganensis 1 Klebsiella michiganensis 1 NA

GCF_001030055 Contig Klebsiella aerogenes UCI98 Klebsiella aerogenes 1 Klebsiella aerogenes Klebsiella aerogenes NA

GCF_001030125 Scaffold Klebsiella aerogenes UCI89 Klebsiella aerogenes 1 Klebsiella aerogenes Klebsiella aerogenes NA

GCF_001030165 Scaffold Klebsiella aerogenes UCI90 Klebsiella aerogenes 1 Klebsiella aerogenes Klebsiella aerogenes NA

GCF_001030185 Scaffold Klebsiella aerogenes UCI97 Klebsiella aerogenes 1 Klebsiella aerogenes Klebsiella aerogenes NA

GCF_001030705 Scaffold Klebsiella oxytoca CHS143 Klebsiella oxytoca 1 Klebsiella oxytoca 1 Klebsiella oxytoca 1 NA

GCF_001030715 Scaffold Klebsiella pneumoniae CHS153 Klebsiella pneumoniae 1 Klebsiella pneumoniae 1 Klebsiella pneumoniae 1 NA

GCF_001030745 Scaffold Klebsiella pneumoniae CHS222 Klebsiella pneumoniae 1 Klebsiella pneumoniae 1 Klebsiella pneumoniae 1 NA

GCF_001030755 Scaffold Klebsiella michiganensis MGH87 Klebsiella michiganensis 1 Klebsiella michiganensis 1 Klebsiella michiganensis 1 NA

GCF_001030775 Scaffold Klebsiella oxytoca MGH88 Klebsiella oxytoca 1 Klebsiella oxytoca 1 Klebsiella oxytoca 1 NA

GCF_001030795 Scaffold Klebsiella pneumoniae CHS81 Klebsiella pneumoniae 1 Klebsiella pneumoniae 1 Klebsiella pneumoniae 1 NA

GCF_001030825 Scaffold Klebsiella pneumoniae CHS82 Klebsiella pneumoniae 1 Klebsiella pneumoniae 1 Klebsiella pneumoniae 1 NA

GCF_001030835 Scaffold Klebsiella pneumoniae CHS83 Klebsiella pneumoniae 1 Klebsiella pneumoniae 1 Klebsiella pneumoniae 1 NA

GCF_001030845 Scaffold Klebsiella pneumoniae CHS84 Klebsiella pneumoniae 1 Klebsiella pneumoniae 1 Klebsiella pneumoniae 1 NA

GCF_001030875 Scaffold Klebsiella pneumoniae CHS85 Klebsiella pneumoniae 1 Klebsiella pneumoniae 1 Klebsiella pneumoniae 1 NA

GCF_001030905 Scaffold Klebsiella pneumoniae CHS88 Klebsiella pneumoniae 1 Klebsiella pneumoniae 1 Klebsiella pneumoniae 1 NA

GCF_001030915 Scaffold Klebsiella pneumoniae CHS89 Klebsiella pneumoniae 1 Klebsiella pneumoniae 1 Klebsiella pneumoniae 1 NA

GCF_001030935 Scaffold Klebsiella pneumoniae CHS90 Klebsiella pneumoniae 1 Klebsiella pneumoniae 1 Klebsiella pneumoniae 1 NA

GCF_001030945 Scaffold Klebsiella pneumoniae CHS91 Klebsiella pneumoniae 1 Klebsiella pneumoniae 1 Klebsiella pneumoniae 1 NA

GCF_001030985 Scaffold Klebsiella pneumoniae CHS92 Klebsiella pneumoniae 1 Klebsiella pneumoniae 1 Klebsiella pneumoniae 1 NA

GCF_001030995 Scaffold Klebsiella pneumoniae CHS93 Klebsiella pneumoniae 1 Klebsiella pneumoniae 1 Klebsiella pneumoniae 1 NA

GCF_001031015 Scaffold Klebsiella pneumoniae CHS94 Klebsiella pneumoniae 1 Klebsiella pneumoniae 1 Klebsiella pneumoniae 1 NA

GCF_001031025 Scaffold Klebsiella pneumoniae CHS95 Klebsiella pneumoniae 1 Klebsiella pneumoniae 1 Klebsiella pneumoniae 1 NA

GCF_001031065 Scaffold Klebsiella pneumoniae CHS96 Klebsiella pneumoniae 1 Klebsiella pneumoniae 1 Klebsiella pneumoniae 1 NA

GCF_001031075 Scaffold Klebsiella pneumoniae CHS97 Klebsiella pneumoniae 1 Klebsiella pneumoniae 1 Klebsiella pneumoniae 1 NA

GCF_001031095 Scaffold Klebsiella pneumoniae CHS98 Klebsiella pneumoniae 1 Klebsiella pneumoniae 1 Klebsiella pneumoniae 1 NA

GCF_001031105 Scaffold Klebsiella pneumoniae CHS99 Klebsiella pneumoniae 1 Klebsiella pneumoniae 1 Klebsiella pneumoniae 1 NA

GCF_001031145 Scaffold Klebsiella pneumoniae CHS100 Klebsiella pneumoniae 1 Klebsiella pneumoniae 1 Klebsiella pneumoniae 1 NA

GCF_001031155 Scaffold Klebsiella pneumoniae CHS101 Klebsiella pneumoniae 1 Klebsiella pneumoniae 1 Klebsiella pneumoniae 1 NA

GCF_001031175 Scaffold Klebsiella pneumoniae CHS102 Klebsiella pneumoniae 1 Klebsiella pneumoniae 1 Klebsiella pneumoniae 1 NA

GCF_001031185 Scaffold Klebsiella pneumoniae CHS104 Klebsiella pneumoniae 1 Klebsiella pneumoniae 1 Klebsiella pneumoniae 1 NA

GCF_001031225 Scaffold Klebsiella pneumoniae CHS105 Klebsiella pneumoniae 1 Klebsiella pneumoniae 1 Klebsiella pneumoniae 1 NA

GCF_001031235 Scaffold Klebsiella pneumoniae CHS106 Klebsiella pneumoniae 1 Klebsiella pneumoniae 1 Klebsiella pneumoniae 1 NA

GCF_001031245 Scaffold Klebsiella pneumoniae CHS107 Klebsiella pneumoniae 1 Klebsiella pneumoniae 1 Klebsiella pneumoniae 1 NA

GCF_001031265 Scaffold Klebsiella pneumoniae CHS109 Klebsiella pneumoniae 1 Klebsiella pneumoniae 1 Klebsiella pneumoniae 1 NA

GCF_001031305 Scaffold Klebsiella pneumoniae CHS110 Klebsiella pneumoniae 1 Klebsiella pneumoniae 1 Klebsiella pneumoniae 1 NA

GCF_001031315 Scaffold Klebsiella pneumoniae CHS111 Klebsiella pneumoniae 1 Klebsiella pneumoniae 1 Klebsiella pneumoniae 1 NA

GCF_001031325 Scaffold Klebsiella pneumoniae CHS112 Klebsiella pneumoniae 1 Klebsiella pneumoniae 1 Klebsiella pneumoniae 1 NA

GCF_001031345 Scaffold Klebsiella pneumoniae CHS113 Klebsiella pneumoniae 1 Klebsiella pneumoniae 1 Klebsiella pneumoniae 1 NA

GCF_001031385 Scaffold Klebsiella pneumoniae CHS114 Klebsiella pneumoniae 1 Klebsiella pneumoniae 1 Klebsiella pneumoniae 1 NA

GCF_001031395 Scaffold Klebsiella pneumoniae CHS115 Klebsiella pneumoniae 1 Klebsiella pneumoniae 1 Klebsiella pneumoniae 1 NA

GCF_001031405 Scaffold Klebsiella pneumoniae CHS116 Klebsiella pneumoniae 1 Klebsiella pneumoniae 1 Klebsiella pneumoniae 1 NA

GCF_001031415 Scaffold Klebsiella pneumoniae CHS117 Klebsiella pneumoniae 1 Klebsiella pneumoniae 1 Klebsiella pneumoniae 1 NA

GCF_001031465 Scaffold Klebsiella pneumoniae CHS118 Klebsiella pneumoniae 1 Klebsiella pneumoniae 1 Klebsiella pneumoniae 1 NA

GCF_001031475 Scaffold Klebsiella pneumoniae CHS121 Klebsiella pneumoniae 1 Klebsiella pneumoniae 1 Klebsiella pneumoniae 1 NA

GCF_001031485 Scaffold Klebsiella pneumoniae CHS122 Klebsiella pneumoniae 1 Klebsiella pneumoniae 1 Klebsiella pneumoniae 1 NA

GCF_001031515 Scaffold Klebsiella pneumoniae CHS123 Klebsiella pneumoniae 1 Klebsiella pneumoniae 1 Klebsiella pneumoniae 1 NA

GCF_001031545 Scaffold Klebsiella pneumoniae CHS126 Klebsiella pneumoniae 1 Klebsiella pneumoniae 1 Klebsiella pneumoniae 1 NA

GCF_001031555 Scaffold Klebsiella pneumoniae CHS125 Klebsiella pneumoniae 1 Klebsiella pneumoniae 1 Klebsiella pneumoniae 1 NA

GCF_001031565 Scaffold Klebsiella pneumoniae CHS127 Klebsiella pneumoniae 1 Klebsiella pneumoniae 1 Klebsiella pneumoniae 1 NA

GCF_001031595 Scaffold Klebsiella pneumoniae CHS128 Klebsiella pneumoniae 1 Klebsiella pneumoniae 1 Klebsiella pneumoniae 1 NA

GCF_001031625 Scaffold Klebsiella pneumoniae CHS129 Klebsiella pneumoniae 1 Klebsiella pneumoniae 1 Klebsiella pneumoniae 1 NA

GCF_001031635 Scaffold Klebsiella pneumoniae CHS131 Klebsiella pneumoniae 1 Klebsiella pneumoniae 1 Klebsiella pneumoniae 1 NA

GCF_001031645 Scaffold Klebsiella pneumoniae CHS132 Klebsiella pneumoniae 1 Klebsiella pneumoniae 1 Klebsiella pneumoniae 1 NA

GCF_001031675 Scaffold Klebsiella pneumoniae CHS133 Klebsiella pneumoniae 1 Klebsiella pneumoniae 1 Klebsiella pneumoniae 1 NA

GCF_001031705 Scaffold Klebsiella pneumoniae CHS134 Klebsiella pneumoniae 1 Klebsiella pneumoniae 1 Klebsiella pneumoniae 1 NA

GCF_001031715 Scaffold Klebsiella pneumoniae CHS135 Klebsiella pneumoniae 1 Klebsiella pneumoniae 1 Klebsiella pneumoniae 1 NA

GCF_001031725 Scaffold Klebsiella pneumoniae CHS137 Klebsiella pneumoniae 1 Klebsiella pneumoniae 1 Klebsiella pneumoniae 1 NA

GCF_001031745 Scaffold Klebsiella pneumoniae CHS138 Klebsiella pneumoniae 1 Klebsiella pneumoniae 1 Klebsiella pneumoniae 1 NA

GCF_001031785 Scaffold Klebsiella pneumoniae CHS139 Klebsiella pneumoniae 1 Klebsiella pneumoniae 1 Klebsiella pneumoniae 1 NA

GCF_001031795 Scaffold Klebsiella pneumoniae CHS140 Klebsiella pneumoniae 1 Klebsiella pneumoniae 1 Klebsiella pneumoniae 1 NA

GCF_001031805 Scaffold Klebsiella pneumoniae CHS141 Klebsiella pneumoniae 1 Klebsiella pneumoniae 1 Klebsiella pneumoniae 1 NA

GCF_001031825 Scaffold Klebsiella pneumoniae CHS142 Klebsiella pneumoniae 1 Klebsiella pneumoniae 1 Klebsiella pneumoniae 1 NA

GCF_001031865 Scaffold Klebsiella pneumoniae CHS144 Klebsiella pneumoniae 1 Klebsiella pneumoniae 1 Klebsiella pneumoniae 1 NA

GCF_001031875 Scaffold Klebsiella pneumoniae CHS145 Klebsiella pneumoniae 1 Klebsiella pneumoniae 1 Klebsiella pneumoniae 1 NA

GCF_001031895 Scaffold Klebsiella pneumoniae CHS146 Klebsiella pneumoniae 1 Klebsiella pneumoniae 1 Klebsiella pneumoniae 1 NA

GCF_001031905 Scaffold Klebsiella pneumoniae CHS147 Klebsiella pneumoniae 1 Klebsiella pneumoniae 1 Klebsiella pneumoniae 1 NA

GCF_001031945 Scaffold Klebsiella pneumoniae CHS148 Klebsiella pneumoniae 1 Klebsiella pneumoniae 1 Klebsiella pneumoniae 1 NA

GCF_001031955 Scaffold Klebsiella pneumoniae CHS150 Klebsiella pneumoniae 1 Klebsiella pneumoniae 1 Klebsiella pneumoniae 1 NA

GCF_001031965 Scaffold Klebsiella pneumoniae CHS149 Klebsiella pneumoniae 1 Klebsiella pneumoniae 1 Klebsiella pneumoniae 1 NA

GCF_001031995 Scaffold Klebsiella pneumoniae CHS151 Klebsiella pneumoniae 1 Klebsiella pneumoniae 1 Klebsiella pneumoniae 1 NA

GCF_001032025 Scaffold Klebsiella pneumoniae CHS152 Klebsiella pneumoniae 1 Klebsiella pneumoniae 1 Klebsiella pneumoniae 1 NA

GCF_001032035 Scaffold Klebsiella pneumoniae CHS154 Klebsiella pneumoniae 1 Klebsiella pneumoniae 1 Klebsiella pneumoniae 1 NA

GCF_001032045 Scaffold Klebsiella pneumoniae CHS155 Klebsiella pneumoniae 1 Klebsiella pneumoniae 1 Klebsiella pneumoniae 1 NA

GCF_001032075 Scaffold Klebsiella pneumoniae CHS156 Klebsiella pneumoniae 1 Klebsiella pneumoniae 1 Klebsiella pneumoniae 1 NA

GCF_001032105 Scaffold Klebsiella pneumoniae CHS157 Klebsiella pneumoniae 1 Klebsiella pneumoniae 1 Klebsiella pneumoniae 1 NA

GCF_001032115 Scaffold Klebsiella pneumoniae CHS158 Klebsiella pneumoniae 1 Klebsiella pneumoniae 1 Klebsiella pneumoniae 1 NA

GCF_001032125 Scaffold Klebsiella pneumoniae CHS159 Klebsiella pneumoniae 1 Klebsiella pneumoniae 1 Klebsiella pneumoniae 1 NA

GCF_001032145 Scaffold Klebsiella pneumoniae CHS160 Klebsiella pneumoniae 1 Klebsiella pneumoniae 1 Klebsiella pneumoniae 1 NA

GCF_001032185 Scaffold Klebsiella pneumoniae CHS161 Klebsiella pneumoniae 1 Klebsiella pneumoniae 1 Klebsiella pneumoniae 1 NA

GCF_001032195 Scaffold Klebsiella pneumoniae CHS163 Klebsiella pneumoniae 1 Klebsiella pneumoniae 1 Klebsiella pneumoniae 1 NA

GCF_001032205 Scaffold Klebsiella pneumoniae CHS162 Klebsiella pneumoniae 1 Klebsiella pneumoniae 1 Klebsiella pneumoniae 1 NA

GCF_001032225 Scaffold Klebsiella pneumoniae CHS164 Klebsiella pneumoniae 1 Klebsiella pneumoniae 1 Klebsiella pneumoniae 1 NA

GCF_001032265 Scaffold Klebsiella pneumoniae CHS165 Klebsiella pneumoniae 1 Klebsiella pneumoniae 1 Klebsiella pneumoniae 1 NA

GCF_001032275 Scaffold Klebsiella pneumoniae CHS166 Klebsiella pneumoniae 1 Klebsiella pneumoniae 1 Klebsiella pneumoniae 1 NA

GCF_001032285 Scaffold Klebsiella pneumoniae CHS167 Klebsiella pneumoniae 1 Klebsiella pneumoniae 1 Klebsiella pneumoniae 1 NA

GCF_001032305 Scaffold Klebsiella pneumoniae CHS168 Klebsiella pneumoniae 1 Klebsiella pneumoniae 1 Klebsiella pneumoniae 1 NA

GCF_001032345 Scaffold Klebsiella pneumoniae CHS169 Klebsiella pneumoniae 1 Klebsiella pneumoniae 1 Klebsiella pneumoniae 1 NA

GCF_001032355 Scaffold Klebsiella pneumoniae CHS170 Klebsiella pneumoniae 1 Klebsiella pneumoniae 1 Klebsiella pneumoniae 1 NA

GCF_001032365 Scaffold Klebsiella pneumoniae CHS172 Klebsiella pneumoniae 1 Klebsiella pneumoniae 1 Klebsiella pneumoniae 1 NA

GCF_001032375 Scaffold Klebsiella pneumoniae CHS173 Klebsiella pneumoniae 1 Klebsiella pneumoniae 1 Klebsiella pneumoniae 1 NA

GCF_001032425 Scaffold Klebsiella pneumoniae CHS174 Klebsiella pneumoniae 1 Klebsiella pneumoniae 1 Klebsiella pneumoniae 1 NA

GCF_001032435 Scaffold Klebsiella pneumoniae CHS176 Klebsiella pneumoniae 1 Klebsiella pneumoniae 1 Klebsiella pneumoniae 1 NA

GCF_001032445 Scaffold Klebsiella pneumoniae CHS177 Klebsiella pneumoniae 1 Klebsiella pneumoniae 1 Klebsiella pneumoniae 1 NA

GCF_001032455 Scaffold Klebsiella pneumoniae CHS179 Klebsiella pneumoniae 1 Klebsiella pneumoniae 1 Klebsiella pneumoniae 1 NA

GCF_001032505 Scaffold Klebsiella pneumoniae CHS181 Klebsiella pneumoniae 1 Klebsiella pneumoniae 1 Klebsiella pneumoniae 1 NA

GCF_001032525 Scaffold Klebsiella pneumoniae CHS182 Klebsiella pneumoniae 1 Klebsiella pneumoniae 1 Klebsiella pneumoniae 1 NA

GCF_001032535 Scaffold Klebsiella pneumoniae CHS183 Klebsiella pneumoniae 1 Klebsiella pneumoniae 1 Klebsiella pneumoniae 1 NA

GCF_001032545 Scaffold Klebsiella pneumoniae CHS184 Klebsiella pneumoniae 1 Klebsiella pneumoniae 1 Klebsiella pneumoniae 1 NA

GCF_001032585 Scaffold Klebsiella pneumoniae CHS185 Klebsiella pneumoniae 1 Klebsiella pneumoniae 1 Klebsiella pneumoniae 1 NA

GCF_001032605 Scaffold Klebsiella pneumoniae CHS186 Klebsiella pneumoniae 1 Klebsiella pneumoniae 1 Klebsiella pneumoniae 1 NA

GCF_001032615 Scaffold Klebsiella pneumoniae CHS189 Klebsiella pneumoniae 1 Klebsiella pneumoniae 1 Klebsiella pneumoniae 1 NA

GCF_001032625 Scaffold Klebsiella pneumoniae CHS191 Klebsiella pneumoniae 1 Klebsiella pneumoniae 1 Klebsiella pneumoniae 1 NA

GCF_001032665 Scaffold Klebsiella pneumoniae CHS192 Klebsiella pneumoniae 1 Klebsiella pneumoniae 1 Klebsiella pneumoniae 1 NA

GCF_001032685 Scaffold Klebsiella pneumoniae CHS194 Klebsiella pneumoniae 1 Klebsiella pneumoniae 1 Klebsiella pneumoniae 1 NA

GCF_001032695 Scaffold Klebsiella pneumoniae CHS195 Klebsiella pneumoniae 1 Klebsiella pneumoniae 1 Klebsiella pneumoniae 1 NA

GCF_001032705 Scaffold Klebsiella pneumoniae CHS197 Klebsiella pneumoniae 1 Klebsiella pneumoniae 1 Klebsiella pneumoniae 1 NA

GCF_001032765 Scaffold Klebsiella pneumoniae CHS200 Klebsiella pneumoniae 1 Klebsiella pneumoniae 1 Klebsiella pneumoniae 1 NA

GCF_001032775 Scaffold Klebsiella pneumoniae CHS201 Klebsiella pneumoniae 1 Klebsiella pneumoniae 1 Klebsiella pneumoniae 1 NA

GCF_001032795 Scaffold Klebsiella pneumoniae CHS202 Klebsiella pneumoniae 1 Klebsiella pneumoniae 1 Klebsiella pneumoniae 1 NA

GCF_001032815 Scaffold Klebsiella pneumoniae CHS205 Klebsiella pneumoniae 1 Klebsiella pneumoniae 1 Klebsiella pneumoniae 1 NA

GCF_001032845 Scaffold Klebsiella pneumoniae CHS206 Klebsiella pneumoniae 1 Klebsiella pneumoniae 1 Klebsiella pneumoniae 1 NA

GCF_001032855 Scaffold Klebsiella pneumoniae CHS207 Klebsiella pneumoniae 1 Klebsiella pneumoniae 1 Klebsiella pneumoniae 1 NA

GCF_001032875 Scaffold Klebsiella pneumoniae CHS208 Klebsiella pneumoniae 1 Klebsiella pneumoniae 1 Klebsiella pneumoniae 1 NA

GCF_001032895 Scaffold Klebsiella pneumoniae CHS209 Klebsiella pneumoniae 1 Klebsiella pneumoniae 1 Klebsiella pneumoniae 1 NA

GCF_001032925 Scaffold Klebsiella pneumoniae CHS210 Klebsiella pneumoniae 1 Klebsiella pneumoniae 1 Klebsiella pneumoniae 1 NA

GCF_001032935 Scaffold Klebsiella pneumoniae CHS211 Klebsiella pneumoniae 1 Klebsiella pneumoniae 1 Klebsiella pneumoniae 1 NA

GCF_001032965 Scaffold Klebsiella pneumoniae CHS213 Klebsiella pneumoniae 1 Klebsiella pneumoniae 1 Klebsiella pneumoniae 1 NA

GCF_001032975 Scaffold Klebsiella pneumoniae CHS214 Klebsiella pneumoniae 1 Klebsiella pneumoniae 1 Klebsiella pneumoniae 1 NA

GCF_001033005 Scaffold Klebsiella pneumoniae CHS215 Klebsiella pneumoniae 1 Klebsiella pneumoniae 1 Klebsiella pneumoniae 1 NA

GCF_001033015 Scaffold Klebsiella pneumoniae CHS216 Klebsiella pneumoniae 1 Klebsiella pneumoniae 1 Klebsiella pneumoniae 1 NA

GCF_001033045 Scaffold Klebsiella pneumoniae CHS217 Klebsiella pneumoniae 1 Klebsiella pneumoniae 1 Klebsiella pneumoniae 1 NA

GCF_001033055 Scaffold Klebsiella pneumoniae CHS218 Klebsiella pneumoniae 1 Klebsiella pneumoniae 1 Klebsiella pneumoniae 1 NA

GCF_001033085 Scaffold Klebsiella pneumoniae CHS219 Klebsiella pneumoniae 1 Klebsiella pneumoniae 1 Klebsiella pneumoniae 1 NA

GCF_001033095 Scaffold Klebsiella pneumoniae CHS220 Klebsiella pneumoniae 1 Klebsiella pneumoniae 1 Klebsiella pneumoniae 1 NA

GCF_001033125 Scaffold Klebsiella pneumoniae CHS221 Klebsiella pneumoniae 1 Klebsiella pneumoniae 1 Klebsiella pneumoniae 1 NA

GCF_001033135 Scaffold Klebsiella pneumoniae CHS223 Klebsiella pneumoniae 1 Klebsiella pneumoniae 1 Klebsiella pneumoniae 1 NA

GCF_001033165 Scaffold Klebsiella pneumoniae CHS224 Klebsiella pneumoniae 1 Klebsiella pneumoniae 1 Klebsiella pneumoniae 1 NA

GCF_001033175 Scaffold Klebsiella pneumoniae CHS225 Klebsiella pneumoniae 1 Klebsiella pneumoniae 1 Klebsiella pneumoniae 1 NA

GCF_001033205 Scaffold Klebsiella pneumoniae CHS226 Klebsiella pneumoniae 1 Klebsiella pneumoniae 1 Klebsiella pneumoniae 1 NA

GCF_001033215 Scaffold Klebsiella pneumoniae CHS228 Klebsiella pneumoniae 1 Klebsiella pneumoniae 1 Klebsiella pneumoniae 1 NA

GCF_001033245 Scaffold Klebsiella pneumoniae CHS229 Klebsiella pneumoniae 1 Klebsiella pneumoniae 1 Klebsiella pneumoniae 1 NA

GCF_001033255 Scaffold Klebsiella pneumoniae CHS230 Klebsiella pneumoniae 1 Klebsiella pneumoniae 1 Klebsiella pneumoniae 1 NA

GCF_001033285 Scaffold Klebsiella pneumoniae CHS231 Klebsiella pneumoniae 1 Klebsiella pneumoniae 1 Klebsiella pneumoniae 1 NA

GCF_001033295 Scaffold Klebsiella pneumoniae CHS232 Klebsiella pneumoniae 1 Klebsiella pneumoniae 1 Klebsiella pneumoniae 1 NA

GCF_001033305 Scaffold Klebsiella pneumoniae CHS234 Klebsiella pneumoniae 1 Klebsiella pneumoniae 1 Klebsiella pneumoniae 1 NA

GCF_001033335 Scaffold Klebsiella pneumoniae CHS235 Klebsiella pneumoniae 1 Klebsiella pneumoniae 1 Klebsiella pneumoniae 1 NA

GCF_001033365 Scaffold Klebsiella pneumoniae CHS236 Klebsiella pneumoniae 1 Klebsiella pneumoniae 1 Klebsiella pneumoniae 1 NA

GCF_001033375 Scaffold Klebsiella pneumoniae CHS237 Klebsiella pneumoniae 1 Klebsiella pneumoniae 1 Klebsiella pneumoniae 1 NA

GCF_001033385 Scaffold Klebsiella pneumoniae BWH53 Klebsiella pneumoniae 1 Klebsiella pneumoniae 1 Klebsiella pneumoniae 1 NA

GCF_001033415 Scaffold Klebsiella pneumoniae BWH58 Klebsiella pneumoniae 1 Klebsiella pneumoniae 1 Klebsiella pneumoniae 1 NA

GCF_001033445 Scaffold Klebsiella pneumoniae BWH62 Klebsiella pneumoniae 1 Klebsiella pneumoniae 1 Klebsiella pneumoniae 1 NA

GCF_001033455 Scaffold Klebsiella pneumoniae MGH81 Klebsiella pneumoniae 1 Klebsiella pneumoniae 1 Klebsiella pneumoniae 1 NA

GCF_001033465 Scaffold Klebsiella pneumoniae MGH82 Klebsiella pneumoniae 1 Klebsiella pneumoniae 1 Klebsiella pneumoniae 1 NA

GCF_001033505 Scaffold Klebsiella pneumoniae MGH84 Klebsiella pneumoniae 1 Klebsiella pneumoniae 1 Klebsiella pneumoniae 1 NA

GCF_001033525 Scaffold Klebsiella pneumoniae MGH89 Klebsiella pneumoniae 1 Klebsiella pneumoniae 1 Klebsiella pneumoniae 1 NA

GCF_001033535 Scaffold Klebsiella pneumoniae MGH90 Klebsiella pneumoniae 1 Klebsiella pneumoniae 1 Klebsiella pneumoniae 1 NA

GCF_001033565 Scaffold Klebsiella pneumoniae MGH91 Klebsiella pneumoniae 1 Klebsiella pneumoniae 1 Klebsiella pneumoniae 1 NA

GCF_001033575 Scaffold Klebsiella variicola MGH92 Klebsiella variicola Klebsiella variicola Klebsiella variicola NA

GCF_001033585 Scaffold Klebsiella pneumoniae MGH93 Klebsiella pneumoniae 1 Klebsiella pneumoniae 1 Klebsiella pneumoniae 1 NA

GCF_001033615 Scaffold Klebsiella pneumoniae MGH94 Klebsiella pneumoniae 1 Klebsiella pneumoniae 1 Klebsiella pneumoniae 1 NA

GCF_001033645 Scaffold Klebsiella pneumoniae MGH95 Klebsiella pneumoniae 1 Klebsiella pneumoniae 1 Klebsiella pneumoniae 1 NA

GCF_001033665 Scaffold Klebsiella quasipneumoniae quasipneumoniae MGH96 Klebsiella quasipneumoniae 2 Klebsiella quasipneumoniae 2 Klebsiella quasipneumoniae NA

GCF_001033675 Scaffold Klebsiella pneumoniae MGH97 Klebsiella pneumoniae 1 Klebsiella pneumoniae 1 Klebsiella pneumoniae 1 NA

GCF_001033685 Scaffold Klebsiella pneumoniae MGH98 Klebsiella pneumoniae 1 Klebsiella pneumoniae 1 Klebsiella pneumoniae 1 NA

GCF_001033725 Scaffold Klebsiella pneumoniae MGH101 Klebsiella pneumoniae 1 Klebsiella pneumoniae 1 Klebsiella pneumoniae 1 NA

GCF_001033745 Scaffold Klebsiella pneumoniae MGH102 Klebsiella pneumoniae 1 Klebsiella pneumoniae 1 Klebsiella pneumoniae 1 NA

GCF_001033755 Scaffold Klebsiella pneumoniae MGH111 Klebsiella pneumoniae 1 Klebsiella pneumoniae 1 Klebsiella pneumoniae 1 NA

GCF_001033765 Scaffold Klebsiella pneumoniae MGH112 Klebsiella pneumoniae 1 Klebsiella pneumoniae 1 Klebsiella pneumoniae 1 NA

GCF_001033805 Scaffold Klebsiella quasipneumoniae MGH113 Klebsiella quasipneumoniae 2 Klebsiella quasipneumoniae 2 Klebsiella quasipneumoniae NA

GCF_001033825 Scaffold Klebsiella variicola MGH114 Klebsiella variicola Klebsiella variicola Klebsiella variicola NA

GCF_001033835 Scaffold Klebsiella pneumoniae MGH115 Klebsiella pneumoniae 1 Klebsiella pneumoniae 1 Klebsiella pneumoniae 1 NA

GCF_001033845 Scaffold Klebsiella pneumoniae MGH116 Klebsiella pneumoniae 1 Klebsiella pneumoniae 1 Klebsiella pneumoniae 1 NA

GCF_001033885 Scaffold Klebsiella pneumoniae MGH117 Klebsiella pneumoniae 1 Klebsiella pneumoniae 1 Klebsiella pneumoniae 1 NA

GCF_001033895 Scaffold Klebsiella pneumoniae MGH118 Klebsiella pneumoniae 1 Klebsiella pneumoniae 1 Klebsiella pneumoniae 1 NA

GCF_001033915 Scaffold Klebsiella quasipneumoniae MGH123 Klebsiella quasipneumoniae 1 Klebsiella quasipneumoniae 1 Klebsiella quasipneumoniae NA

GCF_001033925 Scaffold Klebsiella pneumoniae MGH124 Klebsiella pneumoniae 1 Klebsiella pneumoniae 1 Klebsiella pneumoniae 1 NA

GCF_001033965 Scaffold Klebsiella pneumoniae MGH125 Klebsiella pneumoniae 1 Klebsiella pneumoniae 1 Klebsiella pneumoniae 1 NA

GCF_001033975 Scaffold Klebsiella pneumoniae MGH126 Klebsiella pneumoniae 1 Klebsiella pneumoniae 1 Klebsiella pneumoniae 1 NA

GCF_001033995 Scaffold Klebsiella pneumoniae BIDMC85 Klebsiella pneumoniae 1 Klebsiella pneumoniae 1 Klebsiella pneumoniae 1 NA

GCF_001034005 Scaffold Klebsiella pneumoniae BIDMC86 Klebsiella pneumoniae 1 Klebsiella pneumoniae 1 Klebsiella pneumoniae 1 NA

GCF_001034045 Scaffold Klebsiella variicola BIDMC88 Klebsiella variicola Klebsiella variicola Klebsiella variicola NA

GCF_001034055 Scaffold Klebsiella pneumoniae BIDMC89 Klebsiella pneumoniae 1 Klebsiella pneumoniae 1 Klebsiella pneumoniae 1 NA

GCF_001034085 Scaffold Klebsiella pneumoniae BIDMC91 Klebsiella pneumoniae 1 Klebsiella pneumoniae 1 Klebsiella pneumoniae 1 NA

GCF_001034125 Scaffold Klebsiella quasipneumoniae BIDMC95 Klebsiella quasipneumoniae 1 Klebsiella quasipneumoniae 1 Klebsiella quasipneumoniae NA

GCF_001034135 Scaffold Klebsiella pneumoniae BIDMC96 Klebsiella pneumoniae 1 Klebsiella pneumoniae 1 Klebsiella pneumoniae 1 NA

GCF_001034155 Scaffold Klebsiella pneumoniae UCI70 Klebsiella pneumoniae 1 Klebsiella pneumoniae 1 Klebsiella pneumoniae 1 NA

GCF_001034165 Scaffold Klebsiella pneumoniae UCI75 Klebsiella pneumoniae 1 Klebsiella pneumoniae 1 Klebsiella pneumoniae 1 NA

GCF_001034205 Scaffold Klebsiella pneumoniae UCI76 Klebsiella pneumoniae 1 Klebsiella pneumoniae 1 Klebsiella pneumoniae 1 NA

GCF_001034215 Scaffold Klebsiella pneumoniae UCI81 Klebsiella pneumoniae 1 Klebsiella pneumoniae 1 Klebsiella pneumoniae 1 NA

GCF_001034235 Scaffold Klebsiella pneumoniae UCI82 Klebsiella pneumoniae 1 Klebsiella pneumoniae 1 Klebsiella pneumoniae 1 NA

GCF_001034245 Scaffold Klebsiella pneumoniae UCI91 Klebsiella pneumoniae 1 Klebsiella pneumoniae 1 Klebsiella pneumoniae 1 NA

GCF_001034285 Scaffold Klebsiella pneumoniae UCI92 Klebsiella pneumoniae 1 Klebsiella pneumoniae 1 Klebsiella pneumoniae 1 NA

GCF_001034295 Scaffold Klebsiella pneumoniae UCI93 Klebsiella pneumoniae 1 Klebsiella pneumoniae 1 Klebsiella pneumoniae 1 NA

GCF_001034305 Scaffold Klebsiella pneumoniae UCI94 Klebsiella pneumoniae 1 Klebsiella pneumoniae 1 Klebsiella pneumoniae 1 NA

GCF_001034325 Scaffold Klebsiella pneumoniae UCI95 Klebsiella pneumoniae 1 Klebsiella pneumoniae 1 Klebsiella pneumoniae 1 NA

GCF_001034365 Scaffold Klebsiella pneumoniae UCI96 Klebsiella pneumoniae 1 Klebsiella pneumoniae 1 Klebsiella pneumoniae 1 NA

GCF_001038305 Scaffold Klebsiella michiganensis GY84G39 Klebsiella michiganensis 1 Klebsiella michiganensis 1 Klebsiella michiganensis 1 NA

GCF_001038365 Contig Klebsiella pneumoniae JS86C16 Klebsiella pneumoniae 1 Klebsiella pneumoniae 1 Klebsiella pneumoniae 1 NA

GCF_001051455 Contig Klebsiella michiganensis CH5 Klebsiella michiganensis 1 Klebsiella michiganensis 1 Klebsiella michiganensis 1 NA

GCF_001052035 Contig Klebsiella michiganensis 1081 KOXY Klebsiella michiganensis 1 Klebsiella michiganensis 1 Klebsiella michiganensis 1 NA

GCF_001052045 Contig Klebsiella michiganensis 1082 KOXY Klebsiella michiganensis 1 Klebsiella michiganensis 1 Klebsiella michiganensis 1 NA

GCF_001052095 Scaffold Klebsiella aerogenes 1020 EAER Klebsiella aerogenes 1 Klebsiella aerogenes Klebsiella aerogenes NA

GCF_001052235 Scaffold Klebsiella oxytoca 1148 KOXY Klebsiella grimontii Klebsiella grimontii Klebsiella grimontii NA

GCF_001052475 Scaffold Klebsiella pneumoniae 1236 KOXY Klebsiella pneumoniae 1 Klebsiella pneumoniae 1 Klebsiella pneumoniae 1 NA

GCF_001052515 Scaffold Klebsiella pneumoniae pneumoniae 1243 CKOS Klebsiella pneumoniae 1 Klebsiella pneumoniae 1 Klebsiella pneumoniae 1 NA

GCF_001052525 Scaffold Klebsiella michiganensis 1246 KOXY Klebsiella michiganensis 1 Klebsiella michiganensis 1 Klebsiella michiganensis 1 NA

GCF_001052565 Scaffold Klebsiella aerogenes 1277 EAER Klebsiella aerogenes 1 Klebsiella aerogenes Klebsiella aerogenes NA

GCF_001052785 Contig Klebsiella michiganensis 1084 KOXY Klebsiella michiganensis 1 Klebsiella michiganensis 1 Klebsiella michiganensis 1 NA

GCF_001052825 Scaffold Klebsiella michiganensis 1154 KOXY Klebsiella grimontii Klebsiella grimontii Klebsiella grimontii NA

GCF_001053235 Scaffold Klebsiella aerogenes 1282 EAER Klebsiella aerogenes 1 Klebsiella aerogenes Klebsiella aerogenes NA

GCF_001053595 Scaffold Klebsiella aerogenes 1019 EAER Klebsiella aerogenes 1 Klebsiella aerogenes Klebsiella aerogenes NA

GCF_001053665 Scaffold Klebsiella michiganensis 1037 KOXY Klebsiella grimontii Klebsiella grimontii Klebsiella grimontii NA

GCF_001053715 Scaffold Klebsiella oxytoca 1047 KOXY Klebsiella oxytoca 1 Klebsiella oxytoca 1 Klebsiella oxytoca 1 NA

GCF_001053845 Contig Klebsiella michiganensis 1085 KOXY Klebsiella michiganensis 1 Klebsiella michiganensis 1 Klebsiella michiganensis 1 NA

GCF_001053875 Contig Klebsiella michiganensis 1113 KOXY Klebsiella michiganensis 1 Klebsiella michiganensis 1 Klebsiella michiganensis 1 NA

GCF_001054275 Scaffold Klebsiella aerogenes 1278 EAER Klebsiella aerogenes 1 Klebsiella aerogenes Klebsiella aerogenes NA

GCF_001054315 Contig Klebsiella aerogenes 1281 EAER Klebsiella aerogenes 1 Klebsiella aerogenes Klebsiella aerogenes NA

GCF_001054405 Scaffold Klebsiella aerogenes 151 EAER Klebsiella aerogenes 1 Klebsiella aerogenes Klebsiella aerogenes NA

GCF_001054575 Scaffold Klebsiella oxytoca 197 KOXY Klebsiella oxytoca 1 Klebsiella oxytoca 1 Klebsiella oxytoca 1 NA

GCF_001054755 Scaffold Klebsiella michiganensis 247 KOXY Klebsiella michiganensis 1 Klebsiella michiganensis 1 Klebsiella michiganensis 1 NA

GCF_001054935 Scaffold Klebsiella oxytoca 346 KOXY Klebsiella oxytoca 1 Klebsiella oxytoca 1 Klebsiella oxytoca 1 NA

GCF_001054995 Scaffold Klebsiella michiganensis 371 KOXY Klebsiella grimontii Klebsiella grimontii Klebsiella grimontii NA

GCF_001055015 Scaffold Klebsiella pneumoniae pneumoniae 383 ECLO Klebsiella pneumoniae 1 Klebsiella pneumoniae 1 Klebsiella pneumoniae 1 NA

GCF_001055555 Contig Klebsiella aerogenes 170 EAER Klebsiella aerogenes 1 Klebsiella aerogenes Klebsiella aerogenes NA

GCF_001055635 Scaffold Klebsiella oxytoca 181 KOXY Klebsiella oxytoca 1 Klebsiella oxytoca 1 Klebsiella oxytoca 1 NA

GCF_001055795 Scaffold Klebsiella quasipneumoniae similipneumoniae 193 KOXY Klebsiella quasipneumoniae 1 Klebsiella quasipneumoniae 1 Klebsiella quasipneumoniae NA

GCF_001056275 Scaffold Klebsiella pneumoniae pneumoniae 291 EBAC Klebsiella pneumoniae 1 Klebsiella pneumoniae 1 Klebsiella pneumoniae 1 NA

GCF_001056405 Scaffold Klebsiella michiganensis 337 KOXY Klebsiella michiganensis 1 Klebsiella michiganensis 1 Klebsiella michiganensis 1 NA

GCF_001056445 Scaffold Klebsiella michiganensis 345 KOXY Klebsiella michiganensis 1 Klebsiella michiganensis 1 Klebsiella michiganensis 1 NA

GCF_001056485 Scaffold Klebsiella pneumoniae ozaenae 359 ECLO Klebsiella pneumoniae 1 Klebsiella pneumoniae 1 Klebsiella pneumoniae 1 NA

GCF_001056525 Scaffold Klebsiella quasipneumoniae similipneumoniae 385 ECLO Klebsiella quasipneumoniae 1 Klebsiella quasipneumoniae 1 Klebsiella quasipneumoniae NA

GCF_001056765 Scaffold Klebsiella michiganensis 442 KOXY Klebsiella michiganensis 1 Klebsiella michiganensis 1 Klebsiella michiganensis 1 NA

GCF_001057405 Scaffold Klebsiella oxytoca 582 KOXY Klebsiella oxytoca 1 Klebsiella oxytoca 1 Klebsiella oxytoca 1 NA

GCF_001057685 Contig Klebsiella michiganensis 625 KOXY Klebsiella pasteurii Klebsiella pasteurii Klebsiella grimontii NA

GCF_001057945 Contig Klebsiella aerogenes 682 EAER Klebsiella aerogenes 1 Klebsiella aerogenes Klebsiella aerogenes NA

GCF_001058365 Scaffold Klebsiella pneumoniae pneumoniae 76 ECLO Klebsiella pneumoniae 1 Klebsiella pneumoniae 1 Klebsiella pneumoniae 1 NA

GCF_001058645 Scaffold Klebsiella aerogenes 86 EAER Klebsiella aerogenes 1 Klebsiella aerogenes Klebsiella aerogenes NA

GCF_001059975 Contig Klebsiella aerogenes 965 EAER Klebsiella aerogenes 1 Klebsiella aerogenes Klebsiella aerogenes NA

GCF_001060105 Scaffold Klebsiella pneumoniae 1012 KPNE Klebsiella pneumoniae 1 Klebsiella pneumoniae 1 Klebsiella pneumoniae 1 NA

GCF_001060275 Scaffold Klebsiella pneumoniae 1098 KPNE Klebsiella pneumoniae 1 Klebsiella pneumoniae 1 Klebsiella pneumoniae 1 NA

GCF_001060405 Scaffold Klebsiella michiganensis 1169 SBOY Klebsiella grimontii Klebsiella grimontii Klebsiella grimontii NA

GCF_001060495 Scaffold Klebsiella pneumoniae 1183 KPNE Klebsiella pneumoniae 1 Klebsiella pneumoniae 1 Klebsiella pneumoniae 1 NA

GCF_001060825 Contig Klebsiella quasipneumoniae similipneumoniae 1294 KPNE Klebsiella quasipneumoniae 1 Klebsiella quasipneumoniae 1 Klebsiella quasipneumoniae NA

GCF_001060895 Scaffold Klebsiella pneumoniae 1041 KPNE Klebsiella pneumoniae 1 Klebsiella pneumoniae 1 Klebsiella pneumoniae 1 NA

GCF_001061315 Scaffold Klebsiella pneumoniae 1239 KPNE Klebsiella pneumoniae 1 Klebsiella pneumoniae 1 Klebsiella pneumoniae 1 NA

GCF_001061605 Scaffold Klebsiella pneumoniae 1331 KPNE Klebsiella pneumoniae 1 Klebsiella pneumoniae 1 Klebsiella pneumoniae 1 NA

GCF_001061725 Scaffold Klebsiella pneumoniae 1010 KPNE Klebsiella pneumoniae 1 Klebsiella pneumoniae 1 Klebsiella pneumoniae 1 NA

GCF_001061755 Contig Klebsiella pneumoniae 1035 KPNE Klebsiella pneumoniae 1 Klebsiella pneumoniae 1 Klebsiella pneumoniae 1 NA

GCF_001061805 Scaffold Klebsiella pneumoniae pneumoniae 1050 SSON Klebsiella pneumoniae 1 Klebsiella pneumoniae 1 Klebsiella pneumoniae 1 NA

GCF_001062535 Scaffold Klebsiella pneumoniae 1263 KPNE Klebsiella pneumoniae 1 Klebsiella pneumoniae 1 Klebsiella pneumoniae 1 NA

GCF_001062915 Scaffold Klebsiella pneumoniae 194 KPNE Klebsiella pneumoniae 1 Klebsiella pneumoniae 1 Klebsiella pneumoniae 1 NA

GCF_001063525 Scaffold Klebsiella pneumoniae 500 KPNE Klebsiella pneumoniae 1 Klebsiella pneumoniae 1 Klebsiella pneumoniae 1 NA

GCF_001063755 Scaffold Klebsiella pneumoniae 570 KPNE Klebsiella pneumoniae 1 Klebsiella pneumoniae 1 Klebsiella pneumoniae 1 NA

GCF_001063775 Contig Klebsiella oxytoca 588 KOXY Klebsiella oxytoca 1 Klebsiella oxytoca 1 Klebsiella oxytoca 1 NA

GCF_001063935 Scaffold Klebsiella pneumoniae 187 KPNE Klebsiella pneumoniae 1 Klebsiella pneumoniae 1 Klebsiella pneumoniae 1 NA

GCF_001064035 Scaffold Klebsiella pneumoniae 221 KPNE Klebsiella pneumoniae 1 Klebsiella pneumoniae 1 Klebsiella pneumoniae 1 NA

GCF_001064955 Scaffold Klebsiella pneumoniae 436 KPNE Klebsiella pneumoniae 1 Klebsiella pneumoniae 1 Klebsiella pneumoniae 1 NA

GCF_001065235 Scaffold Klebsiella pneumoniae 501 KPNE Klebsiella pneumoniae 1 Klebsiella pneumoniae 1 Klebsiella pneumoniae 1 NA

GCF_001065705 Scaffold Klebsiella michiganensis 628 KOXY Klebsiella pasteurii Klebsiella pasteurii Klebsiella grimontii NA

GCF_001065715 Scaffold Klebsiella oxytoca 633 KOXY Klebsiella oxytoca 1 Klebsiella oxytoca 1 Klebsiella oxytoca 1 NA

GCF_001065765 Scaffold Klebsiella michiganensis 636 KOXY Klebsiella grimontii Klebsiella grimontii Klebsiella grimontii NA

GCF_001065795 Contig Klebsiella pneumoniae 648 KPNE Klebsiella pneumoniae 1 Klebsiella pneumoniae 1 Klebsiella pneumoniae 1 NA

GCF_001065835 Scaffold Klebsiella variicola 653 KVAR Klebsiella variicola Klebsiella variicola Klebsiella variicola NA

GCF_001065855 Scaffold Klebsiella variicola 671 KVAR Klebsiella variicola Klebsiella variicola Klebsiella variicola NA

GCF_001066095 Contig Klebsiella pneumoniae 731 KPNE Klebsiella pneumoniae 1 Klebsiella pneumoniae 1 Klebsiella pneumoniae 1 NA

GCF_001066115 Contig Klebsiella pneumoniae 733 KPNE Klebsiella pneumoniae 1 Klebsiella pneumoniae 1 Klebsiella pneumoniae 1 NA

GCF_001066155 Contig Klebsiella pneumoniae 741 KPNE Klebsiella pneumoniae 1 Klebsiella pneumoniae 1 Klebsiella pneumoniae 1 NA

GCF_001066165 Contig Klebsiella pneumoniae 743 KPNE Klebsiella pneumoniae 1 Klebsiella pneumoniae 1 Klebsiella pneumoniae 1 NA

GCF_001066265 Scaffold Klebsiella variicola 780 KPNE Klebsiella variicola Klebsiella variicola Klebsiella variicola NA

GCF_001066315 Contig Klebsiella variicola 789 KVAR Klebsiella variicola Klebsiella variicola Klebsiella variicola NA

GCF_001066415 Contig Klebsiella pneumoniae 813 KPNE Klebsiella pneumoniae 1 Klebsiella pneumoniae 1 Klebsiella pneumoniae 1 NA

GCF_001066505 Scaffold Klebsiella variicola 833 KPNE Klebsiella variicola Klebsiella variicola Klebsiella variicola NA

GCF_001066535 Scaffold Klebsiella variicola 835 KPNE Klebsiella variicola Klebsiella variicola Klebsiella variicola NA

GCF_001066555 Scaffold Klebsiella variicola 836 KPNE Klebsiella variicola Klebsiella variicola Klebsiella variicola NA

GCF_001066585 Scaffold Klebsiella pneumoniae 845 KPNE Klebsiella pneumoniae 1 Klebsiella pneumoniae 1 Klebsiella pneumoniae 1 NA

GCF_001066725 Contig Klebsiella pneumoniae 887 KPNE Klebsiella pneumoniae 1 Klebsiella pneumoniae 1 Klebsiella pneumoniae 1 NA

GCF_001066775 Scaffold Klebsiella michiganensis 649 KOXY Klebsiella grimontii Klebsiella grimontii Klebsiella grimontii NA

GCF_001066805 Scaffold Klebsiella michiganensis 659 KOXY Klebsiella michiganensis 1 Klebsiella michiganensis 1 Klebsiella michiganensis 1 NA

GCF_001067135 Scaffold Klebsiella pneumoniae 755rep1 KPNE Klebsiella pneumoniae 1 Klebsiella pneumoniae 1 Klebsiella pneumoniae 1 NA

GCF_001067155 Contig Klebsiella pneumoniae 755rep2 KPNE Klebsiella pneumoniae 1 Klebsiella pneumoniae 1 Klebsiella pneumoniae 1 NA

GCF_001067475 Scaffold Klebsiella variicola 834 KPNE Klebsiella variicola Klebsiella variicola Klebsiella variicola NA

GCF_001067495 Scaffold Klebsiella variicola 837 KPNE Klebsiella variicola Klebsiella variicola Klebsiella variicola NA

GCF_001067575 Contig Klebsiella pneumoniae 863 KPNE Klebsiella pneumoniae 1 Klebsiella pneumoniae 1 Klebsiella pneumoniae 1 NA

GCF_001067585 Contig Klebsiella quasipneumoniae quasipneumoniae 865 KPNE Klebsiella quasipneumoniae 2 Klebsiella quasipneumoniae 2 Klebsiella quasipneumoniae NA

GCF_001067895 Contig Klebsiella pneumoniae 913 KPNE Klebsiella pneumoniae 1 Klebsiella pneumoniae 1 Klebsiella pneumoniae 1 NA

GCF_001067955 Contig Klebsiella pneumoniae 930 KPNE Klebsiella pneumoniae 1 Klebsiella pneumoniae 1 Klebsiella pneumoniae 1 NA

GCF_001067975 Contig Klebsiella pneumoniae ozaenae 977rep1 SDYS Klebsiella pneumoniae 1 Klebsiella pneumoniae 1 Klebsiella pneumoniae 1 NA

GCF_001068035 Contig Klebsiella pneumoniae 98 KPNE Klebsiella pneumoniae 1 Klebsiella pneumoniae 1 Klebsiella pneumoniae 1 NA

GCF_001068335 Contig Klebsiella pneumoniae ozaenae 977rep2 SDYS Klebsiella pneumoniae 1 Klebsiella pneumoniae 1 Klebsiella pneumoniae 1 NA

GCF_001068855 Scaffold Klebsiella variicola 124 ECLO Klebsiella variicola Klebsiella variicola Klebsiella variicola NA

GCF_001070955 Scaffold Klebsiella michiganensis 375 KOXY Klebsiella grimontii Klebsiella grimontii Klebsiella grimontii NA

GCF_001071835 Scaffold Klebsiella aerogenes 225 EAER Klebsiella aerogenes 2 Klebsiella aerogenes Klebsiella aerogenes NA

GCF_001072735 Scaffold Klebsiella michiganensis 397 KOXY Klebsiella grimontii Klebsiella grimontii Klebsiella grimontii NA

GCF_001072835 Scaffold Klebsiella michiganensis 409 KOXY Klebsiella grimontii Klebsiella grimontii Klebsiella grimontii NA

GCF_001076365 Scaffold Klebsiella variicola 1335 KPNE Klebsiella variicola Klebsiella variicola Klebsiella variicola NA

GCF_001076525 Scaffold Klebsiella variicola 521 SSON Klebsiella variicola Klebsiella variicola Klebsiella variicola NA

GCF_001076675 Scaffold Klebsiella pneumoniae pneumoniae 298 SBOY Klebsiella pneumoniae 1 Klebsiella pneumoniae 1 Klebsiella pneumoniae 1 NA

GCF_001076805 Scaffold Klebsiella michiganensis 452 SSON Klebsiella grimontii Klebsiella grimontii Klebsiella grimontii NA

GCF_001076915 Scaffold Klebsiella variicola 524 SBOY Klebsiella variicola Klebsiella variicola Klebsiella variicola NA

GCF_001077175 Scaffold Klebsiella michiganensis 793 KOXY Klebsiella michiganensis 1 Klebsiella michiganensis 1 Klebsiella michiganensis 1 NA

GCF_001077215 Contig Klebsiella pneumoniae 912 KPNE Klebsiella pneumoniae 1 Klebsiella pneumoniae 1 Klebsiella pneumoniae 1 NA

GCF_001078175 Scaffold Klebsiella oxytoca 09-7231 Klebsiella oxytoca 1 Klebsiella oxytoca 1 Klebsiella oxytoca 1 NA

GCF_001078195 Scaffold Klebsiella oxytoca 10-5244 Klebsiella oxytoca 1 Klebsiella oxytoca 1 Klebsiella oxytoca 1 NA

GCF_001078235 Scaffold Klebsiella oxytoca 10-5248 Klebsiella oxytoca 1 Klebsiella oxytoca 1 Klebsiella oxytoca 1 NA

GCF_001078255 Scaffold Klebsiella oxytoca 10-5249 Klebsiella oxytoca 1 Klebsiella oxytoca 1 Klebsiella oxytoca 1 NA

GCF_001182385 Contig Klebsiella pneumoniae KpVA-1 Klebsiella pneumoniae 1 Klebsiella pneumoniae 1 Klebsiella pneumoniae 1 NA

GCF_001182405 Contig Klebsiella pneumoniae KpVA-2 Klebsiella pneumoniae 1 Klebsiella pneumoniae 1 Klebsiella pneumoniae 1 NA

GCF_001182425 Contig Klebsiella pneumoniae KpVA-3 Klebsiella pneumoniae 1 Klebsiella pneumoniae 1 Klebsiella pneumoniae 1 NA

GCF_001182445 Contig Klebsiella pneumoniae KpVA-4 Klebsiella pneumoniae 1 Klebsiella pneumoniae 1 Klebsiella pneumoniae 1 NA

GCF_001182465 Contig Klebsiella pneumoniae KpVA-5 Klebsiella pneumoniae 1 Klebsiella pneumoniae 1 Klebsiella pneumoniae 1 NA

GCF_001182485 Contig Klebsiella pneumoniae KpVA-6 Klebsiella pneumoniae 1 Klebsiella pneumoniae 1 Klebsiella pneumoniae 1 NA

GCF_001182505 Contig Klebsiella pneumoniae KpVA-7 Klebsiella pneumoniae 1 Klebsiella pneumoniae 1 Klebsiella pneumoniae 1 NA

GCF_001182525 Contig Klebsiella pneumoniae KpVA-8 Klebsiella pneumoniae 1 Klebsiella pneumoniae 1 Klebsiella pneumoniae 1 NA

GCF_001182545 Contig Klebsiella pneumoniae KpVA-9 Klebsiella pneumoniae 1 Klebsiella pneumoniae 1 Klebsiella pneumoniae 1 NA

GCF_001182565 Contig Klebsiella pneumoniae KpVA-10 Klebsiella pneumoniae 1 Klebsiella pneumoniae 1 Klebsiella pneumoniae 1 NA

GCF_001182585 Contig Klebsiella pneumoniae KpVA-11 Klebsiella pneumoniae 1 Klebsiella pneumoniae 1 Klebsiella pneumoniae 1 NA

GCF_001182605 Contig Klebsiella pneumoniae KpVA-12 Klebsiella pneumoniae 1 Klebsiella pneumoniae 1 Klebsiella pneumoniae 1 NA

GCF_001182625 Contig Klebsiella pneumoniae KpVA-13 Klebsiella pneumoniae 1 Klebsiella pneumoniae 1 Klebsiella pneumoniae 1 NA

GCF_001182645 Contig Klebsiella pneumoniae KpVA-14 Klebsiella pneumoniae 1 Klebsiella pneumoniae 1 Klebsiella pneumoniae 1 NA

GCF_001182665 Contig Klebsiella pneumoniae KpVA-15 Klebsiella pneumoniae 1 Klebsiella pneumoniae 1 Klebsiella pneumoniae 1 NA

GCF_001182685 Scaffold Klebsiella pneumoniae KpVA-16 Klebsiella pneumoniae 1 Klebsiella pneumoniae 1 Klebsiella pneumoniae 1 NA

GCF_001185665 Complete Klebsiella pneumoniae MGH83 Klebsiella pneumoniae 1 Klebsiella pneumoniae 1 Klebsiella pneumoniae 1 NA

GCF_001185675 Scaffold Klebsiella pneumoniae CHS240 Klebsiella pneumoniae 1 Klebsiella pneumoniae 1 Klebsiella pneumoniae 1 NA

GCF_001185685 Scaffold Klebsiella pneumoniae UCI69 Klebsiella pneumoniae 1 Klebsiella pneumoniae 1 Klebsiella pneumoniae 1 NA

GCF_001185695 Scaffold Klebsiella pneumoniae CHS239 Klebsiella pneumoniae 1 Klebsiella pneumoniae 1 Klebsiella pneumoniae 1 NA

GCF_001185745 Scaffold Klebsiella pneumoniae CHS238 Klebsiella pneumoniae 1 Klebsiella pneumoniae 1 Klebsiella pneumoniae 1 NA

GCF_001185755 Scaffold Klebsiella pneumoniae CHS212 Klebsiella pneumoniae 1 Klebsiella pneumoniae 1 Klebsiella pneumoniae 1 NA

GCF_001185765 Scaffold Klebsiella pneumoniae CHS198 Klebsiella pneumoniae 1 Klebsiella pneumoniae 1 Klebsiella pneumoniae 1 NA

GCF_001185775 Scaffold Klebsiella pneumoniae CHS196 Klebsiella pneumoniae 1 Klebsiella pneumoniae 1 Klebsiella pneumoniae 1 NA

GCF_001185825 Scaffold Klebsiella pneumoniae CHS187 Klebsiella pneumoniae 1 Klebsiella pneumoniae 1 Klebsiella pneumoniae 1 NA

GCF_001185835 Scaffold Klebsiella pneumoniae CHS178 Klebsiella pneumoniae 1 Klebsiella pneumoniae 1 Klebsiella pneumoniae 1 NA

GCF_001185865 Scaffold Klebsiella pneumoniae CHS175 Klebsiella pneumoniae 1 Klebsiella pneumoniae 1 Klebsiella pneumoniae 1 NA

GCF_001185895 Scaffold Klebsiella pneumoniae CHS171 Klebsiella pneumoniae 1 Klebsiella pneumoniae 1 Klebsiella pneumoniae 1 NA

GCF_001185915 Scaffold Klebsiella pneumoniae CHS136 Klebsiella pneumoniae 1 Klebsiella pneumoniae 1 Klebsiella pneumoniae 1 NA

GCF_001185925 Scaffold Klebsiella pneumoniae CHS130 Klebsiella pneumoniae 1 Klebsiella pneumoniae 1 Klebsiella pneumoniae 1 NA

GCF_001185945 Scaffold Klebsiella pneumoniae CHS124 Klebsiella pneumoniae 1 Klebsiella pneumoniae 1 Klebsiella pneumoniae 1 NA

GCF_001185955 Scaffold Klebsiella pneumoniae CHS120 Klebsiella pneumoniae 1 Klebsiella pneumoniae 1 Klebsiella pneumoniae 1 NA

GCF_001185985 Scaffold Klebsiella pneumoniae CHS119 Klebsiella pneumoniae 1 Klebsiella pneumoniae 1 Klebsiella pneumoniae 1 NA

GCF_001186005 Scaffold Klebsiella pneumoniae CHS108 Klebsiella pneumoniae 1 Klebsiella pneumoniae 1 Klebsiella pneumoniae 1 NA

GCF_001186025 Scaffold Klebsiella pneumoniae CHS103 Klebsiella pneumoniae 1 Klebsiella pneumoniae 1 Klebsiella pneumoniae 1 NA

GCF_001186035 Scaffold Klebsiella pneumoniae CHS87 Klebsiella pneumoniae 1 Klebsiella pneumoniae 1 Klebsiella pneumoniae 1 NA

GCF_001186065 Scaffold Klebsiella pneumoniae CHS86 Klebsiella pneumoniae 1 Klebsiella pneumoniae 1 Klebsiella pneumoniae 1 NA

GCF_001187805 Contig Klebsiella variicola NL49 Klebsiella variicola Klebsiella variicola Klebsiella variicola NA

GCF_001187825 Contig Klebsiella variicola NL58 Klebsiella variicola Klebsiella variicola Klebsiella variicola NA

GCF_001187865 Contig Klebsiella sp RIT-PI-d Unspecified 3 Unspecified 3 Unspecified 2 NA

GCF_001208885 Contig Klebsiella quasipneumoniae quasipneumoniae FI HV2014 Klebsiella quasipneumoniae 2 Klebsiella quasipneumoniae 2 Klebsiella quasipneumoniae NA

GCF_001238585 Scaffold Klebsiella pneumoniae ST258 Klebsiella pneumoniae 1 Klebsiella pneumoniae 1 Klebsiella pneumoniae 1 NA

GCF_001261795 Scaffold Klebsiella variicola T29A Klebsiella variicola Klebsiella variicola Klebsiella variicola NA

GCF_001261815 Contig Klebsiella variicola 3 Klebsiella variicola Klebsiella variicola Klebsiella variicola NA

GCF_001261835 Scaffold Klebsiella variicola 6A2 Klebsiella variicola Klebsiella variicola Klebsiella variicola NA

GCF_001261855 Contig Klebsiella variicola 4880 Klebsiella variicola Klebsiella variicola Klebsiella variicola NA

GCF_001261875 Contig Klebsiella variicola 06-268 Klebsiella variicola Klebsiella variicola Klebsiella variicola NA

GCF_001275285 Contig Klebsiella pneumoniae SY-A Klebsiella pneumoniae 1 Klebsiella pneumoniae 1 Klebsiella pneumoniae 1 NA

GCF_001275295 Contig Klebsiella pneumoniae SY-B Klebsiella pneumoniae 1 Klebsiella pneumoniae 1 Klebsiella pneumoniae 1 NA

GCF_001275305 Contig Klebsiella pneumoniae SY-B2 Klebsiella pneumoniae 1 Klebsiella pneumoniae 1 Klebsiella pneumoniae 1 NA

GCF_001278905 Complete Klebsiella quasipneumoniae HKUOPLA Klebsiella quasipneumoniae 1 Klebsiella quasipneumoniae 1 Klebsiella quasipneumoniae NA

GCF_001280925 Complete Klebsiella quasipneumoniae HKUOPLC Klebsiella quasipneumoniae 1 Klebsiella quasipneumoniae 1 Klebsiella quasipneumoniae NA

GCF_001307175 Complete Klebsiella pneumoniae KP617 Klebsiella pneumoniae 1 Klebsiella pneumoniae 1 Klebsiella pneumoniae 1 NA

GCF_001308905 Scaffold Klebsiella sp AA405 Klebsiella pneumoniae 1 Klebsiella pneumoniae 1 Klebsiella pneumoniae 1 NA

GCF_001316495 Contig Klebsiella pneumoniae pneumoniae ST101 950171785 Klebsiella pneumoniae 1 Klebsiella pneumoniae 1 Klebsiella pneumoniae 1 NA

GCF_001316565 Complete Klebsiella pneumoniae pneumoniae ST101 960186733 Klebsiella pneumoniae 1 Klebsiella pneumoniae 1 Klebsiella pneumoniae 1 NA

GCF_001316645 Contig Klebsiella pneumoniae pneumoniae ST101 951373950 Klebsiella pneumoniae 1 Klebsiella pneumoniae 1 Klebsiella pneumoniae 1 NA

GCF_001316695 Contig Klebsiella pneumoniae pneumoniae ST101 945165838 Klebsiella pneumoniae 1 Klebsiella pneumoniae 1 Klebsiella pneumoniae 1 NA

GCF_001316745 Contig Klebsiella pneumoniae pneumoniae ST101 945169659 Klebsiella pneumoniae 1 Klebsiella pneumoniae 1 Klebsiella pneumoniae 1 NA

GCF_001316765 Contig Klebsiella pneumoniae pneumoniae ST2017 950118422 Klebsiella pneumoniae 1 Klebsiella pneumoniae 1 Klebsiella pneumoniae 1 NA

GCF_001316785 Contig Klebsiella pneumoniae pneumoniae ST101 950173000 Klebsiella pneumoniae 1 Klebsiella pneumoniae 1 Klebsiella pneumoniae 1 NA

GCF_001316845 Contig Klebsiella pneumoniae pneumoniae ST323 941530379 Klebsiella pneumoniae 1 Klebsiella pneumoniae 1 Klebsiella pneumoniae 1 NA

GCF_001316865 Contig Klebsiella pneumoniae pneumoniae ST14 944535499 Klebsiella pneumoniae 1 Klebsiella pneumoniae 1 Klebsiella pneumoniae 1 NA

GCF_001316895 Contig Klebsiella pneumoniae pneumoniae ST2016 951362657 Klebsiella pneumoniae 1 Klebsiella pneumoniae 1 Klebsiella pneumoniae 1 NA

GCF_001316925 Contig Klebsiella pneumoniae pneumoniae ST2017 950117510 Klebsiella pneumoniae 1 Klebsiella pneumoniae 1 Klebsiella pneumoniae 1 NA

GCF_001316945 Chromosome Klebsiella pneumoniae pneumoniae ST2017 950142398 Klebsiella pneumoniae 1 Klebsiella pneumoniae 1 Klebsiella pneumoniae 1 NA

GCF_001316955 Contig Klebsiella pneumoniae pneumoniae ST101 957083896 Klebsiella pneumoniae 1 Klebsiella pneumoniae 1 Klebsiella pneumoniae 1 NA

GCF_001316985 Contig Klebsiella pneumoniae pneumoniae ST101 951384356 Klebsiella pneumoniae 1 Klebsiella pneumoniae 1 Klebsiella pneumoniae 1 NA

GCF_001317005 Contig Klebsiella pneumoniae pneumoniae ST101 957089165 Klebsiella pneumoniae 1 Klebsiella pneumoniae 1 Klebsiella pneumoniae 1 NA

GCF_001317015 Contig Klebsiella pneumoniae pneumoniae ST101 957083320 Klebsiella pneumoniae 1 Klebsiella pneumoniae 1 Klebsiella pneumoniae 1 NA

GCF_001317035 Contig Klebsiella pneumoniae pneumoniae ST101 U44822 26/7/13 Klebsiella pneumoniae 1 Klebsiella pneumoniae 1 Klebsiella pneumoniae 1 NA

GCF_001317095 Contig Klebsiella variicola ST1478 939997103 Klebsiella variicola Klebsiella variicola Klebsiella variicola NA

GCF_001317165 Contig Klebsiella pneumoniae pneumoniae ST101 939996824 Klebsiella pneumoniae 1 Klebsiella pneumoniae 1 Klebsiella pneumoniae 1 NA

GCF_001317245 Contig Klebsiella michiganensis ST170 939742031 Klebsiella michiganensis 1 Klebsiella michiganensis 1 Klebsiella michiganensis 1 NA

GCF_001317295 Contig Klebsiella pneumoniae pneumoniae ST101 951363981 Klebsiella pneumoniae 1 Klebsiella pneumoniae 1 Klebsiella pneumoniae 1 NA

GCF_001317375 Contig Klebsiella pneumoniae pneumoniae ST101 945154233 Klebsiella pneumoniae 1 Klebsiella pneumoniae 1 Klebsiella pneumoniae 1 NA

GCF_001373075 Contig Klebsiella quasipneumoniae 45T1-2A Klebsiella quasipneumoniae 1 Klebsiella quasipneumoniae 1 Klebsiella quasipneumoniae NA

GCF_001399815 Contig Klebsiella pneumoniae pneumoniae KK207/1 Klebsiella pneumoniae 1 Klebsiella pneumoniae 1 Klebsiella pneumoniae 1 NA

GCF_001404095 Contig Klebsiella pneumoniae CH1034 Klebsiella pneumoniae 1 Klebsiella pneumoniae 1 Klebsiella pneumoniae 1 NA

GCF_001441285 Scaffold Klebsiella pneumoniae B86 Klebsiella pneumoniae 1 Klebsiella pneumoniae 1 Klebsiella pneumoniae 1 NA

GCF_001441295 Scaffold Klebsiella pneumoniae B199 Klebsiella pneumoniae 1 Klebsiella pneumoniae 1 Klebsiella pneumoniae 1 NA

GCF_001443245 Contig Klebsiella sp KGM-IMP216 Klebsiella pneumoniae 1 Klebsiella pneumoniae 1 Klebsiella pneumoniae 1 NA

GCF_001455995 Complete Klebsiella pneumoniae MS6671 Klebsiella pneumoniae 1 Klebsiella pneumoniae 1 Klebsiella pneumoniae 1 NA

GCF_001456055 Complete Klebsiella pneumoniae KpN01 Klebsiella pneumoniae 1 Klebsiella pneumoniae 1 Klebsiella pneumoniae 1 NA

GCF_001456095 Complete Klebsiella pneumoniae KpN06 Klebsiella pneumoniae 1 Klebsiella pneumoniae 1 Klebsiella pneumoniae 1 NA

GCF_001456135 Complete Klebsiella pneumoniae CAV1193 Klebsiella pneumoniae 1 Klebsiella pneumoniae 1 Klebsiella pneumoniae 1 NA

GCF_001457255 Scaffold Klebsiella pneumoniae KPOI1/1 Klebsiella pneumoniae 1 Klebsiella pneumoniae 1 Klebsiella pneumoniae 1 NA

GCF_001457265 Scaffold Klebsiella pneumoniae KPOI1/2 Klebsiella pneumoniae 1 Klebsiella pneumoniae 1 Klebsiella pneumoniae 1 NA

GCF_001457275 Scaffold Klebsiella pneumoniae KPOI1/3 Klebsiella pneumoniae 1 Klebsiella pneumoniae 1 Klebsiella pneumoniae 1 NA

GCF_001457285 Contig Klebsiella pneumoniae KPOI1/4 Klebsiella pneumoniae 1 Klebsiella pneumoniae 1 Klebsiella pneumoniae 1 NA

GCF_001457335 Contig Klebsiella pneumoniae KPOI2 Klebsiella pneumoniae 1 Klebsiella pneumoniae 1 Klebsiella pneumoniae 1 NA

GCF_001460475 Contig Klebsiella pneumoniae KPOI5 Klebsiella pneumoniae 1 Klebsiella pneumoniae 1 Klebsiella pneumoniae 1 NA

GCF_001460495 Contig Klebsiella pneumoniae KPEI2 Klebsiella pneumoniae 1 Klebsiella pneumoniae 1 Klebsiella pneumoniae 1 NA

GCF_001460505 Scaffold Klebsiella pneumoniae KP11U Klebsiella pneumoniae 1 Klebsiella pneumoniae 1 Klebsiella pneumoniae 1 NA

GCF_001460565 Scaffold Klebsiella pneumoniae KPOI3 Klebsiella pneumoniae 1 Klebsiella pneumoniae 1 Klebsiella pneumoniae 1 NA

GCF_001460575 Scaffold Klebsiella pneumoniae KP54M Klebsiella pneumoniae 1 Klebsiella pneumoniae 1 Klebsiella pneumoniae 1 NA

GCF_001460975 Contig Klebsiella pneumoniae KPEI1 Klebsiella pneumoniae 1 Klebsiella pneumoniae 1 Klebsiella pneumoniae 1 NA

GCF_001460985 Scaffold Klebsiella pneumoniae KPOI4 Klebsiella pneumoniae 1 Klebsiella pneumoniae 1 Klebsiella pneumoniae 1 NA

GCF_001462525 Contig Klebsiella pneumoniae K48-58 Klebsiella pneumoniae 1 Klebsiella pneumoniae 1 Klebsiella pneumoniae 1 NA

GCF_001462535 Contig Klebsiella pneumoniae K46-62 Klebsiella pneumoniae 1 Klebsiella pneumoniae 1 Klebsiella pneumoniae 1 NA

GCF_001462545 Contig Klebsiella pneumoniae K52-10 Klebsiella pneumoniae 1 Klebsiella pneumoniae 1 Klebsiella pneumoniae 1 NA

GCF_001462555 Contig Klebsiella pneumoniae K47-25 Klebsiella pneumoniae 1 Klebsiella pneumoniae 1 Klebsiella pneumoniae 1 NA

GCF_001462605 Contig Klebsiella pneumoniae K52-36 Klebsiella pneumoniae 1 Klebsiella pneumoniae 1 Klebsiella pneumoniae 1 NA

GCF_001462615 Contig Klebsiella pneumoniae K52-43 Klebsiella pneumoniae 1 Klebsiella pneumoniae 1 Klebsiella pneumoniae 1 NA

GCF_001462645 Contig Klebsiella pneumoniae K53-64 Klebsiella pneumoniae 1 Klebsiella pneumoniae 1 Klebsiella pneumoniae 1 NA

GCF_001462655 Contig Klebsiella pneumoniae K52-74 Klebsiella pneumoniae 1 Klebsiella pneumoniae 1 Klebsiella pneumoniae 1 NA

GCF_001462685 Contig Klebsiella pneumoniae K54-05 Klebsiella pneumoniae 1 Klebsiella pneumoniae 1 Klebsiella pneumoniae 1 NA

GCF_001462705 Contig Klebsiella pneumoniae K57-33 Klebsiella pneumoniae 1 Klebsiella pneumoniae 1 Klebsiella pneumoniae 1 NA

GCF_001462765 Contig Klebsiella pneumoniae K66-62 Klebsiella pneumoniae 1 Klebsiella pneumoniae 1 Klebsiella pneumoniae 1 NA

GCF_001462785 Contig Klebsiella pneumoniae K66-73 Klebsiella pneumoniae 1 Klebsiella pneumoniae 1 Klebsiella pneumoniae 1 NA

GCF_001462795 Contig Klebsiella pneumoniae K67-17 Klebsiella pneumoniae 1 Klebsiella pneumoniae 1 Klebsiella pneumoniae 1 NA

GCF_001462825 Contig Klebsiella pneumoniae K68-18 Klebsiella pneumoniae 1 Klebsiella pneumoniae 1 Klebsiella pneumoniae 1 NA

GCF_001462845 Contig Klebsiella pneumoniae K68-73 Klebsiella pneumoniae 1 Klebsiella pneumoniae 1 Klebsiella pneumoniae 1 NA

GCF_001462865 Contig Klebsiella pneumoniae 50518740 Klebsiella pneumoniae 1 Klebsiella pneumoniae 1 Klebsiella pneumoniae 1 NA

GCF_001462885 Contig Klebsiella pneumoniae 50531633 Klebsiella pneumoniae 1 Klebsiella pneumoniae 1 Klebsiella pneumoniae 1 NA

GCF_001462905 Contig Klebsiella pneumoniae 50572569 Klebsiella pneumoniae 1 Klebsiella pneumoniae 1 Klebsiella pneumoniae 1 NA

GCF_001462965 Contig Klebsiella pneumoniae 50625602 Klebsiella pneumoniae 1 Klebsiella pneumoniae 1 Klebsiella pneumoniae 1 NA

GCF_001463055 Contig Klebsiella pneumoniae 50509588 Klebsiella pneumoniae 1 Klebsiella pneumoniae 1 Klebsiella pneumoniae 1 NA

GCF_001463085 Contig Klebsiella pneumoniae 50580255 Klebsiella pneumoniae 1 Klebsiella pneumoniae 1 Klebsiella pneumoniae 1 NA

GCF_001463105 Contig Klebsiella pneumoniae 50606221 Klebsiella pneumoniae 1 Klebsiella pneumoniae 1 Klebsiella pneumoniae 1 NA

GCF_001463125 Contig Klebsiella pneumoniae 50627996 Klebsiella pneumoniae 1 Klebsiella pneumoniae 1 Klebsiella pneumoniae 1 NA

GCF_001463165 Chromosome Klebsiella pneumoniae U25 Klebsiella pneumoniae 1 Klebsiella pneumoniae 1 Klebsiella pneumoniae 1 NA

GCF_001463185 Contig Klebsiella quasipneumoniae 50667959 Klebsiella quasipneumoniae 2 Klebsiella quasipneumoniae 2 Klebsiella quasipneumoniae NA

GCF_001463215 Contig Klebsiella pneumoniae 50675619 Klebsiella pneumoniae 1 Klebsiella pneumoniae 1 Klebsiella pneumoniae 1 NA

GCF_001463285 Contig Klebsiella pneumoniae 50732159 Klebsiella pneumoniae 1 Klebsiella pneumoniae 1 Klebsiella pneumoniae 1 NA

GCF_001463295 Contig Klebsiella pneumoniae 50700924 Klebsiella pneumoniae 1 Klebsiella pneumoniae 1 Klebsiella pneumoniae 1 NA

GCF_001463415 Contig Klebsiella pneumoniae 50834782 Klebsiella pneumoniae 1 Klebsiella pneumoniae 1 Klebsiella pneumoniae 1 NA

GCF_001463445 Contig Klebsiella pneumoniae 50877064 Klebsiella pneumoniae 1 Klebsiella pneumoniae 1 Klebsiella pneumoniae 1 NA

GCF_001463475 Contig Klebsiella pneumoniae 50884375 Klebsiella pneumoniae 1 Klebsiella pneumoniae 1 Klebsiella pneumoniae 1 NA

GCF_001463525 Contig Klebsiella pneumoniae 50893576 Klebsiella pneumoniae 1 Klebsiella pneumoniae 1 Klebsiella pneumoniae 1 NA

GCF_001463555 Contig Klebsiella pneumoniae 50690310 Klebsiella pneumoniae 1 Klebsiella pneumoniae 1 Klebsiella pneumoniae 1 NA

GCF_001463605 Contig Klebsiella pneumoniae 50806829 Klebsiella pneumoniae 1 Klebsiella pneumoniae 1 Klebsiella pneumoniae 1 NA

GCF_001463615 Contig Klebsiella pneumoniae 50752501 Klebsiella pneumoniae 1 Klebsiella pneumoniae 1 Klebsiella pneumoniae 1 NA

GCF_001463665 Contig Klebsiella pneumoniae 50825040 Klebsiella pneumoniae 1 Klebsiella pneumoniae 1 Klebsiella pneumoniae 1 NA

GCF_001463685 Contig Klebsiella variicola 50878013 Klebsiella variicola Klebsiella variicola Klebsiella variicola NA

GCF_001466765 Contig Klebsiella quasipneumoniae II EMP3 Klebsiella quasipneumoniae 1 Klebsiella quasipneumoniae 1 Klebsiella quasipneumoniae NA

GCF_001471625 Contig Klebsiella pneumoniae FDAARGOS127 Klebsiella pneumoniae 1 Klebsiella pneumoniae 1 Klebsiella pneumoniae 1 NA

GCF_001471835 Contig Klebsiella pneumoniae FDAARGOS91 Klebsiella pneumoniae 1 Klebsiella pneumoniae 1 Klebsiella pneumoniae 1 NA

GCF_001472155 Contig Klebsiella aerogenes SMART350 Klebsiella aerogenes 1 Klebsiella aerogenes Klebsiella aerogenes NA

GCF_001472275 Contig Klebsiella aerogenes SMART429 Klebsiella aerogenes 1 Klebsiella aerogenes Klebsiella aerogenes NA

GCF_001472415 Contig Klebsiella aerogenes SMART543 Klebsiella aerogenes 1 Klebsiella aerogenes Klebsiella aerogenes NA

GCF_001472725 Contig Klebsiella aerogenes SMART773 Klebsiella aerogenes 1 Klebsiella aerogenes Klebsiella aerogenes NA

GCF_001472755 Contig Klebsiella aerogenes SMART774 Klebsiella aerogenes 1 Klebsiella aerogenes Klebsiella aerogenes NA

GCF_001472935 Contig Klebsiella aerogenes SMART888 Klebsiella aerogenes 1 Klebsiella aerogenes Klebsiella aerogenes NA

GCF_001473095 Contig Klebsiella aerogenes SMART1060 Klebsiella aerogenes 1 Klebsiella aerogenes Klebsiella aerogenes NA

GCF_001482345 Complete Klebsiella pneumoniae J1 Klebsiella pneumoniae 1 Klebsiella pneumoniae 1 Klebsiella pneumoniae 1 NA

GCF_001482605 Scaffold Klebsiella pneumoniae CPO2015-0003 Klebsiella pneumoniae 1 Klebsiella pneumoniae 1 Klebsiella pneumoniae 1 NA

GCF_001482645 Scaffold Klebsiella pneumoniae AMA782 Klebsiella pneumoniae 1 Klebsiella pneumoniae 1 Klebsiella pneumoniae 1 NA

GCF_001482995 Contig Klebsiella pneumoniae OC217 Klebsiella pneumoniae 1 Klebsiella pneumoniae 1 Klebsiella pneumoniae 1 NA

GCF_001483005 Contig Klebsiella pneumoniae Z3209 Klebsiella pneumoniae 1 Klebsiella pneumoniae 1 Klebsiella pneumoniae 1 NA

GCF_001483045 Contig Klebsiella pneumoniae OC511 Klebsiella pneumoniae 1 Klebsiella pneumoniae 1 Klebsiella pneumoniae 1 NA

GCF_001483055 Contig Klebsiella pneumoniae K1 Klebsiella pneumoniae 1 Klebsiella pneumoniae 1 Klebsiella pneumoniae 1 NA

GCF_001483105 Contig Klebsiella pneumoniae OC648 Klebsiella pneumoniae 1 Klebsiella pneumoniae 1 Klebsiella pneumoniae 1 NA

GCF_001483725 Contig Klebsiella pneumoniae K45-67 Klebsiella pneumoniae 1 Klebsiella pneumoniae 1 Klebsiella pneumoniae 1 NA

GCF_001510995 Contig Klebsiella pneumoniae 143500514 Klebsiella pneumoniae 1 Klebsiella pneumoniae 1 Klebsiella pneumoniae 1 NA

GCF_001511015 Contig Klebsiella pneumoniae 143500513 Klebsiella pneumoniae 1 Klebsiella pneumoniae 1 Klebsiella pneumoniae 1 NA

GCF_001511035 Scaffold Klebsiella pneumoniae 143500505 Klebsiella pneumoniae 1 Klebsiella pneumoniae 1 Klebsiella pneumoniae 1 NA

GCF_001511055 Scaffold Klebsiella pneumoniae 143500500 Klebsiella pneumoniae 1 Klebsiella pneumoniae 1 Klebsiella pneumoniae 1 NA

GCF_001511095 Scaffold Klebsiella pneumoniae 143500512 Klebsiella pneumoniae 1 Klebsiella pneumoniae 1 Klebsiella pneumoniae 1 NA

GCF_001511115 Scaffold Klebsiella pneumoniae 143500541 Klebsiella pneumoniae 1 Klebsiella pneumoniae 1 Klebsiella pneumoniae 1 NA

GCF_001511135 Scaffold Klebsiella pneumoniae 143500538 Klebsiella pneumoniae 1 Klebsiella pneumoniae 1 Klebsiella pneumoniae 1 NA

GCF_001511155 Scaffold Klebsiella pneumoniae 143500549 Klebsiella pneumoniae 1 Klebsiella pneumoniae 1 Klebsiella pneumoniae 1 NA

GCF_001511175 Scaffold Klebsiella pneumoniae 143500535 Klebsiella pneumoniae 1 Klebsiella pneumoniae 1 Klebsiella pneumoniae 1 NA

GCF_001511195 Scaffold Klebsiella pneumoniae 143500552 Klebsiella pneumoniae 1 Klebsiella pneumoniae 1 Klebsiella pneumoniae 1 NA

GCF_001511215 Scaffold Klebsiella pneumoniae 143500554 Klebsiella pneumoniae 1 Klebsiella pneumoniae 1 Klebsiella pneumoniae 1 NA

GCF_001511255 Contig Klebsiella pneumoniae 143500529 Klebsiella pneumoniae 1 Klebsiella pneumoniae 1 Klebsiella pneumoniae 1 NA

GCF_001511275 Scaffold Klebsiella pneumoniae 143500528 Klebsiella pneumoniae 1 Klebsiella pneumoniae 1 Klebsiella pneumoniae 1 NA

GCF_001511295 Scaffold Klebsiella pneumoniae 143500537 Klebsiella pneumoniae 1 Klebsiella pneumoniae 1 Klebsiella pneumoniae 1 NA

GCF_001511315 Scaffold Klebsiella pneumoniae 143500548 Klebsiella pneumoniae 1 Klebsiella pneumoniae 1 Klebsiella pneumoniae 1 NA

GCF_001511335 Contig Klebsiella pneumoniae 143500531 Klebsiella pneumoniae 1 Klebsiella pneumoniae 1 Klebsiella pneumoniae 1 NA

GCF_001511355 Scaffold Klebsiella pneumoniae 143500506 Klebsiella pneumoniae 1 Klebsiella pneumoniae 1 Klebsiella pneumoniae 1 NA

GCF_001511375 Contig Klebsiella pneumoniae 143500540 Klebsiella pneumoniae 1 Klebsiella pneumoniae 1 Klebsiella pneumoniae 1 NA

GCF_001511395 Scaffold Klebsiella pneumoniae 143500551 Klebsiella pneumoniae 1 Klebsiella pneumoniae 1 Klebsiella pneumoniae 1 NA

GCF_001511415 Scaffold Klebsiella pneumoniae 143500509 Klebsiella pneumoniae 1 Klebsiella pneumoniae 1 Klebsiella pneumoniae 1 NA

GCF_001511435 Scaffold Klebsiella pneumoniae 143500534 Klebsiella pneumoniae 1 Klebsiella pneumoniae 1 Klebsiella pneumoniae 1 NA

GCF_001511455 Scaffold Klebsiella pneumoniae 143500545 Klebsiella pneumoniae 1 Klebsiella pneumoniae 1 Klebsiella pneumoniae 1 NA

GCF_001511475 Scaffold Klebsiella pneumoniae 143500555 Klebsiella pneumoniae 1 Klebsiella pneumoniae 1 Klebsiella pneumoniae 1 NA

GCF_001511495 Scaffold Klebsiella pneumoniae 143500519 Klebsiella pneumoniae 1 Klebsiella pneumoniae 1 Klebsiella pneumoniae 1 NA

GCF_001511535 Scaffold Klebsiella pneumoniae 143500546 Klebsiella pneumoniae 1 Klebsiella pneumoniae 1 Klebsiella pneumoniae 1 NA

GCF_001511555 Scaffold Klebsiella pneumoniae 143500550 Klebsiella pneumoniae 1 Klebsiella pneumoniae 1 Klebsiella pneumoniae 1 NA

GCF_001511575 Scaffold Klebsiella pneumoniae 143500517 Klebsiella pneumoniae 1 Klebsiella pneumoniae 1 Klebsiella pneumoniae 1 NA

GCF_001511595 Scaffold Klebsiella pneumoniae 143500536 Klebsiella pneumoniae 1 Klebsiella pneumoniae 1 Klebsiella pneumoniae 1 NA

GCF_001511635 Scaffold Klebsiella pneumoniae 143500525 Klebsiella pneumoniae 1 Klebsiella pneumoniae 1 Klebsiella pneumoniae 1 NA

GCF_001511675 Scaffold Klebsiella pneumoniae 143500532 Klebsiella pneumoniae 1 Klebsiella pneumoniae 1 Klebsiella pneumoniae 1 NA

GCF_001511695 Scaffold Klebsiella pneumoniae 143500542 Klebsiella pneumoniae 1 Klebsiella pneumoniae 1 Klebsiella pneumoniae 1 NA

GCF_001511715 Contig Klebsiella pneumoniae 143500510 Klebsiella pneumoniae 1 Klebsiella pneumoniae 1 Klebsiella pneumoniae 1 NA

GCF_001511735 Scaffold Klebsiella pneumoniae 143500543 Klebsiella pneumoniae 1 Klebsiella pneumoniae 1 Klebsiella pneumoniae 1 NA

GCF_001517715 Contig Klebsiella pneumoniae M098440 Klebsiella pneumoniae 1 Klebsiella pneumoniae 1 Klebsiella pneumoniae 1 NA

GCF_001517725 Scaffold Klebsiella pneumoniae M098513 Klebsiella pneumoniae 1 Klebsiella pneumoniae 1 Klebsiella pneumoniae 1 NA

GCF_001517735 Contig Klebsiella pneumoniae M097273 Klebsiella pneumoniae 1 Klebsiella pneumoniae 1 Klebsiella pneumoniae 1 NA

GCF_001517745 Scaffold Klebsiella pneumoniae M098451 Klebsiella pneumoniae 1 Klebsiella pneumoniae 1 Klebsiella pneumoniae 1 NA

GCF_001517795 Contig Klebsiella pneumoniae M098442 Klebsiella pneumoniae 1 Klebsiella pneumoniae 1 Klebsiella pneumoniae 1 NA

GCF_001517805 Scaffold Klebsiella pneumoniae M104145 Klebsiella pneumoniae 1 Klebsiella pneumoniae 1 Klebsiella pneumoniae 1 NA

GCF_001517955 Scaffold Klebsiella pneumoniae M098441 Klebsiella pneumoniae 1 Klebsiella pneumoniae 1 Klebsiella pneumoniae 1 NA

GCF_001518035 Contig Klebsiella aerogenes GN02710 Klebsiella aerogenes 1 Klebsiella aerogenes Klebsiella aerogenes NA

GCF_001518115 Contig Klebsiella aerogenes GN03482 Klebsiella aerogenes 1 Klebsiella aerogenes Klebsiella aerogenes NA

GCF_001518125 Contig Klebsiella aerogenes GN05809 Klebsiella aerogenes 1 Klebsiella aerogenes Klebsiella aerogenes NA

GCF_001518435 Contig Klebsiella aerogenes GN05782 Klebsiella aerogenes 2 Klebsiella aerogenes Klebsiella aerogenes NA

GCF_001518675 Contig Klebsiella aerogenes GN05748 Klebsiella aerogenes 1 Klebsiella aerogenes Klebsiella aerogenes NA

GCF_001521895 Complete Klebsiella pneumoniae pneumoniae NUHL24835 Klebsiella pneumoniae 1 Klebsiella pneumoniae 1 Klebsiella pneumoniae 1 NA

GCF_001525245 Contig Klebsiella aerogenes SMART1372 Klebsiella aerogenes 1 Klebsiella aerogenes Klebsiella aerogenes NA

GCF_001525265 Contig Klebsiella aerogenes SMART1249 Klebsiella aerogenes 1 Klebsiella aerogenes Klebsiella aerogenes NA

GCF_001525505 Contig Klebsiella aerogenes SMART1248 Klebsiella aerogenes 1 Klebsiella aerogenes Klebsiella aerogenes NA

GCF_001529935 Complete Klebsiella pneumoniae pneumoniae RJF999 Klebsiella pneumoniae 1 Klebsiella pneumoniae 1 Klebsiella pneumoniae 1 NA

GCF_001530015 Complete Klebsiella pneumoniae pneumoniae RJF293 Klebsiella pneumoniae 1 Klebsiella pneumoniae 1 Klebsiella pneumoniae 1 NA

GCF_001534855 Contig Klebsiella pneumoniae FDAARGOS89 Klebsiella pneumoniae 1 Klebsiella pneumoniae 1 Klebsiella pneumoniae 1 NA

GCF_001546515 Scaffold Klebsiella pneumoniae MJR8396D Klebsiella pneumoniae 1 Klebsiella pneumoniae 1 Klebsiella pneumoniae 1 NA

GCF_001548315 Complete Klebsiella pneumoniae YH43 Klebsiella variicola Klebsiella variicola Klebsiella variicola NA

GCF_001549975 Contig Klebsiella variicola GPKP Klebsiella variicola Klebsiella variicola Klebsiella variicola NA

GCF_001549995 Contig Klebsiella pneumoniae LPKP Klebsiella pneumoniae 1 Klebsiella pneumoniae 1 Klebsiella pneumoniae 1 NA

GCF_001557575 Scaffold Klebsiella pneumoniae CQMUS2 Klebsiella pneumoniae 1 Klebsiella pneumoniae 1 Klebsiella pneumoniae 1 NA

GCF_001559215 Complete Klebsiella aerogenes FDAARGOS152 Klebsiella aerogenes 1 Klebsiella aerogenes Klebsiella aerogenes NA

GCF_001566595 Contig Klebsiella pneumoniae pneumoniae KP48 Klebsiella pneumoniae 1 Klebsiella pneumoniae 1 Klebsiella pneumoniae 1 NA

GCF_001571545 Complete Klebsiella aerogenes G7 Klebsiella aerogenes 1 Klebsiella aerogenes Klebsiella aerogenes NA

GCF_001574505 Scaffold Klebsiella pneumoniae U41 Klebsiella pneumoniae 1 Klebsiella pneumoniae 1 Klebsiella pneumoniae 1 NA

GCF_001583485 Contig Klebsiella michiganensis UCD-JA29 Klebsiella michiganensis 1 Klebsiella michiganensis 1 Klebsiella michiganensis 1 NA

GCF_001593585 Complete Klebsiella aerogenes FDAARGOS139 Klebsiella aerogenes 1 Klebsiella aerogenes Klebsiella aerogenes NA

GCF_001594375 Scaffold Klebsiella oxytoca ICU1-2b Klebsiella oxytoca 1 Klebsiella oxytoca 1 Klebsiella oxytoca 1 NA

GCF_001596075 Complete Klebsiella quasipneumoniae ATCC700603 Klebsiella quasipneumoniae 1 Klebsiella quasipneumoniae 1 Klebsiella quasipneumoniae NA

GCF_001596905 Scaffold Klebsiella pneumoniae KPOII4 Klebsiella pneumoniae 1 Klebsiella pneumoniae 1 Klebsiella pneumoniae 1 NA

GCF_001596915 Scaffold Klebsiella pneumoniae KPOII2 Klebsiella pneumoniae 1 Klebsiella pneumoniae 1 Klebsiella pneumoniae 1 NA

GCF_001596935 Scaffold Klebsiella pneumoniae KPOII1 Klebsiella pneumoniae 1 Klebsiella pneumoniae 1 Klebsiella pneumoniae 1 NA

GCF_001596985 Scaffold Klebsiella pneumoniae KPOII7 Klebsiella pneumoniae 1 Klebsiella pneumoniae 1 Klebsiella pneumoniae 1 NA

GCF_001596995 Contig Klebsiella pneumoniae KPOII10 Klebsiella pneumoniae 1 Klebsiella pneumoniae 1 Klebsiella pneumoniae 1 NA

GCF_001597015 Scaffold Klebsiella pneumoniae KP33P Klebsiella pneumoniae 1 Klebsiella pneumoniae 1 Klebsiella pneumoniae 1 NA

GCF_001597035 Contig Klebsiella pneumoniae KP45D Klebsiella pneumoniae 1 Klebsiella pneumoniae 1 Klebsiella pneumoniae 1 NA

GCF_001597065 Scaffold Klebsiella pneumoniae KP86L Klebsiella pneumoniae 1 Klebsiella pneumoniae 1 Klebsiella pneumoniae 1 NA

GCF_001597075 Scaffold Klebsiella pneumoniae KP33F Klebsiella pneumoniae 1 Klebsiella pneumoniae 1 Klebsiella pneumoniae 1 NA

GCF_001597115 Scaffold Klebsiella pneumoniae KPOII6 Klebsiella pneumoniae 1 Klebsiella pneumoniae 1 Klebsiella pneumoniae 1 NA

GCF_001597145 Scaffold Klebsiella pneumoniae KPOII8 Klebsiella pneumoniae 1 Klebsiella pneumoniae 1 Klebsiella pneumoniae 1 NA

GCF_001597165 Scaffold Klebsiella pneumoniae KPOII9 Klebsiella pneumoniae 1 Klebsiella pneumoniae 1 Klebsiella pneumoniae 1 NA

GCF_001597175 Scaffold Klebsiella pneumoniae KPEII1 Klebsiella pneumoniae 1 Klebsiella pneumoniae 1 Klebsiella pneumoniae 1 NA

GCF_001597195 Contig Klebsiella pneumoniae KPEII2 Klebsiella pneumoniae 1 Klebsiella pneumoniae 1 Klebsiella pneumoniae 1 NA

GCF_001597225 Scaffold Klebsiella pneumoniae KPEII3 Klebsiella pneumoniae 1 Klebsiella pneumoniae 1 Klebsiella pneumoniae 1 NA

GCF_001598695 Contig Klebsiella oxytoca NBRC105695 Klebsiella oxytoca 1 Klebsiella oxytoca 1 Klebsiella oxytoca 1 Type strain

GCF_001598715 Contig Klebsiella pneumoniae ozaenae NBRC105683 Klebsiella pneumoniae 1 Klebsiella pneumoniae 1 Klebsiella pneumoniae 1 Type strain

GCF_001611055 Complete Klebsiella pneumoniae pneumoniae TGH8 Klebsiella pneumoniae 1 Klebsiella pneumoniae 1 Klebsiella pneumoniae 1 NA

GCF_001611095 Complete Klebsiella pneumoniae pneumoniae TGH10 Klebsiella pneumoniae 1 Klebsiella pneumoniae 1 Klebsiella pneumoniae 1 NA

GCF_001611185 Contig Klebsiella quasipneumoniae similipneumoniae KP-Z4175 Klebsiella quasipneumoniae 1 Klebsiella quasipneumoniae 1 Klebsiella quasipneumoniae NA

GCF_001613565 Scaffold Klebsiella pneumoniae TR191 Klebsiella pneumoniae 1 Klebsiella pneumoniae 1 Klebsiella pneumoniae 1 NA

GCF_001613575 Scaffold Klebsiella pneumoniae TRqt-41 Klebsiella pneumoniae 1 Klebsiella pneumoniae 1 Klebsiella pneumoniae 1 NA

GCF_001613585 Scaffold Klebsiella pneumoniae TRqt-37 Klebsiella pneumoniae 1 Klebsiella pneumoniae 1 Klebsiella pneumoniae 1 NA

GCF_001613595 Scaffold Klebsiella pneumoniae TR198 Klebsiella pneumoniae 1 Klebsiella pneumoniae 1 Klebsiella pneumoniae 1 NA

GCF_001613645 Scaffold Klebsiella pneumoniae TRqt-49 Klebsiella pneumoniae 1 Klebsiella pneumoniae 1 Klebsiella pneumoniae 1 NA

GCF_001613655 Scaffold Klebsiella pneumoniae TR200 Klebsiella pneumoniae 1 Klebsiella pneumoniae 1 Klebsiella pneumoniae 1 NA

GCF_001613665 Scaffold Klebsiella pneumoniae TR262 Klebsiella pneumoniae 1 Klebsiella pneumoniae 1 Klebsiella pneumoniae 1 NA

GCF_001613715 Scaffold Klebsiella pneumoniae TR187 Klebsiella pneumoniae 1 Klebsiella pneumoniae 1 Klebsiella pneumoniae 1 NA

GCF_001617965 Scaffold Klebsiella variicola KV321 Klebsiella variicola Klebsiella variicola Klebsiella variicola NA

GCF_001620885 Scaffold Klebsiella sp D5A Klebsiella variicola Klebsiella variicola Klebsiella variicola NA

GCF_001630805 Scaffold Klebsiella pneumoniae SCLZ15-011 Klebsiella pneumoniae 1 Klebsiella pneumoniae 1 Klebsiella pneumoniae 1 NA

GCF_001631185 Contig Klebsiella aerogenes GN05695 Klebsiella aerogenes 1 Klebsiella aerogenes Klebsiella aerogenes NA

GCF_001631215 Contig Klebsiella aerogenes GN05905 Klebsiella aerogenes 1 Klebsiella aerogenes Klebsiella aerogenes NA

GCF_001631305 Contig Klebsiella aerogenes GN05439 Klebsiella aerogenes 1 Klebsiella aerogenes Klebsiella aerogenes NA

GCF_001631315 Contig Klebsiella aerogenes GN05979 Klebsiella aerogenes 1 Klebsiella aerogenes Klebsiella aerogenes NA

GCF_001631345 Contig Klebsiella aerogenes GN05352 Klebsiella aerogenes 1 Klebsiella aerogenes Klebsiella aerogenes NA

GCF_001631565 Contig Klebsiella aerogenes GN05662 Klebsiella aerogenes 1 Klebsiella aerogenes Klebsiella aerogenes NA

GCF_001631605 Contig Klebsiella aerogenes GN06193 Klebsiella aerogenes 2 Klebsiella aerogenes Klebsiella aerogenes NA

GCF_001631645 Contig Klebsiella aerogenes GN05388 Klebsiella aerogenes 1 Klebsiella aerogenes Klebsiella aerogenes NA

GCF_001631655 Contig Klebsiella aerogenes GN05857 Klebsiella aerogenes 1 Klebsiella aerogenes Klebsiella aerogenes NA

GCF_001631725 Contig Klebsiella aerogenes GN06347 Klebsiella aerogenes 1 Klebsiella aerogenes Klebsiella aerogenes NA

GCF_001631735 Contig Klebsiella aerogenes GN06516 Klebsiella aerogenes 1 Klebsiella aerogenes Klebsiella aerogenes NA

GCF_001631755 Contig Klebsiella aerogenes GN05939 Klebsiella aerogenes 1 Klebsiella aerogenes Klebsiella aerogenes NA

GCF_001631815 Contig Klebsiella aerogenes GN04222 Klebsiella aerogenes 1 Klebsiella aerogenes Klebsiella aerogenes NA

GCF_001631845 Contig Klebsiella aerogenes GN06255 Klebsiella aerogenes 2 Klebsiella aerogenes Klebsiella aerogenes NA

GCF_001631895 Contig Klebsiella aerogenes GN06322 Klebsiella aerogenes 1 Klebsiella aerogenes Klebsiella aerogenes NA

GCF_001633115 Contig Klebsiella michiganensis M5al Klebsiella grimontii Klebsiella grimontii Klebsiella grimontii NA

GCF_001640185 Contig Klebsiella pneumoniae EA Carb Klebsiella pneumoniae 1 Klebsiella pneumoniae 1 Klebsiella pneumoniae 1 NA

GCF_001644765 Complete Klebsiella pneumoniae SKGH01 Klebsiella pneumoniae 1 Klebsiella pneumoniae 1 Klebsiella pneumoniae 1 NA

GCF_001645745 Contig Klebsiella pneumoniae pneumoniae KPC45 Klebsiella pneumoniae 1 Klebsiella pneumoniae 1 Klebsiella pneumoniae 1 NA

GCF_001646625 Complete Klebsiella pneumoniae W14 Klebsiella pneumoniae 1 Klebsiella pneumoniae 1 Klebsiella pneumoniae 1 NA

GCF_001647455 Scaffold Klebsiella pneumoniae 101436 Klebsiella pneumoniae 1 Klebsiella pneumoniae 1 Klebsiella pneumoniae 1 NA

GCF_001647465 Scaffold Klebsiella pneumoniae 105371 Klebsiella pneumoniae 1 Klebsiella pneumoniae 1 Klebsiella pneumoniae 1 NA

GCF_001647475 Scaffold Klebsiella pneumoniae 101731 Klebsiella pneumoniae 1 Klebsiella pneumoniae 1 Klebsiella pneumoniae 1 NA

GCF_001648215 Complete Klebsiella pneumoniae AATZP Klebsiella pneumoniae 1 Klebsiella pneumoniae 1 Klebsiella pneumoniae 1 NA

GCF_001649605 Scaffold Klebsiella aerogenes Ea77 Klebsiella aerogenes 1 Klebsiella aerogenes Klebsiella aerogenes NA

GCF_001653695 Scaffold Klebsiella pneumoniae INSali390 Klebsiella pneumoniae 1 Klebsiella pneumoniae 1 Klebsiella pneumoniae 1 NA

GCF_001661895 Contig Klebsiella pneumoniae AS Klebsiella pneumoniae 1 Klebsiella pneumoniae 1 Klebsiella pneumoniae 1 NA

GCF_001662695 Scaffold Klebsiella aerogenes D3 Klebsiella aerogenes 1 Klebsiella aerogenes Klebsiella aerogenes NA

GCF_001662705 Scaffold Klebsiella aerogenes D2 Klebsiella aerogenes 1 Klebsiella aerogenes Klebsiella aerogenes NA

GCF_001662715 Scaffold Klebsiella aerogenes E9 Klebsiella aerogenes 1 Klebsiella aerogenes Klebsiella aerogenes NA

GCF_001662765 Scaffold Klebsiella aerogenes C10 Klebsiella aerogenes 1 Klebsiella aerogenes Klebsiella aerogenes NA

GCF_001663195 Complete Klebsiella pneumoniae BR Klebsiella pneumoniae 1 Klebsiella pneumoniae 1 Klebsiella pneumoniae 1 NA

GCF_001663295 Complete Klebsiella pneumoniae KPNIH39 Klebsiella pneumoniae 1 Klebsiella pneumoniae 1 Klebsiella pneumoniae 1 NA

GCF_001663435 Complete Klebsiella pneumoniae Kpn223 Klebsiella pneumoniae 1 Klebsiella pneumoniae 1 Klebsiella pneumoniae 1 NA

GCF_001663455 Complete Klebsiella pneumoniae Kpn555 Klebsiella pneumoniae 1 Klebsiella pneumoniae 1 Klebsiella pneumoniae 1 NA

GCF_001663535 Contig Klebsiella quasipneumoniae similipneumoniae MB373 Klebsiella quasipneumoniae 1 Klebsiella quasipneumoniae 1 Klebsiella quasipneumoniae NA

GCF_001665995 Contig Klebsiella pneumoniae KPNIH38 Klebsiella pneumoniae 1 Klebsiella pneumoniae 1 Klebsiella pneumoniae 1 NA

GCF_001666005 Contig Klebsiella pneumoniae KPNIH40 Klebsiella pneumoniae 1 Klebsiella pneumoniae 1 Klebsiella pneumoniae 1 NA

GCF_001666015 Contig Klebsiella pneumoniae KPNIH35 Klebsiella pneumoniae 1 Klebsiella pneumoniae 1 Klebsiella pneumoniae 1 NA

GCF_001667545 Contig Klebsiella pneumoniae KPNIH37 Klebsiella pneumoniae 1 Klebsiella pneumoniae 1 Klebsiella pneumoniae 1 NA

GCF_001675125 Complete Klebsiella pneumoniae KPNIH36 Klebsiella pneumoniae 1 Klebsiella pneumoniae 1 Klebsiella pneumoniae 1 NA

GCF_001676825 Complete Klebsiella pneumoniae TH1 Klebsiella pneumoniae 1 Klebsiella pneumoniae 1 Klebsiella pneumoniae 1 NA

GCF_001678695 Contig Klebsiella pneumoniae Kp08 Klebsiella pneumoniae 1 Klebsiella pneumoniae 1 Klebsiella pneumoniae 1 NA

GCF_001679915 Scaffold Klebsiella pneumoniae KP6884 Klebsiella pneumoniae 1 Klebsiella pneumoniae 1 Klebsiella pneumoniae 1 NA

GCF_001693715 Contig Klebsiella pneumoniae Kp33 Klebsiella pneumoniae 1 Klebsiella pneumoniae 1 Klebsiella pneumoniae 1 NA

GCF_001695655 Contig Klebsiella pneumoniae KPNIH34 Klebsiella pneumoniae 1 Klebsiella pneumoniae 1 Klebsiella pneumoniae 1 NA

GCF_001695775 Scaffold Klebsiella pneumoniae MB361 Klebsiella pneumoniae 1 Klebsiella pneumoniae 1 Klebsiella pneumoniae 1 NA

GCF_001698945 Contig Klebsiella pneumoniae XH212 Klebsiella pneumoniae 1 Klebsiella pneumoniae 1 Klebsiella pneumoniae 1 NA

GCF_001699015 Contig Klebsiella pneumoniae XH211 Klebsiella pneumoniae 1 Klebsiella pneumoniae 1 Klebsiella pneumoniae 1 NA

GCF_001699025 Contig Klebsiella pneumoniae XH213 Klebsiella pneumoniae 1 Klebsiella pneumoniae 1 Klebsiella pneumoniae 1 NA

GCF_001699035 Contig Klebsiella pneumoniae XH214 Klebsiella pneumoniae 1 Klebsiella pneumoniae 1 Klebsiella pneumoniae 1 NA

GCF_001699045 Contig Klebsiella pneumoniae XH215 Klebsiella pneumoniae 1 Klebsiella pneumoniae 1 Klebsiella pneumoniae 1 NA

GCF_001699095 Contig Klebsiella pneumoniae XH216 Klebsiella pneumoniae 1 Klebsiella pneumoniae 1 Klebsiella pneumoniae 1 NA

GCF_001699105 Complete Klebsiella pneumoniae XH210 Klebsiella pneumoniae 1 Klebsiella pneumoniae 1 Klebsiella pneumoniae 1 NA

GCF_001701355 Contig Klebsiella pneumoniae 19 GR14 Klebsiella pneumoniae 1 Klebsiella pneumoniae 1 Klebsiella pneumoniae 1 NA

GCF_001701365 Contig Klebsiella pneumoniae 13 GR14 Klebsiella pneumoniae 1 Klebsiella pneumoniae 1 Klebsiella pneumoniae 1 NA

GCF_001701395 Contig Klebsiella pneumoniae 22 GR12 Klebsiella pneumoniae 1 Klebsiella pneumoniae 1 Klebsiella pneumoniae 1 NA

GCF_001701415 Contig Klebsiella pneumoniae 24 GR13 Klebsiella pneumoniae 1 Klebsiella pneumoniae 1 Klebsiella pneumoniae 1 NA

GCF_001701425 Complete Klebsiella pneumoniae 1 GR13 Klebsiella pneumoniae 1 Klebsiella pneumoniae 1 Klebsiella pneumoniae 1 NA

GCF_001701445 Contig Klebsiella pneumoniae 7 GR13 Klebsiella pneumoniae 1 Klebsiella pneumoniae 1 Klebsiella pneumoniae 1 NA

GCF_001701475 Contig Klebsiella pneumoniae 15 GR13 Klebsiella pneumoniae 1 Klebsiella pneumoniae 1 Klebsiella pneumoniae 1 NA

GCF_001701495 Contig Klebsiella pneumoniae 17 GR14 Klebsiella pneumoniae 1 Klebsiella pneumoniae 1 Klebsiella pneumoniae 1 NA

GCF_001701505 Contig Klebsiella pneumoniae 8 GR13 Klebsiella pneumoniae 1 Klebsiella pneumoniae 1 Klebsiella pneumoniae 1 NA

GCF_001701525 Contig Klebsiella pneumoniae 18 GR14 Klebsiella pneumoniae 1 Klebsiella pneumoniae 1 Klebsiella pneumoniae 1 NA

GCF_001701555 Contig Klebsiella pneumoniae 23 GR12 Klebsiella pneumoniae 1 Klebsiella pneumoniae 1 Klebsiella pneumoniae 1 NA

GCF_001701575 Scaffold Klebsiella pneumoniae SKLX2722 Klebsiella pneumoniae 1 Klebsiella pneumoniae 1 Klebsiella pneumoniae 1 NA

GCF_001701585 Scaffold Klebsiella pneumoniae SKLX2467 Klebsiella pneumoniae 1 Klebsiella pneumoniae 1 Klebsiella pneumoniae 1 NA

GCF_001701615 Scaffold Klebsiella pneumoniae SKLX2821 Klebsiella pneumoniae 1 Klebsiella pneumoniae 1 Klebsiella pneumoniae 1 NA

GCF_001701625 Scaffold Klebsiella pneumoniae SKLX2836 Klebsiella pneumoniae 1 Klebsiella pneumoniae 1 Klebsiella pneumoniae 1 NA

GCF_001701635 Scaffold Klebsiella quasipneumoniae similipneumoniae SKLX2736 Klebsiella quasipneumoniae 1 Klebsiella quasipneumoniae 1 Klebsiella quasipneumoniae NA

GCF_001701645 Scaffold Klebsiella quasipneumoniae similipneumoniae SKLX2781 Klebsiella quasipneumoniae 1 Klebsiella quasipneumoniae 1 Klebsiella quasipneumoniae NA

GCF_001701695 Scaffold Klebsiella pneumoniae SKLX2845 Klebsiella pneumoniae 1 Klebsiella pneumoniae 1 Klebsiella pneumoniae 1 NA

GCF_001701705 Scaffold Klebsiella pneumoniae SKLX2900 Klebsiella pneumoniae 1 Klebsiella pneumoniae 1 Klebsiella pneumoniae 1 NA

GCF_001701725 Scaffold Klebsiella pneumoniae SKLX2993 Klebsiella pneumoniae 1 Klebsiella pneumoniae 1 Klebsiella pneumoniae 1 NA

GCF_001701755 Scaffold Klebsiella pneumoniae SKLX4211 Klebsiella pneumoniae 1 Klebsiella pneumoniae 1 Klebsiella pneumoniae 1 NA

GCF_001701765 Scaffold Klebsiella pneumoniae SKLX2891 Klebsiella pneumoniae 1 Klebsiella pneumoniae 1 Klebsiella pneumoniae 1 NA

GCF_001701795 Scaffold Klebsiella pneumoniae SKLX2848 Klebsiella pneumoniae 1 Klebsiella pneumoniae 1 Klebsiella pneumoniae 1 NA

GCF_001701815 Contig Klebsiella pneumoniae 14 GR14 Klebsiella pneumoniae 1 Klebsiella pneumoniae 1 Klebsiella pneumoniae 1 NA

GCF_001701835 Complete Klebsiella pneumoniae 16 GR13 Klebsiella pneumoniae 1 Klebsiella pneumoniae 1 Klebsiella pneumoniae 1 NA

GCF_001701845 Complete Klebsiella pneumoniae 20 GR12 Klebsiella pneumoniae 1 Klebsiella pneumoniae 1 Klebsiella pneumoniae 1 NA

GCF_001701875 Contig Klebsiella quasipneumoniae 21 GR13 Klebsiella quasipneumoniae 2 Klebsiella quasipneumoniae 2 Klebsiella quasipneumoniae NA

GCF_001701895 Contig Klebsiella pneumoniae 12 BR13 Klebsiella pneumoniae 1 Klebsiella pneumoniae 1 Klebsiella pneumoniae 1 NA

GCF_001701915 Contig Klebsiella pneumoniae 11 BR13 Klebsiella pneumoniae 1 Klebsiella pneumoniae 1 Klebsiella pneumoniae 1 NA

GCF_001701925 Contig Klebsiella pneumoniae 9 GR12 Klebsiella pneumoniae 1 Klebsiella pneumoniae 1 Klebsiella pneumoniae 1 NA

GCF_001701935 Contig Klebsiella pneumoniae 6 GR12 Klebsiella pneumoniae 1 Klebsiella pneumoniae 1 Klebsiella pneumoniae 1 NA

GCF_001701975 Contig Klebsiella pneumoniae 10 GR13 Klebsiella pneumoniae 1 Klebsiella pneumoniae 1 Klebsiella pneumoniae 1 NA

GCF_001701985 Contig Klebsiella pneumoniae 5 GR13 Klebsiella pneumoniae 1 Klebsiella pneumoniae 1 Klebsiella pneumoniae 1 NA

GCF_001702005 Contig Klebsiella pneumoniae 3 GR13 Klebsiella pneumoniae 1 Klebsiella pneumoniae 1 Klebsiella pneumoniae 1 NA

GCF_001702035 Contig Klebsiella pneumoniae 4 GR12 Klebsiella pneumoniae 1 Klebsiella pneumoniae 1 Klebsiella pneumoniae 1 NA

GCF_001702045 Complete Klebsiella pneumoniae 2 GR12 Klebsiella pneumoniae 1 Klebsiella pneumoniae 1 Klebsiella pneumoniae 1 NA

GCF_001704235 Complete Klebsiella pneumoniae DHQP1002001 Klebsiella pneumoniae 1 Klebsiella pneumoniae 1 Klebsiella pneumoniae 1 NA

GCF_001705385 Complete Klebsiella pneumoniae blood sample 2 Klebsiella pneumoniae 1 Klebsiella pneumoniae 1 Klebsiella pneumoniae 1 NA

GCF_001707135 Scaffold Klebsiella pneumoniae UTSW Atlanta 01 Klebsiella pneumoniae 1 Klebsiella pneumoniae 1 Klebsiella pneumoniae 1 NA

GCF_001708165 Contig Klebsiella pneumoniae pneumoniae KPACU35 Klebsiella pneumoniae 1 Klebsiella pneumoniae 1 Klebsiella pneumoniae 1 NA

GCF_001708225 Complete Klebsiella pneumoniae ED23 Klebsiella pneumoniae 1 Klebsiella pneumoniae 1 Klebsiella pneumoniae 1 NA

GCF_001708245 Complete Klebsiella pneumoniae ED2 Klebsiella pneumoniae 1 Klebsiella pneumoniae 1 Klebsiella pneumoniae 1 NA

GCF_001709275 Complete Klebsiella pneumoniae 11 Klebsiella pneumoniae 1 Klebsiella pneumoniae 1 Klebsiella pneumoniae 1 NA

GCF_001709295 Complete Klebsiella pneumoniae 23 Klebsiella pneumoniae 1 Klebsiella pneumoniae 1 Klebsiella pneumoniae 1 NA

GCF_001715215 Contig Klebsiella pneumoniae CCBH17440 Klebsiella pneumoniae 1 Klebsiella pneumoniae 1 Klebsiella pneumoniae 1 NA

GCF_001715345 Contig Klebsiella quasipneumoniae CCBH16302 Klebsiella quasipneumoniae 1 Klebsiella quasipneumoniae 1 Klebsiella quasipneumoniae NA

GCF_001717795 Contig Klebsiella pneumoniae S1 Klebsiella pneumoniae 1 Klebsiella pneumoniae 1 Klebsiella pneumoniae 1 NA

GCF_001718115 Scaffold Klebsiella pneumoniae 1194 Klebsiella pneumoniae 1 Klebsiella pneumoniae 1 Klebsiella pneumoniae 1 NA

GCF_001718175 Scaffold Klebsiella pneumoniae 606B Klebsiella pneumoniae 1 Klebsiella pneumoniae 1 Klebsiella pneumoniae 1 NA

GCF_001720345 Contig Klebsiella pneumoniae CCBH6984 Klebsiella pneumoniae 1 Klebsiella pneumoniae 1 Klebsiella pneumoniae 1 NA

GCF_001720625 Contig Klebsiella pneumoniae SCPM-O-B7846 Klebsiella pneumoniae 1 Klebsiella pneumoniae 1 Klebsiella pneumoniae 1 NA

GCF_001720635 Contig Klebsiella pneumoniae SCPM-O-B7850 Klebsiella pneumoniae 1 Klebsiella pneumoniae 1 Klebsiella pneumoniae 1 NA

GCF_001720645 Contig Klebsiella pneumoniae SCPM-O-B7749 Klebsiella pneumoniae 1 Klebsiella pneumoniae 1 Klebsiella pneumoniae 1 NA

GCF_001720695 Contig Klebsiella pneumoniae CFSAN044574 Klebsiella pneumoniae 1 Klebsiella pneumoniae 1 Klebsiella pneumoniae 1 NA

GCF_001720705 Contig Klebsiella pneumoniae CFSAN044569 Klebsiella pneumoniae 1 Klebsiella pneumoniae 1 Klebsiella pneumoniae 1 NA

GCF_001720745 Contig Klebsiella pneumoniae CFSAN044573 Klebsiella pneumoniae 1 Klebsiella pneumoniae 1 Klebsiella pneumoniae 1 NA

GCF_001720755 Contig Klebsiella pneumoniae CFSAN044570 Klebsiella pneumoniae 1 Klebsiella pneumoniae 1 Klebsiella pneumoniae 1 NA

GCF_001720765 Contig Klebsiella pneumoniae CFSAN044571 Klebsiella pneumoniae 1 Klebsiella pneumoniae 1 Klebsiella pneumoniae 1 NA

GCF_001720805 Contig Klebsiella pneumoniae CFSAN044568 Klebsiella pneumoniae 1 Klebsiella pneumoniae 1 Klebsiella pneumoniae 1 NA

GCF_001720815 Contig Klebsiella pneumoniae CFSAN044572 Klebsiella pneumoniae 1 Klebsiella pneumoniae 1 Klebsiella pneumoniae 1 NA

GCF_001720845 Contig Klebsiella pneumoniae CFSAN044564 Klebsiella pneumoniae 1 Klebsiella pneumoniae 1 Klebsiella pneumoniae 1 NA

GCF_001720865 Contig Klebsiella pneumoniae CFSAN044563 Klebsiella pneumoniae 1 Klebsiella pneumoniae 1 Klebsiella pneumoniae 1 NA

GCF_001720875 Contig Klebsiella pneumoniae CFSAN044566 Klebsiella pneumoniae 1 Klebsiella pneumoniae 1 Klebsiella pneumoniae 1 NA

GCF_001720905 Contig Klebsiella pneumoniae CFSAN044565 Klebsiella pneumoniae 1 Klebsiella pneumoniae 1 Klebsiella pneumoniae 1 NA

GCF_001721725 Complete Klebsiella pneumoniae UCLAOXA232KP Pt0 Klebsiella pneumoniae 1 Klebsiella pneumoniae 1 Klebsiella pneumoniae 1 NA

GCF_001729665 Scaffold Klebsiella quasipneumoniae B8095 Klebsiella quasipneumoniae 1 Klebsiella quasipneumoniae 1 Klebsiella quasipneumoniae NA

GCF_001741545 Complete Klebsiella pneumoniae UCLAOXA232KP Klebsiella pneumoniae 1 Klebsiella pneumoniae 1 Klebsiella pneumoniae 1 NA

GCF_001746535 Complete Klebsiella pneumoniae pneumoniae TGH13 Klebsiella pneumoniae 1 Klebsiella pneumoniae 1 Klebsiella pneumoniae 1 NA

GCF_001746765 Contig Klebsiella pneumoniae KP171 Klebsiella pneumoniae 1 Klebsiella pneumoniae 1 Klebsiella pneumoniae 1 NA

GCF_001753185 Complete Klebsiella sp LTGPAF6F Klebsiella michiganensis 1 Klebsiella michiganensis 1 Klebsiella michiganensis 1 NA

GCF_001756835 Scaffold Klebsiella pneumoniae 145 Klebsiella pneumoniae 1 Klebsiella pneumoniae 1 Klebsiella pneumoniae 1 NA

GCF_001807645 Scaffold Klebsiella sp HMSC22F09 Klebsiella variicola Klebsiella variicola Klebsiella variicola NA

GCF_001807705 Scaffold Klebsiella sp HMSC25G12 Klebsiella variicola Klebsiella variicola Klebsiella variicola NA

GCF_001808265 Scaffold Klebsiella sp HMSC16A12 Klebsiella pneumoniae 1 Klebsiella pneumoniae 1 Klebsiella pneumoniae 1 NA

GCF_001808325 Scaffold Klebsiella sp HMSC16C06 Klebsiella variicola Klebsiella variicola Klebsiella variicola NA

GCF_001808475 Scaffold Klebsiella sp HMSC09D12 Klebsiella oxytoca 1 Klebsiella oxytoca 1 Klebsiella oxytoca 1 NA

GCF_001855315 Complete Klebsiella pneumoniae KP5 Klebsiella pneumoniae 1 Klebsiella pneumoniae 1 Klebsiella pneumoniae 1 NA

GCF_001856585 Scaffold Klebsiella pneumoniae kp10 Klebsiella pneumoniae 1 Klebsiella pneumoniae 1 Klebsiella pneumoniae 1 NA

GCF_001866785 Scaffold Klebsiella pneumoniae KP41 Klebsiella pneumoniae 1 Klebsiella pneumoniae 1 Klebsiella pneumoniae 1 NA

GCF_001867195 Contig Klebsiella pneumoniae WCHKP1511 Klebsiella pneumoniae 1 Klebsiella pneumoniae 1 Klebsiella pneumoniae 1 NA

GCF_001870165 Complete Klebsiella pneumoniae CAV1016 Klebsiella pneumoniae 1 Klebsiella pneumoniae 1 Klebsiella pneumoniae 1 NA

GCF_001870185 Complete Klebsiella oxytoca CAV1015 Klebsiella oxytoca 1 Klebsiella oxytoca 1 Klebsiella oxytoca 1 NA

GCF_001874695 Contig Klebsiella pneumoniae AR0042 Klebsiella pneumoniae 1 Klebsiella pneumoniae 1 Klebsiella pneumoniae 1 NA

GCF_001874715 Contig Klebsiella pneumoniae AR0043 Klebsiella pneumoniae 1 Klebsiella pneumoniae 1 Klebsiella pneumoniae 1 NA

GCF_001874725 Contig Klebsiella pneumoniae AR0012 Klebsiella pneumoniae 1 Klebsiella pneumoniae 1 Klebsiella pneumoniae 1 NA

GCF_001874865 Contig Klebsiella pneumoniae AR0010 Klebsiella pneumoniae 1 Klebsiella pneumoniae 1 Klebsiella pneumoniae 1 NA

GCF_001874875 Contig Klebsiella pneumoniae AR0039 Klebsiella pneumoniae 1 Klebsiella pneumoniae 1 Klebsiella pneumoniae 1 NA

GCF_001875025 Contig Klebsiella pneumoniae AR0003 Klebsiella pneumoniae 1 Klebsiella pneumoniae 1 Klebsiella pneumoniae 1 NA

GCF_001875045 Contig Klebsiella pneumoniae AR0004 Klebsiella pneumoniae 1 Klebsiella pneumoniae 1 Klebsiella pneumoniae 1 NA

GCF_001875055 Contig Klebsiella pneumoniae AR0005 Klebsiella pneumoniae 1 Klebsiella pneumoniae 1 Klebsiella pneumoniae 1 NA

GCF_001875075 Contig Klebsiella pneumoniae AR0034 Klebsiella pneumoniae 1 Klebsiella pneumoniae 1 Klebsiella pneumoniae 1 NA

GCF_001875105 Contig Klebsiella pneumoniae AR0040 Klebsiella pneumoniae 1 Klebsiella pneumoniae 1 Klebsiella pneumoniae 1 NA

GCF_001875125 Contig Klebsiella pneumoniae AR0041 Klebsiella pneumoniae 1 Klebsiella pneumoniae 1 Klebsiella pneumoniae 1 NA

GCF_001875135 Contig Klebsiella pneumoniae AR0044 Klebsiella pneumoniae 1 Klebsiella pneumoniae 1 Klebsiella pneumoniae 1 NA

GCF_001875745 Contig Klebsiella variicola AR0016 Klebsiella variicola Klebsiella variicola Klebsiella variicola NA

GCF_001876675 Contig Klebsiella pneumoniae AWD5 Klebsiella pneumoniae 1 Klebsiella pneumoniae 1 Klebsiella pneumoniae 1 NA

GCF_001884315 Contig Klebsiella pneumoniae KpN03 Klebsiella pneumoniae 1 Klebsiella pneumoniae 1 Klebsiella pneumoniae 1 NA

GCF_001884325 Contig Klebsiella pneumoniae KpN07 Klebsiella pneumoniae 1 Klebsiella pneumoniae 1 Klebsiella pneumoniae 1 NA

GCF_001884335 Contig Klebsiella pneumoniae KpN02 Klebsiella pneumoniae 1 Klebsiella pneumoniae 1 Klebsiella pneumoniae 1 NA

GCF_001884385 Contig Klebsiella pneumoniae KpN08 Klebsiella pneumoniae 1 Klebsiella pneumoniae 1 Klebsiella pneumoniae 1 NA

GCF_001884395 Contig Klebsiella pneumoniae KpN04 Klebsiella pneumoniae 1 Klebsiella pneumoniae 1 Klebsiella pneumoniae 1 NA

GCF_001884415 Contig Klebsiella pneumoniae KpN05 Klebsiella pneumoniae 1 Klebsiella pneumoniae 1 Klebsiella pneumoniae 1 NA

GCF_001884425 Contig Klebsiella pneumoniae KpN10 Klebsiella pneumoniae 1 Klebsiella pneumoniae 1 Klebsiella pneumoniae 1 NA

GCF_001884465 Contig Klebsiella pneumoniae KpN09 Klebsiella pneumoniae 1 Klebsiella pneumoniae 1 Klebsiella pneumoniae 1 NA

GCF_001884485 Contig Klebsiella pneumoniae KpN11 Klebsiella pneumoniae 1 Klebsiella pneumoniae 1 Klebsiella pneumoniae 1 NA

GCF_001884765 Contig Klebsiella pneumoniae KP04C62 Klebsiella pneumoniae 1 Klebsiella pneumoniae 1 Klebsiella pneumoniae 1 NA

GCF_001885255 Complete Klebsiella pneumoniae Kp Goe 822579 Klebsiella pneumoniae 1 Klebsiella pneumoniae 1 Klebsiella pneumoniae 1 NA

GCF_001887985 Complete Klebsiella pneumoniae WCHKP095649 Klebsiella pneumoniae 1 Klebsiella pneumoniae 1 Klebsiella pneumoniae 1 NA

GCF_001887995 Complete Klebsiella pneumoniae WCHKP095845 Klebsiella pneumoniae 1 Klebsiella pneumoniae 1 Klebsiella pneumoniae 1 NA

GCF_001901855 Contig Klebsiella pneumoniae YMC2011/11/B7578 Klebsiella pneumoniae 1 Klebsiella pneumoniae 1 Klebsiella pneumoniae 1 NA

GCF_001902195 Complete Klebsiella pneumoniae MNCRE78 Klebsiella pneumoniae 1 Klebsiella pneumoniae 1 Klebsiella pneumoniae 1 NA

GCF_001902215 Complete Klebsiella pneumoniae MNCRE69 Klebsiella pneumoniae 1 Klebsiella pneumoniae 1 Klebsiella pneumoniae 1 NA

GCF_001902235 Complete Klebsiella pneumoniae MNCRE53 Klebsiella pneumoniae 1 Klebsiella pneumoniae 1 Klebsiella pneumoniae 1 NA

GCF_001902255 Complete Klebsiella pneumoniae CAV1453 Klebsiella pneumoniae 1 Klebsiella pneumoniae 1 Klebsiella pneumoniae 1 NA

GCF_001902335 Complete Klebsiella pneumoniae Kp Goe 154414 Klebsiella pneumoniae 1 Klebsiella pneumoniae 1 Klebsiella pneumoniae 1 NA

GCF_001902355 Complete Klebsiella pneumoniae Kp Goe 62629 Klebsiella pneumoniae 1 Klebsiella pneumoniae 1 Klebsiella pneumoniae 1 NA

GCF_001902415 Complete Klebsiella pneumoniae Kp Goe 822917 Klebsiella pneumoniae 1 Klebsiella pneumoniae 1 Klebsiella pneumoniae 1 NA

GCF_001902435 Complete Klebsiella pneumoniae Kp Goe 33208 Klebsiella pneumoniae 1 Klebsiella pneumoniae 1 Klebsiella pneumoniae 1 NA

GCF_001902475 Complete Klebsiella pneumoniae SWU01 Klebsiella pneumoniae 1 Klebsiella pneumoniae 1 Klebsiella pneumoniae 1 NA

GCF_001902515 Complete Klebsiella pneumoniae Kp Goe 71070 Klebsiella pneumoniae 1 Klebsiella pneumoniae 1 Klebsiella pneumoniae 1 NA

GCF_001902535 Complete Klebsiella pneumoniae CAV1417 Klebsiella pneumoniae 1 Klebsiella pneumoniae 1 Klebsiella pneumoniae 1 NA

GCF_001902755 Contig Klebsiella pneumoniae CRK0150 Klebsiella pneumoniae 1 Klebsiella pneumoniae 1 Klebsiella pneumoniae 1 NA

GCF_001902765 Contig Klebsiella pneumoniae CRK0161 Klebsiella pneumoniae 1 Klebsiella pneumoniae 1 Klebsiella pneumoniae 1 NA

GCF_001902775 Contig Klebsiella pneumoniae CRK0159 Klebsiella pneumoniae 1 Klebsiella pneumoniae 1 Klebsiella pneumoniae 1 NA

GCF_001902815 Contig Klebsiella pneumoniae CRK0158 Klebsiella pneumoniae 1 Klebsiella pneumoniae 1 Klebsiella pneumoniae 1 NA

GCF_001902835 Contig Klebsiella pneumoniae CRK0130 Klebsiella pneumoniae 1 Klebsiella pneumoniae 1 Klebsiella pneumoniae 1 NA

GCF_001902855 Contig Klebsiella pneumoniae CRK0184 Klebsiella pneumoniae 1 Klebsiella pneumoniae 1 Klebsiella pneumoniae 1 NA

GCF_001902865 Contig Klebsiella pneumoniae CRK0135 Klebsiella pneumoniae 1 Klebsiella pneumoniae 1 Klebsiella pneumoniae 1 NA

GCF_001902875 Contig Klebsiella pneumoniae CRK0131 Klebsiella pneumoniae 1 Klebsiella pneumoniae 1 Klebsiella pneumoniae 1 NA

GCF_001902905 Contig Klebsiella pneumoniae CRK0191 Klebsiella pneumoniae 1 Klebsiella pneumoniae 1 Klebsiella pneumoniae 1 NA

GCF_001902935 Contig Klebsiella pneumoniae CRK0139 Klebsiella pneumoniae 1 Klebsiella pneumoniae 1 Klebsiella pneumoniae 1 NA

GCF_001902945 Contig Klebsiella pneumoniae CRK0136 Klebsiella pneumoniae 1 Klebsiella pneumoniae 1 Klebsiella pneumoniae 1 NA

GCF_001902955 Contig Klebsiella pneumoniae CRK0137 Klebsiella pneumoniae 1 Klebsiella pneumoniae 1 Klebsiella pneumoniae 1 NA

GCF_001902965 Contig Klebsiella pneumoniae CRK0156 Klebsiella pneumoniae 1 Klebsiella pneumoniae 1 Klebsiella pneumoniae 1 NA

GCF_001903015 Contig Klebsiella pneumoniae CRK0153 Klebsiella pneumoniae 1 Klebsiella pneumoniae 1 Klebsiella pneumoniae 1 NA

GCF_001903025 Contig Klebsiella pneumoniae CRK0154 Klebsiella pneumoniae 1 Klebsiella pneumoniae 1 Klebsiella pneumoniae 1 NA

GCF_001903035 Contig Klebsiella pneumoniae CRK0152 Klebsiella pneumoniae 1 Klebsiella pneumoniae 1 Klebsiella pneumoniae 1 NA

GCF_001903045 Contig Klebsiella pneumoniae CRK0145 Klebsiella pneumoniae 1 Klebsiella pneumoniae 1 Klebsiella pneumoniae 1 NA

GCF_001903095 Contig Klebsiella pneumoniae CRK0148 Klebsiella pneumoniae 1 Klebsiella pneumoniae 1 Klebsiella pneumoniae 1 NA

GCF_001903105 Contig Klebsiella pneumoniae CRK0146 Klebsiella pneumoniae 1 Klebsiella pneumoniae 1 Klebsiella pneumoniae 1 NA

GCF_001903125 Contig Klebsiella pneumoniae CRK0186 Klebsiella pneumoniae 1 Klebsiella pneumoniae 1 Klebsiella pneumoniae 1 NA

GCF_001903135 Contig Klebsiella pneumoniae CRK0157 Klebsiella pneumoniae 1 Klebsiella pneumoniae 1 Klebsiella pneumoniae 1 NA

GCF_001903175 Contig Klebsiella pneumoniae CRK0188 Klebsiella pneumoniae 1 Klebsiella pneumoniae 1 Klebsiella pneumoniae 1 NA

GCF_001903195 Contig Klebsiella pneumoniae CRK0187 Klebsiella pneumoniae 1 Klebsiella pneumoniae 1 Klebsiella pneumoniae 1 NA

GCF_001903205 Contig Klebsiella pneumoniae CRK0134 Klebsiella pneumoniae 1 Klebsiella pneumoniae 1 Klebsiella pneumoniae 1 NA

GCF_001903215 Contig Klebsiella pneumoniae CRK0185 Klebsiella pneumoniae 1 Klebsiella pneumoniae 1 Klebsiella pneumoniae 1 NA

GCF_001903255 Contig Klebsiella pneumoniae CRK0133 Klebsiella pneumoniae 1 Klebsiella pneumoniae 1 Klebsiella pneumoniae 1 NA

GCF_001903265 Contig Klebsiella pneumoniae CRK0190 Klebsiella pneumoniae 1 Klebsiella pneumoniae 1 Klebsiella pneumoniae 1 NA

GCF_001903285 Contig Klebsiella pneumoniae CRK0189 Klebsiella pneumoniae 1 Klebsiella pneumoniae 1 Klebsiella pneumoniae 1 NA

GCF_001903295 Contig Klebsiella pneumoniae CRK0142 Klebsiella pneumoniae 1 Klebsiella pneumoniae 1 Klebsiella pneumoniae 1 NA

GCF_001903335 Contig Klebsiella pneumoniae CRK0138 Klebsiella pneumoniae 1 Klebsiella pneumoniae 1 Klebsiella pneumoniae 1 NA

GCF_001903355 Contig Klebsiella pneumoniae CRK0141 Klebsiella pneumoniae 1 Klebsiella pneumoniae 1 Klebsiella pneumoniae 1 NA

GCF_001903365 Contig Klebsiella pneumoniae CRK0144 Klebsiella pneumoniae 1 Klebsiella pneumoniae 1 Klebsiella pneumoniae 1 NA

GCF_001903385 Contig Klebsiella pneumoniae CRK0140 Klebsiella pneumoniae 1 Klebsiella pneumoniae 1 Klebsiella pneumoniae 1 NA

GCF_001903415 Contig Klebsiella pneumoniae CRK0143 Klebsiella pneumoniae 1 Klebsiella pneumoniae 1 Klebsiella pneumoniae 1 NA

GCF_001903435 Contig Klebsiella pneumoniae CRK0149 Klebsiella pneumoniae 1 Klebsiella pneumoniae 1 Klebsiella pneumoniae 1 NA

GCF_001903445 Contig Klebsiella pneumoniae CRK0147 Klebsiella pneumoniae 1 Klebsiella pneumoniae 1 Klebsiella pneumoniae 1 NA

GCF_001903465 Contig Klebsiella pneumoniae CRK0155 Klebsiella pneumoniae 1 Klebsiella pneumoniae 1 Klebsiella pneumoniae 1 NA

GCF_001903495 Contig Klebsiella pneumoniae CRK0181 Klebsiella pneumoniae 1 Klebsiella pneumoniae 1 Klebsiella pneumoniae 1 NA

GCF_001903515 Contig Klebsiella pneumoniae CRK0160 Klebsiella pneumoniae 1 Klebsiella pneumoniae 1 Klebsiella pneumoniae 1 NA

GCF_001903525 Contig Klebsiella pneumoniae CRK0151 Klebsiella pneumoniae 1 Klebsiella pneumoniae 1 Klebsiella pneumoniae 1 NA

GCF_001903535 Contig Klebsiella pneumoniae CRK0175 Klebsiella pneumoniae 1 Klebsiella pneumoniae 1 Klebsiella pneumoniae 1 NA

GCF_001903555 Contig Klebsiella pneumoniae CRK0132 Klebsiella pneumoniae 1 Klebsiella pneumoniae 1 Klebsiella pneumoniae 1 NA

GCF_001903605 Contig Klebsiella pneumoniae CRK0163 Klebsiella pneumoniae 1 Klebsiella pneumoniae 1 Klebsiella pneumoniae 1 NA

GCF_001903615 Contig Klebsiella pneumoniae CRK0164 Klebsiella pneumoniae 1 Klebsiella pneumoniae 1 Klebsiella pneumoniae 1 NA

GCF_001903625 Contig Klebsiella pneumoniae CRK0165 Klebsiella pneumoniae 1 Klebsiella pneumoniae 1 Klebsiella pneumoniae 1 NA

GCF_001903675 Contig Klebsiella pneumoniae CRK0115 Klebsiella pneumoniae 1 Klebsiella pneumoniae 1 Klebsiella pneumoniae 1 NA

GCF_001903685 Contig Klebsiella pneumoniae CRK0102 Klebsiella pneumoniae 1 Klebsiella pneumoniae 1 Klebsiella pneumoniae 1 NA

GCF_001903695 Contig Klebsiella pneumoniae CRK0177 Klebsiella pneumoniae 1 Klebsiella pneumoniae 1 Klebsiella pneumoniae 1 NA

GCF_001903735 Contig Klebsiella pneumoniae CRK0123 Klebsiella pneumoniae 1 Klebsiella pneumoniae 1 Klebsiella pneumoniae 1 NA

GCF_001903755 Contig Klebsiella pneumoniae CRK0183 Klebsiella pneumoniae 1 Klebsiella pneumoniae 1 Klebsiella pneumoniae 1 NA

GCF_001903775 Contig Klebsiella pneumoniae CRK0180 Klebsiella pneumoniae 1 Klebsiella pneumoniae 1 Klebsiella pneumoniae 1 NA

GCF_001903785 Contig Klebsiella pneumoniae CRK0128 Klebsiella pneumoniae 1 Klebsiella pneumoniae 1 Klebsiella pneumoniae 1 NA

GCF_001903795 Contig Klebsiella pneumoniae CRK0118 Klebsiella pneumoniae 1 Klebsiella pneumoniae 1 Klebsiella pneumoniae 1 NA

GCF_001903835 Contig Klebsiella pneumoniae CRK0176 Klebsiella pneumoniae 1 Klebsiella pneumoniae 1 Klebsiella pneumoniae 1 NA

GCF_001903845 Contig Klebsiella pneumoniae CRK0178 Klebsiella pneumoniae 1 Klebsiella pneumoniae 1 Klebsiella pneumoniae 1 NA

GCF_001903865 Contig Klebsiella pneumoniae CRK0182 Klebsiella pneumoniae 1 Klebsiella pneumoniae 1 Klebsiella pneumoniae 1 NA

GCF_001903875 Contig Klebsiella pneumoniae CRK0117 Klebsiella pneumoniae 1 Klebsiella pneumoniae 1 Klebsiella pneumoniae 1 NA

GCF_001903915 Contig Klebsiella pneumoniae CRK0107 Klebsiella pneumoniae 1 Klebsiella pneumoniae 1 Klebsiella pneumoniae 1 NA

GCF_001903925 Contig Klebsiella pneumoniae CRK0119 Klebsiella pneumoniae 1 Klebsiella pneumoniae 1 Klebsiella pneumoniae 1 NA

GCF_001903935 Contig Klebsiella pneumoniae CRK0104 Klebsiella pneumoniae 1 Klebsiella pneumoniae 1 Klebsiella pneumoniae 1 NA

GCF_001903975 Contig Klebsiella pneumoniae CRK0166 Klebsiella pneumoniae 1 Klebsiella pneumoniae 1 Klebsiella pneumoniae 1 NA

GCF_001903995 Contig Klebsiella pneumoniae CRK0121 Klebsiella pneumoniae 1 Klebsiella pneumoniae 1 Klebsiella pneumoniae 1 NA

GCF_001904005 Contig Klebsiella pneumoniae CRK0111 Klebsiella pneumoniae 1 Klebsiella pneumoniae 1 Klebsiella pneumoniae 1 NA

GCF_001904015 Contig Klebsiella pneumoniae CRK0114 Klebsiella pneumoniae 1 Klebsiella pneumoniae 1 Klebsiella pneumoniae 1 NA

GCF_001904045 Contig Klebsiella pneumoniae CRK0179 Klebsiella pneumoniae 1 Klebsiella pneumoniae 1 Klebsiella pneumoniae 1 NA

GCF_001904075 Contig Klebsiella pneumoniae CRK0113 Klebsiella pneumoniae 1 Klebsiella pneumoniae 1 Klebsiella pneumoniae 1 NA

GCF_001904095 Contig Klebsiella pneumoniae CRK0112 Klebsiella pneumoniae 1 Klebsiella pneumoniae 1 Klebsiella pneumoniae 1 NA

GCF_001904105 Contig Klebsiella pneumoniae CRK0110 Klebsiella pneumoniae 1 Klebsiella pneumoniae 1 Klebsiella pneumoniae 1 NA

GCF_001904115 Contig Klebsiella pneumoniae CRK0116 Klebsiella pneumoniae 1 Klebsiella pneumoniae 1 Klebsiella pneumoniae 1 NA

GCF_001904155 Contig Klebsiella pneumoniae CRK0129 Klebsiella pneumoniae 1 Klebsiella pneumoniae 1 Klebsiella pneumoniae 1 NA

GCF_001904165 Contig Klebsiella pneumoniae CRK0126 Klebsiella pneumoniae 1 Klebsiella pneumoniae 1 Klebsiella pneumoniae 1 NA

GCF_001904175 Contig Klebsiella pneumoniae CRK0125 Klebsiella pneumoniae 1 Klebsiella pneumoniae 1 Klebsiella pneumoniae 1 NA

GCF_001904205 Contig Klebsiella pneumoniae CRK0124 Klebsiella pneumoniae 1 Klebsiella pneumoniae 1 Klebsiella pneumoniae 1 NA

GCF_001904235 Contig Klebsiella pneumoniae CRK0120 Klebsiella pneumoniae 1 Klebsiella pneumoniae 1 Klebsiella pneumoniae 1 NA

GCF_001904245 Contig Klebsiella pneumoniae CRK0103 Klebsiella pneumoniae 1 Klebsiella pneumoniae 1 Klebsiella pneumoniae 1 NA

GCF_001904255 Contig Klebsiella pneumoniae CRK0173 Klebsiella pneumoniae 1 Klebsiella pneumoniae 1 Klebsiella pneumoniae 1 NA

GCF_001904295 Contig Klebsiella pneumoniae CRK0109 Klebsiella pneumoniae 1 Klebsiella pneumoniae 1 Klebsiella pneumoniae 1 NA

GCF_001904315 Contig Klebsiella pneumoniae CRK0108 Klebsiella pneumoniae 1 Klebsiella pneumoniae 1 Klebsiella pneumoniae 1 NA

GCF_001904325 Contig Klebsiella pneumoniae CRK0170 Klebsiella pneumoniae 1 Klebsiella pneumoniae 1 Klebsiella pneumoniae 1 NA

GCF_001904335 Contig Klebsiella pneumoniae CRK0168 Klebsiella pneumoniae 1 Klebsiella pneumoniae 1 Klebsiella pneumoniae 1 NA

GCF_001904375 Contig Klebsiella pneumoniae CRK0167 Klebsiella pneumoniae 1 Klebsiella pneumoniae 1 Klebsiella pneumoniae 1 NA

GCF_001904385 Contig Klebsiella pneumoniae CRK0172 Klebsiella pneumoniae 1 Klebsiella pneumoniae 1 Klebsiella pneumoniae 1 NA

GCF_001904405 Contig Klebsiella pneumoniae CRK0169 Klebsiella pneumoniae 1 Klebsiella pneumoniae 1 Klebsiella pneumoniae 1 NA

GCF_001904425 Contig Klebsiella pneumoniae CRK0171 Klebsiella pneumoniae 1 Klebsiella pneumoniae 1 Klebsiella pneumoniae 1 NA

GCF_001904455 Contig Klebsiella pneumoniae CRK0174 Klebsiella pneumoniae 1 Klebsiella pneumoniae 1 Klebsiella pneumoniae 1 NA

GCF_001904475 Contig Klebsiella pneumoniae CRK0127 Klebsiella pneumoniae 1 Klebsiella pneumoniae 1 Klebsiella pneumoniae 1 NA

GCF_001904485 Contig Klebsiella pneumoniae CRK0122 Klebsiella pneumoniae 1 Klebsiella pneumoniae 1 Klebsiella pneumoniae 1 NA

GCF_001904495 Contig Klebsiella pneumoniae CRK0105 Klebsiella pneumoniae 1 Klebsiella pneumoniae 1 Klebsiella pneumoniae 1 NA

GCF_001904535 Contig Klebsiella pneumoniae CRK0106 Klebsiella pneumoniae 1 Klebsiella pneumoniae 1 Klebsiella pneumoniae 1 NA

GCF_001905185 Contig Klebsiella pneumoniae 14382-A Klebsiella pneumoniae 1 Klebsiella pneumoniae 1 Klebsiella pneumoniae 1 NA

GCF_001905195 Contig Klebsiella pneumoniae 15062-D Klebsiella pneumoniae 1 Klebsiella pneumoniae 1 Klebsiella pneumoniae 1 NA

GCF_001905225 Contig Klebsiella pneumoniae 15203-B Klebsiella pneumoniae 1 Klebsiella pneumoniae 1 Klebsiella pneumoniae 1 NA

GCF_001905235 Contig Klebsiella pneumoniae 15263-C Klebsiella pneumoniae 1 Klebsiella pneumoniae 1 Klebsiella pneumoniae 1 NA

GCF_001905315 Contig Klebsiella pneumoniae 14393-A Klebsiella pneumoniae 1 Klebsiella pneumoniae 1 Klebsiella pneumoniae 1 NA

GCF_001906645 Contig Klebsiella pneumoniae 14922-C Klebsiella pneumoniae 1 Klebsiella pneumoniae 1 Klebsiella pneumoniae 1 NA

GCF_001906685 Contig Klebsiella pneumoniae 14912-B Klebsiella pneumoniae 1 Klebsiella pneumoniae 1 Klebsiella pneumoniae 1 NA

GCF_001906705 Contig Klebsiella pneumoniae 14461-B Klebsiella pneumoniae 1 Klebsiella pneumoniae 1 Klebsiella pneumoniae 1 NA

GCF_001906715 Contig Klebsiella pneumoniae 14311-C Klebsiella pneumoniae 1 Klebsiella pneumoniae 1 Klebsiella pneumoniae 1 NA

GCF_001906725 Contig Klebsiella pneumoniae 14211-A Klebsiella pneumoniae 1 Klebsiella pneumoniae 1 Klebsiella pneumoniae 1 NA

GCF_001907955 Contig Klebsiella pneumoniae 301 Klebsiella pneumoniae 1 Klebsiella pneumoniae 1 Klebsiella pneumoniae 1 NA

GCF_001908515 Complete Klebsiella pneumoniae CAV1042 Klebsiella pneumoniae 1 Klebsiella pneumoniae 1 Klebsiella pneumoniae 1 NA

GCF_001908595 Complete Klebsiella pneumoniae Kp Goe 149473 Klebsiella pneumoniae 1 Klebsiella pneumoniae 1 Klebsiella pneumoniae 1 NA

GCF_001908625 Complete Klebsiella pneumoniae Kp Goe 827024 Klebsiella pneumoniae 1 Klebsiella pneumoniae 1 Klebsiella pneumoniae 1 NA

GCF_001908655 Complete Klebsiella pneumoniae Kp Goe 827026 Klebsiella pneumoniae 1 Klebsiella pneumoniae 1 Klebsiella pneumoniae 1 NA

GCF_001908675 Complete Klebsiella pneumoniae Kp Goe 152021 Klebsiella pneumoniae 1 Klebsiella pneumoniae 1 Klebsiella pneumoniae 1 NA

GCF_001908695 Complete Klebsiella pneumoniae KP Goe 828304 Klebsiella pneumoniae 1 Klebsiella pneumoniae 1 Klebsiella pneumoniae 1 NA

GCF_001908715 Complete Klebsiella pneumoniae CAV1217 Klebsiella pneumoniae 1 Klebsiella pneumoniae 1 Klebsiella pneumoniae 1 NA

GCF_001908875 Complete Klebsiella pneumoniae Kp Goe 149832 Klebsiella pneumoniae 1 Klebsiella pneumoniae 1 Klebsiella pneumoniae 1 NA

GCF_001908895 Complete Klebsiella pneumoniae Kp Goe 821588 Klebsiella pneumoniae 1 Klebsiella pneumoniae 1 Klebsiella pneumoniae 1 NA

GCF_001913175 Complete Klebsiella pneumoniae Kp Goe 121641 Klebsiella pneumoniae 1 Klebsiella pneumoniae 1 Klebsiella pneumoniae 1 NA

GCF_001922465 Complete Klebsiella pneumoniae AR0049 Klebsiella pneumoniae 1 Klebsiella pneumoniae 1 Klebsiella pneumoniae 1 NA

GCF_001929345 Contig Klebsiella pneumoniae pneumoniae ATCC9621 Klebsiella pneumoniae 1 Klebsiella pneumoniae 1 Klebsiella pneumoniae 1 NA

GCF_001931705 Contig Klebsiella pneumoniae 38 wz Klebsiella pneumoniae 1 Klebsiella pneumoniae 1 Klebsiella pneumoniae 1 NA

GCF_001936035 Complete Klebsiella pneumoniae ATCC35657 Klebsiella pneumoniae 1 Klebsiella pneumoniae 1 Klebsiella pneumoniae 1 NA

GCF_001938035 Contig Klebsiella pneumoniae KLEB-LEB Klebsiella pneumoniae 1 Klebsiella pneumoniae 1 Klebsiella pneumoniae 1 NA

GCF_001938605 Contig Klebsiella michiganensis CAV1755 Klebsiella michiganensis 1 Klebsiella michiganensis 1 Klebsiella michiganensis 1 NA

GCF_001939855 Contig Klebsiella pneumoniae GN3 Klebsiella pneumoniae 1 Klebsiella pneumoniae 1 Klebsiella pneumoniae 1 NA

GCF_001939885 Complete Klebsiella pneumoniae GN2 Klebsiella pneumoniae 1 Klebsiella pneumoniae 1 Klebsiella pneumoniae 1 NA

GCF_001939895 Contig Klebsiella aerogenes eae Klebsiella aerogenes 1 Klebsiella aerogenes Klebsiella aerogenes NA

GCF_001945455 Contig Klebsiella michiganensis 97 38 Klebsiella michiganensis 1 Klebsiella michiganensis 1 Klebsiella michiganensis 1 NA

GCF_001945475 Contig Klebsiella pneumoniae 97 58 Klebsiella pneumoniae 1 Klebsiella pneumoniae 1 Klebsiella pneumoniae 1 NA

GCF_001950635 Contig Klebsiella pneumoniae MRY10-808 Klebsiella pneumoniae 1 Klebsiella pneumoniae 1 Klebsiella pneumoniae 1 NA

GCF_001950655 Contig Klebsiella pneumoniae MRY10-848 Klebsiella pneumoniae 1 Klebsiella pneumoniae 1 Klebsiella pneumoniae 1 NA

GCF_001950675 Contig Klebsiella pneumoniae MRY10-897 Klebsiella pneumoniae 1 Klebsiella pneumoniae 1 Klebsiella pneumoniae 1 NA

GCF_001952835 Complete Klebsiella pneumoniae 1756 Klebsiella pneumoniae 1 Klebsiella pneumoniae 1 Klebsiella pneumoniae 1 NA

GCF_001952875 Complete Klebsiella pneumoniae CN1 Klebsiella pneumoniae 1 Klebsiella pneumoniae 1 Klebsiella pneumoniae 1 NA

GCF_001952895 Complete Klebsiella pneumoniae NY9 Klebsiella pneumoniae 1 Klebsiella pneumoniae 1 Klebsiella pneumoniae 1 NA

GCF_001952915 Complete Klebsiella pneumoniae CR14 Klebsiella pneumoniae 1 Klebsiella pneumoniae 1 Klebsiella pneumoniae 1 NA

GCF_001956965 Complete Klebsiella pneumoniae 825795-1 Klebsiella pneumoniae 1 Klebsiella pneumoniae 1 Klebsiella pneumoniae 1 NA

GCF_001968745 Scaffold Klebsiella pneumoniae 196 Klebsiella pneumoniae 1 Klebsiella pneumoniae 1 Klebsiella pneumoniae 1 NA

GCF_001968755 Scaffold Klebsiella pneumoniae 148 Klebsiella pneumoniae 1 Klebsiella pneumoniae 1 Klebsiella pneumoniae 1 NA

GCF_001969305 Complete Klebsiella variicola LMG23571 Klebsiella variicola Klebsiella variicola Klebsiella variicola NA

GCF_001970155 Contig Klebsiella pneumoniae KP254 Klebsiella pneumoniae 1 Klebsiella pneumoniae 1 Klebsiella pneumoniae 1 NA

GCF_001970165 Contig Klebsiella pneumoniae KP1083 Klebsiella pneumoniae 1 Klebsiella pneumoniae 1 Klebsiella pneumoniae 1 NA

GCF_001970175 Contig Klebsiella pneumoniae KP314 Klebsiella pneumoniae 1 Klebsiella pneumoniae 1 Klebsiella pneumoniae 1 NA

GCF_001970835 Complete Klebsiella oxytoca CAV1752 Klebsiella michiganensis 1 Klebsiella michiganensis 1 Klebsiella michiganensis 1 NA

GCF_001974865 Contig Klebsiella aerogenes Klebsiella aerogenes 1 Klebsiella aerogenes Klebsiella aerogenes NA

GCF_001984895 Contig Klebsiella pneumoniae KP41-2015 Klebsiella pneumoniae 1 Klebsiella pneumoniae 1 Klebsiella pneumoniae 1 NA

GCF_001989495 Complete Klebsiella variicola GJ1 Klebsiella variicola Klebsiella variicola Klebsiella variicola NA

GCF_001989515 Complete Klebsiella variicola GJ2 Klebsiella variicola Klebsiella variicola Klebsiella variicola NA

GCF_001989535 Complete Klebsiella variicola GJ3 Klebsiella variicola Klebsiella variicola Klebsiella variicola NA

GCF_002007665 Chromosome Klebsiella pneumoniae pneumoniae KPN KPC HUG07 Klebsiella pneumoniae 1 Klebsiella pneumoniae 1 Klebsiella pneumoniae 1 NA

GCF_002055765 Complete Klebsiella pneumoniae AR0117 Klebsiella pneumoniae 1 Klebsiella pneumoniae 1 Klebsiella pneumoniae 1 NA

GCF_002055855 Complete Klebsiella pneumoniae AR0068 Klebsiella pneumoniae 1 Klebsiella pneumoniae 1 Klebsiella pneumoniae 1 NA

GCF_002056385 Complete Klebsiella pneumoniae AR0115 Klebsiella pneumoniae 1 Klebsiella pneumoniae 1 Klebsiella pneumoniae 1 NA

GCF_002056475 Complete Klebsiella pneumoniae AR0098 Klebsiella pneumoniae 1 Klebsiella pneumoniae 1 Klebsiella pneumoniae 1 NA

GCF_002072655 Complete Klebsiella oxytoca AR0147 Klebsiella michiganensis 1 Klebsiella michiganensis 1 Klebsiella michiganensis 1 NA

GCF_002080105 Scaffold Klebsiella oxytoca DSM29614 Klebsiella grimontii Klebsiella grimontii Klebsiella grimontii NA

GCF_002085855 Scaffold Klebsiella pneumoniae KPN KPC HUG08 Klebsiella pneumoniae 1 Klebsiella pneumoniae 1 Klebsiella pneumoniae 1 NA

GCF_002085865 Scaffold Klebsiella pneumoniae KPN KPC HUG09 Klebsiella pneumoniae 1 Klebsiella pneumoniae 1 Klebsiella pneumoniae 1 NA

GCF_002085895 Scaffold Klebsiella pneumoniae pneumoniae KPN KPC HUG11 Klebsiella pneumoniae 1 Klebsiella pneumoniae 1 Klebsiella pneumoniae 1 NA

GCF_002085915 Scaffold Klebsiella pneumoniae pneumoniae KPN KPC HUG10 Klebsiella pneumoniae 1 Klebsiella pneumoniae 1 Klebsiella pneumoniae 1 NA

GCF_002085935 Scaffold Klebsiella pneumoniae pneumoniae KPN KPC HUG12 Klebsiella pneumoniae 1 Klebsiella pneumoniae 1 Klebsiella pneumoniae 1 NA

GCF_002085975 Scaffold Klebsiella pneumoniae pneumoniae KPN KPC HUG13 Klebsiella pneumoniae 1 Klebsiella pneumoniae 1 Klebsiella pneumoniae 1 NA

GCF_002085995 Scaffold Klebsiella pneumoniae pneumoniae KPN KPC HUG B1 Klebsiella pneumoniae 1 Klebsiella pneumoniae 1 Klebsiella pneumoniae 1 NA

GCF_002086005 Scaffold Klebsiella pneumoniae pneumoniae KPN KPC HUG B3 Klebsiella pneumoniae 1 Klebsiella pneumoniae 1 Klebsiella pneumoniae 1 NA

GCF_002086025 Scaffold Klebsiella pneumoniae pneumoniae KPN KPC HUG B2 Klebsiella pneumoniae 1 Klebsiella pneumoniae 1 Klebsiella pneumoniae 1 NA

GCF_002086055 Scaffold Klebsiella pneumoniae KPN KPC HUG B4 Klebsiella pneumoniae 1 Klebsiella pneumoniae 1 Klebsiella pneumoniae 1 NA

GCF_002086075 Scaffold Klebsiella pneumoniae pneumoniae KPN KPC HUG B5 Klebsiella pneumoniae 1 Klebsiella pneumoniae 1 Klebsiella pneumoniae 1 NA

GCF_002086095 Scaffold Klebsiella pneumoniae pneumoniae KPN KPC HUG B6 Klebsiella pneumoniae 1 Klebsiella pneumoniae 1 Klebsiella pneumoniae 1 NA

GCF_002087215 Contig Klebsiella pneumoniae pneumoniae KpvST147L NDM Klebsiella pneumoniae 1 Klebsiella pneumoniae 1 Klebsiella pneumoniae 1 NA

GCF_002087495 Scaffold Klebsiella pneumoniae OS09 011 10 Klebsiella pneumoniae 1 Klebsiella pneumoniae 1 Klebsiella pneumoniae 1 NA

GCF_002087645 Scaffold Klebsiella pneumoniae OS09 011 15 Klebsiella pneumoniae 1 Klebsiella pneumoniae 1 Klebsiella pneumoniae 1 NA

GCF_002087715 Scaffold Klebsiella pneumoniae OS09 011 14 Klebsiella pneumoniae 1 Klebsiella pneumoniae 1 Klebsiella pneumoniae 1 NA

GCF_002090195 Complete Klebsiella sp M5al Klebsiella grimontii Klebsiella grimontii Klebsiella grimontii NA

GCF_002099165 Scaffold Klebsiella pneumoniae CCBH24080 Klebsiella pneumoniae 1 Klebsiella pneumoniae 1 Klebsiella pneumoniae 1 NA

GCF_002102635 Scaffold Klebsiella pneumoniae Kp703 Klebsiella pneumoniae 1 Klebsiella pneumoniae 1 Klebsiella pneumoniae 1 NA

GCF_002102655 Scaffold Klebsiella pneumoniae Kp45 Klebsiella pneumoniae 1 Klebsiella pneumoniae 1 Klebsiella pneumoniae 1 NA

GCF_002102665 Scaffold Klebsiella pneumoniae Kp2948 Klebsiella pneumoniae 1 Klebsiella pneumoniae 1 Klebsiella pneumoniae 1 NA

GCF_002103515 Scaffold Klebsiella pneumoniae MNCRE82 Klebsiella pneumoniae 1 Klebsiella pneumoniae 1 Klebsiella pneumoniae 1 NA

GCF_002103525 Scaffold Klebsiella pneumoniae MNCRE80 Klebsiella pneumoniae 1 Klebsiella pneumoniae 1 Klebsiella pneumoniae 1 NA

GCF_002103555 Scaffold Klebsiella pneumoniae MNCRE54 Klebsiella pneumoniae 1 Klebsiella pneumoniae 1 Klebsiella pneumoniae 1 NA

GCF_002103565 Scaffold Klebsiella pneumoniae MNCRE60 Klebsiella pneumoniae 1 Klebsiella pneumoniae 1 Klebsiella pneumoniae 1 NA

GCF_002103595 Scaffold Klebsiella pneumoniae MNCRE61 Klebsiella pneumoniae 1 Klebsiella pneumoniae 1 Klebsiella pneumoniae 1 NA

GCF_002103605 Scaffold Klebsiella pneumoniae MNCRE64 Klebsiella pneumoniae 1 Klebsiella pneumoniae 1 Klebsiella pneumoniae 1 NA

GCF_002103635 Scaffold Klebsiella pneumoniae MNCRE66 Klebsiella pneumoniae 1 Klebsiella pneumoniae 1 Klebsiella pneumoniae 1 NA

GCF_002103645 Scaffold Klebsiella pneumoniae MNCRE67 Klebsiella pneumoniae 1 Klebsiella pneumoniae 1 Klebsiella pneumoniae 1 NA

GCF_002103655 Scaffold Klebsiella pneumoniae MNCRE68 Klebsiella pneumoniae 1 Klebsiella pneumoniae 1 Klebsiella pneumoniae 1 NA

GCF_002103675 Scaffold Klebsiella pneumoniae MNCRE70 Klebsiella pneumoniae 1 Klebsiella pneumoniae 1 Klebsiella pneumoniae 1 NA

GCF_002103715 Scaffold Klebsiella pneumoniae MNCRE74 Klebsiella pneumoniae 1 Klebsiella pneumoniae 1 Klebsiella pneumoniae 1 NA

GCF_002103725 Scaffold Klebsiella pneumoniae MNCRE73 Klebsiella pneumoniae 1 Klebsiella pneumoniae 1 Klebsiella pneumoniae 1 NA

GCF_002103735 Scaffold Klebsiella pneumoniae MNCRE75 Klebsiella pneumoniae 1 Klebsiella pneumoniae 1 Klebsiella pneumoniae 1 NA

GCF_002103745 Scaffold Klebsiella pneumoniae MNCRE77 Klebsiella pneumoniae 1 Klebsiella pneumoniae 1 Klebsiella pneumoniae 1 NA

GCF_002104175 Contig Klebsiella pneumoniae 1714 Klebsiella pneumoniae 1 Klebsiella pneumoniae 1 Klebsiella pneumoniae 1 NA

GCF_002104185 Contig Klebsiella pneumoniae 4490 Klebsiella pneumoniae 1 Klebsiella pneumoniae 1 Klebsiella pneumoniae 1 NA

GCF_002104215 Contig Klebsiella pneumoniae 4182 Klebsiella pneumoniae 1 Klebsiella pneumoniae 1 Klebsiella pneumoniae 1 NA

GCF_002104255 Contig Klebsiella pneumoniae 4281 Klebsiella pneumoniae 1 Klebsiella pneumoniae 1 Klebsiella pneumoniae 1 NA

GCF_002104265 Contig Klebsiella pneumoniae 1849 Klebsiella pneumoniae 1 Klebsiella pneumoniae 1 Klebsiella pneumoniae 1 NA

GCF_002104295 Contig Klebsiella pneumoniae 4448 Klebsiella pneumoniae 1 Klebsiella pneumoniae 1 Klebsiella pneumoniae 1 NA

GCF_002104315 Contig Klebsiella pneumoniae 1799 Klebsiella pneumoniae 1 Klebsiella pneumoniae 1 Klebsiella pneumoniae 1 NA

GCF_002104355 Contig Klebsiella pneumoniae 1787 Klebsiella pneumoniae 1 Klebsiella pneumoniae 1 Klebsiella pneumoniae 1 NA

GCF_002104365 Contig Klebsiella pneumoniae 4477 Klebsiella pneumoniae 1 Klebsiella pneumoniae 1 Klebsiella pneumoniae 1 NA

GCF_002104395 Scaffold Klebsiella pneumoniae JM2CRO Klebsiella pneumoniae 1 Klebsiella pneumoniae 1 Klebsiella pneumoniae 1 NA

GCF_002104635 Scaffold Klebsiella pneumoniae BO2 Klebsiella pneumoniae 1 Klebsiella pneumoniae 1 Klebsiella pneumoniae 1 NA

GCF_002104705 Contig Klebsiella pneumoniae 1853 Klebsiella pneumoniae 1 Klebsiella pneumoniae 1 Klebsiella pneumoniae 1 NA

GCF_002104715 Contig Klebsiella pneumoniae 4326 Klebsiella pneumoniae 1 Klebsiella pneumoniae 1 Klebsiella pneumoniae 1 NA

GCF_002105205 Scaffold Klebsiella pneumoniae 314 Klebsiella pneumoniae 1 Klebsiella pneumoniae 1 Klebsiella pneumoniae 1 NA

GCF_002108235 Scaffold Klebsiella pneumoniae ARPG340 Klebsiella pneumoniae 1 Klebsiella pneumoniae 1 Klebsiella pneumoniae 1 NA

GCF_002108245 Scaffold Klebsiella pneumoniae ARPG372 Klebsiella pneumoniae 1 Klebsiella pneumoniae 1 Klebsiella pneumoniae 1 NA

GCF_002108275 Scaffold Klebsiella pneumoniae ARPG318 Klebsiella pneumoniae 1 Klebsiella pneumoniae 1 Klebsiella pneumoniae 1 NA

GCF_002108285 Scaffold Klebsiella pneumoniae ARPG379 Klebsiella pneumoniae 1 Klebsiella pneumoniae 1 Klebsiella pneumoniae 1 NA

GCF_002108315 Scaffold Klebsiella pneumoniae ARPG315 Klebsiella pneumoniae 1 Klebsiella pneumoniae 1 Klebsiella pneumoniae 1 NA

GCF_002108335 Scaffold Klebsiella pneumoniae ARP657 Klebsiella pneumoniae 1 Klebsiella pneumoniae 1 Klebsiella pneumoniae 1 NA

GCF_002108345 Scaffold Klebsiella pneumoniae ARPG281 Klebsiella pneumoniae 1 Klebsiella pneumoniae 1 Klebsiella pneumoniae 1 NA

GCF_002108355 Scaffold Klebsiella pneumoniae ARP664 Klebsiella pneumoniae 1 Klebsiella pneumoniae 1 Klebsiella pneumoniae 1 NA

GCF_002108395 Scaffold Klebsiella pneumoniae ARPG489 Klebsiella pneumoniae 1 Klebsiella pneumoniae 1 Klebsiella pneumoniae 1 NA

GCF_002108405 Scaffold Klebsiella pneumoniae ARPG380 Klebsiella pneumoniae 1 Klebsiella pneumoniae 1 Klebsiella pneumoniae 1 NA

GCF_002108415 Scaffold Klebsiella pneumoniae ARPG381 Klebsiella pneumoniae 1 Klebsiella pneumoniae 1 Klebsiella pneumoniae 1 NA

GCF_002111445 Scaffold Klebsiella oxytoca TDB1 Klebsiella michiganensis 1 Klebsiella michiganensis 1 Klebsiella michiganensis 1 NA

GCF_002113865 Complete Klebsiella pneumoniae K66-45 Klebsiella pneumoniae 1 Klebsiella pneumoniae 1 Klebsiella pneumoniae 1 NA

GCF_002114225 Scaffold Klebsiella pneumoniae ICIS278 PBV Klebsiella pneumoniae 1 Klebsiella pneumoniae 1 Klebsiella pneumoniae 1 NA

GCF_002115605 Contig Klebsiella pneumoniae KP1246 Klebsiella pneumoniae 1 Klebsiella pneumoniae 1 Klebsiella pneumoniae 1 NA

GCF_002115625 Contig Klebsiella pneumoniae KP1244 Klebsiella pneumoniae 1 Klebsiella pneumoniae 1 Klebsiella pneumoniae 1 NA

GCF_002115645 Contig Klebsiella pneumoniae KP1245 Klebsiella pneumoniae 1 Klebsiella pneumoniae 1 Klebsiella pneumoniae 1 NA

GCF_002116885 Complete Klebsiella pneumoniae kp757 Klebsiella pneumoniae 1 Klebsiella pneumoniae 1 Klebsiella pneumoniae 1 NA

GCF_002119075 Contig Klebsiella pneumoniae Kp76 Klebsiella pneumoniae 1 Klebsiella pneumoniae 1 Klebsiella pneumoniae 1 NA

GCF_002119085 Contig Klebsiella pneumoniae KPHU468 Klebsiella pneumoniae 1 Klebsiella pneumoniae 1 Klebsiella pneumoniae 1 NA

GCF_002119865 Contig Klebsiella variicola KP007 Klebsiella variicola Klebsiella variicola Klebsiella variicola NA

GCF_002119875 Contig Klebsiella michiganensis 3T412C Klebsiella michiganensis 1 Klebsiella michiganensis 1 Klebsiella michiganensis 1 NA

GCF_002119885 Contig Klebsiella pneumoniae KL027 Klebsiella pneumoniae 1 Klebsiella pneumoniae 1 Klebsiella pneumoniae 1 NA

GCF_002119895 Contig Klebsiella pneumoniae KL033 Klebsiella pneumoniae 1 Klebsiella pneumoniae 1 Klebsiella pneumoniae 1 NA

GCF_002119945 Contig Klebsiella quasipneumoniae KP025 Klebsiella quasipneumoniae 2 Klebsiella quasipneumoniae 2 Klebsiella quasipneumoniae NA

GCF_002119955 Contig Klebsiella pneumoniae KP024 Klebsiella pneumoniae 1 Klebsiella pneumoniae 1 Klebsiella pneumoniae 1 NA

GCF_002120065 Contig Klebsiella pneumoniae KL064 Klebsiella pneumoniae 1 Klebsiella pneumoniae 1 Klebsiella pneumoniae 1 NA

GCF_002120105 Contig Klebsiella pneumoniae KL015 Klebsiella pneumoniae 1 Klebsiella pneumoniae 1 Klebsiella pneumoniae 1 NA

GCF_002140135 Scaffold Klebsiella pneumoniae WM-Tun-3771 Klebsiella pneumoniae 1 Klebsiella pneumoniae 1 Klebsiella pneumoniae 1 NA

GCF_002148835 Scaffold Klebsiella pneumoniae KPN11 Klebsiella pneumoniae 1 Klebsiella pneumoniae 1 Klebsiella pneumoniae 1 NA

GCF_002150645 Scaffold Klebsiella pneumoniae C2-D8-12 Klebsiella pneumoniae 1 Klebsiella pneumoniae 1 Klebsiella pneumoniae 1 NA

GCF_002152295 Scaffold Klebsiella variicola BIDMC118 Klebsiella variicola Klebsiella variicola Klebsiella variicola NA

GCF_002152315 Scaffold Klebsiella michiganensis MGH175 Klebsiella michiganensis 1 Klebsiella michiganensis 1 Klebsiella michiganensis 1 NA

GCF_002152325 Scaffold Klebsiella michiganensis MGH176 Klebsiella michiganensis 1 Klebsiella michiganensis 1 Klebsiella michiganensis 1 NA

GCF_002152355 Scaffold Klebsiella pneumoniae BWH66 Klebsiella pneumoniae 1 Klebsiella pneumoniae 1 Klebsiella pneumoniae 1 NA

GCF_002152365 Scaffold Klebsiella pneumoniae BWH67 Klebsiella pneumoniae 1 Klebsiella pneumoniae 1 Klebsiella pneumoniae 1 NA

GCF_002152395 Scaffold Klebsiella pneumoniae CHS242 Klebsiella pneumoniae 1 Klebsiella pneumoniae 1 Klebsiella pneumoniae 1 NA

GCF_002152405 Scaffold Klebsiella pneumoniae MGH133 Klebsiella pneumoniae 1 Klebsiella pneumoniae 1 Klebsiella pneumoniae 1 NA

GCF_002152435 Scaffold Klebsiella pneumoniae MGH134 Klebsiella pneumoniae 1 Klebsiella pneumoniae 1 Klebsiella pneumoniae 1 NA

GCF_002152445 Scaffold Klebsiella pneumoniae MGH147 Klebsiella pneumoniae 1 Klebsiella pneumoniae 1 Klebsiella pneumoniae 1 NA

GCF_002152475 Scaffold Klebsiella pneumoniae MGH148 Klebsiella pneumoniae 1 Klebsiella pneumoniae 1 Klebsiella pneumoniae 1 NA

GCF_002152485 Scaffold Klebsiella pneumoniae MGH153 Klebsiella pneumoniae 1 Klebsiella pneumoniae 1 Klebsiella pneumoniae 1 NA

GCF_002152515 Scaffold Klebsiella pneumoniae MGH155 Klebsiella pneumoniae 1 Klebsiella pneumoniae 1 Klebsiella pneumoniae 1 NA

GCF_002152525 Scaffold Klebsiella pneumoniae MGH157 Klebsiella pneumoniae 1 Klebsiella pneumoniae 1 Klebsiella pneumoniae 1 NA

GCF_002152555 Scaffold Klebsiella pneumoniae MGH158 Klebsiella pneumoniae 1 Klebsiella pneumoniae 1 Klebsiella pneumoniae 1 NA

GCF_002152565 Scaffold Klebsiella pneumoniae MGH162 Klebsiella pneumoniae 1 Klebsiella pneumoniae 1 Klebsiella pneumoniae 1 NA

GCF_002152595 Scaffold Klebsiella pneumoniae MGH165 Klebsiella pneumoniae 1 Klebsiella pneumoniae 1 Klebsiella pneumoniae 1 NA

GCF_002152605 Scaffold Klebsiella pneumoniae MGH166 Klebsiella pneumoniae 1 Klebsiella pneumoniae 1 Klebsiella pneumoniae 1 NA

GCF_002152635 Scaffold Klebsiella pneumoniae MGH167 Klebsiella pneumoniae 1 Klebsiella pneumoniae 1 Klebsiella pneumoniae 1 NA

GCF_002152655 Scaffold Klebsiella pneumoniae MGH172 Klebsiella pneumoniae 1 Klebsiella pneumoniae 1 Klebsiella pneumoniae 1 NA

GCF_002152675 Scaffold Klebsiella pneumoniae MGH185 Klebsiella pneumoniae 1 Klebsiella pneumoniae 1 Klebsiella pneumoniae 1 NA

GCF_002152685 Scaffold Klebsiella pneumoniae MGH186 Klebsiella pneumoniae 1 Klebsiella pneumoniae 1 Klebsiella pneumoniae 1 NA

GCF_002152695 Scaffold Klebsiella pneumoniae UCI106 Klebsiella pneumoniae 1 Klebsiella pneumoniae 1 Klebsiella pneumoniae 1 NA

GCF_002152735 Scaffold Klebsiella pneumoniae UCI109 Klebsiella pneumoniae 1 Klebsiella pneumoniae 1 Klebsiella pneumoniae 1 NA

GCF_002152755 Scaffold Klebsiella pneumoniae UCI113 Klebsiella pneumoniae 1 Klebsiella pneumoniae 1 Klebsiella pneumoniae 1 NA

GCF_002152765 Scaffold Klebsiella pneumoniae UCI112 Klebsiella pneumoniae 1 Klebsiella pneumoniae 1 Klebsiella pneumoniae 1 NA

GCF_002152775 Scaffold Klebsiella pneumoniae UCI114 Klebsiella pneumoniae 1 Klebsiella pneumoniae 1 Klebsiella pneumoniae 1 NA

GCF_002152815 Scaffold Klebsiella pneumoniae UCI115 Klebsiella pneumoniae 1 Klebsiella pneumoniae 1 Klebsiella pneumoniae 1 NA

GCF_002152825 Scaffold Klebsiella pneumoniae UCI116 Klebsiella pneumoniae 1 Klebsiella pneumoniae 1 Klebsiella pneumoniae 1 NA

GCF_002152865 Scaffold Klebsiella pneumoniae UCI118 Klebsiella pneumoniae 1 Klebsiella pneumoniae 1 Klebsiella pneumoniae 1 NA

GCF_002152895 Scaffold Klebsiella aerogenes MGH174 Klebsiella aerogenes 1 Klebsiella aerogenes Klebsiella aerogenes NA

GCF_002152915 Scaffold Klebsiella aerogenes UCI119 Klebsiella aerogenes 1 Klebsiella aerogenes Klebsiella aerogenes NA

GCF_002152925 Scaffold Klebsiella aerogenes UCI120 Klebsiella aerogenes 1 Klebsiella aerogenes Klebsiella aerogenes NA

GCF_002153075 Scaffold Klebsiella variicola BIDMC117 Klebsiella variicola Klebsiella variicola Klebsiella variicola NA

GCF_002153085 Scaffold Klebsiella pneumoniae CHS241 Klebsiella pneumoniae 1 Klebsiella pneumoniae 1 Klebsiella pneumoniae 1 NA

GCF_002153105 Scaffold Klebsiella pneumoniae MGH140 Klebsiella pneumoniae 1 Klebsiella pneumoniae 1 Klebsiella pneumoniae 1 NA

GCF_002153115 Scaffold Klebsiella pneumoniae MGH149 Klebsiella pneumoniae 1 Klebsiella pneumoniae 1 Klebsiella pneumoniae 1 NA

GCF_002153155 Scaffold Klebsiella pneumoniae MGH168 Klebsiella pneumoniae 1 Klebsiella pneumoniae 1 Klebsiella pneumoniae 1 NA

GCF_002153165 Scaffold Klebsiella pneumoniae MGH156 Klebsiella pneumoniae 1 Klebsiella pneumoniae 1 Klebsiella pneumoniae 1 NA

GCF_002153175 Scaffold Klebsiella pneumoniae MGH161 Klebsiella pneumoniae 1 Klebsiella pneumoniae 1 Klebsiella pneumoniae 1 NA

GCF_002153215 Scaffold Klebsiella pneumoniae UCI105 Klebsiella pneumoniae 1 Klebsiella pneumoniae 1 Klebsiella pneumoniae 1 NA

GCF_002153225 Scaffold Klebsiella pneumoniae MGH181 Klebsiella pneumoniae 1 Klebsiella pneumoniae 1 Klebsiella pneumoniae 1 NA

GCF_002153255 Scaffold Klebsiella pneumoniae UCI110 Klebsiella pneumoniae 1 Klebsiella pneumoniae 1 Klebsiella pneumoniae 1 NA

GCF_002153275 Scaffold Klebsiella pneumoniae MGH171 Klebsiella pneumoniae 1 Klebsiella pneumoniae 1 Klebsiella pneumoniae 1 NA

GCF_002153285 Scaffold Klebsiella pneumoniae UCI107 Klebsiella pneumoniae 1 Klebsiella pneumoniae 1 Klebsiella pneumoniae 1 NA

GCF_002153305 Scaffold Klebsiella pneumoniae UCI111 Klebsiella pneumoniae 1 Klebsiella pneumoniae 1 Klebsiella pneumoniae 1 NA

GCF_002154825 Contig Klebsiella pneumoniae SJRP/Kp10 Klebsiella pneumoniae 1 Klebsiella pneumoniae 1 Klebsiella pneumoniae 1 NA

GCF_002156725 Complete Klebsiella pneumoniae BK13043 Klebsiella pneumoniae 1 Klebsiella pneumoniae 1 Klebsiella pneumoniae 1 NA

GCF_002156745 Complete Klebsiella pneumoniae KPN1482 Klebsiella pneumoniae 1 Klebsiella pneumoniae 1 Klebsiella pneumoniae 1 NA

GCF_002156765 Complete Klebsiella variicola KPN1481 Klebsiella variicola Klebsiella variicola Klebsiella variicola NA

GCF_002156785 Complete Klebsiella pneumoniae KPN528 Klebsiella pneumoniae 1 Klebsiella pneumoniae 1 Klebsiella pneumoniae 1 NA

GCF_002157345 Contig Klebsiella pneumoniae ICBKpBL-III021 Klebsiella pneumoniae 1 Klebsiella pneumoniae 1 Klebsiella pneumoniae 1 NA

GCF_002162395 Contig Klebsiella pneumoniae 39384 Klebsiella pneumoniae 1 Klebsiella pneumoniae 1 Klebsiella pneumoniae 1 NA

GCF_002162405 Contig Klebsiella pneumoniae 40889 Klebsiella pneumoniae 1 Klebsiella pneumoniae 1 Klebsiella pneumoniae 1 NA

GCF_002162425 Contig Klebsiella pneumoniae 39884 Klebsiella pneumoniae 1 Klebsiella pneumoniae 1 Klebsiella pneumoniae 1 NA

GCF_002163225 Scaffold Klebsiella pneumoniae UCI108 Klebsiella pneumoniae 1 Klebsiella pneumoniae 1 Klebsiella pneumoniae 1 NA

GCF_002163895 Complete Klebsiella pneumoniae pneumoniae RJA166 Klebsiella pneumoniae 1 Klebsiella pneumoniae 1 Klebsiella pneumoniae 1 NA

GCF_002164605 Scaffold Klebsiella pneumoniae WCHKP113 Klebsiella pneumoniae 1 Klebsiella pneumoniae 1 Klebsiella pneumoniae 1 NA

GCF_002164675 Scaffold Klebsiella pneumoniae WCHKP108 Klebsiella pneumoniae 1 Klebsiella pneumoniae 1 Klebsiella pneumoniae 1 NA

GCF_002164685 Scaffold Klebsiella pneumoniae WCHKP103 Klebsiella pneumoniae 1 Klebsiella pneumoniae 1 Klebsiella pneumoniae 1 NA

GCF_002164695 Scaffold Klebsiella pneumoniae WCHKP97 Klebsiella pneumoniae 1 Klebsiella pneumoniae 1 Klebsiella pneumoniae 1 NA

GCF_002164725 Scaffold Klebsiella pneumoniae WCHKP95 Klebsiella pneumoniae 1 Klebsiella pneumoniae 1 Klebsiella pneumoniae 1 NA

GCF_002164755 Scaffold Klebsiella pneumoniae WCHKP91 Klebsiella pneumoniae 1 Klebsiella pneumoniae 1 Klebsiella pneumoniae 1 NA

GCF_002164765 Scaffold Klebsiella pneumoniae WCHKP73 Klebsiella pneumoniae 1 Klebsiella pneumoniae 1 Klebsiella pneumoniae 1 NA

GCF_002164795 Scaffold Klebsiella pneumoniae WCHKP70 Klebsiella pneumoniae 1 Klebsiella pneumoniae 1 Klebsiella pneumoniae 1 NA

GCF_002164805 Complete Klebsiella variicola WCHKP19 Klebsiella variicola Klebsiella variicola Klebsiella variicola NA

GCF_002164835 Complete Klebsiella pneumoniae WCHKP2 Klebsiella pneumoniae 1 Klebsiella pneumoniae 1 Klebsiella pneumoniae 1 NA

GCF_002164855 Complete Klebsiella pneumoniae SCKP020003 Klebsiella pneumoniae 1 Klebsiella pneumoniae 1 Klebsiella pneumoniae 1 NA

GCF_002164875 Scaffold Klebsiella pneumoniae WCHKP10 Klebsiella pneumoniae 1 Klebsiella pneumoniae 1 Klebsiella pneumoniae 1 NA

GCF_002166915 Contig Klebsiella pneumoniae Kp-KL49 Klebsiella pneumoniae 1 Klebsiella pneumoniae 1 Klebsiella pneumoniae 1 NA

GCF_002166955 Contig Klebsiella pneumoniae CIV4 Klebsiella pneumoniae 1 Klebsiella pneumoniae 1 Klebsiella pneumoniae 1 NA

GCF_002166965 Contig Klebsiella pneumoniae KH43 Klebsiella pneumoniae 1 Klebsiella pneumoniae 1 Klebsiella pneumoniae 1 NA

GCF_002167025 Scaffold Klebsiella pneumoniae H151440672 Klebsiella pneumoniae 1 Klebsiella pneumoniae 1 Klebsiella pneumoniae 1 NA

GCF_002167035 Contig Klebsiella pneumoniae HH150820806-UK Klebsiella pneumoniae 1 Klebsiella pneumoniae 1 Klebsiella pneumoniae 1 NA

GCF_002167065 Contig Klebsiella pneumoniae H151300628-UK Klebsiella pneumoniae 1 Klebsiella pneumoniae 1 Klebsiella pneumoniae 1 NA

GCF_002167075 Scaffold Klebsiella pneumoniae H151400610 Klebsiella pneumoniae 1 Klebsiella pneumoniae 1 Klebsiella pneumoniae 1 NA

GCF_002167105 Contig Klebsiella pneumoniae H154440769 Klebsiella pneumoniae 1 Klebsiella pneumoniae 1 Klebsiella pneumoniae 1 NA

GCF_002167115 Scaffold Klebsiella pneumoniae H151400611 Klebsiella pneumoniae 1 Klebsiella pneumoniae 1 Klebsiella pneumoniae 1 NA

GCF_002167135 Contig Klebsiella pneumoniae H151440671 Klebsiella pneumoniae 1 Klebsiella pneumoniae 1 Klebsiella pneumoniae 1 NA

GCF_002167145 Contig Klebsiella pneumoniae H155360912 Klebsiella pneumoniae 1 Klebsiella pneumoniae 1 Klebsiella pneumoniae 1 NA

GCF_002173825 Contig Klebsiella pneumoniae 402 Klebsiella pneumoniae 1 Klebsiella pneumoniae 1 Klebsiella pneumoniae 1 NA

GCF_002173875 Contig Klebsiella pneumoniae 759 Klebsiella pneumoniae 1 Klebsiella pneumoniae 1 Klebsiella pneumoniae 1 NA

GCF_002173885 Contig Klebsiella pneumoniae 1479 Klebsiella pneumoniae 1 Klebsiella pneumoniae 1 Klebsiella pneumoniae 1 NA

GCF_002173905 Contig Klebsiella pneumoniae 337 Klebsiella pneumoniae 1 Klebsiella pneumoniae 1 Klebsiella pneumoniae 1 NA

GCF_002173955 Contig Klebsiella pneumoniae 1456 Klebsiella pneumoniae 1 Klebsiella pneumoniae 1 Klebsiella pneumoniae 1 NA

GCF_002173965 Contig Klebsiella pneumoniae 824 Klebsiella pneumoniae 1 Klebsiella pneumoniae 1 Klebsiella pneumoniae 1 NA

GCF_002174005 Contig Klebsiella pneumoniae H11 Klebsiella pneumoniae 1 Klebsiella pneumoniae 1 Klebsiella pneumoniae 1 NA

GCF_002174015 Contig Klebsiella pneumoniae 2045 Klebsiella pneumoniae 1 Klebsiella pneumoniae 1 Klebsiella pneumoniae 1 NA

GCF_002174025 Contig Klebsiella pneumoniae H18 Klebsiella pneumoniae 1 Klebsiella pneumoniae 1 Klebsiella pneumoniae 1 NA

GCF_002174075 Contig Klebsiella pneumoniae H15 Klebsiella pneumoniae 1 Klebsiella pneumoniae 1 Klebsiella pneumoniae 1 NA

GCF_002174085 Contig Klebsiella variicola H39 Klebsiella variicola Klebsiella variicola Klebsiella variicola NA

GCF_002174175 Contig Klebsiella pneumoniae 344 Klebsiella pneumoniae 1 Klebsiella pneumoniae 1 Klebsiella pneumoniae 1 NA

GCF_002179565 Contig Klebsiella variicola KP21F Klebsiella variicola Klebsiella variicola Klebsiella variicola NA

GCF_002180075 Complete Klebsiella pneumoniae AR0047 Klebsiella pneumoniae 1 Klebsiella pneumoniae 1 Klebsiella pneumoniae 1 NA

GCF_002180155 Complete Klebsiella pneumoniae AR0112 Klebsiella pneumoniae 1 Klebsiella pneumoniae 1 Klebsiella pneumoniae 1 NA

GCF_002180175 Complete Klebsiella pneumoniae AR0146 Klebsiella pneumoniae 1 Klebsiella pneumoniae 1 Klebsiella pneumoniae 1 NA

GCF_002180255 Complete Klebsiella pneumoniae AR0158 Klebsiella pneumoniae 1 Klebsiella pneumoniae 1 Klebsiella pneumoniae 1 NA

GCF_002180295 Complete Klebsiella pneumoniae AR0129 Klebsiella pneumoniae 1 Klebsiella pneumoniae 1 Klebsiella pneumoniae 1 NA

GCF_002180315 Complete Klebsiella pneumoniae AR0143 Klebsiella pneumoniae 1 Klebsiella pneumoniae 1 Klebsiella pneumoniae 1 NA

GCF_002180485 Contig Klebsiella pneumoniae 41949 Klebsiella pneumoniae 1 Klebsiella pneumoniae 1 Klebsiella pneumoniae 1 NA

GCF_002180495 Contig Klebsiella pneumoniae 39383 Klebsiella pneumoniae 1 Klebsiella pneumoniae 1 Klebsiella pneumoniae 1 NA

GCF_002180525 Contig Klebsiella pneumoniae 41947 Klebsiella pneumoniae 1 Klebsiella pneumoniae 1 Klebsiella pneumoniae 1 NA

GCF_002180535 Contig Klebsiella pneumoniae 39385 Klebsiella pneumoniae 1 Klebsiella pneumoniae 1 Klebsiella pneumoniae 1 NA

GCF_002180565 Contig Klebsiella pneumoniae 39428 Klebsiella pneumoniae 1 Klebsiella pneumoniae 1 Klebsiella pneumoniae 1 NA

GCF_002180605 Contig Klebsiella pneumoniae 39865 Klebsiella pneumoniae 1 Klebsiella pneumoniae 1 Klebsiella pneumoniae 1 NA

GCF_002180645 Contig Klebsiella pneumoniae 39879 Klebsiella pneumoniae 1 Klebsiella pneumoniae 1 Klebsiella pneumoniae 1 NA

GCF_002180655 Contig Klebsiella pneumoniae 39887 Klebsiella pneumoniae 1 Klebsiella pneumoniae 1 Klebsiella pneumoniae 1 NA

GCF_002180685 Contig Klebsiella pneumoniae 39897 Klebsiella pneumoniae 1 Klebsiella pneumoniae 1 Klebsiella pneumoniae 1 NA

GCF_002180695 Contig Klebsiella pneumoniae 39901 Klebsiella pneumoniae 1 Klebsiella pneumoniae 1 Klebsiella pneumoniae 1 NA

GCF_002180725 Contig Klebsiella pneumoniae 39902 Klebsiella pneumoniae 1 Klebsiella pneumoniae 1 Klebsiella pneumoniae 1 NA

GCF_002180735 Contig Klebsiella pneumoniae 39903 Klebsiella pneumoniae 1 Klebsiella pneumoniae 1 Klebsiella pneumoniae 1 NA

GCF_002180765 Contig Klebsiella pneumoniae 39910 Klebsiella pneumoniae 1 Klebsiella pneumoniae 1 Klebsiella pneumoniae 1 NA

GCF_002180775 Contig Klebsiella pneumoniae 39908 Klebsiella pneumoniae 1 Klebsiella pneumoniae 1 Klebsiella pneumoniae 1 NA

GCF_002180805 Contig Klebsiella pneumoniae 39911 Klebsiella pneumoniae 1 Klebsiella pneumoniae 1 Klebsiella pneumoniae 1 NA

GCF_002180845 Contig Klebsiella pneumoniae 40886 Klebsiella pneumoniae 1 Klebsiella pneumoniae 1 Klebsiella pneumoniae 1 NA

GCF_002180855 Contig Klebsiella pneumoniae 40862 Klebsiella pneumoniae 1 Klebsiella pneumoniae 1 Klebsiella pneumoniae 1 NA

GCF_002180885 Contig Klebsiella pneumoniae 40888 Klebsiella pneumoniae 1 Klebsiella pneumoniae 1 Klebsiella pneumoniae 1 NA

GCF_002180895 Contig Klebsiella pneumoniae 44829 Klebsiella pneumoniae 1 Klebsiella pneumoniae 1 Klebsiella pneumoniae 1 NA

GCF_002180925 Contig Klebsiella pneumoniae 44830 Klebsiella pneumoniae 1 Klebsiella pneumoniae 1 Klebsiella pneumoniae 1 NA

GCF_002180935 Contig Klebsiella pneumoniae 44831 Klebsiella pneumoniae 1 Klebsiella pneumoniae 1 Klebsiella pneumoniae 1 NA

GCF_002180965 Contig Klebsiella pneumoniae 45705 Klebsiella pneumoniae 1 Klebsiella pneumoniae 1 Klebsiella pneumoniae 1 NA

GCF_002181005 Contig Klebsiella pneumoniae 42810 Klebsiella pneumoniae 1 Klebsiella pneumoniae 1 Klebsiella pneumoniae 1 NA

GCF_002181015 Contig Klebsiella pneumoniae 45708 Klebsiella pneumoniae 1 Klebsiella pneumoniae 1 Klebsiella pneumoniae 1 NA

GCF_002181045 Contig Klebsiella pneumoniae 42812 Klebsiella pneumoniae 1 Klebsiella pneumoniae 1 Klebsiella pneumoniae 1 NA

GCF_002181055 Contig Klebsiella pneumoniae 42811 Klebsiella pneumoniae 1 Klebsiella pneumoniae 1 Klebsiella pneumoniae 1 NA

GCF_002181085 Contig Klebsiella pneumoniae 42814 Klebsiella pneumoniae 1 Klebsiella pneumoniae 1 Klebsiella pneumoniae 1 NA

GCF_002181095 Contig Klebsiella pneumoniae 42859 Klebsiella pneumoniae 1 Klebsiella pneumoniae 1 Klebsiella pneumoniae 1 NA

GCF_002181105 Contig Klebsiella pneumoniae 43328 Klebsiella pneumoniae 1 Klebsiella pneumoniae 1 Klebsiella pneumoniae 1 NA

GCF_002181145 Contig Klebsiella pneumoniae 43329 Klebsiella pneumoniae 1 Klebsiella pneumoniae 1 Klebsiella pneumoniae 1 NA

GCF_002181165 Contig Klebsiella pneumoniae 43331 Klebsiella pneumoniae 1 Klebsiella pneumoniae 1 Klebsiella pneumoniae 1 NA

GCF_002181175 Contig Klebsiella pneumoniae 43422 Klebsiella pneumoniae 1 Klebsiella pneumoniae 1 Klebsiella pneumoniae 1 NA

GCF_002181185 Contig Klebsiella pneumoniae 44808 Klebsiella pneumoniae 1 Klebsiella pneumoniae 1 Klebsiella pneumoniae 1 NA

GCF_002181225 Contig Klebsiella pneumoniae 44809 Klebsiella pneumoniae 1 Klebsiella pneumoniae 1 Klebsiella pneumoniae 1 NA

GCF_002181245 Contig Klebsiella pneumoniae 44811 Klebsiella pneumoniae 1 Klebsiella pneumoniae 1 Klebsiella pneumoniae 1 NA

GCF_002181265 Contig Klebsiella pneumoniae 44810 Klebsiella pneumoniae 1 Klebsiella pneumoniae 1 Klebsiella pneumoniae 1 NA

GCF_002181275 Contig Klebsiella pneumoniae 44813 Klebsiella pneumoniae 1 Klebsiella pneumoniae 1 Klebsiella pneumoniae 1 NA

GCF_002181305 Contig Klebsiella pneumoniae 44814 Klebsiella pneumoniae 1 Klebsiella pneumoniae 1 Klebsiella pneumoniae 1 NA

GCF_002181325 Contig Klebsiella pneumoniae 44815 Klebsiella pneumoniae 1 Klebsiella pneumoniae 1 Klebsiella pneumoniae 1 NA

GCF_002181345 Contig Klebsiella pneumoniae 44816 Klebsiella pneumoniae 1 Klebsiella pneumoniae 1 Klebsiella pneumoniae 1 NA

GCF_002181355 Contig Klebsiella pneumoniae 44817 Klebsiella pneumoniae 1 Klebsiella pneumoniae 1 Klebsiella pneumoniae 1 NA

GCF_002181385 Contig Klebsiella pneumoniae 44818 Klebsiella pneumoniae 1 Klebsiella pneumoniae 1 Klebsiella pneumoniae 1 NA

GCF_002181405 Contig Klebsiella pneumoniae 44819 Klebsiella pneumoniae 1 Klebsiella pneumoniae 1 Klebsiella pneumoniae 1 NA

GCF_002181415 Contig Klebsiella pneumoniae 44820 Klebsiella pneumoniae 1 Klebsiella pneumoniae 1 Klebsiella pneumoniae 1 NA

GCF_002181445 Contig Klebsiella pneumoniae 44824 Klebsiella pneumoniae 1 Klebsiella pneumoniae 1 Klebsiella pneumoniae 1 NA

GCF_002181455 Contig Klebsiella pneumoniae 41964 Klebsiella pneumoniae 1 Klebsiella pneumoniae 1 Klebsiella pneumoniae 1 NA

GCF_002181485 Contig Klebsiella pneumoniae 41965 Klebsiella pneumoniae 1 Klebsiella pneumoniae 1 Klebsiella pneumoniae 1 NA

GCF_002181505 Contig Klebsiella pneumoniae 41966 Klebsiella pneumoniae 1 Klebsiella pneumoniae 1 Klebsiella pneumoniae 1 NA

GCF_002181525 Contig Klebsiella pneumoniae 41950 Klebsiella pneumoniae 1 Klebsiella pneumoniae 1 Klebsiella pneumoniae 1 NA

GCF_002181545 Contig Klebsiella pneumoniae 41959 Klebsiella pneumoniae 1 Klebsiella pneumoniae 1 Klebsiella pneumoniae 1 NA

GCF_002181555 Contig Klebsiella pneumoniae 41963 Klebsiella pneumoniae 1 Klebsiella pneumoniae 1 Klebsiella pneumoniae 1 NA

GCF_002181585 Contig Klebsiella pneumoniae 39883 Klebsiella pneumoniae 1 Klebsiella pneumoniae 1 Klebsiella pneumoniae 1 NA

GCF_002181595 Contig Klebsiella pneumoniae 39885 Klebsiella pneumoniae 1 Klebsiella pneumoniae 1 Klebsiella pneumoniae 1 NA

GCF_002181635 Contig Klebsiella pneumoniae 39904 Klebsiella pneumoniae 1 Klebsiella pneumoniae 1 Klebsiella pneumoniae 1 NA

GCF_002181665 Contig Klebsiella pneumoniae 39909 Klebsiella pneumoniae 1 Klebsiella pneumoniae 1 Klebsiella pneumoniae 1 NA

GCF_002181675 Contig Klebsiella pneumoniae 40882 Klebsiella pneumoniae 1 Klebsiella pneumoniae 1 Klebsiella pneumoniae 1 NA

GCF_002181705 Contig Klebsiella pneumoniae 44825 Klebsiella pneumoniae 1 Klebsiella pneumoniae 1 Klebsiella pneumoniae 1 NA

GCF_002181715 Contig Klebsiella pneumoniae 44826 Klebsiella pneumoniae 1 Klebsiella pneumoniae 1 Klebsiella pneumoniae 1 NA

GCF_002181745 Contig Klebsiella pneumoniae 44827 Klebsiella pneumoniae 1 Klebsiella pneumoniae 1 Klebsiella pneumoniae 1 NA

GCF_002181755 Contig Klebsiella pneumoniae 44828 Klebsiella pneumoniae 1 Klebsiella pneumoniae 1 Klebsiella pneumoniae 1 NA

GCF_002181785 Contig Klebsiella pneumoniae 45704 Klebsiella pneumoniae 1 Klebsiella pneumoniae 1 Klebsiella pneumoniae 1 NA

GCF_002181795 Contig Klebsiella pneumoniae 45707 Klebsiella pneumoniae 1 Klebsiella pneumoniae 1 Klebsiella pneumoniae 1 NA

GCF_002181825 Contig Klebsiella pneumoniae 43416 Klebsiella pneumoniae 1 Klebsiella pneumoniae 1 Klebsiella pneumoniae 1 NA

GCF_002181835 Contig Klebsiella pneumoniae 45709 Klebsiella pneumoniae 1 Klebsiella pneumoniae 1 Klebsiella pneumoniae 1 NA

GCF_002181865 Contig Klebsiella pneumoniae 44821 Klebsiella pneumoniae 1 Klebsiella pneumoniae 1 Klebsiella pneumoniae 1 NA

GCF_002181875 Contig Klebsiella pneumoniae 43420 Klebsiella pneumoniae 1 Klebsiella pneumoniae 1 Klebsiella pneumoniae 1 NA

GCF_002181895 Contig Klebsiella pneumoniae 44822 Klebsiella pneumoniae 1 Klebsiella pneumoniae 1 Klebsiella pneumoniae 1 NA

GCF_002181915 Contig Klebsiella pneumoniae 43419 Klebsiella pneumoniae 1 Klebsiella pneumoniae 1 Klebsiella pneumoniae 1 NA

GCF_002181945 Contig Klebsiella pneumoniae 44812 Klebsiella pneumoniae 1 Klebsiella pneumoniae 1 Klebsiella pneumoniae 1 NA

GCF_002181955 Contig Klebsiella pneumoniae 43421 Klebsiella pneumoniae 1 Klebsiella pneumoniae 1 Klebsiella pneumoniae 1 NA

GCF_002181965 Contig Klebsiella pneumoniae 44823 Klebsiella pneumoniae 1 Klebsiella pneumoniae 1 Klebsiella pneumoniae 1 NA

GCF_002182005 Contig Klebsiella pneumoniae 42184 Klebsiella pneumoniae 1 Klebsiella pneumoniae 1 Klebsiella pneumoniae 1 NA

GCF_002182045 Contig Klebsiella pneumoniae 42194 Klebsiella pneumoniae 1 Klebsiella pneumoniae 1 Klebsiella pneumoniae 1 NA

GCF_002182055 Contig Klebsiella pneumoniae 42196 Klebsiella pneumoniae 1 Klebsiella pneumoniae 1 Klebsiella pneumoniae 1 NA

GCF_002182065 Contig Klebsiella pneumoniae 42188 Klebsiella pneumoniae 1 Klebsiella pneumoniae 1 Klebsiella pneumoniae 1 NA

GCF_002182135 Contig Klebsiella pneumoniae 42261 Klebsiella pneumoniae 1 Klebsiella pneumoniae 1 Klebsiella pneumoniae 1 NA

GCF_002182145 Contig Klebsiella pneumoniae 42339 Klebsiella pneumoniae 1 Klebsiella pneumoniae 1 Klebsiella pneumoniae 1 NA

GCF_002182155 Contig Klebsiella pneumoniae 42363 Klebsiella pneumoniae 1 Klebsiella pneumoniae 1 Klebsiella pneumoniae 1 NA

GCF_002182205 Contig Klebsiella pneumoniae 42364 Klebsiella pneumoniae 1 Klebsiella pneumoniae 1 Klebsiella pneumoniae 1 NA

GCF_002182215 Contig Klebsiella pneumoniae 42807 Klebsiella pneumoniae 1 Klebsiella pneumoniae 1 Klebsiella pneumoniae 1 NA

GCF_002182235 Contig Klebsiella pneumoniae 42365 Klebsiella pneumoniae 1 Klebsiella pneumoniae 1 Klebsiella pneumoniae 1 NA

GCF_002182245 Contig Klebsiella pneumoniae 42809 Klebsiella pneumoniae 1 Klebsiella pneumoniae 1 Klebsiella pneumoniae 1 NA

GCF_002182285 Contig Klebsiella pneumoniae 45710 Klebsiella pneumoniae 1 Klebsiella pneumoniae 1 Klebsiella pneumoniae 1 NA

GCF_002182325 Contig Klebsiella pneumoniae 42808 Klebsiella pneumoniae 1 Klebsiella pneumoniae 1 Klebsiella pneumoniae 1 NA

GCF_002182525 Contig Klebsiella pneumoniae 45712 Klebsiella pneumoniae 1 Klebsiella pneumoniae 1 Klebsiella pneumoniae 1 NA

GCF_002182535 Contig Klebsiella pneumoniae 42186 Klebsiella pneumoniae 1 Klebsiella pneumoniae 1 Klebsiella pneumoniae 1 NA

GCF_002182575 Contig Klebsiella pneumoniae 42195 Klebsiella pneumoniae 1 Klebsiella pneumoniae 1 Klebsiella pneumoniae 1 NA

GCF_002184115 Contig Klebsiella pneumoniae 39387 Klebsiella pneumoniae 1 Klebsiella pneumoniae 1 Klebsiella pneumoniae 1 NA

GCF_002184225 Contig Klebsiella pneumoniae 45711 Klebsiella pneumoniae 1 Klebsiella pneumoniae 1 Klebsiella pneumoniae 1 NA

GCF_002184395 Contig Klebsiella pneumoniae CRK0010 Klebsiella pneumoniae 1 Klebsiella pneumoniae 1 Klebsiella pneumoniae 1 NA

GCF_002184415 Contig Klebsiella pneumoniae CRK0009 Klebsiella pneumoniae 1 Klebsiella pneumoniae 1 Klebsiella pneumoniae 1 NA

GCF_002184485 Contig Klebsiella pneumoniae CRK0011 Klebsiella pneumoniae 1 Klebsiella pneumoniae 1 Klebsiella pneumoniae 1 NA

GCF_002184505 Contig Klebsiella pneumoniae CRK0007 Klebsiella pneumoniae 1 Klebsiella pneumoniae 1 Klebsiella pneumoniae 1 NA

GCF_002184515 Contig Klebsiella pneumoniae CRK0008 Klebsiella pneumoniae 1 Klebsiella pneumoniae 1 Klebsiella pneumoniae 1 NA

GCF_002184525 Contig Klebsiella pneumoniae CRK0004 Klebsiella pneumoniae 1 Klebsiella pneumoniae 1 Klebsiella pneumoniae 1 NA

GCF_002184575 Contig Klebsiella aerogenes CRK0059 Klebsiella aerogenes 1 Klebsiella aerogenes Klebsiella aerogenes NA

GCF_002184625 Contig Klebsiella aerogenes CRK0057 Klebsiella aerogenes 1 Klebsiella aerogenes Klebsiella aerogenes NA

GCF_002184705 Contig Klebsiella pneumoniae CRK0039 Klebsiella pneumoniae 1 Klebsiella pneumoniae 1 Klebsiella pneumoniae 1 NA

GCF_002184715 Contig Klebsiella pneumoniae CRK0037 Klebsiella pneumoniae 1 Klebsiella pneumoniae 1 Klebsiella pneumoniae 1 NA

GCF_002184745 Contig Klebsiella pneumoniae CRK0028 Klebsiella pneumoniae 1 Klebsiella pneumoniae 1 Klebsiella pneumoniae 1 NA

GCF_002184755 Contig Klebsiella pneumoniae CRK0026 Klebsiella pneumoniae 1 Klebsiella pneumoniae 1 Klebsiella pneumoniae 1 NA

GCF_002184785 Contig Klebsiella pneumoniae CRK0022 Klebsiella pneumoniae 1 Klebsiella pneumoniae 1 Klebsiella pneumoniae 1 NA

GCF_002184795 Contig Klebsiella pneumoniae CRK0021 Klebsiella pneumoniae 1 Klebsiella pneumoniae 1 Klebsiella pneumoniae 1 NA

GCF_002184825 Contig Klebsiella pneumoniae CRK0020 Klebsiella pneumoniae 1 Klebsiella pneumoniae 1 Klebsiella pneumoniae 1 NA

GCF_002184835 Contig Klebsiella pneumoniae CRK0018 Klebsiella pneumoniae 1 Klebsiella pneumoniae 1 Klebsiella pneumoniae 1 NA

GCF_002184865 Contig Klebsiella pneumoniae CRK0016 Klebsiella pneumoniae 1 Klebsiella pneumoniae 1 Klebsiella pneumoniae 1 NA

GCF_002184875 Contig Klebsiella pneumoniae CRK0015 Klebsiella pneumoniae 1 Klebsiella pneumoniae 1 Klebsiella pneumoniae 1 NA

GCF_002184905 Contig Klebsiella quasipneumoniae quasipneumoniae CRK0052 Klebsiella quasipneumoniae 2 Klebsiella quasipneumoniae 2 Klebsiella quasipneumoniae NA

GCF_002184915 Contig Klebsiella pneumoniae CRK0014 Klebsiella pneumoniae 1 Klebsiella pneumoniae 1 Klebsiella pneumoniae 1 NA

GCF_002184945 Contig Klebsiella pneumoniae CRK0045 Klebsiella pneumoniae 1 Klebsiella pneumoniae 1 Klebsiella pneumoniae 1 NA

GCF_002184955 Contig Klebsiella pneumoniae CRK0050 Klebsiella pneumoniae 1 Klebsiella pneumoniae 1 Klebsiella pneumoniae 1 NA

GCF_002184985 Contig Klebsiella pneumoniae CRK0085 Klebsiella pneumoniae 1 Klebsiella pneumoniae 1 Klebsiella pneumoniae 1 NA

GCF_002184995 Contig Klebsiella pneumoniae CRK0079 Klebsiella pneumoniae 1 Klebsiella pneumoniae 1 Klebsiella pneumoniae 1 NA

GCF_002185025 Contig Klebsiella pneumoniae CRK0076 Klebsiella pneumoniae 1 Klebsiella pneumoniae 1 Klebsiella pneumoniae 1 NA

GCF_002185045 Contig Klebsiella pneumoniae CRK0075 Klebsiella pneumoniae 1 Klebsiella pneumoniae 1 Klebsiella pneumoniae 1 NA

GCF_002185055 Contig Klebsiella pneumoniae CRK0073 Klebsiella pneumoniae 1 Klebsiella pneumoniae 1 Klebsiella pneumoniae 1 NA

GCF_002185075 Contig Klebsiella pneumoniae CRK0066 Klebsiella pneumoniae 1 Klebsiella pneumoniae 1 Klebsiella pneumoniae 1 NA

GCF_002185105 Contig Klebsiella pneumoniae CRK0062 Klebsiella pneumoniae 1 Klebsiella pneumoniae 1 Klebsiella pneumoniae 1 NA

GCF_002185125 Contig Klebsiella pneumoniae CRK0061 Klebsiella pneumoniae 1 Klebsiella pneumoniae 1 Klebsiella pneumoniae 1 NA

GCF_002185135 Contig Klebsiella pneumoniae CRK0101 Klebsiella pneumoniae 1 Klebsiella pneumoniae 1 Klebsiella pneumoniae 1 NA

GCF_002185155 Contig Klebsiella pneumoniae CRK0100 Klebsiella pneumoniae 1 Klebsiella pneumoniae 1 Klebsiella pneumoniae 1 NA

GCF_002185185 Contig Klebsiella pneumoniae CRK0097 Klebsiella pneumoniae 1 Klebsiella pneumoniae 1 Klebsiella pneumoniae 1 NA

GCF_002185205 Contig Klebsiella pneumoniae CRK0093 Klebsiella pneumoniae 1 Klebsiella pneumoniae 1 Klebsiella pneumoniae 1 NA

GCF_002185215 Contig Klebsiella pneumoniae CRK0086 Klebsiella pneumoniae 1 Klebsiella pneumoniae 1 Klebsiella pneumoniae 1 NA

GCF_002185285 Contig Klebsiella pneumoniae CRK0038 Klebsiella pneumoniae 1 Klebsiella pneumoniae 1 Klebsiella pneumoniae 1 NA

GCF_002185345 Contig Klebsiella aerogenes CRK0055 Klebsiella aerogenes 2 Klebsiella aerogenes Klebsiella aerogenes NA

GCF_002185355 Contig Klebsiella pneumoniae CRK0036 Klebsiella pneumoniae 1 Klebsiella pneumoniae 1 Klebsiella pneumoniae 1 NA

GCF_002185385 Contig Klebsiella pneumoniae CRK0032 Klebsiella pneumoniae 1 Klebsiella pneumoniae 1 Klebsiella pneumoniae 1 NA

GCF_002185405 Contig Klebsiella pneumoniae CRK0030 Klebsiella pneumoniae 1 Klebsiella pneumoniae 1 Klebsiella pneumoniae 1 NA

GCF_002185415 Contig Klebsiella pneumoniae CRK0023 Klebsiella pneumoniae 1 Klebsiella pneumoniae 1 Klebsiella pneumoniae 1 NA

GCF_002185425 Contig Klebsiella pneumoniae CRK0024 Klebsiella pneumoniae 1 Klebsiella pneumoniae 1 Klebsiella pneumoniae 1 NA

GCF_002185465 Contig Klebsiella pneumoniae CRK0013 Klebsiella pneumoniae 1 Klebsiella pneumoniae 1 Klebsiella pneumoniae 1 NA

GCF_002185485 Contig Klebsiella pneumoniae CRK0053 Klebsiella pneumoniae 1 Klebsiella pneumoniae 1 Klebsiella pneumoniae 1 NA

GCF_002185495 Contig Klebsiella pneumoniae CRK0051 Klebsiella pneumoniae 1 Klebsiella pneumoniae 1 Klebsiella pneumoniae 1 NA

GCF_002185515 Contig Klebsiella pneumoniae CRK0048 Klebsiella pneumoniae 1 Klebsiella pneumoniae 1 Klebsiella pneumoniae 1 NA

GCF_002185545 Contig Klebsiella pneumoniae CRK0046 Klebsiella pneumoniae 1 Klebsiella pneumoniae 1 Klebsiella pneumoniae 1 NA

GCF_002185555 Contig Klebsiella pneumoniae CRK0044 Klebsiella pneumoniae 1 Klebsiella pneumoniae 1 Klebsiella pneumoniae 1 NA

GCF_002185565 Contig Klebsiella pneumoniae CRK0042 Klebsiella pneumoniae 1 Klebsiella pneumoniae 1 Klebsiella pneumoniae 1 NA

GCF_002185575 Contig Klebsiella pneumoniae CRK0080 Klebsiella pneumoniae 1 Klebsiella pneumoniae 1 Klebsiella pneumoniae 1 NA

GCF_002185625 Contig Klebsiella pneumoniae CRK0069 Klebsiella pneumoniae 1 Klebsiella pneumoniae 1 Klebsiella pneumoniae 1 NA

GCF_002185645 Contig Klebsiella pneumoniae CRK0077 Klebsiella pneumoniae 1 Klebsiella pneumoniae 1 Klebsiella pneumoniae 1 NA

GCF_002185655 Contig Klebsiella pneumoniae CRK0065 Klebsiella pneumoniae 1 Klebsiella pneumoniae 1 Klebsiella pneumoniae 1 NA

GCF_002185665 Contig Klebsiella pneumoniae CRK0070 Klebsiella pneumoniae 1 Klebsiella pneumoniae 1 Klebsiella pneumoniae 1 NA

GCF_002185705 Contig Klebsiella pneumoniae CRK0063 Klebsiella pneumoniae 1 Klebsiella pneumoniae 1 Klebsiella pneumoniae 1 NA

GCF_002185725 Contig Klebsiella pneumoniae CRK0095 Klebsiella pneumoniae 1 Klebsiella pneumoniae 1 Klebsiella pneumoniae 1 NA

GCF_002185735 Contig Klebsiella pneumoniae CRK0094 Klebsiella pneumoniae 1 Klebsiella pneumoniae 1 Klebsiella pneumoniae 1 NA

GCF_002185745 Contig Klebsiella pneumoniae CRK0090 Klebsiella pneumoniae 1 Klebsiella pneumoniae 1 Klebsiella pneumoniae 1 NA

GCF_002185755 Contig Klebsiella pneumoniae CRK0089 Klebsiella pneumoniae 1 Klebsiella pneumoniae 1 Klebsiella pneumoniae 1 NA

GCF_002185805 Contig Klebsiella pneumoniae CRK0088 Klebsiella pneumoniae 1 Klebsiella pneumoniae 1 Klebsiella pneumoniae 1 NA

GCF_002185815 Contig Klebsiella pneumoniae CRK0040 Klebsiella pneumoniae 1 Klebsiella pneumoniae 1 Klebsiella pneumoniae 1 NA

GCF_002185835 Contig Klebsiella pneumoniae CRK0087 Klebsiella pneumoniae 1 Klebsiella pneumoniae 1 Klebsiella pneumoniae 1 NA

GCF_002185885 Contig Klebsiella pneumoniae CRK0035 Klebsiella pneumoniae 1 Klebsiella pneumoniae 1 Klebsiella pneumoniae 1 NA

GCF_002185905 Contig Klebsiella pneumoniae CRK0034 Klebsiella pneumoniae 1 Klebsiella pneumoniae 1 Klebsiella pneumoniae 1 NA

GCF_002185915 Contig Klebsiella pneumoniae CRK0031 Klebsiella pneumoniae 1 Klebsiella pneumoniae 1 Klebsiella pneumoniae 1 NA

GCF_002185925 Contig Klebsiella pneumoniae CRK0033 Klebsiella pneumoniae 1 Klebsiella pneumoniae 1 Klebsiella pneumoniae 1 NA

GCF_002185965 Contig Klebsiella pneumoniae CRK0029 Klebsiella pneumoniae 1 Klebsiella pneumoniae 1 Klebsiella pneumoniae 1 NA

GCF_002185975 Contig Klebsiella pneumoniae CRK0025 Klebsiella pneumoniae 1 Klebsiella pneumoniae 1 Klebsiella pneumoniae 1 NA

GCF_002186005 Contig Klebsiella pneumoniae CRK0019 Klebsiella pneumoniae 1 Klebsiella pneumoniae 1 Klebsiella pneumoniae 1 NA

GCF_002186015 Contig Klebsiella pneumoniae CRK0017 Klebsiella pneumoniae 1 Klebsiella pneumoniae 1 Klebsiella pneumoniae 1 NA

GCF_002186045 Contig Klebsiella pneumoniae CRK0049 Klebsiella pneumoniae 1 Klebsiella pneumoniae 1 Klebsiella pneumoniae 1 NA

GCF_002186055 Contig Klebsiella pneumoniae CRK0012 Klebsiella pneumoniae 1 Klebsiella pneumoniae 1 Klebsiella pneumoniae 1 NA

GCF_002186085 Contig Klebsiella pneumoniae CRK0047 Klebsiella pneumoniae 1 Klebsiella pneumoniae 1 Klebsiella pneumoniae 1 NA

GCF_002186095 Contig Klebsiella pneumoniae CRK0043 Klebsiella pneumoniae 1 Klebsiella pneumoniae 1 Klebsiella pneumoniae 1 NA

GCF_002186125 Contig Klebsiella pneumoniae CRK0083 Klebsiella pneumoniae 1 Klebsiella pneumoniae 1 Klebsiella pneumoniae 1 NA

GCF_002186135 Contig Klebsiella pneumoniae CRK0084 Klebsiella pneumoniae 1 Klebsiella pneumoniae 1 Klebsiella pneumoniae 1 NA

GCF_002186165 Contig Klebsiella pneumoniae CRK0082 Klebsiella pneumoniae 1 Klebsiella pneumoniae 1 Klebsiella pneumoniae 1 NA

GCF_002186185 Contig Klebsiella pneumoniae CRK0081 Klebsiella pneumoniae 1 Klebsiella pneumoniae 1 Klebsiella pneumoniae 1 NA

GCF_002186205 Contig Klebsiella pneumoniae CRK0074 Klebsiella pneumoniae 1 Klebsiella pneumoniae 1 Klebsiella pneumoniae 1 NA

GCF_002186215 Contig Klebsiella pneumoniae CRK0078 Klebsiella pneumoniae 1 Klebsiella pneumoniae 1 Klebsiella pneumoniae 1 NA

GCF_002186245 Contig Klebsiella pneumoniae CRK0071 Klebsiella pneumoniae 1 Klebsiella pneumoniae 1 Klebsiella pneumoniae 1 NA

GCF_002186255 Contig Klebsiella pneumoniae CRK0072 Klebsiella pneumoniae 1 Klebsiella pneumoniae 1 Klebsiella pneumoniae 1 NA

GCF_002186265 Contig Klebsiella pneumoniae CRK0068 Klebsiella pneumoniae 1 Klebsiella pneumoniae 1 Klebsiella pneumoniae 1 NA

GCF_002186275 Contig Klebsiella pneumoniae CRK0067 Klebsiella pneumoniae 1 Klebsiella pneumoniae 1 Klebsiella pneumoniae 1 NA

GCF_002186325 Contig Klebsiella pneumoniae CRK0064 Klebsiella pneumoniae 1 Klebsiella pneumoniae 1 Klebsiella pneumoniae 1 NA

GCF_002186335 Contig Klebsiella pneumoniae CRK0098 Klebsiella pneumoniae 1 Klebsiella pneumoniae 1 Klebsiella pneumoniae 1 NA

GCF_002186365 Contig Klebsiella pneumoniae CRK0099 Klebsiella pneumoniae 1 Klebsiella pneumoniae 1 Klebsiella pneumoniae 1 NA

GCF_002186375 Contig Klebsiella pneumoniae CRK0096 Klebsiella pneumoniae 1 Klebsiella pneumoniae 1 Klebsiella pneumoniae 1 NA

GCF_002186405 Contig Klebsiella pneumoniae CRK0091 Klebsiella pneumoniae 1 Klebsiella pneumoniae 1 Klebsiella pneumoniae 1 NA

GCF_002186415 Contig Klebsiella pneumoniae CRK0092 Klebsiella pneumoniae 1 Klebsiella pneumoniae 1 Klebsiella pneumoniae 1 NA

GCF_002186435 Contig Klebsiella pneumoniae UNMC8078 Klebsiella pneumoniae 1 Klebsiella pneumoniae 1 Klebsiella pneumoniae 1 NA

GCF_002186465 Contig Klebsiella pneumoniae UNMC7905 Klebsiella pneumoniae 1 Klebsiella pneumoniae 1 Klebsiella pneumoniae 1 NA

GCF_002186485 Contig Klebsiella quasipneumoniae similipneumoniae UNMC7493 Klebsiella quasipneumoniae 1 Klebsiella quasipneumoniae 1 Klebsiella quasipneumoniae NA

GCF_002186505 Contig Klebsiella pneumoniae PO967 Klebsiella pneumoniae 1 Klebsiella pneumoniae 1 Klebsiella pneumoniae 1 NA

GCF_002186545 Contig Klebsiella pneumoniae PO450 Klebsiella pneumoniae 1 Klebsiella pneumoniae 1 Klebsiella pneumoniae 1 NA

GCF_002186565 Contig Klebsiella quasipneumoniae similipneumoniae PO416 Klebsiella quasipneumoniae 1 Klebsiella quasipneumoniae 1 Klebsiella quasipneumoniae NA

GCF_002186585 Contig Klebsiella pneumoniae PO633 Klebsiella pneumoniae 1 Klebsiella pneumoniae 1 Klebsiella pneumoniae 1 NA

GCF_002186595 Contig Klebsiella pneumoniae PO906 Klebsiella pneumoniae 1 Klebsiella pneumoniae 1 Klebsiella pneumoniae 1 NA

GCF_002186625 Contig Klebsiella pneumoniae PO613 Klebsiella pneumoniae 1 Klebsiella pneumoniae 1 Klebsiella pneumoniae 1 NA

GCF_002186635 Contig Klebsiella pneumoniae PO457 Klebsiella pneumoniae 1 Klebsiella pneumoniae 1 Klebsiella pneumoniae 1 NA

GCF_002186665 Contig Klebsiella pneumoniae MM2436 Klebsiella pneumoniae 1 Klebsiella pneumoniae 1 Klebsiella pneumoniae 1 NA

GCF_002186695 Contig Klebsiella pneumoniae PO2673 Klebsiella pneumoniae 1 Klebsiella pneumoniae 1 Klebsiella pneumoniae 1 NA

GCF_002186705 Contig Klebsiella pneumoniae PO379 Klebsiella pneumoniae 1 Klebsiella pneumoniae 1 Klebsiella pneumoniae 1 NA

GCF_002186725 Contig Klebsiella pneumoniae PO343 Klebsiella pneumoniae 1 Klebsiella pneumoniae 1 Klebsiella pneumoniae 1 NA

GCF_002186735 Contig Klebsiella michiganensis PO2731 Klebsiella pasteurii Klebsiella pasteurii Klebsiella grimontii NA

GCF_002186775 Contig Klebsiella pneumoniae PO2429 Klebsiella pneumoniae 1 Klebsiella pneumoniae 1 Klebsiella pneumoniae 1 NA

GCF_002186785 Contig Klebsiella pneumoniae PO1932 Klebsiella pneumoniae 1 Klebsiella pneumoniae 1 Klebsiella pneumoniae 1 NA

GCF_002186805 Contig Klebsiella pneumoniae PO1805 Klebsiella pneumoniae 1 Klebsiella pneumoniae 1 Klebsiella pneumoniae 1 NA

GCF_002186835 Contig Klebsiella pneumoniae PO1842 Klebsiella pneumoniae 1 Klebsiella pneumoniae 1 Klebsiella pneumoniae 1 NA

GCF_002186855 Contig Klebsiella pneumoniae PO1659 Klebsiella pneumoniae 1 Klebsiella pneumoniae 1 Klebsiella pneumoniae 1 NA

GCF_002186865 Contig Klebsiella pneumoniae PO1516 Klebsiella pneumoniae 1 Klebsiella pneumoniae 1 Klebsiella pneumoniae 1 NA

GCF_002186895 Contig Klebsiella pneumoniae PO1380 Klebsiella pneumoniae 1 Klebsiella pneumoniae 1 Klebsiella pneumoniae 1 NA

GCF_002186915 Contig Klebsiella quasipneumoniae quasipneumoniae PO1285 Klebsiella quasipneumoniae 2 Klebsiella quasipneumoniae 2 Klebsiella quasipneumoniae NA

GCF_002186935 Contig Klebsiella pneumoniae MM MK620 Klebsiella pneumoniae 1 Klebsiella pneumoniae 1 Klebsiella pneumoniae 1 NA

GCF_002186945 Contig Klebsiella pneumoniae PO1238 Klebsiella pneumoniae 1 Klebsiella pneumoniae 1 Klebsiella pneumoniae 1 NA

GCF_002186965 Contig Klebsiella quasipneumoniae similipneumoniae MM2867 Klebsiella quasipneumoniae 1 Klebsiella quasipneumoniae 1 Klebsiella quasipneumoniae NA

GCF_002186995 Contig Klebsiella pneumoniae HB Z2591 Klebsiella pneumoniae 1 Klebsiella pneumoniae 1 Klebsiella pneumoniae 1 NA

GCF_002187015 Contig Klebsiella pneumoniae HB2044 Klebsiella pneumoniae 1 Klebsiella pneumoniae 1 Klebsiella pneumoniae 1 NA

GCF_002187025 Contig Klebsiella pneumoniae G749 Klebsiella pneumoniae 1 Klebsiella pneumoniae 1 Klebsiella pneumoniae 1 NA

GCF_002187055 Contig Klebsiella pneumoniae G4749 Klebsiella pneumoniae 1 Klebsiella pneumoniae 1 Klebsiella pneumoniae 1 NA

GCF_002187065 Contig Klebsiella pneumoniae G4747 Klebsiella pneumoniae 1 Klebsiella pneumoniae 1 Klebsiella pneumoniae 1 NA

GCF_002187095 Contig Klebsiella pneumoniae G4731 Klebsiella pneumoniae 1 Klebsiella pneumoniae 1 Klebsiella pneumoniae 1 NA

GCF_002187105 Contig Klebsiella pneumoniae G4730 Klebsiella pneumoniae 1 Klebsiella pneumoniae 1 Klebsiella pneumoniae 1 NA

GCF_002187135 Contig Klebsiella pneumoniae G4717 Klebsiella pneumoniae 1 Klebsiella pneumoniae 1 Klebsiella pneumoniae 1 NA

GCF_002187145 Contig Klebsiella pneumoniae G4712 Klebsiella pneumoniae 1 Klebsiella pneumoniae 1 Klebsiella pneumoniae 1 NA

GCF_002187175 Contig Klebsiella quasipneumoniae similipneumoniae G4612 Klebsiella quasipneumoniae 1 Klebsiella quasipneumoniae 1 Klebsiella quasipneumoniae NA

GCF_002187185 Contig Klebsiella pneumoniae G4605 Klebsiella pneumoniae 1 Klebsiella pneumoniae 1 Klebsiella pneumoniae 1 NA

GCF_002187215 Contig Klebsiella quasipneumoniae similipneumoniae G4582 Klebsiella quasipneumoniae 1 Klebsiella quasipneumoniae 1 Klebsiella quasipneumoniae NA

GCF_002187225 Contig Klebsiella quasipneumoniae similipneumoniae G4601 Klebsiella quasipneumoniae 1 Klebsiella quasipneumoniae 1 Klebsiella quasipneumoniae NA

GCF_002187245 Contig Klebsiella pneumoniae G3704 Klebsiella pneumoniae 1 Klebsiella pneumoniae 1 Klebsiella pneumoniae 1 NA

GCF_002187255 Contig Klebsiella pneumoniae G4348 Klebsiella pneumoniae 1 Klebsiella pneumoniae 1 Klebsiella pneumoniae 1 NA

GCF_002187295 Contig Klebsiella pneumoniae G3504 Klebsiella pneumoniae 1 Klebsiella pneumoniae 1 Klebsiella pneumoniae 1 NA

GCF_002187305 Contig Klebsiella pneumoniae G3435 Klebsiella pneumoniae 1 Klebsiella pneumoniae 1 Klebsiella pneumoniae 1 NA

GCF_002187315 Contig Klebsiella pneumoniae G2666 Klebsiella pneumoniae 1 Klebsiella pneumoniae 1 Klebsiella pneumoniae 1 NA

GCF_002187335 Contig Klebsiella pneumoniae G292 Klebsiella pneumoniae 1 Klebsiella pneumoniae 1 Klebsiella pneumoniae 1 NA

GCF_002187375 Contig Klebsiella pneumoniae G2629 Klebsiella pneumoniae 1 Klebsiella pneumoniae 1 Klebsiella pneumoniae 1 NA

GCF_002187385 Contig Klebsiella pneumoniae G2280 Klebsiella pneumoniae 1 Klebsiella pneumoniae 1 Klebsiella pneumoniae 1 NA

GCF_002187395 Contig Klebsiella pneumoniae G2275 Klebsiella pneumoniae 1 Klebsiella pneumoniae 1 Klebsiella pneumoniae 1 NA

GCF_002187405 Contig Klebsiella pneumoniae G1598 Klebsiella pneumoniae 1 Klebsiella pneumoniae 1 Klebsiella pneumoniae 1 NA

GCF_002187455 Contig Klebsiella pneumoniae G1512 Klebsiella pneumoniae 1 Klebsiella pneumoniae 1 Klebsiella pneumoniae 1 NA

GCF_002187465 Contig Klebsiella quasipneumoniae similipneumoniae CAB1577 Klebsiella quasipneumoniae 1 Klebsiella quasipneumoniae 1 Klebsiella quasipneumoniae NA

GCF_002187475 Contig Klebsiella pneumoniae G1015 Klebsiella pneumoniae 1 Klebsiella pneumoniae 1 Klebsiella pneumoniae 1 NA

GCF_002187485 Contig Klebsiella quasipneumoniae similipneumoniae G1129 Klebsiella quasipneumoniae 2 Klebsiella quasipneumoniae 2 Klebsiella quasipneumoniae NA

GCF_002187535 Contig Klebsiella pneumoniae CAB1354 Klebsiella pneumoniae 1 Klebsiella pneumoniae 1 Klebsiella pneumoniae 1 NA

GCF_002187545 Contig Klebsiella pneumoniae BH3723 Klebsiella pneumoniae 1 Klebsiella pneumoniae 1 Klebsiella pneumoniae 1 NA

GCF_002187575 Contig Klebsiella pneumoniae CAB1442 Klebsiella pneumoniae 1 Klebsiella pneumoniae 1 Klebsiella pneumoniae 1 NA

GCF_002187585 Contig Klebsiella pneumoniae BH3953 Klebsiella pneumoniae 1 Klebsiella pneumoniae 1 Klebsiella pneumoniae 1 NA

GCF_002187615 Contig Klebsiella pneumoniae BH3183 Klebsiella pneumoniae 1 Klebsiella pneumoniae 1 Klebsiella pneumoniae 1 NA

GCF_002187625 Contig Klebsiella pneumoniae BH1986 Klebsiella pneumoniae 1 Klebsiella pneumoniae 1 Klebsiella pneumoniae 1 NA

GCF_002187655 Contig Klebsiella pneumoniae AK905 Klebsiella pneumoniae 1 Klebsiella pneumoniae 1 Klebsiella pneumoniae 1 NA

GCF_002187665 Contig Klebsiella pneumoniae BH1877 Klebsiella pneumoniae 1 Klebsiella pneumoniae 1 Klebsiella pneumoniae 1 NA

GCF_002187685 Contig Klebsiella pneumoniae AK800 Klebsiella pneumoniae 1 Klebsiella pneumoniae 1 Klebsiella pneumoniae 1 NA

GCF_002187715 Contig Klebsiella pneumoniae AK896 Klebsiella pneumoniae 1 Klebsiella pneumoniae 1 Klebsiella pneumoniae 1 NA

GCF_002187735 Contig Klebsiella pneumoniae AK1025 Klebsiella pneumoniae 1 Klebsiella pneumoniae 1 Klebsiella pneumoniae 1 NA

GCF_002187745 Contig Klebsiella pneumoniae PO380 Klebsiella pneumoniae 1 Klebsiella pneumoniae 1 Klebsiella pneumoniae 1 NA

GCF_002187775 Contig Klebsiella pneumoniae PO2659 Klebsiella pneumoniae 1 Klebsiella pneumoniae 1 Klebsiella pneumoniae 1 NA

GCF_002187785 Contig Klebsiella pneumoniae AK1023 Klebsiella pneumoniae 1 Klebsiella pneumoniae 1 Klebsiella pneumoniae 1 NA

GCF_002187805 Contig Klebsiella pneumoniae PO1563 Klebsiella pneumoniae 1 Klebsiella pneumoniae 1 Klebsiella pneumoniae 1 NA

GCF_002187835 Contig Klebsiella pneumoniae PO1758 Klebsiella pneumoniae 1 Klebsiella pneumoniae 1 Klebsiella pneumoniae 1 NA

GCF_002187855 Contig Klebsiella pneumoniae PO1407 Klebsiella pneumoniae 1 Klebsiella pneumoniae 1 Klebsiella pneumoniae 1 NA

GCF_002187865 Contig Klebsiella pneumoniae HB AB3196 Klebsiella pneumoniae 1 Klebsiella pneumoniae 1 Klebsiella pneumoniae 1 NA

GCF_002187895 Contig Klebsiella pneumoniae HB4285 Klebsiella pneumoniae 1 Klebsiella pneumoniae 1 Klebsiella pneumoniae 1 NA

GCF_002187915 Contig Klebsiella pneumoniae HB1051 Klebsiella pneumoniae 1 Klebsiella pneumoniae 1 Klebsiella pneumoniae 1 NA

GCF_002187935 Complete Klebsiella quasipneumoniae similipneumoniae G747 Klebsiella quasipneumoniae 1 Klebsiella quasipneumoniae 1 Klebsiella quasipneumoniae NA

GCF_002187945 Contig Klebsiella quasipneumoniae similipneumoniae G4704 Klebsiella quasipneumoniae 1 Klebsiella quasipneumoniae 1 Klebsiella quasipneumoniae NA

GCF_002187975 Contig Klebsiella pneumoniae G4606 Klebsiella pneumoniae 1 Klebsiella pneumoniae 1 Klebsiella pneumoniae 1 NA

GCF_002187995 Contig Klebsiella pneumoniae G4049 Klebsiella pneumoniae 1 Klebsiella pneumoniae 1 Klebsiella pneumoniae 1 NA

GCF_002188015 Contig Klebsiella pneumoniae G4597 Klebsiella pneumoniae 1 Klebsiella pneumoniae 1 Klebsiella pneumoniae 1 NA

GCF_002188025 Contig Klebsiella quasipneumoniae similipneumoniae G4593 Klebsiella quasipneumoniae 1 Klebsiella quasipneumoniae 1 Klebsiella quasipneumoniae NA

GCF_002188055 Contig Klebsiella pneumoniae G3051 Klebsiella pneumoniae 1 Klebsiella pneumoniae 1 Klebsiella pneumoniae 1 NA

GCF_002188075 Contig Klebsiella pneumoniae G186 Klebsiella pneumoniae 1 Klebsiella pneumoniae 1 Klebsiella pneumoniae 1 NA

GCF_002188095 Contig Klebsiella pneumoniae G160 Klebsiella pneumoniae 1 Klebsiella pneumoniae 1 Klebsiella pneumoniae 1 NA

GCF_002188105 Contig Klebsiella pneumoniae G1457 Klebsiella pneumoniae 1 Klebsiella pneumoniae 1 Klebsiella pneumoniae 1 NA

GCF_002188135 Contig Klebsiella pneumoniae CAB1505 Klebsiella pneumoniae 1 Klebsiella pneumoniae 1 Klebsiella pneumoniae 1 NA

GCF_002188145 Contig Klebsiella pneumoniae CAB1458 Klebsiella pneumoniae 1 Klebsiella pneumoniae 1 Klebsiella pneumoniae 1 NA

GCF_002188175 Contig Klebsiella pneumoniae BH2937 Klebsiella pneumoniae 1 Klebsiella pneumoniae 1 Klebsiella pneumoniae 1 NA

GCF_002188185 Contig Klebsiella pneumoniae BH2511 Klebsiella pneumoniae 1 Klebsiella pneumoniae 1 Klebsiella pneumoniae 1 NA

GCF_002188215 Contig Klebsiella quasipneumoniae quasipneumoniae AK SD007 Klebsiella quasipneumoniae 2 Klebsiella quasipneumoniae 2 Klebsiella quasipneumoniae NA

GCF_002188235 Contig Klebsiella pneumoniae BH2308 Klebsiella pneumoniae 1 Klebsiella pneumoniae 1 Klebsiella pneumoniae 1 NA

GCF_002188245 Contig Klebsiella pneumoniae AK SD011 Klebsiella pneumoniae 1 Klebsiella pneumoniae 1 Klebsiella pneumoniae 1 NA

GCF_002188255 Contig Klebsiella pneumoniae AK991 Klebsiella pneumoniae 1 Klebsiella pneumoniae 1 Klebsiella pneumoniae 1 NA

GCF_002188295 Contig Klebsiella pneumoniae AK1024 Klebsiella pneumoniae 1 Klebsiella pneumoniae 1 Klebsiella pneumoniae 1 NA

GCF_002189165 Scaffold Klebsiella pneumoniae C3-D9-12 Klebsiella pneumoniae 1 Klebsiella pneumoniae 1 Klebsiella pneumoniae 1 NA

GCF_002189175 Scaffold Klebsiella pneumoniae C5-E1-13 Klebsiella pneumoniae 1 Klebsiella pneumoniae 1 Klebsiella pneumoniae 1 NA

GCF_002189185 Scaffold Klebsiella pneumoniae C4-D10-13 Klebsiella pneumoniae 1 Klebsiella pneumoniae 1 Klebsiella pneumoniae 1 NA

GCF_002189245 Scaffold Klebsiella pneumoniae C10-E6-15 Klebsiella pneumoniae 1 Klebsiella pneumoniae 1 Klebsiella pneumoniae 1 NA

GCF_002189255 Contig Klebsiella pneumoniae C11-E7-15 Klebsiella pneumoniae 1 Klebsiella pneumoniae 1 Klebsiella pneumoniae 1 NA

GCF_002189265 Contig Klebsiella pneumoniae C6-E2-14 Klebsiella pneumoniae 1 Klebsiella pneumoniae 1 Klebsiella pneumoniae 1 NA

GCF_002189305 Contig Klebsiella pneumoniae C12-E8-15 Klebsiella pneumoniae 1 Klebsiella pneumoniae 1 Klebsiella pneumoniae 1 NA

GCF_002189325 Contig Klebsiella pneumoniae C13-E9-15 Klebsiella pneumoniae 1 Klebsiella pneumoniae 1 Klebsiella pneumoniae 1 NA

GCF_002189335 Contig Klebsiella pneumoniae C7-E3-14 Klebsiella pneumoniae 1 Klebsiella pneumoniae 1 Klebsiella pneumoniae 1 NA

GCF_002189345 Scaffold Klebsiella pneumoniae C8-E4-15 Klebsiella pneumoniae 1 Klebsiella pneumoniae 1 Klebsiella pneumoniae 1 NA

GCF_002189385 Scaffold Klebsiella pneumoniae C9-E5-15 Klebsiella pneumoniae 1 Klebsiella pneumoniae 1 Klebsiella pneumoniae 1 NA

GCF_002189415 Contig Klebsiella pneumoniae C14-E10-16 Klebsiella pneumoniae 1 Klebsiella pneumoniae 1 Klebsiella pneumoniae 1 NA

GCF_002192315 Complete Klebsiella pneumoniae AR0126 Klebsiella pneumoniae 1 Klebsiella pneumoniae 1 Klebsiella pneumoniae 1 NA

GCF_002192335 Complete Klebsiella pneumoniae AR0113 Klebsiella pneumoniae 1 Klebsiella pneumoniae 1 Klebsiella pneumoniae 1 NA

GCF_002192375 Complete Klebsiella pneumoniae AR0138 Klebsiella pneumoniae 1 Klebsiella pneumoniae 1 Klebsiella pneumoniae 1 NA

GCF_002192755 Contig Klebsiella michiganensis DPB3 Klebsiella michiganensis 1 Klebsiella michiganensis 1 Klebsiella michiganensis 1 NA

GCF_002192975 Contig Klebsiella pneumoniae 1721 Klebsiella pneumoniae 1 Klebsiella pneumoniae 1 Klebsiella pneumoniae 1 NA

GCF_002197225 Complete Klebsiella pneumoniae AR0120 Klebsiella pneumoniae 1 Klebsiella pneumoniae 1 Klebsiella pneumoniae 1 NA

GCF_002197505 Complete Klebsiella pneumoniae AR0125 Klebsiella pneumoniae 1 Klebsiella pneumoniae 1 Klebsiella pneumoniae 1 NA

GCF_002197675 Contig Klebsiella quasipneumoniae MGF005 Klebsiella quasipneumoniae 1 Klebsiella quasipneumoniae 1 Klebsiella quasipneumoniae NA

GCF_002197725 Contig Klebsiella quasipneumoniae MGF008 Klebsiella quasipneumoniae 1 Klebsiella quasipneumoniae 1 Klebsiella quasipneumoniae NA

GCF_002202195 Complete Klebsiella pneumoniae AR0139 Klebsiella pneumoniae 1 Klebsiella pneumoniae 1 Klebsiella pneumoniae 1 NA

GCF_002202215 Complete Klebsiella pneumoniae AR0145 Klebsiella pneumoniae 1 Klebsiella pneumoniae 1 Klebsiella pneumoniae 1 NA

GCF_002202235 Complete Klebsiella pneumoniae AR0152 Klebsiella pneumoniae 1 Klebsiella pneumoniae 1 Klebsiella pneumoniae 1 NA

GCF_002202255 Complete Klebsiella pneumoniae AR0148 Klebsiella pneumoniae 1 Klebsiella pneumoniae 1 Klebsiella pneumoniae 1 NA

GCF_002202275 Complete Klebsiella pneumoniae AR0107 Klebsiella pneumoniae 1 Klebsiella pneumoniae 1 Klebsiella pneumoniae 1 NA

GCF_002204605 Scaffold Klebsiella aerogenes PX01 Klebsiella aerogenes 2 Klebsiella aerogenes Klebsiella aerogenes NA

GCF_002205015 Contig Klebsiella pneumoniae 852K Klebsiella pneumoniae 1 Klebsiella pneumoniae 1 Klebsiella pneumoniae 1 NA

GCF_002205025 Contig Klebsiella pneumoniae 852 Klebsiella pneumoniae 1 Klebsiella pneumoniae 1 Klebsiella pneumoniae 1 NA

GCF_002205045 Contig Klebsiella pneumoniae 1337LF Klebsiella pneumoniae 1 Klebsiella pneumoniae 1 Klebsiella pneumoniae 1 NA

GCF_002205055 Contig Klebsiella pneumoniae 872 Klebsiella pneumoniae 1 Klebsiella pneumoniae 1 Klebsiella pneumoniae 1 NA

GCF_002205985 Contig Klebsiella pneumoniae 302 Klebsiella pneumoniae 1 Klebsiella pneumoniae 1 Klebsiella pneumoniae 1 NA

GCF_002205995 Contig Klebsiella pneumoniae 327 Klebsiella pneumoniae 1 Klebsiella pneumoniae 1 Klebsiella pneumoniae 1 NA

GCF_002206015 Contig Klebsiella pneumoniae 329 Klebsiella pneumoniae 1 Klebsiella pneumoniae 1 Klebsiella pneumoniae 1 NA

GCF_002209405 Complete Klebsiella pneumoniae 19051 Klebsiella pneumoniae 1 Klebsiella pneumoniae 1 Klebsiella pneumoniae 1 NA

GCF_002209465 Contig Klebsiella pneumoniae KPC40 Klebsiella pneumoniae 1 Klebsiella pneumoniae 1 Klebsiella pneumoniae 1 NA

GCF_002210445 Contig Klebsiella pneumoniae pneumoniae KpvST23L OXA48 Klebsiella pneumoniae 1 Klebsiella pneumoniae 1 Klebsiella pneumoniae 1 NA

GCF_002211125 Scaffold Klebsiella pneumoniae SCPM-O-B7851 KPI3014 Klebsiella pneumoniae 1 Klebsiella pneumoniae 1 Klebsiella pneumoniae 1 NA

GCF_002211425 Contig Klebsiella variicola MB351 Klebsiella variicola Klebsiella variicola Klebsiella variicola NA

GCF_002211665 Chromosome Klebsiella pneumoniae 704SK6 Klebsiella pneumoniae 1 Klebsiella pneumoniae 1 Klebsiella pneumoniae 1 NA

GCF_002211905 Complete Klebsiella pneumoniae DHQP1605752 NV Klebsiella pneumoniae 1 Klebsiella pneumoniae 1 Klebsiella pneumoniae 1 NA

GCF_002216835 Complete Klebsiella michiganensis K516 Klebsiella michiganensis 1 Klebsiella michiganensis 1 Klebsiella michiganensis 1 NA

GCF_002234875 Contig Klebsiella pneumoniae KPM2895 Klebsiella pneumoniae 1 Klebsiella pneumoniae 1 Klebsiella pneumoniae 1 NA

GCF_002234895 Contig Klebsiella pneumoniae KP1099 Klebsiella pneumoniae 1 Klebsiella pneumoniae 1 Klebsiella pneumoniae 1 NA

GCF_002235035 Scaffold Klebsiella pneumoniae 67 Klebsiella pneumoniae 1 Klebsiella pneumoniae 1 Klebsiella pneumoniae 1 NA

GCF_002235045 Contig Klebsiella pneumoniae 68 Klebsiella pneumoniae 1 Klebsiella pneumoniae 1 Klebsiella pneumoniae 1 NA

GCF_002235055 Scaffold Klebsiella pneumoniae 66 Klebsiella pneumoniae 1 Klebsiella pneumoniae 1 Klebsiella pneumoniae 1 NA

GCF_002235095 Scaffold Klebsiella pneumoniae 63 Klebsiella pneumoniae 1 Klebsiella pneumoniae 1 Klebsiella pneumoniae 1 NA

GCF_002235105 Scaffold Klebsiella pneumoniae 62 Klebsiella pneumoniae 1 Klebsiella pneumoniae 1 Klebsiella pneumoniae 1 NA

GCF_002235115 Scaffold Klebsiella pneumoniae 61 Klebsiella pneumoniae 1 Klebsiella pneumoniae 1 Klebsiella pneumoniae 1 NA

GCF_002235155 Scaffold Klebsiella pneumoniae 59 Klebsiella pneumoniae 1 Klebsiella pneumoniae 1 Klebsiella pneumoniae 1 NA

GCF_002235165 Scaffold Klebsiella pneumoniae 57 Klebsiella pneumoniae 1 Klebsiella pneumoniae 1 Klebsiella pneumoniae 1 NA

GCF_002235175 Scaffold Klebsiella pneumoniae 58 Klebsiella pneumoniae 1 Klebsiella pneumoniae 1 Klebsiella pneumoniae 1 NA

GCF_002235215 Scaffold Klebsiella pneumoniae 56 Klebsiella pneumoniae 1 Klebsiella pneumoniae 1 Klebsiella pneumoniae 1 NA

GCF_002235235 Contig Klebsiella pneumoniae 54 Klebsiella pneumoniae 1 Klebsiella pneumoniae 1 Klebsiella pneumoniae 1 NA

GCF_002235265 Scaffold Klebsiella pneumoniae 52 Klebsiella pneumoniae 1 Klebsiella pneumoniae 1 Klebsiella pneumoniae 1 NA

GCF_002235295 Scaffold Klebsiella pneumoniae 51 Klebsiella pneumoniae 1 Klebsiella pneumoniae 1 Klebsiella pneumoniae 1 NA

GCF_002235315 Scaffold Klebsiella pneumoniae 50 Klebsiella pneumoniae 1 Klebsiella pneumoniae 1 Klebsiella pneumoniae 1 NA

GCF_002235325 Contig Klebsiella pneumoniae 49 Klebsiella pneumoniae 1 Klebsiella pneumoniae 1 Klebsiella pneumoniae 1 NA

GCF_002235355 Scaffold Klebsiella pneumoniae 46 Klebsiella pneumoniae 1 Klebsiella pneumoniae 1 Klebsiella pneumoniae 1 NA

GCF_002235375 Scaffold Klebsiella pneumoniae 44 Klebsiella pneumoniae 1 Klebsiella pneumoniae 1 Klebsiella pneumoniae 1 NA

GCF_002235395 Scaffold Klebsiella pneumoniae 43 Klebsiella pneumoniae 1 Klebsiella pneumoniae 1 Klebsiella pneumoniae 1 NA

GCF_002235415 Scaffold Klebsiella pneumoniae 42 Klebsiella pneumoniae 1 Klebsiella pneumoniae 1 Klebsiella pneumoniae 1 NA

GCF_002235435 Contig Klebsiella pneumoniae 45 Klebsiella pneumoniae 1 Klebsiella pneumoniae 1 Klebsiella pneumoniae 1 NA

GCF_002235455 Scaffold Klebsiella pneumoniae 40 Klebsiella pneumoniae 1 Klebsiella pneumoniae 1 Klebsiella pneumoniae 1 NA

GCF_002235475 Scaffold Klebsiella pneumoniae 34 Klebsiella pneumoniae 1 Klebsiella pneumoniae 1 Klebsiella pneumoniae 1 NA

GCF_002235495 Contig Klebsiella pneumoniae 14 Klebsiella pneumoniae 1 Klebsiella pneumoniae 1 Klebsiella pneumoniae 1 NA

GCF_002235515 Contig Klebsiella pneumoniae 18 Klebsiella pneumoniae 1 Klebsiella pneumoniae 1 Klebsiella pneumoniae 1 NA

GCF_002235595 Contig Klebsiella pneumoniae 29 Klebsiella pneumoniae 1 Klebsiella pneumoniae 1 Klebsiella pneumoniae 1 NA

GCF_002235615 Scaffold Klebsiella pneumoniae 28 Klebsiella pneumoniae 1 Klebsiella pneumoniae 1 Klebsiella pneumoniae 1 NA

GCF_002235635 Scaffold Klebsiella pneumoniae 26 Klebsiella pneumoniae 1 Klebsiella pneumoniae 1 Klebsiella pneumoniae 1 NA

GCF_002235655 Contig Klebsiella pneumoniae 25 Klebsiella pneumoniae 1 Klebsiella pneumoniae 1 Klebsiella pneumoniae 1 NA

GCF_002235705 Contig Klebsiella pneumoniae 10 Klebsiella pneumoniae 1 Klebsiella pneumoniae 1 Klebsiella pneumoniae 1 NA

GCF_002235735 Contig Klebsiella pneumoniae 3 Klebsiella pneumoniae 1 Klebsiella pneumoniae 1 Klebsiella pneumoniae 1 NA

GCF_002235825 Scaffold Klebsiella pneumoniae 30 Klebsiella pneumoniae 1 Klebsiella pneumoniae 1 Klebsiella pneumoniae 1 NA

GCF_002235835 Contig Klebsiella pneumoniae 27 Klebsiella pneumoniae 1 Klebsiella pneumoniae 1 Klebsiella pneumoniae 1 NA

GCF_002235935 Contig Klebsiella pneumoniae 17 Klebsiella pneumoniae 1 Klebsiella pneumoniae 1 Klebsiella pneumoniae 1 NA

GCF_002235945 Scaffold Klebsiella pneumoniae 16 Klebsiella pneumoniae 1 Klebsiella pneumoniae 1 Klebsiella pneumoniae 1 NA

GCF_002235975 Scaffold Klebsiella pneumoniae 13 Klebsiella pneumoniae 1 Klebsiella pneumoniae 1 Klebsiella pneumoniae 1 NA

GCF_002235985 Scaffold Klebsiella pneumoniae 2 Klebsiella pneumoniae 1 Klebsiella pneumoniae 1 Klebsiella pneumoniae 1 NA

GCF_002236015 Contig Klebsiella pneumoniae 7 Klebsiella pneumoniae 1 Klebsiella pneumoniae 1 Klebsiella pneumoniae 1 NA

GCF_002236055 Contig Klebsiella pneumoniae 4 Klebsiella pneumoniae 1 Klebsiella pneumoniae 1 Klebsiella pneumoniae 1 NA

GCF_002236145 Scaffold Klebsiella pneumoniae 60 Klebsiella pneumoniae 1 Klebsiella pneumoniae 1 Klebsiella pneumoniae 1 NA

GCF_002236155 Scaffold Klebsiella pneumoniae 55 Klebsiella pneumoniae 1 Klebsiella pneumoniae 1 Klebsiella pneumoniae 1 NA

GCF_002236185 Scaffold Klebsiella pneumoniae 48 Klebsiella pneumoniae 1 Klebsiella pneumoniae 1 Klebsiella pneumoniae 1 NA

GCF_002236205 Scaffold Klebsiella pneumoniae 47 Klebsiella pneumoniae 1 Klebsiella pneumoniae 1 Klebsiella pneumoniae 1 NA

GCF_002236245 Scaffold Klebsiella pneumoniae 41 Klebsiella pneumoniae 1 Klebsiella pneumoniae 1 Klebsiella pneumoniae 1 NA

GCF_002236265 Scaffold Klebsiella pneumoniae 32 Klebsiella pneumoniae 1 Klebsiella pneumoniae 1 Klebsiella pneumoniae 1 NA

GCF_002236305 Scaffold Klebsiella pneumoniae 38 Klebsiella pneumoniae 1 Klebsiella pneumoniae 1 Klebsiella pneumoniae 1 NA

GCF_002236315 Scaffold Klebsiella pneumoniae 31 Klebsiella pneumoniae 1 Klebsiella pneumoniae 1 Klebsiella pneumoniae 1 NA

GCF_002236345 Scaffold Klebsiella pneumoniae 21 Klebsiella pneumoniae 1 Klebsiella pneumoniae 1 Klebsiella pneumoniae 1 NA

GCF_002236385 Contig Klebsiella pneumoniae 9 Klebsiella pneumoniae 1 Klebsiella pneumoniae 1 Klebsiella pneumoniae 1 NA

GCF_002236405 Scaffold Klebsiella pneumoniae 8 Klebsiella pneumoniae 1 Klebsiella pneumoniae 1 Klebsiella pneumoniae 1 NA

GCF_002236425 Scaffold Klebsiella pneumoniae 24 Klebsiella pneumoniae 1 Klebsiella pneumoniae 1 Klebsiella pneumoniae 1 NA

GCF_002237985 Contig Klebsiella pneumoniae VD412 Klebsiella pneumoniae 1 Klebsiella pneumoniae 1 Klebsiella pneumoniae 1 NA

GCF_002238125 Scaffold Klebsiella pneumoniae OK8 Klebsiella pneumoniae 1 Klebsiella pneumoniae 1 Klebsiella pneumoniae 1 NA

GCF_002239705 Contig Klebsiella pneumoniae pneumoniae CREC014-1 Klebsiella pneumoniae 1 Klebsiella pneumoniae 1 Klebsiella pneumoniae 1 NA

GCF_002239715 Contig Klebsiella pneumoniae pneumoniae CREC003-1 Klebsiella pneumoniae 1 Klebsiella pneumoniae 1 Klebsiella pneumoniae 1 NA

GCF_002239805 Contig Klebsiella pneumoniae pneumoniae 4REI026-2 Klebsiella pneumoniae 1 Klebsiella pneumoniae 1 Klebsiella pneumoniae 1 NA

GCF_002239815 Contig Klebsiella pneumoniae pneumoniae CREI026-1 Klebsiella pneumoniae 1 Klebsiella pneumoniae 1 Klebsiella pneumoniae 1 NA

GCF_002239875 Contig Klebsiella pneumoniae ESBL-SIUK7 Klebsiella pneumoniae 1 Klebsiella pneumoniae 1 Klebsiella pneumoniae 1 NA

GCF_002239895 Complete Klebsiella quasipneumoniae G4584 Klebsiella quasipneumoniae 1 Klebsiella quasipneumoniae 1 Klebsiella quasipneumoniae NA

GCF_002239965 Contig Klebsiella pneumoniae G746 Klebsiella pneumoniae 1 Klebsiella pneumoniae 1 Klebsiella pneumoniae 1 NA

GCF_002240295 Complete Klebsiella quasipneumoniae HKUOPA4 Klebsiella quasipneumoniae 1 Klebsiella quasipneumoniae 1 Klebsiella quasipneumoniae NA

GCF_002240315 Complete Klebsiella quasipneumoniae HKUOPJ4 Klebsiella quasipneumoniae 1 Klebsiella quasipneumoniae 1 Klebsiella quasipneumoniae NA

GCF_002240335 Complete Klebsiella quasipneumoniae HKUOPL4 Klebsiella quasipneumoniae 1 Klebsiella quasipneumoniae 1 Klebsiella quasipneumoniae NA

GCF_002241035 Contig Klebsiella pneumoniae ICBKpBL-III031 Klebsiella pneumoniae 1 Klebsiella pneumoniae 1 Klebsiella pneumoniae 1 NA

GCF_002243445 Complete Klebsiella pneumoniae BIC1 Klebsiella pneumoniae 1 Klebsiella pneumoniae 1 Klebsiella pneumoniae 1 NA

GCF_002245455 Scaffold Klebsiella variicola KV093 Klebsiella variicola Klebsiella variicola Klebsiella variicola NA

GCF_002246035 Contig Klebsiella pneumoniae pneumoniae KPCTRPRTH04 Klebsiella pneumoniae 1 Klebsiella pneumoniae 1 Klebsiella pneumoniae 1 NA

GCF_002246045 Contig Klebsiella pneumoniae pneumoniae KPCTRPRTH05 Klebsiella pneumoniae 1 Klebsiella pneumoniae 1 Klebsiella pneumoniae 1 NA

GCF_002246075 Contig Klebsiella pneumoniae ECCTRSRTH01 Klebsiella pneumoniae 1 Klebsiella pneumoniae 1 Klebsiella pneumoniae 1 NA

GCF_002246125 Contig Klebsiella pneumoniae pneumoniae KPCRETH16 Klebsiella pneumoniae 1 Klebsiella pneumoniae 1 Klebsiella pneumoniae 1 NA

GCF_002246155 Contig Klebsiella pneumoniae pneumoniae ECCRETH03 Klebsiella pneumoniae 1 Klebsiella pneumoniae 1 Klebsiella pneumoniae 1 NA

GCF_002246235 Contig Klebsiella pneumoniae pneumoniae KPCRETH20 Klebsiella pneumoniae 1 Klebsiella pneumoniae 1 Klebsiella pneumoniae 1 NA

GCF_002246245 Contig Klebsiella pneumoniae pneumoniae KPCRETH18 Klebsiella pneumoniae 1 Klebsiella pneumoniae 1 Klebsiella pneumoniae 1 NA

GCF_002246275 Contig Klebsiella pneumoniae pneumoniae KPCRETH22 Klebsiella pneumoniae 1 Klebsiella pneumoniae 1 Klebsiella pneumoniae 1 NA

GCF_002246285 Contig Klebsiella pneumoniae pneumoniae KPCRETH23 Klebsiella pneumoniae 1 Klebsiella pneumoniae 1 Klebsiella pneumoniae 1 NA

GCF_002246315 Contig Klebsiella pneumoniae pneumoniae KPCRETH25 Klebsiella pneumoniae 1 Klebsiella pneumoniae 1 Klebsiella pneumoniae 1 NA

GCF_002246325 Contig Klebsiella pneumoniae pneumoniae KPCRETH24 Klebsiella pneumoniae 1 Klebsiella pneumoniae 1 Klebsiella pneumoniae 1 NA

GCF_002246355 Contig Klebsiella pneumoniae pneumoniae KPCRETH27 Klebsiella pneumoniae 1 Klebsiella pneumoniae 1 Klebsiella pneumoniae 1 NA

GCF_002246365 Contig Klebsiella pneumoniae pneumoniae KPCRETH28 Klebsiella pneumoniae 1 Klebsiella pneumoniae 1 Klebsiella pneumoniae 1 NA

GCF_002246395 Contig Klebsiella pneumoniae pneumoniae KPCRETH30 Klebsiella pneumoniae 1 Klebsiella pneumoniae 1 Klebsiella pneumoniae 1 NA

GCF_002246415 Contig Klebsiella pneumoniae pneumoniae KPCRETH29 Klebsiella pneumoniae 1 Klebsiella pneumoniae 1 Klebsiella pneumoniae 1 NA

GCF_002246435 Contig Klebsiella pneumoniae pneumoniae KPCRETH32 Klebsiella pneumoniae 1 Klebsiella pneumoniae 1 Klebsiella pneumoniae 1 NA

GCF_002246455 Contig Klebsiella pneumoniae pneumoniae KPCRETH33 Klebsiella pneumoniae 1 Klebsiella pneumoniae 1 Klebsiella pneumoniae 1 NA

GCF_002246475 Contig Klebsiella pneumoniae pneumoniae ECCRETH07 Klebsiella pneumoniae 1 Klebsiella pneumoniae 1 Klebsiella pneumoniae 1 NA

GCF_002246555 Contig Klebsiella pneumoniae pneumoniae KPCTRPRTH02 Klebsiella pneumoniae 1 Klebsiella pneumoniae 1 Klebsiella pneumoniae 1 NA

GCF_002246575 Contig Klebsiella pneumoniae pneumoniae KPCRETH09 Klebsiella pneumoniae 1 Klebsiella pneumoniae 1 Klebsiella pneumoniae 1 NA

GCF_002246635 Contig Klebsiella pneumoniae pneumoniae KPCRETH19 Klebsiella pneumoniae 1 Klebsiella pneumoniae 1 Klebsiella pneumoniae 1 NA

GCF_002246655 Contig Klebsiella pneumoniae pneumoniae KPCRETH21 Klebsiella pneumoniae 1 Klebsiella pneumoniae 1 Klebsiella pneumoniae 1 NA

GCF_002246675 Contig Klebsiella quasipneumoniae similipneumoniae KPCRETH06 Klebsiella quasipneumoniae 1 Klebsiella quasipneumoniae 1 Klebsiella quasipneumoniae NA

GCF_002246685 Contig Klebsiella pneumoniae pneumoniae KPCRETH26 Klebsiella pneumoniae 1 Klebsiella pneumoniae 1 Klebsiella pneumoniae 1 NA

GCF_002246695 Contig Klebsiella pneumoniae pneumoniae KPCRETH07 Klebsiella pneumoniae 1 Klebsiella pneumoniae 1 Klebsiella pneumoniae 1 NA

GCF_002246705 Contig Klebsiella pneumoniae pneumoniae KPCRETH08 Klebsiella pneumoniae 1 Klebsiella pneumoniae 1 Klebsiella pneumoniae 1 NA

GCF_002246765 Contig Klebsiella pneumoniae pneumoniae KPCTRPRTH03 Klebsiella pneumoniae 1 Klebsiella pneumoniae 1 Klebsiella pneumoniae 1 NA

GCF_002246785 Contig Klebsiella pneumoniae pneumoniae ECCRETH01 Klebsiella pneumoniae 1 Klebsiella pneumoniae 1 Klebsiella pneumoniae 1 NA

GCF_002246835 Contig Klebsiella pneumoniae pneumoniae KPCRETH10 Klebsiella pneumoniae 1 Klebsiella pneumoniae 1 Klebsiella pneumoniae 1 NA

GCF_002246845 Contig Klebsiella pneumoniae pneumoniae KPCRETH14 Klebsiella pneumoniae 1 Klebsiella pneumoniae 1 Klebsiella pneumoniae 1 NA

GCF_002246875 Contig Klebsiella pneumoniae pneumoniae KPCTRPRTH01 Klebsiella pneumoniae 1 Klebsiella pneumoniae 1 Klebsiella pneumoniae 1 NA

GCF_002246895 Contig Klebsiella pneumoniae pneumoniae KPCRETH05 Klebsiella pneumoniae 1 Klebsiella pneumoniae 1 Klebsiella pneumoniae 1 NA

GCF_002247365 Contig Klebsiella pneumoniae pneumoniae KPCRETH11 Klebsiella pneumoniae 1 Klebsiella pneumoniae 1 Klebsiella pneumoniae 1 NA

GCF_002247375 Contig Klebsiella pneumoniae pneumoniae KPCRETH12 Klebsiella pneumoniae 1 Klebsiella pneumoniae 1 Klebsiella pneumoniae 1 NA

GCF_002247385 Contig Klebsiella pneumoniae pneumoniae KPCRETH15 Klebsiella pneumoniae 1 Klebsiella pneumoniae 1 Klebsiella pneumoniae 1 NA

GCF_002247625 Contig Klebsiella pneumoniae pneumoniae KPSW+01 Klebsiella pneumoniae 1 Klebsiella pneumoniae 1 Klebsiella pneumoniae 1 NA

GCF_002247645 Contig Klebsiella pneumoniae pneumoniae KPSW+02 Klebsiella pneumoniae 1 Klebsiella pneumoniae 1 Klebsiella pneumoniae 1 NA

GCF_002247665 Contig Klebsiella pneumoniae pneumoniae KPSW+03 Klebsiella pneumoniae 1 Klebsiella pneumoniae 1 Klebsiella pneumoniae 1 NA

GCF_002247865 Contig Klebsiella pneumoniae pneumoniae KPH+01 Klebsiella pneumoniae 1 Klebsiella pneumoniae 1 Klebsiella pneumoniae 1 NA

GCF_002247885 Contig Klebsiella pneumoniae pneumoniae KPH+02 Klebsiella pneumoniae 1 Klebsiella pneumoniae 1 Klebsiella pneumoniae 1 NA

GCF_002247895 Contig Klebsiella pneumoniae pneumoniae KPH+03 Klebsiella pneumoniae 1 Klebsiella pneumoniae 1 Klebsiella pneumoniae 1 NA

GCF_002248045 Contig Klebsiella pneumoniae pneumoniae KPCTRSRTH02 Klebsiella pneumoniae 1 Klebsiella pneumoniae 1 Klebsiella pneumoniae 1 NA

GCF_002248055 Contig Klebsiella quasipneumoniae similipneumoniae KPCTRSRTH03 Klebsiella quasipneumoniae 1 Klebsiella quasipneumoniae 1 Klebsiella quasipneumoniae NA

GCF_002248085 Contig Klebsiella quasipneumoniae similipneumoniae KPCTRSRTH04 Klebsiella quasipneumoniae 1 Klebsiella quasipneumoniae 1 Klebsiella quasipneumoniae NA

GCF_002248105 Contig Klebsiella pneumoniae pneumoniae KPCTRSRTH06 Klebsiella pneumoniae 1 Klebsiella pneumoniae 1 Klebsiella pneumoniae 1 NA

GCF_002248115 Complete Klebsiella pneumoniae pneumoniae KPCTRSRTH07 Klebsiella pneumoniae 1 Klebsiella pneumoniae 1 Klebsiella pneumoniae 1 NA

GCF_002248135 Contig Klebsiella pneumoniae KPCTRSRTH08 Klebsiella pneumoniae 1 Klebsiella pneumoniae 1 Klebsiella pneumoniae 1 NA

GCF_002248425 Contig Klebsiella pneumoniae pneumoniae KPPUTH02 Klebsiella pneumoniae 1 Klebsiella pneumoniae 1 Klebsiella pneumoniae 1 NA

GCF_002248435 Contig Klebsiella pneumoniae pneumoniae KPPUTH03 Klebsiella pneumoniae 1 Klebsiella pneumoniae 1 Klebsiella pneumoniae 1 NA

GCF_002248485 Contig Klebsiella pneumoniae pneumoniae KPPUTH04 Klebsiella pneumoniae 1 Klebsiella pneumoniae 1 Klebsiella pneumoniae 1 NA

GCF_002248505 Contig Klebsiella pneumoniae pneumoniae KPPUTH08 Klebsiella pneumoniae 1 Klebsiella pneumoniae 1 Klebsiella pneumoniae 1 NA

GCF_002248525 Contig Klebsiella pneumoniae KPPUTH07 Klebsiella pneumoniae 1 Klebsiella pneumoniae 1 Klebsiella pneumoniae 1 NA

GCF_002248545 Contig Klebsiella pneumoniae pneumoniae KPPSTH01 Klebsiella pneumoniae 1 Klebsiella pneumoniae 1 Klebsiella pneumoniae 1 NA

GCF_002248565 Contig Klebsiella quasipneumoniae similipneumoniae KPPSTH03 Klebsiella quasipneumoniae 1 Klebsiella quasipneumoniae 1 Klebsiella quasipneumoniae NA

GCF_002248585 Contig Klebsiella pneumoniae pneumoniae KPPSTH02 Klebsiella pneumoniae 1 Klebsiella pneumoniae 1 Klebsiella pneumoniae 1 NA

GCF_002248595 Contig Klebsiella pneumoniae pneumoniae KPPSTH04 Klebsiella pneumoniae 1 Klebsiella pneumoniae 1 Klebsiella pneumoniae 1 NA

GCF_002248625 Contig Klebsiella pneumoniae pneumoniae KPCRETH13 Klebsiella pneumoniae 1 Klebsiella pneumoniae 1 Klebsiella pneumoniae 1 NA

GCF_002248635 Contig Klebsiella pneumoniae pneumoniae KPPSTH05 Klebsiella pneumoniae 1 Klebsiella pneumoniae 1 Klebsiella pneumoniae 1 NA

GCF_002248815 Contig Klebsiella pneumoniae pneumoniae KPH+04 Klebsiella pneumoniae 1 Klebsiella pneumoniae 1 Klebsiella pneumoniae 1 NA

GCF_002248845 Contig Klebsiella pneumoniae pneumoniae KPH+05 Klebsiella pneumoniae 1 Klebsiella pneumoniae 1 Klebsiella pneumoniae 1 NA

GCF_002248945 Contig Klebsiella quasipneumoniae similipneumoniae KPCTRSRTH05 Klebsiella quasipneumoniae 1 Klebsiella quasipneumoniae 1 Klebsiella quasipneumoniae NA

GCF_002248955 Contig Klebsiella pneumoniae pneumoniae KPCTRSRTH09 Klebsiella pneumoniae 1 Klebsiella pneumoniae 1 Klebsiella pneumoniae 1 NA

GCF_002249125 Contig Klebsiella pneumoniae pneumoniae KPPSTH06 Klebsiella pneumoniae 1 Klebsiella pneumoniae 1 Klebsiella pneumoniae 1 NA

GCF_002249165 Contig Klebsiella pneumoniae pneumoniae KPPUTH05 Klebsiella pneumoniae 1 Klebsiella pneumoniae 1 Klebsiella pneumoniae 1 NA

GCF_002249195 Contig Klebsiella pneumoniae pneumoniae KPPSTH08 Klebsiella pneumoniae 1 Klebsiella pneumoniae 1 Klebsiella pneumoniae 1 NA

GCF_002249535 Contig Klebsiella pneumoniae pneumoniae KPPSTH07 Klebsiella pneumoniae 1 Klebsiella pneumoniae 1 Klebsiella pneumoniae 1 NA

GCF_002249975 Complete Klebsiella pneumoniae pneumoniae AUSMDU00008079 Klebsiella pneumoniae 1 Klebsiella pneumoniae 1 Klebsiella pneumoniae 1 NA

GCF_002250985 Scaffold Klebsiella pneumoniae D3-2b-17 Klebsiella pneumoniae 1 Klebsiella pneumoniae 1 Klebsiella pneumoniae 1 NA

GCF_002251715 Contig Klebsiella pneumoniae 3111F Klebsiella pneumoniae 1 Klebsiella pneumoniae 1 Klebsiella pneumoniae 1 NA

GCF_002251875 Complete Klebsiella pneumoniae pneumoniae KPCTRSRTH01 Klebsiella pneumoniae 1 Klebsiella pneumoniae 1 Klebsiella pneumoniae 1 NA

GCF_002251895 Contig Klebsiella pneumoniae pneumoniae KPCRETH31 Klebsiella pneumoniae 1 Klebsiella pneumoniae 1 Klebsiella pneumoniae 1 NA

GCF_002252415 Scaffold Klebsiella pneumoniae SCKP-LL83 Klebsiella pneumoniae 1 Klebsiella pneumoniae 1 Klebsiella pneumoniae 1 NA

GCF_002257765 Complete Klebsiella pneumoniae WCHKP2080 Klebsiella pneumoniae 1 Klebsiella pneumoniae 1 Klebsiella pneumoniae 1 NA

GCF_002257775 Scaffold Klebsiella pneumoniae WCHKP7J8 Klebsiella pneumoniae 1 Klebsiella pneumoniae 1 Klebsiella pneumoniae 1 NA

GCF_002257785 Scaffold Klebsiella pneumoniae WCHKP85522 Klebsiella pneumoniae 1 Klebsiella pneumoniae 1 Klebsiella pneumoniae 1 NA

GCF_002257795 Contig Klebsiella pneumoniae WCHKP1716 Klebsiella pneumoniae 1 Klebsiella pneumoniae 1 Klebsiella pneumoniae 1 NA

GCF_002257845 Scaffold Klebsiella pneumoniae WCHKP1608 Klebsiella pneumoniae 1 Klebsiella pneumoniae 1 Klebsiella pneumoniae 1 NA

GCF_002257855 Scaffold Klebsiella pneumoniae WCHKP9G2 Klebsiella pneumoniae 1 Klebsiella pneumoniae 1 Klebsiella pneumoniae 1 NA

GCF_002257865 Contig Klebsiella pneumoniae WCHKP1368 Klebsiella pneumoniae 1 Klebsiella pneumoniae 1 Klebsiella pneumoniae 1 NA

GCF_002257875 Scaffold Klebsiella pneumoniae WCHKP14G4 Klebsiella pneumoniae 1 Klebsiella pneumoniae 1 Klebsiella pneumoniae 1 NA

GCF_002257925 Scaffold Klebsiella pneumoniae WCHKP13F4 Klebsiella pneumoniae 1 Klebsiella pneumoniae 1 Klebsiella pneumoniae 1 NA

GCF_002257935 Complete Klebsiella pneumoniae WCHKP13F2 Klebsiella pneumoniae 1 Klebsiella pneumoniae 1 Klebsiella pneumoniae 1 NA

GCF_002257965 Scaffold Klebsiella pneumoniae WCHKP10H6 Klebsiella pneumoniae 1 Klebsiella pneumoniae 1 Klebsiella pneumoniae 1 NA

GCF_002257975 Scaffold Klebsiella pneumoniae WCHKP107220 Klebsiella pneumoniae 1 Klebsiella pneumoniae 1 Klebsiella pneumoniae 1 NA

GCF_002258005 Scaffold Klebsiella pneumoniae WCHKP10G4 Klebsiella pneumoniae 1 Klebsiella pneumoniae 1 Klebsiella pneumoniae 1 NA

GCF_002258045 Scaffold Klebsiella pneumoniae WCHKP8D7 Klebsiella pneumoniae 1 Klebsiella pneumoniae 1 Klebsiella pneumoniae 1 NA

GCF_002258055 Complete Klebsiella pneumoniae WCHKP7E2 Klebsiella pneumoniae 1 Klebsiella pneumoniae 1 Klebsiella pneumoniae 1 NA

GCF_002258085 Scaffold Klebsiella pneumoniae WCHKP26 Klebsiella pneumoniae 1 Klebsiella pneumoniae 1 Klebsiella pneumoniae 1 NA

GCF_002258105 Contig Klebsiella pneumoniae WCHKP2005 Klebsiella pneumoniae 1 Klebsiella pneumoniae 1 Klebsiella pneumoniae 1 NA

GCF_002258115 Scaffold Klebsiella pneumoniae WCHKP1548 Klebsiella pneumoniae 1 Klebsiella pneumoniae 1 Klebsiella pneumoniae 1 NA

GCF_002258145 Complete Klebsiella pneumoniae WCHKP8F4 Klebsiella pneumoniae 1 Klebsiella pneumoniae 1 Klebsiella pneumoniae 1 NA

GCF_002258155 Scaffold Klebsiella pneumoniae WCHKP1911 Klebsiella pneumoniae 1 Klebsiella pneumoniae 1 Klebsiella pneumoniae 1 NA

GCF_002258175 Scaffold Klebsiella pneumoniae WCHKP31 Klebsiella pneumoniae 1 Klebsiella pneumoniae 1 Klebsiella pneumoniae 1 NA

GCF_002260725 Contig Klebsiella pneumoniae 40B3 Klebsiella pneumoniae 1 Klebsiella pneumoniae 1 Klebsiella pneumoniae 1 NA

GCF_002260885 Contig Klebsiella pneumoniae 34E1 Klebsiella pneumoniae 1 Klebsiella pneumoniae 1 Klebsiella pneumoniae 1 NA

GCF_002260905 Scaffold Klebsiella pneumoniae 2H7 Klebsiella pneumoniae 1 Klebsiella pneumoniae 1 Klebsiella pneumoniae 1 NA

GCF_002261545 Scaffold Klebsiella pneumoniae L96 Klebsiella pneumoniae 1 Klebsiella pneumoniae 1 Klebsiella pneumoniae 1 NA

GCF_002261575 Contig Klebsiella pneumoniae L84 Klebsiella pneumoniae 1 Klebsiella pneumoniae 1 Klebsiella pneumoniae 1 NA

GCF_002261585 Contig Klebsiella pneumoniae L86 Klebsiella pneumoniae 1 Klebsiella pneumoniae 1 Klebsiella pneumoniae 1 NA

GCF_002261615 Scaffold Klebsiella pneumoniae L81 Klebsiella pneumoniae 1 Klebsiella pneumoniae 1 Klebsiella pneumoniae 1 NA

GCF_002261625 Contig Klebsiella pneumoniae L9 Klebsiella pneumoniae 1 Klebsiella pneumoniae 1 Klebsiella pneumoniae 1 NA

GCF_002261655 Scaffold Klebsiella pneumoniae L70 Klebsiella pneumoniae 1 Klebsiella pneumoniae 1 Klebsiella pneumoniae 1 NA

GCF_002261665 Scaffold Klebsiella pneumoniae L79 Klebsiella pneumoniae 1 Klebsiella pneumoniae 1 Klebsiella pneumoniae 1 NA

GCF_002261675 Scaffold Klebsiella pneumoniae L69 Klebsiella pneumoniae 1 Klebsiella pneumoniae 1 Klebsiella pneumoniae 1 NA

GCF_002261685 Contig Klebsiella pneumoniae L8 Klebsiella pneumoniae 1 Klebsiella pneumoniae 1 Klebsiella pneumoniae 1 NA

GCF_002261735 Contig Klebsiella pneumoniae L61 Klebsiella pneumoniae 1 Klebsiella pneumoniae 1 Klebsiella pneumoniae 1 NA

GCF_002261745 Contig Klebsiella pneumoniae L60 Klebsiella pneumoniae 1 Klebsiella pneumoniae 1 Klebsiella pneumoniae 1 NA

GCF_002261775 Scaffold Klebsiella pneumoniae L58 Klebsiella pneumoniae 1 Klebsiella pneumoniae 1 Klebsiella pneumoniae 1 NA

GCF_002261785 Scaffold Klebsiella pneumoniae L63 Klebsiella pneumoniae 1 Klebsiella pneumoniae 1 Klebsiella pneumoniae 1 NA

GCF_002261815 Contig Klebsiella pneumoniae L528 Klebsiella pneumoniae 1 Klebsiella pneumoniae 1 Klebsiella pneumoniae 1 NA

GCF_002261825 Contig Klebsiella pneumoniae L531 Klebsiella pneumoniae 1 Klebsiella pneumoniae 1 Klebsiella pneumoniae 1 NA

GCF_002261855 Contig Klebsiella pneumoniae L535 Klebsiella pneumoniae 1 Klebsiella pneumoniae 1 Klebsiella pneumoniae 1 NA

GCF_002261875 Contig Klebsiella pneumoniae L529 Klebsiella pneumoniae 1 Klebsiella pneumoniae 1 Klebsiella pneumoniae 1 NA

GCF_002261895 Contig Klebsiella pneumoniae L527 Klebsiella pneumoniae 1 Klebsiella pneumoniae 1 Klebsiella pneumoniae 1 NA

GCF_002261915 Contig Klebsiella pneumoniae L520 Klebsiella pneumoniae 1 Klebsiella pneumoniae 1 Klebsiella pneumoniae 1 NA

GCF_002261935 Contig Klebsiella pneumoniae L526 Klebsiella pneumoniae 1 Klebsiella pneumoniae 1 Klebsiella pneumoniae 1 NA

GCF_002261945 Contig Klebsiella pneumoniae L519 Klebsiella pneumoniae 1 Klebsiella pneumoniae 1 Klebsiella pneumoniae 1 NA

GCF_002261975 Contig Klebsiella pneumoniae L513 Klebsiella pneumoniae 1 Klebsiella pneumoniae 1 Klebsiella pneumoniae 1 NA

GCF_002261985 Contig Klebsiella pneumoniae L505 Klebsiella pneumoniae 1 Klebsiella pneumoniae 1 Klebsiella pneumoniae 1 NA

GCF_002262015 Contig Klebsiella pneumoniae L514 Klebsiella pneumoniae 1 Klebsiella pneumoniae 1 Klebsiella pneumoniae 1 NA

GCF_002262035 Contig Klebsiella pneumoniae L511 Klebsiella pneumoniae 1 Klebsiella pneumoniae 1 Klebsiella pneumoniae 1 NA

GCF_002262055 Contig Klebsiella pneumoniae L48 Klebsiella pneumoniae 1 Klebsiella pneumoniae 1 Klebsiella pneumoniae 1 NA

GCF_002262115 Contig Klebsiella pneumoniae L50 Klebsiella pneumoniae 1 Klebsiella pneumoniae 1 Klebsiella pneumoniae 1 NA

GCF_002262135 Contig Klebsiella pneumoniae L451 Klebsiella pneumoniae 1 Klebsiella pneumoniae 1 Klebsiella pneumoniae 1 NA

GCF_002262145 Contig Klebsiella pneumoniae L447 Klebsiella pneumoniae 1 Klebsiella pneumoniae 1 Klebsiella pneumoniae 1 NA

GCF_002262175 Scaffold Klebsiella pneumoniae L442 Klebsiella pneumoniae 1 Klebsiella pneumoniae 1 Klebsiella pneumoniae 1 NA

GCF_002262185 Contig Klebsiella pneumoniae L462 Klebsiella pneumoniae 1 Klebsiella pneumoniae 1 Klebsiella pneumoniae 1 NA

GCF_002262215 Contig Klebsiella pneumoniae L400 Klebsiella pneumoniae 1 Klebsiella pneumoniae 1 Klebsiella pneumoniae 1 NA

GCF_002262235 Contig Klebsiella pneumoniae L39 Klebsiella pneumoniae 1 Klebsiella pneumoniae 1 Klebsiella pneumoniae 1 NA

GCF_002262275 Contig Klebsiella pneumoniae L440 Klebsiella pneumoniae 1 Klebsiella pneumoniae 1 Klebsiella pneumoniae 1 NA

GCF_002262295 Contig Klebsiella pneumoniae L38 Klebsiella pneumoniae 1 Klebsiella pneumoniae 1 Klebsiella pneumoniae 1 NA

GCF_002262305 Contig Klebsiella pneumoniae L384 Klebsiella pneumoniae 1 Klebsiella pneumoniae 1 Klebsiella pneumoniae 1 NA

GCF_002262335 Scaffold Klebsiella pneumoniae L374 Klebsiella pneumoniae 1 Klebsiella pneumoniae 1 Klebsiella pneumoniae 1 NA

GCF_002262345 Contig Klebsiella pneumoniae L386 Klebsiella pneumoniae 1 Klebsiella pneumoniae 1 Klebsiella pneumoniae 1 NA

GCF_002262375 Contig Klebsiella pneumoniae L31 Klebsiella pneumoniae 1 Klebsiella pneumoniae 1 Klebsiella pneumoniae 1 NA

GCF_002262385 Contig Klebsiella pneumoniae L36 Klebsiella pneumoniae 1 Klebsiella pneumoniae 1 Klebsiella pneumoniae 1 NA

GCF_002262415 Contig Klebsiella pneumoniae L37 Klebsiella pneumoniae 1 Klebsiella pneumoniae 1 Klebsiella pneumoniae 1 NA

GCF_002262425 Contig Klebsiella pneumoniae L350 Klebsiella pneumoniae 1 Klebsiella pneumoniae 1 Klebsiella pneumoniae 1 NA

GCF_002262455 Contig Klebsiella pneumoniae L26 Klebsiella pneumoniae 1 Klebsiella pneumoniae 1 Klebsiella pneumoniae 1 NA

GCF_002262465 Contig Klebsiella pneumoniae L299 Klebsiella pneumoniae 1 Klebsiella pneumoniae 1 Klebsiella pneumoniae 1 NA

GCF_002262475 Contig Klebsiella pneumoniae L256 Klebsiella pneumoniae 1 Klebsiella pneumoniae 1 Klebsiella pneumoniae 1 NA

GCF_002262485 Contig Klebsiella pneumoniae L30 Klebsiella pneumoniae 1 Klebsiella pneumoniae 1 Klebsiella pneumoniae 1 NA

GCF_002262535 Contig Klebsiella pneumoniae L223 Klebsiella pneumoniae 1 Klebsiella pneumoniae 1 Klebsiella pneumoniae 1 NA

GCF_002262545 Contig Klebsiella pneumoniae L211 Klebsiella pneumoniae 1 Klebsiella pneumoniae 1 Klebsiella pneumoniae 1 NA

GCF_002262555 Contig Klebsiella pneumoniae L222 Klebsiella pneumoniae 1 Klebsiella pneumoniae 1 Klebsiella pneumoniae 1 NA

GCF_002262565 Contig Klebsiella pneumoniae L23 Klebsiella pneumoniae 1 Klebsiella pneumoniae 1 Klebsiella pneumoniae 1 NA

GCF_002262615 Contig Klebsiella pneumoniae L182 Klebsiella pneumoniae 1 Klebsiella pneumoniae 1 Klebsiella pneumoniae 1 NA

GCF_002262625 Contig Klebsiella pneumoniae L20 Klebsiella pneumoniae 1 Klebsiella pneumoniae 1 Klebsiella pneumoniae 1 NA

GCF_002262635 Contig Klebsiella pneumoniae L196 Klebsiella pneumoniae 1 Klebsiella pneumoniae 1 Klebsiella pneumoniae 1 NA

GCF_002262695 Contig Klebsiella variicola L18 Klebsiella variicola Klebsiella variicola Klebsiella variicola NA

GCF_002262705 Contig Klebsiella pneumoniae L136 Klebsiella pneumoniae 1 Klebsiella pneumoniae 1 Klebsiella pneumoniae 1 NA

GCF_002262735 Contig Klebsiella pneumoniae L176 Klebsiella pneumoniae 1 Klebsiella pneumoniae 1 Klebsiella pneumoniae 1 NA

GCF_002262745 Contig Klebsiella pneumoniae L142 Klebsiella pneumoniae 1 Klebsiella pneumoniae 1 Klebsiella pneumoniae 1 NA

GCF_002262775 Contig Klebsiella pneumoniae L124 Klebsiella pneumoniae 1 Klebsiella pneumoniae 1 Klebsiella pneumoniae 1 NA

GCF_002262785 Scaffold Klebsiella pneumoniae L111 Klebsiella pneumoniae 1 Klebsiella pneumoniae 1 Klebsiella pneumoniae 1 NA

GCF_002262795 Contig Klebsiella pneumoniae L123 Klebsiella pneumoniae 1 Klebsiella pneumoniae 1 Klebsiella pneumoniae 1 NA

GCF_002262805 Contig Klebsiella pneumoniae L122 Klebsiella pneumoniae 1 Klebsiella pneumoniae 1 Klebsiella pneumoniae 1 NA

GCF_002262855 Contig Klebsiella pneumoniae L117 Klebsiella pneumoniae 1 Klebsiella pneumoniae 1 Klebsiella pneumoniae 1 NA

GCF_002263345 Scaffold Klebsiella pneumoniae SCPM-O-B7954 KPB417/16 Klebsiella pneumoniae 1 Klebsiella pneumoniae 1 Klebsiella pneumoniae 1 NA

GCF_002263375 Scaffold Klebsiella pneumoniae SCPM-O-B8045 KPB1470/16 Klebsiella pneumoniae 1 Klebsiella pneumoniae 1 Klebsiella pneumoniae 1 NA

GCF_002263395 Scaffold Klebsiella pneumoniae SCPM-O-B8044 KPB1617/16 Klebsiella pneumoniae 1 Klebsiella pneumoniae 1 Klebsiella pneumoniae 1 NA

GCF_002264165 Scaffold Klebsiella pneumoniae TRKP1 Klebsiella pneumoniae 1 Klebsiella pneumoniae 1 Klebsiella pneumoniae 1 NA

GCF_002264195 Contig Klebsiella pneumoniae K950 Klebsiella pneumoniae 1 Klebsiella pneumoniae 1 Klebsiella pneumoniae 1 NA

GCF_002264245 Contig Klebsiella pneumoniae K4314 Klebsiella pneumoniae 1 Klebsiella pneumoniae 1 Klebsiella pneumoniae 1 NA

GCF_002264265 Contig Klebsiella pneumoniae K4324 Klebsiella pneumoniae 1 Klebsiella pneumoniae 1 Klebsiella pneumoniae 1 NA

GCF_002264365 Contig Klebsiella pneumoniae K4307 Klebsiella pneumoniae 1 Klebsiella pneumoniae 1 Klebsiella pneumoniae 1 NA

GCF_002264425 Contig Klebsiella pneumoniae K4311 Klebsiella pneumoniae 1 Klebsiella pneumoniae 1 Klebsiella pneumoniae 1 NA

GCF_002264435 Contig Klebsiella pneumoniae K5065 Klebsiella pneumoniae 1 Klebsiella pneumoniae 1 Klebsiella pneumoniae 1 NA

GCF_002264465 Contig Klebsiella pneumoniae K2701 Klebsiella pneumoniae 1 Klebsiella pneumoniae 1 Klebsiella pneumoniae 1 NA

GCF_002264475 Contig Klebsiella pneumoniae K4280 Klebsiella pneumoniae 1 Klebsiella pneumoniae 1 Klebsiella pneumoniae 1 NA

GCF_002264505 Contig Klebsiella pneumoniae K3194 Klebsiella pneumoniae 1 Klebsiella pneumoniae 1 Klebsiella pneumoniae 1 NA

GCF_002264525 Contig Klebsiella pneumoniae K2080 Klebsiella pneumoniae 1 Klebsiella pneumoniae 1 Klebsiella pneumoniae 1 NA

GCF_002264545 Contig Klebsiella pneumoniae K1177 Klebsiella pneumoniae 1 Klebsiella pneumoniae 1 Klebsiella pneumoniae 1 NA

GCF_002264565 Contig Klebsiella variicola K2631 Klebsiella variicola Klebsiella variicola Klebsiella variicola NA

GCF_002264605 Contig Klebsiella pneumoniae K2646 Klebsiella pneumoniae 1 Klebsiella pneumoniae 1 Klebsiella pneumoniae 1 NA

GCF_002264645 Contig Klebsiella pneumoniae K3902 Klebsiella pneumoniae 1 Klebsiella pneumoniae 1 Klebsiella pneumoniae 1 NA

GCF_002264685 Contig Klebsiella pneumoniae K3862 Klebsiella pneumoniae 1 Klebsiella pneumoniae 1 Klebsiella pneumoniae 1 NA

GCF_002264695 Contig Klebsiella pneumoniae K5014 Klebsiella pneumoniae 1 Klebsiella pneumoniae 1 Klebsiella pneumoniae 1 NA

GCF_002264705 Contig Klebsiella pneumoniae K3893 Klebsiella pneumoniae 1 Klebsiella pneumoniae 1 Klebsiella pneumoniae 1 NA

GCF_002264715 Contig Klebsiella pneumoniae K3871 Klebsiella pneumoniae 1 Klebsiella pneumoniae 1 Klebsiella pneumoniae 1 NA

GCF_002264765 Contig Klebsiella pneumoniae K1433 Klebsiella pneumoniae 1 Klebsiella pneumoniae 1 Klebsiella pneumoniae 1 NA

GCF_002264775 Contig Klebsiella pneumoniae K5012 Klebsiella pneumoniae 1 Klebsiella pneumoniae 1 Klebsiella pneumoniae 1 NA

GCF_002264785 Contig Klebsiella pneumoniae K3175 Klebsiella pneumoniae 1 Klebsiella pneumoniae 1 Klebsiella pneumoniae 1 NA

GCF_002264795 Contig Klebsiella pneumoniae K4803 Klebsiella pneumoniae 1 Klebsiella pneumoniae 1 Klebsiella pneumoniae 1 NA

GCF_002264845 Contig Klebsiella pneumoniae K604 Klebsiella pneumoniae 1 Klebsiella pneumoniae 1 Klebsiella pneumoniae 1 NA

GCF_002264855 Contig Klebsiella pneumoniae K2147 Klebsiella pneumoniae 1 Klebsiella pneumoniae 1 Klebsiella pneumoniae 1 NA

GCF_002264905 Contig Klebsiella pneumoniae K669 Klebsiella pneumoniae 1 Klebsiella pneumoniae 1 Klebsiella pneumoniae 1 NA

GCF_002264915 Contig Klebsiella pneumoniae K666 Klebsiella pneumoniae 1 Klebsiella pneumoniae 1 Klebsiella pneumoniae 1 NA

GCF_002264925 Contig Klebsiella pneumoniae K85 Klebsiella pneumoniae 1 Klebsiella pneumoniae 1 Klebsiella pneumoniae 1 NA

GCF_002265015 Contig Klebsiella pneumoniae K3657 Klebsiella pneumoniae 1 Klebsiella pneumoniae 1 Klebsiella pneumoniae 1 NA

GCF_002265085 Contig Klebsiella oxytoca K3678 Klebsiella oxytoca 1 Klebsiella oxytoca 1 Klebsiella oxytoca 1 NA

GCF_002265195 Contig Klebsiella michiganensis K1439 Klebsiella michiganensis 1 Klebsiella michiganensis 1 Klebsiella michiganensis 1 NA

GCF_002265425 Scaffold Klebsiella pneumoniae QJJ29 Klebsiella pneumoniae 1 Klebsiella pneumoniae 1 Klebsiella pneumoniae 1 NA

GCF_002265525 Scaffold Klebsiella pneumoniae QJJ36 Klebsiella pneumoniae 1 Klebsiella pneumoniae 1 Klebsiella pneumoniae 1 NA

GCF_002265565 Scaffold Klebsiella pneumoniae QJJ51 Klebsiella pneumoniae 1 Klebsiella pneumoniae 1 Klebsiella pneumoniae 1 NA

GCF_002265605 Scaffold Klebsiella pneumoniae QJJ49 Klebsiella pneumoniae 1 Klebsiella pneumoniae 1 Klebsiella pneumoniae 1 NA

GCF_002268655 Contig Klebsiella pneumoniae H5 Klebsiella pneumoniae 1 Klebsiella pneumoniae 1 Klebsiella pneumoniae 1 NA

GCF_002268785 Contig Klebsiella pneumoniae C3 Klebsiella pneumoniae 1 Klebsiella pneumoniae 1 Klebsiella pneumoniae 1 NA

GCF_002268825 Contig Klebsiella pneumoniae I2 Klebsiella pneumoniae 1 Klebsiella pneumoniae 1 Klebsiella pneumoniae 1 NA

GCF_002268855 Contig Klebsiella pneumoniae H4 Klebsiella pneumoniae 1 Klebsiella pneumoniae 1 Klebsiella pneumoniae 1 NA

GCF_002268865 Contig Klebsiella pneumoniae F8 Klebsiella pneumoniae 1 Klebsiella pneumoniae 1 Klebsiella pneumoniae 1 NA

GCF_002268925 Contig Klebsiella pneumoniae I1 Klebsiella pneumoniae 1 Klebsiella pneumoniae 1 Klebsiella pneumoniae 1 NA

GCF_002269255 Complete Klebsiella quasivariicola KPN1705 Klebsiella quasivariicola 1 Klebsiella quasivariicola Klebsiella quasivariicola Type strain

GCF_002269295 Complete Klebsiella pneumoniae 911021 Klebsiella pneumoniae 1 Klebsiella pneumoniae 1 Klebsiella pneumoniae 1 NA

GCF_002269805 Scaffold Klebsiella pneumoniae K3113 Klebsiella pneumoniae 1 Klebsiella pneumoniae 1 Klebsiella pneumoniae 1 NA

GCF_002269865 Contig Klebsiella pneumoniae K7118 Klebsiella pneumoniae 1 Klebsiella pneumoniae 1 Klebsiella pneumoniae 1 NA

GCF_002269905 Contig Klebsiella pneumoniae A5 Klebsiella pneumoniae 1 Klebsiella pneumoniae 1 Klebsiella pneumoniae 1 NA

GCF_002269925 Contig Klebsiella pneumoniae A2 Klebsiella pneumoniae 1 Klebsiella pneumoniae 1 Klebsiella pneumoniae 1 NA

GCF_002271285 Contig Klebsiella pneumoniae NU-CRE265 Klebsiella pneumoniae 1 Klebsiella pneumoniae 1 Klebsiella pneumoniae 1 NA

GCF_002271295 Contig Klebsiella pneumoniae NU-CRE212 Klebsiella pneumoniae 1 Klebsiella pneumoniae 1 Klebsiella pneumoniae 1 NA

GCF_002271335 Contig Klebsiella pneumoniae NU-CRE101 Klebsiella pneumoniae 1 Klebsiella pneumoniae 1 Klebsiella pneumoniae 1 NA

GCF_002271345 Contig Klebsiella pneumoniae NU-CRE176 Klebsiella pneumoniae 1 Klebsiella pneumoniae 1 Klebsiella pneumoniae 1 NA

GCF_002278055 Complete Klebsiella pneumoniae 721005 Klebsiella pneumoniae 1 Klebsiella pneumoniae 1 Klebsiella pneumoniae 1 NA

GCF_002283495 Contig Klebsiella pneumoniae GMR-RA45316 Klebsiella pneumoniae 1 Klebsiella pneumoniae 1 Klebsiella pneumoniae 1 NA

GCF_002288845 Scaffold Klebsiella pneumoniae WBB417 Klebsiella quasipneumoniae 1 Klebsiella quasipneumoniae 1 Klebsiella quasipneumoniae NA

GCF_002289105 Scaffold Klebsiella pneumoniae KPC02 Klebsiella pneumoniae 1 Klebsiella pneumoniae 1 Klebsiella pneumoniae 1 NA

GCF_002289165 Scaffold Klebsiella pneumoniae B16 Klebsiella pneumoniae 1 Klebsiella pneumoniae 1 Klebsiella pneumoniae 1 NA

GCF_002290285 Complete Klebsiella michiganensis K518 Klebsiella michiganensis 1 Klebsiella michiganensis 1 Klebsiella michiganensis 1 NA

GCF_002295145 Scaffold Klebsiella pneumoniae B04 Klebsiella pneumoniae 1 Klebsiella pneumoniae 1 Klebsiella pneumoniae 1 NA

GCF_002295185 Scaffold Klebsiella pneumoniae KPC05 Klebsiella pneumoniae 1 Klebsiella pneumoniae 1 Klebsiella pneumoniae 1 NA

GCF_002300675 Complete Klebsiella pneumoniae VBA2172 Klebsiella pneumoniae 1 Klebsiella pneumoniae 1 Klebsiella pneumoniae 1 NA

GCF_002300715 Scaffold Klebsiella pneumoniae B03 Klebsiella pneumoniae 1 Klebsiella pneumoniae 1 Klebsiella pneumoniae 1 NA

GCF_002300725 Scaffold Klebsiella pneumoniae B11 Klebsiella pneumoniae 1 Klebsiella pneumoniae 1 Klebsiella pneumoniae 1 NA

GCF_002300755 Scaffold Klebsiella pneumoniae B29 Klebsiella pneumoniae 1 Klebsiella pneumoniae 1 Klebsiella pneumoniae 1 NA

GCF_002300765 Scaffold Klebsiella pneumoniae B35 Klebsiella pneumoniae 1 Klebsiella pneumoniae 1 Klebsiella pneumoniae 1 NA

GCF_002300775 Scaffold Klebsiella pneumoniae B17 Klebsiella pneumoniae 1 Klebsiella pneumoniae 1 Klebsiella pneumoniae 1 NA

GCF_002300785 Scaffold Klebsiella pneumoniae B30 Klebsiella pneumoniae 1 Klebsiella pneumoniae 1 Klebsiella pneumoniae 1 NA

GCF_002304205 Contig Klebsiella pneumoniae KPN535 Klebsiella pneumoniae 1 Klebsiella pneumoniae 1 Klebsiella pneumoniae 1 NA

GCF_002317885 Contig Klebsiella pneumoniae pneumoniae KPLC+01 Klebsiella pneumoniae 1 Klebsiella pneumoniae 1 Klebsiella pneumoniae 1 NA

GCF_002365935 Contig Klebsiella pneumoniae pneumoniae KPFood+02 Klebsiella pneumoniae 1 Klebsiella pneumoniae 1 Klebsiella pneumoniae 1 NA

GCF_002369875 Scaffold Klebsiella pneumoniae FJ10 Klebsiella pneumoniae 1 Klebsiella pneumoniae 1 Klebsiella pneumoniae 1 NA

GCF_002369905 Scaffold Klebsiella pneumoniae FJ8 Klebsiella pneumoniae 1 Klebsiella pneumoniae 1 Klebsiella pneumoniae 1 NA

GCF_002369925 Contig Klebsiella pneumoniae 1088 Klebsiella pneumoniae 1 Klebsiella pneumoniae 1 Klebsiella pneumoniae 1 NA

GCF_002370065 Scaffold Klebsiella pneumoniae 1 Klebsiella pneumoniae 1 Klebsiella pneumoniae 1 Klebsiella pneumoniae 1 NA

GCF_002374295 Scaffold Klebsiella pneumoniae C1 Klebsiella pneumoniae 1 Klebsiella pneumoniae 1 Klebsiella pneumoniae 1 NA

GCF_002386225 Chromosome Klebsiella pneumoniae FDAARGOS445 Klebsiella pneumoniae 1 Klebsiella pneumoniae 1 Klebsiella pneumoniae 1 NA

GCF_002406585 Scaffold Klebsiella pneumoniae C5 Klebsiella pneumoniae 1 Klebsiella pneumoniae 1 Klebsiella pneumoniae 1 NA

GCF_002406915 Scaffold Klebsiella pneumoniae C16 Klebsiella pneumoniae 1 Klebsiella pneumoniae 1 Klebsiella pneumoniae 1 NA

GCF_002406945 Scaffold Klebsiella pneumoniae C17 Klebsiella pneumoniae 1 Klebsiella pneumoniae 1 Klebsiella pneumoniae 1 NA

GCF_002406965 Scaffold Klebsiella pneumoniae C18 Klebsiella pneumoniae 1 Klebsiella pneumoniae 1 Klebsiella pneumoniae 1 NA

GCF_002406995 Scaffold Klebsiella pneumoniae C22 Klebsiella pneumoniae 1 Klebsiella pneumoniae 1 Klebsiella pneumoniae 1 NA

GCF_002411665 Contig Klebsiella quasipneumoniae 46 Klebsiella quasipneumoniae 1 Klebsiella quasipneumoniae 1 Klebsiella quasipneumoniae NA

GCF_002411715 Contig Klebsiella pneumoniae 183 Klebsiella pneumoniae 1 Klebsiella pneumoniae 1 Klebsiella pneumoniae 1 NA

GCF_002411735 Contig Klebsiella pneumoniae 155 Klebsiella pneumoniae 1 Klebsiella pneumoniae 1 Klebsiella pneumoniae 1 NA

GCF_002411885 Contig Klebsiella pneumoniae 76 Klebsiella pneumoniae 1 Klebsiella pneumoniae 1 Klebsiella pneumoniae 1 NA

GCF_002411905 Contig Klebsiella pneumoniae 117 Klebsiella pneumoniae 1 Klebsiella pneumoniae 1 Klebsiella pneumoniae 1 NA

GCF_002411925 Contig Klebsiella pneumoniae 70 Klebsiella pneumoniae 1 Klebsiella pneumoniae 1 Klebsiella pneumoniae 1 NA

GCF_002412175 Scaffold Klebsiella pneumoniae 96R-Kp Klebsiella pneumoniae 1 Klebsiella pneumoniae 1 Klebsiella pneumoniae 1 NA

GCF_002416615 Contig Klebsiella pneumoniae ST152 K021 Klebsiella pneumoniae 1 Klebsiella pneumoniae 1 Klebsiella pneumoniae 1 NA

GCF_002416655 Contig Klebsiella pneumoniae ST1552 K089 Klebsiella pneumoniae 1 Klebsiella pneumoniae 1 Klebsiella pneumoniae 1 NA

GCF_002416695 Contig Klebsiella pneumoniae ST39 K0179 Klebsiella pneumoniae 1 Klebsiella pneumoniae 1 Klebsiella pneumoniae 1 NA

GCF_002416705 Contig Klebsiella pneumoniae ST15 E041 Klebsiella pneumoniae 1 Klebsiella pneumoniae 1 Klebsiella pneumoniae 1 NA

GCF_002416775 Contig Klebsiella variicola ST1791 KOO1 Klebsiella variicola Klebsiella variicola Klebsiella variicola NA

GCF_002416895 Contig Klebsiella pneumoniae ST1414 K058 Klebsiella pneumoniae 1 Klebsiella pneumoniae 1 Klebsiella pneumoniae 1 NA

GCF_002416985 Contig Klebsiella pneumoniae ST152 KO25 Klebsiella pneumoniae 1 Klebsiella pneumoniae 1 Klebsiella pneumoniae 1 NA

GCF_002416995 Contig Klebsiella pneumoniae ST152 K031 Klebsiella pneumoniae 1 Klebsiella pneumoniae 1 Klebsiella pneumoniae 1 NA

GCF_002417015 Contig Klebsiella pneumoniae ST152 K051 Klebsiella pneumoniae 1 Klebsiella pneumoniae 1 Klebsiella pneumoniae 1 NA

GCF_002417065 Contig Klebsiella pneumoniae ST234 K059 Klebsiella pneumoniae 1 Klebsiella pneumoniae 1 Klebsiella pneumoniae 1 NA

GCF_002417075 Contig Klebsiella pneumoniae ST1414 K078 Klebsiella pneumoniae 1 Klebsiella pneumoniae 1 Klebsiella pneumoniae 1 NA

GCF_002417085 Contig Klebsiella pneumoniae ST323 K090 Klebsiella pneumoniae 1 Klebsiella pneumoniae 1 Klebsiella pneumoniae 1 NA

GCF_002417105 Contig Klebsiella pneumoniae ST1552 K117 Klebsiella pneumoniae 1 Klebsiella pneumoniae 1 Klebsiella pneumoniae 1 NA

GCF_002417145 Contig Klebsiella pneumoniae ST1414 K141 Klebsiella pneumoniae 1 Klebsiella pneumoniae 1 Klebsiella pneumoniae 1 NA

GCF_002417155 Scaffold Klebsiella pneumoniae ST1552 K077 Klebsiella pneumoniae 1 Klebsiella pneumoniae 1 Klebsiella pneumoniae 1 NA

GCF_002417165 Contig Klebsiella pneumoniae ST152 K071 Klebsiella pneumoniae 1 Klebsiella pneumoniae 1 Klebsiella pneumoniae 1 NA

GCF_002417175 Contig Klebsiella pneumoniae ST152 K181 Klebsiella pneumoniae 1 Klebsiella pneumoniae 1 Klebsiella pneumoniae 1 NA

GCF_002417245 Contig Klebsiella pneumoniae ST101 K085 Klebsiella pneumoniae 1 Klebsiella pneumoniae 1 Klebsiella pneumoniae 1 NA

GCF_002417255 Contig Klebsiella pneumoniae ST25 K161 Klebsiella pneumoniae 1 Klebsiella pneumoniae 1 Klebsiella pneumoniae 1 NA

GCF_002417305 Contig Klebsiella pneumoniae ST1552 K061 Klebsiella pneumoniae 1 Klebsiella pneumoniae 1 Klebsiella pneumoniae 1 NA

GCF_002417325 Contig Klebsiella pneumoniae ST1552 K086 Klebsiella pneumoniae 1 Klebsiella pneumoniae 1 Klebsiella pneumoniae 1 NA

GCF_002417375 Contig Klebsiella pneumoniae ST15 K087 Klebsiella pneumoniae 1 Klebsiella pneumoniae 1 Klebsiella pneumoniae 1 NA

GCF_002417385 Contig Klebsiella pneumoniae ST234 K080 Klebsiella pneumoniae 1 Klebsiella pneumoniae 1 Klebsiella pneumoniae 1 NA

GCF_002417395 Contig Klebsiella pneumoniae ST152 K110 Klebsiella pneumoniae 1 Klebsiella pneumoniae 1 Klebsiella pneumoniae 1 NA

GCF_002417435 Contig Klebsiella pneumoniae ST15 K125 Klebsiella pneumoniae 1 Klebsiella pneumoniae 1 Klebsiella pneumoniae 1 NA

GCF_002417455 Contig Klebsiella pneumoniae ST39 K145 Klebsiella pneumoniae 1 Klebsiella pneumoniae 1 Klebsiella pneumoniae 1 NA

GCF_002417545 Contig Klebsiella pneumoniae ST15 K104 Klebsiella pneumoniae 1 Klebsiella pneumoniae 1 Klebsiella pneumoniae 1 NA

GCF_002508265 Contig Klebsiella oxytoca UNM Klebsiella oxytoca 1 Klebsiella oxytoca 1 Klebsiella oxytoca 1 NA

GCF_002508285 Contig Klebsiella pneumoniae Kp1210 Klebsiella pneumoniae 1 Klebsiella pneumoniae 1 Klebsiella pneumoniae 1 NA

GCF_002510065 Contig Klebsiella pneumoniae ST643 K129 Klebsiella pneumoniae 1 Klebsiella pneumoniae 1 Klebsiella pneumoniae 1 NA

GCF_002510095 Contig Klebsiella pneumoniae ST14 K118 Klebsiella pneumoniae 1 Klebsiella pneumoniae 1 Klebsiella pneumoniae 1 NA

GCF_002510145 Contig Klebsiella pneumoniae ST152 K131 Klebsiella pneumoniae 1 Klebsiella pneumoniae 1 Klebsiella pneumoniae 1 NA

GCF_002529065 Contig Klebsiella pneumoniae IBL24 Klebsiella pneumoniae 1 Klebsiella pneumoniae 1 Klebsiella pneumoniae 1 NA

GCF_002529075 Contig Klebsiella pneumoniae KP488 Klebsiella pneumoniae 1 Klebsiella pneumoniae 1 Klebsiella pneumoniae 1 NA

GCF_002556465 Scaffold Klebsiella sp MBT K1 Klebsiella grimontii Klebsiella grimontii Klebsiella grimontii NA

GCF_002559635 Scaffold Klebsiella sp KG9 Klebsiella grimontii Klebsiella grimontii Klebsiella grimontii NA

GCF_002587165 Complete Klebsiella pneumoniae FDAARGOS444 Klebsiella pneumoniae 1 Klebsiella pneumoniae 1 Klebsiella pneumoniae 1 NA

GCF_002587885 Complete Klebsiella pneumoniae FDAARGOS446 Klebsiella pneumoniae 1 Klebsiella pneumoniae 1 Klebsiella pneumoniae 1 NA

GCF_002588345 Contig Klebsiella oxytoca FDAARGOS432 Klebsiella oxytoca 1 Klebsiella oxytoca 1 Klebsiella oxytoca 1 NA

GCF_002588365 Contig Klebsiella pneumoniae FDAARGOS435 Klebsiella pneumoniae 1 Klebsiella pneumoniae 1 Klebsiella pneumoniae 1 NA

GCF_002591075 Complete Klebsiella pneumoniae FDAARGOS439 Klebsiella pneumoniae 1 Klebsiella pneumoniae 1 Klebsiella pneumoniae 1 NA

GCF_002591115 Complete Klebsiella aerogenes FDAARGOS363 Klebsiella aerogenes 1 Klebsiella aerogenes Klebsiella aerogenes NA

GCF_002610865 Scaffold Klebsiella pneumoniae TR214 Klebsiella pneumoniae 1 Klebsiella pneumoniae 1 Klebsiella pneumoniae 1 NA

GCF_002610925 Scaffold Klebsiella pneumoniae TR213 Klebsiella pneumoniae 1 Klebsiella pneumoniae 1 Klebsiella pneumoniae 1 NA

GCF_002610935 Scaffold Klebsiella pneumoniae TR215 Klebsiella pneumoniae 1 Klebsiella pneumoniae 1 Klebsiella pneumoniae 1 NA

GCF_002610945 Scaffold Klebsiella pneumoniae TR221 Klebsiella pneumoniae 1 Klebsiella pneumoniae 1 Klebsiella pneumoniae 1 NA

GCF_002631045 Contig Klebsiella pneumoniae pneumoniae KPBr1 Klebsiella pneumoniae 1 Klebsiella pneumoniae 1 Klebsiella pneumoniae 1 NA

GCF_002635725 Chromosome Klebsiella pneumoniae FDAARGOS440 Klebsiella pneumoniae 1 Klebsiella pneumoniae 1 Klebsiella pneumoniae 1 NA

GCF_002636095 Chromosome Klebsiella pneumoniae FDAARGOS436 Klebsiella pneumoniae 1 Klebsiella pneumoniae 1 Klebsiella pneumoniae 1 NA

GCF_002636215 Chromosome Klebsiella pneumoniae FDAARGOS447 Klebsiella pneumoniae 1 Klebsiella pneumoniae 1 Klebsiella pneumoniae 1 NA

GCF_002636375 Chromosome Klebsiella pneumoniae FDAARGOS442 Klebsiella pneumoniae 1 Klebsiella pneumoniae 1 Klebsiella pneumoniae 1 NA

GCF_002740615 Scaffold Klebsiella pneumoniae XPY20 Klebsiella pneumoniae 1 Klebsiella pneumoniae 1 Klebsiella pneumoniae 1 NA

GCF_002740625 Scaffold Klebsiella pneumoniae XPY166 Klebsiella pneumoniae 1 Klebsiella pneumoniae 1 Klebsiella pneumoniae 1 NA

GCF_002740645 Scaffold Klebsiella pneumoniae XPY207 Klebsiella pneumoniae 1 Klebsiella pneumoniae 1 Klebsiella pneumoniae 1 NA

GCF_002740705 Scaffold Klebsiella pneumoniae XPY193 Klebsiella pneumoniae 1 Klebsiella pneumoniae 1 Klebsiella pneumoniae 1 NA

GCF_002740845 Scaffold Klebsiella variicola 6TM Klebsiella variicola Klebsiella variicola Klebsiella variicola NA

GCF_002740885 Scaffold Klebsiella variicola 1TM Klebsiella variicola Klebsiella variicola Klebsiella variicola NA

GCF_002741685 Complete Klebsiella pneumoniae KSB1 5D Klebsiella pneumoniae 1 Klebsiella pneumoniae 1 Klebsiella pneumoniae 1 NA

GCF_002744555 Contig Klebsiella pneumoniae pneumoniae KPLC+02 Klebsiella pneumoniae 1 Klebsiella pneumoniae 1 Klebsiella pneumoniae 1 NA

GCF_002751155 Contig Klebsiella pneumoniae A1763 Klebsiella pneumoniae 1 Klebsiella pneumoniae 1 Klebsiella pneumoniae 1 NA

GCF_002751175 Scaffold Klebsiella pneumoniae TR197 Klebsiella pneumoniae 1 Klebsiella pneumoniae 1 Klebsiella pneumoniae 1 NA

GCF_002751185 Scaffold Klebsiella pneumoniae TR196 Klebsiella pneumoniae 1 Klebsiella pneumoniae 1 Klebsiella pneumoniae 1 NA

GCF_002751195 Scaffold Klebsiella pneumoniae TR207 Klebsiella pneumoniae 1 Klebsiella pneumoniae 1 Klebsiella pneumoniae 1 NA

GCF_002751235 Scaffold Klebsiella pneumoniae TR210 Klebsiella pneumoniae 1 Klebsiella pneumoniae 1 Klebsiella pneumoniae 1 NA

GCF_002751295 Scaffold Klebsiella pneumoniae TR209 Klebsiella pneumoniae 1 Klebsiella pneumoniae 1 Klebsiella pneumoniae 1 NA

GCF_002751315 Scaffold Klebsiella pneumoniae TR258 Klebsiella pneumoniae 1 Klebsiella pneumoniae 1 Klebsiella pneumoniae 1 NA

GCF_002751325 Scaffold Klebsiella pneumoniae TR231-m Klebsiella pneumoniae 1 Klebsiella pneumoniae 1 Klebsiella pneumoniae 1 NA

GCF_002751355 Scaffold Klebsiella pneumoniae TRqt-43 Klebsiella pneumoniae 1 Klebsiella pneumoniae 1 Klebsiella pneumoniae 1 NA

GCF_002751375 Scaffold Klebsiella pneumoniae TR249 Klebsiella pneumoniae 1 Klebsiella pneumoniae 1 Klebsiella pneumoniae 1 NA

GCF_002751385 Scaffold Klebsiella pneumoniae TR237 Klebsiella pneumoniae 1 Klebsiella pneumoniae 1 Klebsiella pneumoniae 1 NA

GCF_002751415 Scaffold Klebsiella pneumoniae TRqt-47 Klebsiella pneumoniae 1 Klebsiella pneumoniae 1 Klebsiella pneumoniae 1 NA

GCF_002751425 Scaffold Klebsiella pneumoniae TRqt-38 Klebsiella pneumoniae 1 Klebsiella pneumoniae 1 Klebsiella pneumoniae 1 NA

GCF_002751435 Scaffold Klebsiella pneumoniae TRqt-48 Klebsiella pneumoniae 1 Klebsiella pneumoniae 1 Klebsiella pneumoniae 1 NA

GCF_002751475 Scaffold Klebsiella pneumoniae CF119 Klebsiella pneumoniae 1 Klebsiella pneumoniae 1 Klebsiella pneumoniae 1 NA

GCF_002751485 Scaffold Klebsiella pneumoniae TRqt-44 Klebsiella pneumoniae 1 Klebsiella pneumoniae 1 Klebsiella pneumoniae 1 NA

GCF_002751515 Scaffold Klebsiella pneumoniae CF126 Klebsiella pneumoniae 1 Klebsiella pneumoniae 1 Klebsiella pneumoniae 1 NA

GCF_002751525 Scaffold Klebsiella pneumoniae CF127 Klebsiella pneumoniae 1 Klebsiella pneumoniae 1 Klebsiella pneumoniae 1 NA

GCF_002751555 Scaffold Klebsiella pneumoniae CF917 Klebsiella pneumoniae 1 Klebsiella pneumoniae 1 Klebsiella pneumoniae 1 NA

GCF_002751565 Scaffold Klebsiella pneumoniae CF969 Klebsiella pneumoniae 1 Klebsiella pneumoniae 1 Klebsiella pneumoniae 1 NA

GCF_002751595 Scaffold Klebsiella pneumoniae CF125 Klebsiella pneumoniae 1 Klebsiella pneumoniae 1 Klebsiella pneumoniae 1 NA

GCF_002751605 Scaffold Klebsiella pneumoniae CF124 Klebsiella pneumoniae 1 Klebsiella pneumoniae 1 Klebsiella pneumoniae 1 NA

GCF_002751615 Scaffold Klebsiella pneumoniae CF934 Klebsiella pneumoniae 1 Klebsiella pneumoniae 1 Klebsiella pneumoniae 1 NA

GCF_002751645 Scaffold Klebsiella pneumoniae CF987 Klebsiella pneumoniae 1 Klebsiella pneumoniae 1 Klebsiella pneumoniae 1 NA

GCF_002751675 Scaffold Klebsiella pneumoniae A1732 Klebsiella pneumoniae 1 Klebsiella pneumoniae 1 Klebsiella pneumoniae 1 NA

GCF_002751695 Scaffold Klebsiella pneumoniae CF10728 Klebsiella pneumoniae 1 Klebsiella pneumoniae 1 Klebsiella pneumoniae 1 NA

GCF_002751705 Scaffold Klebsiella pneumoniae A1502 Klebsiella pneumoniae 1 Klebsiella pneumoniae 1 Klebsiella pneumoniae 1 NA

GCF_002751715 Scaffold Klebsiella pneumoniae A1674 Klebsiella pneumoniae 1 Klebsiella pneumoniae 1 Klebsiella pneumoniae 1 NA

GCF_002751755 Scaffold Klebsiella pneumoniae A1771 Klebsiella pneumoniae 1 Klebsiella pneumoniae 1 Klebsiella pneumoniae 1 NA

GCF_002751785 Scaffold Klebsiella pneumoniae A1760 Klebsiella pneumoniae 1 Klebsiella pneumoniae 1 Klebsiella pneumoniae 1 NA

GCF_002752775 Complete Klebsiella pneumoniae INF249 Klebsiella pneumoniae 1 Klebsiella pneumoniae 1 Klebsiella pneumoniae 1 NA

GCF_002752865 Scaffold Klebsiella pneumoniae DS32358 14 Klebsiella quasipneumoniae 1 Klebsiella quasipneumoniae 1 Klebsiella quasipneumoniae NA

GCF_002752905 Complete Klebsiella pneumoniae KSB1 7E Klebsiella pneumoniae 1 Klebsiella pneumoniae 1 Klebsiella pneumoniae 1 NA

GCF_002752955 Complete Klebsiella pneumoniae QS17-0161 Klebsiella pneumoniae 1 Klebsiella pneumoniae 1 Klebsiella pneumoniae 1 NA

GCF_002752975 Complete Klebsiella pneumoniae INF278 Klebsiella pneumoniae 1 Klebsiella pneumoniae 1 Klebsiella pneumoniae 1 NA

GCF_002752995 Complete Klebsiella pneumoniae INF042 Klebsiella pneumoniae 1 Klebsiella pneumoniae 1 Klebsiella pneumoniae 1 NA

GCF_002753055 Complete Klebsiella pneumoniae INF158 Klebsiella pneumoniae 1 Klebsiella pneumoniae 1 Klebsiella pneumoniae 1 NA

GCF_002753075 Complete Klebsiella pneumoniae INF157 Klebsiella pneumoniae 1 Klebsiella pneumoniae 1 Klebsiella pneumoniae 1 NA

GCF_002753165 Complete Klebsiella pneumoniae KSB1 9D Klebsiella pneumoniae 1 Klebsiella pneumoniae 1 Klebsiella pneumoniae 1 NA

GCF_002753355 Complete Klebsiella pneumoniae INF059 Klebsiella pneumoniae 1 Klebsiella pneumoniae 1 Klebsiella pneumoniae 1 NA

GCF_002753375 Complete Klebsiella pneumoniae KSB1 7J Klebsiella pneumoniae 1 Klebsiella pneumoniae 1 Klebsiella pneumoniae 1 NA

GCF_002753405 Complete Klebsiella pneumoniae INF163 Klebsiella pneumoniae 1 Klebsiella pneumoniae 1 Klebsiella pneumoniae 1 NA

GCF_002753555 Complete Klebsiella pneumoniae INF164 Klebsiella pneumoniae 1 Klebsiella pneumoniae 1 Klebsiella pneumoniae 1 NA

GCF_002753605 Complete Klebsiella pneumoniae INF274 Klebsiella pneumoniae 1 Klebsiella pneumoniae 1 Klebsiella pneumoniae 1 NA

GCF_002759035 Scaffold Klebsiella pneumoniae LKP723703-1 Klebsiella pneumoniae 1 Klebsiella pneumoniae 1 Klebsiella pneumoniae 1 NA

GCF_002761315 Complete Klebsiella pneumoniae P1428 Klebsiella pneumoniae 1 Klebsiella pneumoniae 1 Klebsiella pneumoniae 1 NA

GCF_002761515 Complete Klebsiella pneumoniae pneumoniae H11 Klebsiella pneumoniae 1 Klebsiella pneumoniae 1 Klebsiella pneumoniae 1 NA

GCF_002775175 Contig Klebsiella pneumoniae AUH-KIMP209 Klebsiella pneumoniae 1 Klebsiella pneumoniae 1 Klebsiella pneumoniae 1 NA

GCF_002775235 Contig Klebsiella pneumoniae AUH-KIMP184 Klebsiella pneumoniae 1 Klebsiella pneumoniae 1 Klebsiella pneumoniae 1 NA

GCF_002775255 Contig Klebsiella pneumoniae AUH-KIMP300 Klebsiella pneumoniae 1 Klebsiella pneumoniae 1 Klebsiella pneumoniae 1 NA

GCF_002775265 Contig Klebsiella pneumoniae AUH-KIMP195 Klebsiella pneumoniae 1 Klebsiella pneumoniae 1 Klebsiella pneumoniae 1 NA

GCF_002775295 Contig Klebsiella pneumoniae AUH-KIMP196 Klebsiella pneumoniae 1 Klebsiella pneumoniae 1 Klebsiella pneumoniae 1 NA

GCF_002775315 Contig Klebsiella pneumoniae AUH-KIMP192 Klebsiella pneumoniae 1 Klebsiella pneumoniae 1 Klebsiella pneumoniae 1 NA

GCF_002775325 Contig Klebsiella pneumoniae AUH-KIMP198 Klebsiella pneumoniae 1 Klebsiella pneumoniae 1 Klebsiella pneumoniae 1 NA

GCF_002775375 Contig Klebsiella pneumoniae AUH-KIMP204 Klebsiella pneumoniae 1 Klebsiella pneumoniae 1 Klebsiella pneumoniae 1 NA

GCF_002775395 Contig Klebsiella pneumoniae AUH-KIMP215 Klebsiella pneumoniae 1 Klebsiella pneumoniae 1 Klebsiella pneumoniae 1 NA

GCF_002775405 Contig Klebsiella pneumoniae AUH-KIMP217 Klebsiella pneumoniae 1 Klebsiella pneumoniae 1 Klebsiella pneumoniae 1 NA

GCF_002775435 Contig Klebsiella quasipneumoniae AUH-KIMP304 Klebsiella quasipneumoniae 1 Klebsiella quasipneumoniae 1 Klebsiella quasipneumoniae NA

GCF_002775445 Contig Klebsiella pneumoniae AUH-KIMP447 Klebsiella pneumoniae 1 Klebsiella pneumoniae 1 Klebsiella pneumoniae 1 NA

GCF_002775455 Contig Klebsiella pneumoniae AUH-KIMP223 Klebsiella pneumoniae 1 Klebsiella pneumoniae 1 Klebsiella pneumoniae 1 NA

GCF_002775495 Contig Klebsiella pneumoniae AUH-KIMP444 Klebsiella pneumoniae 1 Klebsiella pneumoniae 1 Klebsiella pneumoniae 1 NA

GCF_002775515 Contig Klebsiella quasipneumoniae AUH-KIMP307 Klebsiella quasipneumoniae 1 Klebsiella quasipneumoniae 1 Klebsiella quasipneumoniae NA

GCF_002775525 Contig Klebsiella pneumoniae AUH-KIMP328 Klebsiella pneumoniae 1 Klebsiella pneumoniae 1 Klebsiella pneumoniae 1 NA

GCF_002775555 Contig Klebsiella pneumoniae AUH-KIMP407 Klebsiella pneumoniae 1 Klebsiella pneumoniae 1 Klebsiella pneumoniae 1 NA

GCF_002775575 Contig Klebsiella pneumoniae AUH-KIMP297B Klebsiella pneumoniae 1 Klebsiella pneumoniae 1 Klebsiella pneumoniae 1 NA

GCF_002775585 Contig Klebsiella pneumoniae AUH-KIMP460 Klebsiella pneumoniae 1 Klebsiella pneumoniae 1 Klebsiella pneumoniae 1 NA

GCF_002775595 Contig Klebsiella pneumoniae AUH-KIMP461 Klebsiella pneumoniae 1 Klebsiella pneumoniae 1 Klebsiella pneumoniae 1 NA

GCF_002776355 Contig Klebsiella pneumoniae AUH IMP459 Klebsiella pneumoniae 1 Klebsiella pneumoniae 1 Klebsiella pneumoniae 1 NA

GCF_002776375 Contig Klebsiella pneumoniae AUH IMP216 Klebsiella pneumoniae 1 Klebsiella pneumoniae 1 Klebsiella pneumoniae 1 NA

GCF_002776395 Contig Klebsiella pneumoniae AUH IMP485 Klebsiella pneumoniae 1 Klebsiella pneumoniae 1 Klebsiella pneumoniae 1 NA

GCF_002776435 Contig Klebsiella pneumoniae AUH IMP372 Klebsiella pneumoniae 1 Klebsiella pneumoniae 1 Klebsiella pneumoniae 1 NA

GCF_002786755 Complete Klebsiella pneumoniae CRKP1215 Klebsiella pneumoniae 1 Klebsiella pneumoniae 1 Klebsiella pneumoniae 1 NA

GCF_002787755 Complete Klebsiella pneumoniae CRKP2297 Klebsiella pneumoniae 1 Klebsiella pneumoniae 1 Klebsiella pneumoniae 1 NA

GCF_002795445 Contig Klebsiella pneumoniae ID11 Klebsiella pneumoniae 1 Klebsiella pneumoniae 1 Klebsiella pneumoniae 1 NA

GCF_002795525 Contig Klebsiella pneumoniae ID9 Klebsiella pneumoniae 1 Klebsiella pneumoniae 1 Klebsiella pneumoniae 1 NA

GCF_002795535 Contig Klebsiella pneumoniae ID8 Klebsiella pneumoniae 1 Klebsiella pneumoniae 1 Klebsiella pneumoniae 1 NA

GCF_002795575 Contig Klebsiella pneumoniae ID6 Klebsiella pneumoniae 1 Klebsiella pneumoniae 1 Klebsiella pneumoniae 1 NA

GCF_002795605 Contig Klebsiella pneumoniae ID5 Klebsiella pneumoniae 1 Klebsiella pneumoniae 1 Klebsiella pneumoniae 1 NA

GCF_002795635 Contig Klebsiella pneumoniae ID2 Klebsiella pneumoniae 1 Klebsiella pneumoniae 1 Klebsiella pneumoniae 1 NA

GCF_002795665 Contig Klebsiella pneumoniae ID12 Klebsiella pneumoniae 1 Klebsiella pneumoniae 1 Klebsiella pneumoniae 1 NA

GCF_002795755 Scaffold Klebsiella pneumoniae LKP817909 Klebsiella pneumoniae 1 Klebsiella pneumoniae 1 Klebsiella pneumoniae 1 NA

GCF_002796405 Complete Klebsiella aerogenes AR0018 Klebsiella aerogenes 1 Klebsiella aerogenes Klebsiella aerogenes NA

GCF_002796425 Complete Klebsiella aerogenes AR0007 Klebsiella aerogenes 1 Klebsiella aerogenes Klebsiella aerogenes NA

GCF_002796525 Complete Klebsiella aerogenes AR0009 Klebsiella aerogenes 1 Klebsiella aerogenes Klebsiella aerogenes NA

GCF_002806645 Contig Klebsiella sp H-Nf2 Klebsiella variicola Klebsiella variicola Klebsiella variicola NA

GCF_002806655 Contig Klebsiella sp K-Nf6 Klebsiella variicola Klebsiella variicola Klebsiella variicola NA

GCF_002806695 Contig Klebsiella sp I-Nf8 Klebsiella variicola Klebsiella variicola Klebsiella variicola NA

GCF_002807065 Scaffold Klebsiella pneumoniae pneumoniae KP DC Klebsiella pneumoniae 1 Klebsiella pneumoniae 1 Klebsiella pneumoniae 1 NA

GCF_002810475 Contig Klebsiella sp B-Nf7 Klebsiella variicola Klebsiella variicola Klebsiella variicola NA

GCF_002810495 Contig Klebsiella sp C-Nf10 Klebsiella variicola Klebsiella variicola Klebsiella variicola NA

GCF_002810515 Contig Klebsiella sp D-Nf1 Klebsiella variicola Klebsiella variicola Klebsiella variicola NA

GCF_002810535 Contig Klebsiella sp C1-16S-Nf17 Klebsiella variicola Klebsiella variicola Klebsiella variicola NA

GCF_002810545 Scaffold Klebsiella sp A-Nf5 Klebsiella variicola Klebsiella variicola Klebsiella variicola NA

GCF_002810575 Contig Klebsiella sp E-Nf3 Klebsiella variicola Klebsiella variicola Klebsiella variicola NA

GCF_002810595 Contig Klebsiella sp F-Nf9 Klebsiella variicola Klebsiella variicola Klebsiella variicola NA

GCF_002810615 Contig Klebsiella sp G-Nf4 Klebsiella variicola Klebsiella variicola Klebsiella variicola NA

GCF_002810635 Contig Klebsiella sp G2-16S-Nf13 Klebsiella variicola Klebsiella variicola Klebsiella variicola NA

GCF_002811265 Scaffold Klebsiella pneumoniae WCHKP114 Klebsiella pneumoniae 1 Klebsiella pneumoniae 1 Klebsiella pneumoniae 1 NA

GCF_002811275 Scaffold Klebsiella pneumoniae WCHKP110 Klebsiella pneumoniae 1 Klebsiella pneumoniae 1 Klebsiella pneumoniae 1 NA

GCF_002811305 Complete Klebsiella pneumoniae WCHKP020037 Klebsiella pneumoniae 1 Klebsiella pneumoniae 1 Klebsiella pneumoniae 1 NA

GCF_002811325 Complete Klebsiella pneumoniae WCHKP36 Klebsiella pneumoniae 1 Klebsiella pneumoniae 1 Klebsiella pneumoniae 1 NA

GCF_002811335 Complete Klebsiella pneumoniae WCHKP020034 Klebsiella pneumoniae 1 Klebsiella pneumoniae 1 Klebsiella pneumoniae 1 NA

GCF_002811365 Scaffold Klebsiella pneumoniae WCHKP104 Klebsiella pneumoniae 1 Klebsiella pneumoniae 1 Klebsiella pneumoniae 1 NA

GCF_002811375 Scaffold Klebsiella pneumoniae WCHKP96 Klebsiella pneumoniae 1 Klebsiella pneumoniae 1 Klebsiella pneumoniae 1 NA

GCF_002811385 Scaffold Klebsiella pneumoniae WCHKP99 Klebsiella pneumoniae 1 Klebsiella pneumoniae 1 Klebsiella pneumoniae 1 NA

GCF_002811425 Scaffold Klebsiella pneumoniae WCHKP35 Klebsiella pneumoniae 1 Klebsiella pneumoniae 1 Klebsiella pneumoniae 1 NA

GCF_002811525 Scaffold Klebsiella pneumoniae WCHKP1936 Klebsiella pneumoniae 1 Klebsiella pneumoniae 1 Klebsiella pneumoniae 1 NA

GCF_002811535 Scaffold Klebsiella pneumoniae WCHKP1931 Klebsiella pneumoniae 1 Klebsiella pneumoniae 1 Klebsiella pneumoniae 1 NA

GCF_002811585 Scaffold Klebsiella pneumoniae WCHKP1929 Klebsiella pneumoniae 1 Klebsiella pneumoniae 1 Klebsiella pneumoniae 1 NA

GCF_002811615 Scaffold Klebsiella pneumoniae WCHKP105 Klebsiella pneumoniae 1 Klebsiella pneumoniae 1 Klebsiella pneumoniae 1 NA

GCF_002811735 Scaffold Klebsiella pneumoniae WCHKP118 Klebsiella pneumoniae 1 Klebsiella pneumoniae 1 Klebsiella pneumoniae 1 NA

GCF_002812365 Complete Klebsiella pneumoniae AUSMDU00003562 Klebsiella pneumoniae 1 Klebsiella pneumoniae 1 Klebsiella pneumoniae 1 NA

GCF_002812385 Complete Klebsiella pneumoniae AUSMDU00008119 Klebsiella pneumoniae 1 Klebsiella pneumoniae 1 Klebsiella pneumoniae 1 NA

GCF_002812665 Complete Klebsiella pneumoniae NU-CRE047 Klebsiella pneumoniae 1 Klebsiella pneumoniae 1 Klebsiella pneumoniae 1 NA

GCF_002813595 Complete Klebsiella pneumoniae SGH10 Klebsiella pneumoniae 1 Klebsiella pneumoniae 1 Klebsiella pneumoniae 1 NA

GCF_002814015 Complete Klebsiella pneumoniae 459 Klebsiella pneumoniae 1 Klebsiella pneumoniae 1 Klebsiella pneumoniae 1 NA

GCF_002831525 Chromosome Klebsiella pneumoniae KP6 Klebsiella pneumoniae 1 Klebsiella pneumoniae 1 Klebsiella pneumoniae 1 NA

GCF_002833425 Complete Klebsiella pneumoniae NR5632 Klebsiella pneumoniae 1 Klebsiella pneumoniae 1 Klebsiella pneumoniae 1 NA

GCF_002833445 Complete Klebsiella pneumoniae KP1768 Klebsiella pneumoniae 1 Klebsiella pneumoniae 1 Klebsiella pneumoniae 1 NA

GCF_002833465 Complete Klebsiella pneumoniae KP1766 Klebsiella pneumoniae 1 Klebsiella pneumoniae 1 Klebsiella pneumoniae 1 NA

GCF_002834285 Contig Klebsiella pneumoniae 1ECKPC Klebsiella pneumoniae 1 Klebsiella pneumoniae 1 Klebsiella pneumoniae 1 NA

GCF_002834545 Contig Klebsiella pneumoniae LIM944 Klebsiella pneumoniae 1 Klebsiella pneumoniae 1 Klebsiella pneumoniae 1 NA

GCF_002837615 Scaffold Klebsiella sp X1-16S-Nf21 Klebsiella variicola Klebsiella variicola Klebsiella variicola NA

GCF_002837625 Contig Klebsiella sp T11 Klebsiella variicola Klebsiella variicola Klebsiella variicola NA

GCF_002837655 Contig Klebsiella sp J-Nf11 Klebsiella variicola Klebsiella variicola Klebsiella variicola NA

GCF_002838125 Contig Klebsiella pneumoniae KP05-2017 Klebsiella pneumoniae 1 Klebsiella pneumoniae 1 Klebsiella pneumoniae 1 NA

GCF_002844865 Scaffold Klebsiella pneumoniae BJK20 Klebsiella pneumoniae 1 Klebsiella pneumoniae 1 Klebsiella pneumoniae 1 NA

GCF_002844875 Scaffold Klebsiella pneumoniae BJK22 Klebsiella pneumoniae 1 Klebsiella pneumoniae 1 Klebsiella pneumoniae 1 NA

GCF_002844905 Scaffold Klebsiella pneumoniae AH9 Klebsiella pneumoniae 1 Klebsiella pneumoniae 1 Klebsiella pneumoniae 1 NA

GCF_002844925 Scaffold Klebsiella pneumoniae BJK23 Klebsiella pneumoniae 1 Klebsiella pneumoniae 1 Klebsiella pneumoniae 1 NA

GCF_002844935 Scaffold Klebsiella pneumoniae GD108 Klebsiella pneumoniae 1 Klebsiella pneumoniae 1 Klebsiella pneumoniae 1 NA

GCF_002844965 Scaffold Klebsiella pneumoniae AH29 Klebsiella pneumoniae 1 Klebsiella pneumoniae 1 Klebsiella pneumoniae 1 NA

GCF_002844975 Scaffold Klebsiella pneumoniae SH16 Klebsiella pneumoniae 1 Klebsiella pneumoniae 1 Klebsiella pneumoniae 1 NA

GCF_002844985 Scaffold Klebsiella pneumoniae XJ126 Klebsiella pneumoniae 1 Klebsiella pneumoniae 1 Klebsiella pneumoniae 1 NA

GCF_002844995 Scaffold Klebsiella pneumoniae SH2 Klebsiella pneumoniae 1 Klebsiella pneumoniae 1 Klebsiella pneumoniae 1 NA

GCF_002845045 Scaffold Klebsiella pneumoniae HA74 Klebsiella pneumoniae 1 Klebsiella pneumoniae 1 Klebsiella pneumoniae 1 NA

GCF_002845065 Scaffold Klebsiella pneumoniae HA99 Klebsiella pneumoniae 1 Klebsiella pneumoniae 1 Klebsiella pneumoniae 1 NA

GCF_002845075 Scaffold Klebsiella pneumoniae SH9 Klebsiella pneumoniae 1 Klebsiella pneumoniae 1 Klebsiella pneumoniae 1 NA

GCF_002845095 Scaffold Klebsiella pneumoniae HA2-17 Klebsiella pneumoniae 1 Klebsiella pneumoniae 1 Klebsiella pneumoniae 1 NA

GCF_002845125 Scaffold Klebsiella pneumoniae HN34-1 Klebsiella pneumoniae 1 Klebsiella pneumoniae 1 Klebsiella pneumoniae 1 NA

GCF_002845135 Scaffold Klebsiella pneumoniae GD-KPC30 Klebsiella pneumoniae 1 Klebsiella pneumoniae 1 Klebsiella pneumoniae 1 NA

GCF_002845145 Scaffold Klebsiella pneumoniae JXR113 Klebsiella pneumoniae 1 Klebsiella pneumoniae 1 Klebsiella pneumoniae 1 NA

GCF_002845185 Scaffold Klebsiella pneumoniae HN34-2 Klebsiella pneumoniae 1 Klebsiella pneumoniae 1 Klebsiella pneumoniae 1 NA

GCF_002845205 Scaffold Klebsiella pneumoniae BJK8 Klebsiella pneumoniae 1 Klebsiella pneumoniae 1 Klebsiella pneumoniae 1 NA

GCF_002845225 Scaffold Klebsiella pneumoniae FJ11 Klebsiella pneumoniae 1 Klebsiella pneumoniae 1 Klebsiella pneumoniae 1 NA

GCF_002845235 Scaffold Klebsiella pneumoniae BJK7 Klebsiella pneumoniae 1 Klebsiella pneumoniae 1 Klebsiella pneumoniae 1 NA

GCF_002845265 Scaffold Klebsiella pneumoniae BJ-A1040 Klebsiella pneumoniae 1 Klebsiella pneumoniae 1 Klebsiella pneumoniae 1 NA

GCF_002845285 Scaffold Klebsiella pneumoniae JXR114 Klebsiella pneumoniae 1 Klebsiella pneumoniae 1 Klebsiella pneumoniae 1 NA

GCF_002845315 Scaffold Klebsiella pneumoniae BJK6 Klebsiella pneumoniae 1 Klebsiella pneumoniae 1 Klebsiella pneumoniae 1 NA

GCF_002845345 Scaffold Klebsiella pneumoniae AH10 Klebsiella pneumoniae 1 Klebsiella pneumoniae 1 Klebsiella pneumoniae 1 NA

GCF_002845865 Chromosome Klebsiella pneumoniae KP8 Klebsiella pneumoniae 1 Klebsiella pneumoniae 1 Klebsiella pneumoniae 1 NA

GCF_002845885 Chromosome Klebsiella pneumoniae KP11 Klebsiella pneumoniae 1 Klebsiella pneumoniae 1 Klebsiella pneumoniae 1 NA

GCF_002845905 Chromosome Klebsiella pneumoniae KP9 Klebsiella pneumoniae 1 Klebsiella pneumoniae 1 Klebsiella pneumoniae 1 NA

GCF_002845925 Chromosome Klebsiella pneumoniae KP10 Klebsiella pneumoniae 1 Klebsiella pneumoniae 1 Klebsiella pneumoniae 1 NA

GCF_002845945 Chromosome Klebsiella pneumoniae KP14 Klebsiella pneumoniae 1 Klebsiella pneumoniae 1 Klebsiella pneumoniae 1 NA

GCF_002845965 Chromosome Klebsiella pneumoniae KP7 Klebsiella pneumoniae 1 Klebsiella pneumoniae 1 Klebsiella pneumoniae 1 NA

GCF_002848545 Complete Klebsiella pneumoniae KP69 Klebsiella pneumoniae 1 Klebsiella pneumoniae 1 Klebsiella pneumoniae 1 NA

GCF_002848565 Complete Klebsiella pneumoniae JS187 Klebsiella pneumoniae 1 Klebsiella pneumoniae 1 Klebsiella pneumoniae 1 NA

GCF_002848585 Complete Klebsiella pneumoniae F44 Klebsiella pneumoniae 1 Klebsiella pneumoniae 1 Klebsiella pneumoniae 1 NA

GCF_002848835 Scaffold Klebsiella pneumoniae BD DM413 Klebsiella pneumoniae 1 Klebsiella pneumoniae 1 Klebsiella pneumoniae 1 NA

GCF_002849555 Contig Klebsiella aerogenes Z34975 Klebsiella aerogenes 1 Klebsiella aerogenes Klebsiella aerogenes NA

GCF_002850275 Complete Klebsiella pneumoniae 2N3 Klebsiella pneumoniae 1 Klebsiella pneumoniae 1 Klebsiella pneumoniae 1 NA

GCF_002850335 Chromosome Klebsiella pneumoniae DT1 Klebsiella pneumoniae 1 Klebsiella pneumoniae 1 Klebsiella pneumoniae 1 NA

GCF_002850355 Chromosome Klebsiella pneumoniae DT12 Klebsiella pneumoniae 1 Klebsiella pneumoniae 1 Klebsiella pneumoniae 1 NA

GCF_002850595 Contig Klebsiella pneumoniae pneumoniae SCKP020077 Klebsiella pneumoniae 1 Klebsiella pneumoniae 1 Klebsiella pneumoniae 1 NA

GCF_002850635 Contig Klebsiella pneumoniae pneumoniae WCHKP015148 Klebsiella pneumoniae 1 Klebsiella pneumoniae 1 Klebsiella pneumoniae 1 NA

GCF_002850795 Contig Klebsiella pneumoniae COL-Kpn42 Klebsiella pneumoniae 1 Klebsiella pneumoniae 1 Klebsiella pneumoniae 1 NA

GCF_002850805 Contig Klebsiella pneumoniae COL-Kpn41 Klebsiella pneumoniae 1 Klebsiella pneumoniae 1 Klebsiella pneumoniae 1 NA

GCF_002850835 Contig Klebsiella pneumoniae COL-Kpn47 Klebsiella pneumoniae 1 Klebsiella pneumoniae 1 Klebsiella pneumoniae 1 NA

GCF_002850845 Contig Klebsiella pneumoniae COL-Kpn40 Klebsiella pneumoniae 1 Klebsiella pneumoniae 1 Klebsiella pneumoniae 1 NA

GCF_002850875 Contig Klebsiella pneumoniae COL-Kpn39 Klebsiella pneumoniae 1 Klebsiella pneumoniae 1 Klebsiella pneumoniae 1 NA

GCF_002850885 Contig Klebsiella pneumoniae COL-Kpn34 Klebsiella pneumoniae 1 Klebsiella pneumoniae 1 Klebsiella pneumoniae 1 NA

GCF_002850915 Contig Klebsiella pneumoniae COL-Kpn32 Klebsiella pneumoniae 1 Klebsiella pneumoniae 1 Klebsiella pneumoniae 1 NA

GCF_002850935 Contig Klebsiella pneumoniae COL-Kpn33 Klebsiella pneumoniae 1 Klebsiella pneumoniae 1 Klebsiella pneumoniae 1 NA

GCF_002850955 Contig Klebsiella pneumoniae COL-Kpn30 Klebsiella pneumoniae 1 Klebsiella pneumoniae 1 Klebsiella pneumoniae 1 NA

GCF_002850965 Contig Klebsiella pneumoniae COL-Kpn29 Klebsiella pneumoniae 1 Klebsiella pneumoniae 1 Klebsiella pneumoniae 1 NA

GCF_002850995 Contig Klebsiella pneumoniae COL-Kpn28 Klebsiella pneumoniae 1 Klebsiella pneumoniae 1 Klebsiella pneumoniae 1 NA

GCF_002851015 Contig Klebsiella pneumoniae COL-Kpn27 Klebsiella pneumoniae 1 Klebsiella pneumoniae 1 Klebsiella pneumoniae 1 NA

GCF_002851035 Contig Klebsiella pneumoniae COL-Kpn20 Klebsiella pneumoniae 1 Klebsiella pneumoniae 1 Klebsiella pneumoniae 1 NA

GCF_002851055 Contig Klebsiella pneumoniae COL-Kpn26 Klebsiella pneumoniae 1 Klebsiella pneumoniae 1 Klebsiella pneumoniae 1 NA

GCF_002851075 Contig Klebsiella quasipneumoniae COL-Kpn19 Klebsiella quasipneumoniae 1 Klebsiella quasipneumoniae 1 Klebsiella quasipneumoniae NA

GCF_002851085 Contig Klebsiella quasipneumoniae COL-Kpn18 Klebsiella quasipneumoniae 2 Klebsiella quasipneumoniae 2 Klebsiella quasipneumoniae NA

GCF_002851115 Contig Klebsiella pneumoniae COL-Kpn17 Klebsiella pneumoniae 1 Klebsiella pneumoniae 1 Klebsiella pneumoniae 1 NA

GCF_002851135 Contig Klebsiella pneumoniae COL-Kpn16 Klebsiella pneumoniae 1 Klebsiella pneumoniae 1 Klebsiella pneumoniae 1 NA

GCF_002851155 Contig Klebsiella pneumoniae COL-Kpn15 Klebsiella pneumoniae 1 Klebsiella pneumoniae 1 Klebsiella pneumoniae 1 NA

GCF_002851175 Contig Klebsiella pneumoniae COL-Kpn14 Klebsiella pneumoniae 1 Klebsiella pneumoniae 1 Klebsiella pneumoniae 1 NA

GCF_002851195 Contig Klebsiella pneumoniae COL-Kpn13 Klebsiella pneumoniae 1 Klebsiella pneumoniae 1 Klebsiella pneumoniae 1 NA

GCF_002851215 Contig Klebsiella pneumoniae COL-Kpn12 Klebsiella pneumoniae 1 Klebsiella pneumoniae 1 Klebsiella pneumoniae 1 NA

GCF_002851235 Contig Klebsiella quasipneumoniae COL-Kpn11 Klebsiella quasipneumoniae 1 Klebsiella quasipneumoniae 1 Klebsiella quasipneumoniae NA

GCF_002851245 Contig Klebsiella pneumoniae COL-Kpn10 Klebsiella pneumoniae 1 Klebsiella pneumoniae 1 Klebsiella pneumoniae 1 NA

GCF_002851275 Contig Klebsiella pneumoniae COL-Kpn9 Klebsiella pneumoniae 1 Klebsiella pneumoniae 1 Klebsiella pneumoniae 1 NA

GCF_002851295 Contig Klebsiella quasipneumoniae COL-Kpn6 Klebsiella quasipneumoniae 2 Klebsiella quasipneumoniae 2 Klebsiella quasipneumoniae NA

GCF_002851315 Contig Klebsiella quasipneumoniae COL-Kpn1B Klebsiella quasipneumoniae 2 Klebsiella quasipneumoniae 2 Klebsiella quasipneumoniae NA

GCF_002851335 Contig Klebsiella pneumoniae COL-Kpn3 Klebsiella pneumoniae 1 Klebsiella pneumoniae 1 Klebsiella pneumoniae 1 NA

GCF_002851345 Contig Klebsiella pneumoniae COL-Kpn43 Klebsiella pneumoniae 1 Klebsiella pneumoniae 1 Klebsiella pneumoniae 1 NA

GCF_002851375 Contig Klebsiella pneumoniae COL-Kpn38 Klebsiella pneumoniae 1 Klebsiella pneumoniae 1 Klebsiella pneumoniae 1 NA

GCF_002851395 Contig Klebsiella pneumoniae COL-Kpn37 Klebsiella pneumoniae 1 Klebsiella pneumoniae 1 Klebsiella pneumoniae 1 NA

GCF_002851415 Contig Klebsiella variicola COL-Kpn36 Klebsiella variicola Klebsiella variicola Klebsiella variicola NA

GCF_002851435 Contig Klebsiella pneumoniae COL-Kpn35 Klebsiella pneumoniae 1 Klebsiella pneumoniae 1 Klebsiella pneumoniae 1 NA

GCF_002851455 Contig Klebsiella pneumoniae COL-Kpn31 Klebsiella pneumoniae 1 Klebsiella pneumoniae 1 Klebsiella pneumoniae 1 NA

GCF_002851475 Contig Klebsiella pneumoniae COL-Kpn25 Klebsiella pneumoniae 1 Klebsiella pneumoniae 1 Klebsiella pneumoniae 1 NA

GCF_002851495 Contig Klebsiella pneumoniae COL-Kpn24 Klebsiella pneumoniae 1 Klebsiella pneumoniae 1 Klebsiella pneumoniae 1 NA

GCF_002851515 Contig Klebsiella pneumoniae COL-Kpn22 Klebsiella pneumoniae 1 Klebsiella pneumoniae 1 Klebsiella pneumoniae 1 NA

GCF_002851525 Contig Klebsiella pneumoniae COL-Kpn23 Klebsiella pneumoniae 1 Klebsiella pneumoniae 1 Klebsiella pneumoniae 1 NA

GCF_002851555 Contig Klebsiella pneumoniae COL-Kpn21 Klebsiella pneumoniae 1 Klebsiella pneumoniae 1 Klebsiella pneumoniae 1 NA

GCF_002851575 Contig Klebsiella pneumoniae COL-Kpn8 Klebsiella pneumoniae 1 Klebsiella pneumoniae 1 Klebsiella pneumoniae 1 NA

GCF_002851595 Contig Klebsiella quasipneumoniae COL-Kpn7 Klebsiella quasipneumoniae 1 Klebsiella quasipneumoniae 1 Klebsiella quasipneumoniae NA

GCF_002851615 Contig Klebsiella pneumoniae COL-Kpn5 Klebsiella pneumoniae 1 Klebsiella pneumoniae 1 Klebsiella pneumoniae 1 NA

GCF_002851635 Contig Klebsiella variicola COL-Kpn2 Klebsiella variicola Klebsiella variicola Klebsiella variicola NA

GCF_002851855 Scaffold Klebsiella pneumoniae ozaenae WCHKP030209 Klebsiella pneumoniae 1 Klebsiella pneumoniae 1 Klebsiella pneumoniae 1 NA

GCF_002851875 Scaffold Klebsiella pneumoniae pneumoniae WCHKP015144 Klebsiella pneumoniae 1 Klebsiella pneumoniae 1 Klebsiella pneumoniae 1 NA

GCF_002851905 Scaffold Klebsiella pneumoniae pneumoniae WCHKP015138 Klebsiella pneumoniae 1 Klebsiella pneumoniae 1 Klebsiella pneumoniae 1 NA

GCF_002851935 Scaffold Klebsiella pneumoniae pneumoniae WCHKP015134 Klebsiella pneumoniae 1 Klebsiella pneumoniae 1 Klebsiella pneumoniae 1 NA

GCF_002851955 Scaffold Klebsiella pneumoniae pneumoniae WCHKP015133 Klebsiella pneumoniae 1 Klebsiella pneumoniae 1 Klebsiella pneumoniae 1 NA

GCF_002851975 Scaffold Klebsiella pneumoniae pneumoniae WCHKP015132 Klebsiella pneumoniae 1 Klebsiella pneumoniae 1 Klebsiella pneumoniae 1 NA

GCF_002851995 Scaffold Klebsiella pneumoniae pneumoniae WCHKP015131 Klebsiella pneumoniae 1 Klebsiella pneumoniae 1 Klebsiella pneumoniae 1 NA

GCF_002852015 Scaffold Klebsiella pneumoniae pneumoniae WCHKP015123 Klebsiella pneumoniae 1 Klebsiella pneumoniae 1 Klebsiella pneumoniae 1 NA

GCF_002852035 Scaffold Klebsiella pneumoniae ozaenae WCHKP030795 Klebsiella pneumoniae 1 Klebsiella pneumoniae 1 Klebsiella pneumoniae 1 NA

GCF_002852055 Scaffold Klebsiella pneumoniae pneumoniae WCHKP015121 Klebsiella pneumoniae 1 Klebsiella pneumoniae 1 Klebsiella pneumoniae 1 NA

GCF_002852065 Scaffold Klebsiella pneumoniae ozaenae WCHKP030775 Klebsiella pneumoniae 1 Klebsiella pneumoniae 1 Klebsiella pneumoniae 1 NA

GCF_002852095 Scaffold Klebsiella pneumoniae pneumoniae WCHKP015116 Klebsiella pneumoniae 1 Klebsiella pneumoniae 1 Klebsiella pneumoniae 1 NA

GCF_002852105 Scaffold Klebsiella pneumoniae pneumoniae WCHKP015114 Klebsiella pneumoniae 1 Klebsiella pneumoniae 1 Klebsiella pneumoniae 1 NA

GCF_002852135 Scaffold Klebsiella pneumoniae pneumoniae WCHKP015113 Klebsiella pneumoniae 1 Klebsiella pneumoniae 1 Klebsiella pneumoniae 1 NA

GCF_002852155 Scaffold Klebsiella pneumoniae pneumoniae WCHKP015112 Klebsiella pneumoniae 1 Klebsiella pneumoniae 1 Klebsiella pneumoniae 1 NA

GCF_002852175 Scaffold Klebsiella pneumoniae pneumoniae WCHKP015111 Klebsiella pneumoniae 1 Klebsiella pneumoniae 1 Klebsiella pneumoniae 1 NA

GCF_002852195 Scaffold Klebsiella pneumoniae pneumoniae WCHKP015110 Klebsiella pneumoniae 1 Klebsiella pneumoniae 1 Klebsiella pneumoniae 1 NA

GCF_002852215 Scaffold Klebsiella pneumoniae pneumoniae WCHKP015109 Klebsiella pneumoniae 1 Klebsiella pneumoniae 1 Klebsiella pneumoniae 1 NA

GCF_002852235 Scaffold Klebsiella pneumoniae pneumoniae WCHKP015107 Klebsiella pneumoniae 1 Klebsiella pneumoniae 1 Klebsiella pneumoniae 1 NA

GCF_002852245 Scaffold Klebsiella pneumoniae pneumoniae WCHKP015104 Klebsiella pneumoniae 1 Klebsiella pneumoniae 1 Klebsiella pneumoniae 1 NA

GCF_002852275 Scaffold Klebsiella pneumoniae pneumoniae WCHKP015103 Klebsiella pneumoniae 1 Klebsiella pneumoniae 1 Klebsiella pneumoniae 1 NA

GCF_002852285 Scaffold Klebsiella pneumoniae pneumoniae WCHKP015099 Klebsiella pneumoniae 1 Klebsiella pneumoniae 1 Klebsiella pneumoniae 1 NA

GCF_002852315 Scaffold Klebsiella pneumoniae pneumoniae WCHKP015098 Klebsiella pneumoniae 1 Klebsiella pneumoniae 1 Klebsiella pneumoniae 1 NA

GCF_002852325 Scaffold Klebsiella pneumoniae pneumoniae WCHKP015096 Klebsiella pneumoniae 1 Klebsiella pneumoniae 1 Klebsiella pneumoniae 1 NA

GCF_002852355 Scaffold Klebsiella pneumoniae pneumoniae WCHKP015095 Klebsiella pneumoniae 1 Klebsiella pneumoniae 1 Klebsiella pneumoniae 1 NA

GCF_002852365 Complete Klebsiella pneumoniae pneumoniae WCHKP015093 Klebsiella pneumoniae 1 Klebsiella pneumoniae 1 Klebsiella pneumoniae 1 NA

GCF_002852395 Scaffold Klebsiella pneumoniae pneumoniae WCHKP015091 Klebsiella pneumoniae 1 Klebsiella pneumoniae 1 Klebsiella pneumoniae 1 NA

GCF_002852415 Scaffold Klebsiella pneumoniae pneumoniae WCHKP015085 Klebsiella pneumoniae 1 Klebsiella pneumoniae 1 Klebsiella pneumoniae 1 NA

GCF_002852435 Scaffold Klebsiella pneumoniae pneumoniae WCHKP015079 Klebsiella pneumoniae 1 Klebsiella pneumoniae 1 Klebsiella pneumoniae 1 NA

GCF_002852455 Scaffold Klebsiella pneumoniae pneumoniae WCHKP015080 Klebsiella pneumoniae 1 Klebsiella pneumoniae 1 Klebsiella pneumoniae 1 NA

GCF_002852475 Scaffold Klebsiella pneumoniae pneumoniae WCHKP015078 Klebsiella pneumoniae 1 Klebsiella pneumoniae 1 Klebsiella pneumoniae 1 NA

GCF_002852485 Scaffold Klebsiella pneumoniae pneumoniae WCHKP015076 Klebsiella pneumoniae 1 Klebsiella pneumoniae 1 Klebsiella pneumoniae 1 NA

GCF_002852515 Scaffold Klebsiella pneumoniae pneumoniae WCHKP015075 Klebsiella pneumoniae 1 Klebsiella pneumoniae 1 Klebsiella pneumoniae 1 NA

GCF_002852535 Scaffold Klebsiella pneumoniae pneumoniae WCHKP015073 Klebsiella pneumoniae 1 Klebsiella pneumoniae 1 Klebsiella pneumoniae 1 NA

GCF_002852545 Scaffold Klebsiella pneumoniae pneumoniae WCHKP020133 Klebsiella pneumoniae 1 Klebsiella pneumoniae 1 Klebsiella pneumoniae 1 NA

GCF_002852575 Scaffold Klebsiella pneumoniae pneumoniae WCHKP020132 Klebsiella pneumoniae 1 Klebsiella pneumoniae 1 Klebsiella pneumoniae 1 NA

GCF_002852585 Scaffold Klebsiella pneumoniae pneumoniae WCHKP020131 Klebsiella pneumoniae 1 Klebsiella pneumoniae 1 Klebsiella pneumoniae 1 NA

GCF_002852615 Scaffold Klebsiella pneumoniae ozaenae WCHKP030543 Klebsiella pneumoniae 1 Klebsiella pneumoniae 1 Klebsiella pneumoniae 1 NA

GCF_002852635 Scaffold Klebsiella pneumoniae pneumoniae WCHKP020128 Klebsiella pneumoniae 1 Klebsiella pneumoniae 1 Klebsiella pneumoniae 1 NA

GCF_002852675 Scaffold Klebsiella pneumoniae pneumoniae WCHKP020130 Klebsiella pneumoniae 1 Klebsiella pneumoniae 1 Klebsiella pneumoniae 1 NA

GCF_002852695 Scaffold Klebsiella pneumoniae pneumoniae WCHKP020126 Klebsiella pneumoniae 1 Klebsiella pneumoniae 1 Klebsiella pneumoniae 1 NA

GCF_002852715 Scaffold Klebsiella pneumoniae pneumoniae WCHKP020125 Klebsiella pneumoniae 1 Klebsiella pneumoniae 1 Klebsiella pneumoniae 1 NA

GCF_002852735 Scaffold Klebsiella pneumoniae pneumoniae WCHKP020124 Klebsiella pneumoniae 1 Klebsiella pneumoniae 1 Klebsiella pneumoniae 1 NA

GCF_002852755 Scaffold Klebsiella pneumoniae pneumoniae WCHKP020106 Klebsiella pneumoniae 1 Klebsiella pneumoniae 1 Klebsiella pneumoniae 1 NA

GCF_002852775 Scaffold Klebsiella pneumoniae pneumoniae WCHKP020111 Klebsiella pneumoniae 1 Klebsiella pneumoniae 1 Klebsiella pneumoniae 1 NA

GCF_002852785 Complete Klebsiella pneumoniae pneumoniae WCHKP020120 Klebsiella pneumoniae 1 Klebsiella pneumoniae 1 Klebsiella pneumoniae 1 NA

GCF_002852815 Scaffold Klebsiella pneumoniae pneumoniae SCKP020060 Klebsiella pneumoniae 1 Klebsiella pneumoniae 1 Klebsiella pneumoniae 1 NA

GCF_002852825 Scaffold Klebsiella pneumoniae pneumoniae SCKP020058 Klebsiella pneumoniae 1 Klebsiella pneumoniae 1 Klebsiella pneumoniae 1 NA

GCF_002852855 Scaffold Klebsiella pneumoniae pneumoniae SCKP020059 Klebsiella pneumoniae 1 Klebsiella pneumoniae 1 Klebsiella pneumoniae 1 NA

GCF_002852865 Scaffold Klebsiella aerogenes SCKA020061 Klebsiella aerogenes 1 Klebsiella aerogenes Klebsiella aerogenes NA

GCF_002852895 Scaffold Klebsiella pneumoniae pneumoniae SCKP020078 Klebsiella pneumoniae 1 Klebsiella pneumoniae 1 Klebsiella pneumoniae 1 NA

GCF_002852915 Complete Klebsiella variicola WCHKV030666 Klebsiella variicola Klebsiella variicola Klebsiella variicola NA

GCF_002852935 Scaffold Klebsiella michiganensis SCKM020008 Klebsiella michiganensis 1 Klebsiella michiganensis 1 Klebsiella michiganensis 1 NA

GCF_002852945 Complete Klebsiella pneumoniae pneumoniae SCKP020046 Klebsiella pneumoniae 1 Klebsiella pneumoniae 1 Klebsiella pneumoniae 1 NA

GCF_002852975 Complete Klebsiella pneumoniae pneumoniae SCKP020079 Klebsiella pneumoniae 1 Klebsiella pneumoniae 1 Klebsiella pneumoniae 1 NA

GCF_002852995 Complete Klebsiella pneumoniae SCKP020049 Klebsiella pneumoniae 1 Klebsiella pneumoniae 1 Klebsiella pneumoniae 1 NA

GCF_002853015 Scaffold Klebsiella quasipneumoniae similipneumoniae SCKQ020050 Klebsiella quasipneumoniae 1 Klebsiella quasipneumoniae 1 Klebsiella quasipneumoniae NA

GCF_002853035 Scaffold Klebsiella variicola WCHKV030937 Klebsiella variicola Klebsiella variicola Klebsiella variicola NA

GCF_002853045 Scaffold Klebsiella quasipneumoniae quasipneumoniae SCKQ020041 Klebsiella quasipneumoniae 2 Klebsiella quasipneumoniae 2 Klebsiella quasipneumoniae NA

GCF_002853055 Scaffold Klebsiella michiganensis SCKM020040 Klebsiella michiganensis 1 Klebsiella michiganensis 1 Klebsiella michiganensis 1 NA

GCF_002853095 Scaffold Klebsiella pneumoniae ozaenae WCHKP030320 Klebsiella pneumoniae 1 Klebsiella pneumoniae 1 Klebsiella pneumoniae 1 NA

GCF_002853115 Complete Klebsiella pneumoniae ozaenae WCHKP030925 Klebsiella pneumoniae 1 Klebsiella pneumoniae 1 Klebsiella pneumoniae 1 NA

GCF_002853135 Scaffold Klebsiella pneumoniae pneumoniae WCHKP030755 Klebsiella pneumoniae 1 Klebsiella pneumoniae 1 Klebsiella pneumoniae 1 NA

GCF_002853155 Scaffold Klebsiella pneumoniae pneumoniae WCHKP030295 Klebsiella pneumoniae 1 Klebsiella pneumoniae 1 Klebsiella pneumoniae 1 NA

GCF_002853165 Scaffold Klebsiella pneumoniae ozaenae WCHKP030767 Klebsiella pneumoniae 1 Klebsiella pneumoniae 1 Klebsiella pneumoniae 1 NA

GCF_002853195 Scaffold Klebsiella oxytoca WCHKO085010 Klebsiella oxytoca 1 Klebsiella oxytoca 1 Klebsiella oxytoca 1 NA

GCF_002853215 Scaffold Klebsiella oxytoca WCHKO085006 Klebsiella oxytoca 1 Klebsiella oxytoca 1 Klebsiella oxytoca 1 NA

GCF_002853235 Scaffold Klebsiella pneumoniae pneumoniae WCHKP020100 Klebsiella pneumoniae 1 Klebsiella pneumoniae 1 Klebsiella pneumoniae 1 NA

GCF_002853255 Scaffold Klebsiella pneumoniae pneumoniae WCHKP020092 Klebsiella pneumoniae 1 Klebsiella pneumoniae 1 Klebsiella pneumoniae 1 NA

GCF_002853275 Scaffold Klebsiella variicola SCKV020148 Klebsiella variicola Klebsiella variicola Klebsiella variicola NA

GCF_002853285 Scaffold Klebsiella pneumoniae pneumoniae WCHKP020089 Klebsiella pneumoniae 1 Klebsiella pneumoniae 1 Klebsiella pneumoniae 1 NA

GCF_002853315 Scaffold Klebsiella pneumoniae pneumoniae SCKP020154 Klebsiella pneumoniae 1 Klebsiella pneumoniae 1 Klebsiella pneumoniae 1 NA

GCF_002853335 Scaffold Klebsiella pneumoniae pneumoniae SCKP020153 Klebsiella pneumoniae 1 Klebsiella pneumoniae 1 Klebsiella pneumoniae 1 NA

GCF_002853355 Scaffold Klebsiella pneumoniae ozaenae SCKP020150 Klebsiella pneumoniae 1 Klebsiella pneumoniae 1 Klebsiella pneumoniae 1 NA

GCF_002853375 Scaffold Klebsiella pneumoniae pneumoniae SCKP020152 Klebsiella pneumoniae 1 Klebsiella pneumoniae 1 Klebsiella pneumoniae 1 NA

GCF_002853385 Scaffold Klebsiella pneumoniae ozaenae SCKP020151 Klebsiella pneumoniae 1 Klebsiella pneumoniae 1 Klebsiella pneumoniae 1 NA

GCF_002853395 Scaffold Klebsiella pneumoniae pneumoniae SCKP020146 Klebsiella pneumoniae 1 Klebsiella pneumoniae 1 Klebsiella pneumoniae 1 NA

GCF_002853435 Complete Klebsiella pneumoniae pneumoniae SCKP020143 Klebsiella pneumoniae 1 Klebsiella pneumoniae 1 Klebsiella pneumoniae 1 NA

GCF_002853455 Scaffold Klebsiella pneumoniae SCKP020137 Klebsiella pneumoniae 1 Klebsiella pneumoniae 1 Klebsiella pneumoniae 1 NA

GCF_002853465 Scaffold Klebsiella pneumoniae SCKP020136 Klebsiella pneumoniae 1 Klebsiella pneumoniae 1 Klebsiella pneumoniae 1 NA

GCF_002853475 Complete Klebsiella pneumoniae SCKP020135 Klebsiella pneumoniae 1 Klebsiella pneumoniae 1 Klebsiella pneumoniae 1 NA

GCF_002853515 Scaffold Klebsiella pneumoniae pneumoniae SCKP020071 Klebsiella pneumoniae 1 Klebsiella pneumoniae 1 Klebsiella pneumoniae 1 NA

GCF_002853525 Scaffold Klebsiella pneumoniae ozaenae SCKP020134 Klebsiella pneumoniae 1 Klebsiella pneumoniae 1 Klebsiella pneumoniae 1 NA

GCF_002853535 Scaffold Klebsiella pneumoniae pneumoniae SCKP020075 Klebsiella pneumoniae 1 Klebsiella pneumoniae 1 Klebsiella pneumoniae 1 NA

GCF_002853565 Contig Klebsiella pneumoniae COL-Kpn132 Klebsiella pneumoniae 1 Klebsiella pneumoniae 1 Klebsiella pneumoniae 1 NA

GCF_002853595 Contig Klebsiella pneumoniae COL-Kpn131 Klebsiella pneumoniae 1 Klebsiella pneumoniae 1 Klebsiella pneumoniae 1 NA

GCF_002853615 Contig Klebsiella quasipneumoniae COL-Kpn130 Klebsiella quasipneumoniae 2 Klebsiella quasipneumoniae 2 Klebsiella quasipneumoniae NA

GCF_002853635 Contig Klebsiella quasipneumoniae COL-Kpn128 Klebsiella quasipneumoniae 2 Klebsiella quasipneumoniae 2 Klebsiella quasipneumoniae NA

GCF_002853675 Contig Klebsiella quasipneumoniae COL-Kpn127 Klebsiella quasipneumoniae 1 Klebsiella quasipneumoniae 1 Klebsiella quasipneumoniae NA

GCF_002853695 Contig Klebsiella quasipneumoniae COL-Kpn129 Klebsiella quasipneumoniae 2 Klebsiella quasipneumoniae 2 Klebsiella quasipneumoniae NA

GCF_002853725 Contig Klebsiella pneumoniae COL-Kpn126 Klebsiella pneumoniae 1 Klebsiella pneumoniae 1 Klebsiella pneumoniae 1 NA

GCF_002853735 Contig Klebsiella pneumoniae COL-Kpn125 Klebsiella pneumoniae 1 Klebsiella pneumoniae 1 Klebsiella pneumoniae 1 NA

GCF_002853745 Contig Klebsiella quasipneumoniae COL-Kpn124 Klebsiella quasipneumoniae 1 Klebsiella quasipneumoniae 1 Klebsiella quasipneumoniae NA

GCF_002853795 Contig Klebsiella pneumoniae COL-Kpn123 Klebsiella pneumoniae 1 Klebsiella pneumoniae 1 Klebsiella pneumoniae 1 NA

GCF_002853815 Contig Klebsiella pneumoniae COL-Kpn121 Klebsiella pneumoniae 1 Klebsiella pneumoniae 1 Klebsiella pneumoniae 1 NA

GCF_002853825 Contig Klebsiella pneumoniae COL-Kpn122 Klebsiella pneumoniae 1 Klebsiella pneumoniae 1 Klebsiella pneumoniae 1 NA

GCF_002853875 Contig Klebsiella quasipneumoniae COL-Kpn120 Klebsiella quasipneumoniae 2 Klebsiella quasipneumoniae 2 Klebsiella quasipneumoniae NA

GCF_002853895 Contig Klebsiella pneumoniae COL-Kpn117 Klebsiella pneumoniae 1 Klebsiella pneumoniae 1 Klebsiella pneumoniae 1 NA

GCF_002853915 Contig Klebsiella pneumoniae COL-Kpn114 Klebsiella pneumoniae 1 Klebsiella pneumoniae 1 Klebsiella pneumoniae 1 NA

GCF_002853935 Contig Klebsiella pneumoniae COL-Kpn112 Klebsiella pneumoniae 1 Klebsiella pneumoniae 1 Klebsiella pneumoniae 1 NA

GCF_002853955 Contig Klebsiella pneumoniae COL-Kpn111 Klebsiella pneumoniae 1 Klebsiella pneumoniae 1 Klebsiella pneumoniae 1 NA

GCF_002853975 Contig Klebsiella pneumoniae COL-Kpn118 Klebsiella pneumoniae 1 Klebsiella pneumoniae 1 Klebsiella pneumoniae 1 NA

GCF_002854015 Contig Klebsiella pneumoniae COL-Kpn110 Klebsiella pneumoniae 1 Klebsiella pneumoniae 1 Klebsiella pneumoniae 1 NA

GCF_002854035 Contig Klebsiella pneumoniae COL-Kpn107 Klebsiella pneumoniae 1 Klebsiella pneumoniae 1 Klebsiella pneumoniae 1 NA

GCF_002854055 Contig Klebsiella pneumoniae COL-Kpn109 Klebsiella pneumoniae 1 Klebsiella pneumoniae 1 Klebsiella pneumoniae 1 NA

GCF_002854085 Contig Klebsiella pneumoniae COL-Kpn108 Klebsiella pneumoniae 1 Klebsiella pneumoniae 1 Klebsiella pneumoniae 1 NA

GCF_002854115 Contig Klebsiella pneumoniae COL-Kpn105 Klebsiella pneumoniae 1 Klebsiella pneumoniae 1 Klebsiella pneumoniae 1 NA

GCF_002854135 Contig Klebsiella pneumoniae COL-Kpn106 Klebsiella pneumoniae 1 Klebsiella pneumoniae 1 Klebsiella pneumoniae 1 NA

GCF_002854145 Contig Klebsiella pneumoniae COL-Kpn104 Klebsiella pneumoniae 1 Klebsiella pneumoniae 1 Klebsiella pneumoniae 1 NA

GCF_002854175 Contig Klebsiella pneumoniae COL-Kpn100 Klebsiella pneumoniae 1 Klebsiella pneumoniae 1 Klebsiella pneumoniae 1 NA

GCF_002854195 Contig Klebsiella pneumoniae COL-Kpn95 Klebsiella pneumoniae 1 Klebsiella pneumoniae 1 Klebsiella pneumoniae 1 NA

GCF_002854215 Contig Klebsiella pneumoniae COL-Kpn94 Klebsiella pneumoniae 1 Klebsiella pneumoniae 1 Klebsiella pneumoniae 1 NA

GCF_002854235 Contig Klebsiella pneumoniae COL-Kpn89 Klebsiella pneumoniae 1 Klebsiella pneumoniae 1 Klebsiella pneumoniae 1 NA

GCF_002854255 Contig Klebsiella pneumoniae COL-Kpn93 Klebsiella pneumoniae 1 Klebsiella pneumoniae 1 Klebsiella pneumoniae 1 NA

GCF_002854275 Contig Klebsiella pneumoniae COL-Kpn86 Klebsiella pneumoniae 1 Klebsiella pneumoniae 1 Klebsiella pneumoniae 1 NA

GCF_002854285 Contig Klebsiella pneumoniae COL-Kpn85 Klebsiella pneumoniae 1 Klebsiella pneumoniae 1 Klebsiella pneumoniae 1 NA

GCF_002854295 Contig Klebsiella pneumoniae COL-Kpn83 Klebsiella pneumoniae 1 Klebsiella pneumoniae 1 Klebsiella pneumoniae 1 NA

GCF_002854335 Contig Klebsiella pneumoniae COL-Kpn82 Klebsiella pneumoniae 1 Klebsiella pneumoniae 1 Klebsiella pneumoniae 1 NA

GCF_002854355 Contig Klebsiella pneumoniae COL-Kpn80 Klebsiella pneumoniae 1 Klebsiella pneumoniae 1 Klebsiella pneumoniae 1 NA

GCF_002854375 Contig Klebsiella pneumoniae COL-Kpn77 Klebsiella pneumoniae 1 Klebsiella pneumoniae 1 Klebsiella pneumoniae 1 NA

GCF_002854385 Contig Klebsiella pneumoniae COL-Kpn75 Klebsiella pneumoniae 1 Klebsiella pneumoniae 1 Klebsiella pneumoniae 1 NA

GCF_002854415 Contig Klebsiella pneumoniae COL-Kpn74 Klebsiella pneumoniae 1 Klebsiella pneumoniae 1 Klebsiella pneumoniae 1 NA

GCF_002854435 Contig Klebsiella pneumoniae COL-Kpn71 Klebsiella pneumoniae 1 Klebsiella pneumoniae 1 Klebsiella pneumoniae 1 NA

GCF_002854455 Contig Klebsiella pneumoniae COL-Kpn69 Klebsiella pneumoniae 1 Klebsiella pneumoniae 1 Klebsiella pneumoniae 1 NA

GCF_002854465 Contig Klebsiella pneumoniae COL-Kpn70 Klebsiella pneumoniae 1 Klebsiella pneumoniae 1 Klebsiella pneumoniae 1 NA

GCF_002854495 Contig Klebsiella pneumoniae COL-Kpn65 Klebsiella pneumoniae 1 Klebsiella pneumoniae 1 Klebsiella pneumoniae 1 NA

GCF_002854515 Contig Klebsiella pneumoniae COL-Kpn64 Klebsiella pneumoniae 1 Klebsiella pneumoniae 1 Klebsiella pneumoniae 1 NA

GCF_002854525 Contig Klebsiella pneumoniae COL-Kpn63 Klebsiella pneumoniae 1 Klebsiella pneumoniae 1 Klebsiella pneumoniae 1 NA

GCF_002854535 Contig Klebsiella quasipneumoniae COL-Kpn61 Klebsiella quasipneumoniae 2 Klebsiella quasipneumoniae 2 Klebsiella quasipneumoniae NA

GCF_002854575 Contig Klebsiella pneumoniae COL-Kpn58 Klebsiella pneumoniae 1 Klebsiella pneumoniae 1 Klebsiella pneumoniae 1 NA

GCF_002854585 Contig Klebsiella pneumoniae COL-Kpn57 Klebsiella pneumoniae 1 Klebsiella pneumoniae 1 Klebsiella pneumoniae 1 NA

GCF_002854595 Contig Klebsiella quasipneumoniae COL-Kpn60 Klebsiella quasipneumoniae 2 Klebsiella quasipneumoniae 2 Klebsiella quasipneumoniae NA

GCF_002854635 Contig Klebsiella pneumoniae COL-Kpn54 Klebsiella pneumoniae 1 Klebsiella pneumoniae 1 Klebsiella pneumoniae 1 NA

GCF_002854655 Contig Klebsiella pneumoniae COL-Kpn50 Klebsiella pneumoniae 1 Klebsiella pneumoniae 1 Klebsiella pneumoniae 1 NA

GCF_002854675 Contig Klebsiella pneumoniae COL-Kpn52 Klebsiella pneumoniae 1 Klebsiella pneumoniae 1 Klebsiella pneumoniae 1 NA

GCF_002854695 Contig Klebsiella pneumoniae COL-Kpn51 Klebsiella pneumoniae 1 Klebsiella pneumoniae 1 Klebsiella pneumoniae 1 NA

GCF_002854715 Contig Klebsiella pneumoniae COL-Kpn55 Klebsiella pneumoniae 1 Klebsiella pneumoniae 1 Klebsiella pneumoniae 1 NA

GCF_002854735 Contig Klebsiella pneumoniae COL-Kpn48 Klebsiella pneumoniae 1 Klebsiella pneumoniae 1 Klebsiella pneumoniae 1 NA

GCF_002854745 Contig Klebsiella pneumoniae COL-Kpn119 Klebsiella pneumoniae 1 Klebsiella pneumoniae 1 Klebsiella pneumoniae 1 NA

GCF_002854775 Contig Klebsiella quasipneumoniae COL-Kpn133 Klebsiella quasipneumoniae 2 Klebsiella quasipneumoniae 2 Klebsiella quasipneumoniae NA

GCF_002854795 Contig Klebsiella quasipneumoniae COL-Kpn116 Klebsiella quasipneumoniae 1 Klebsiella quasipneumoniae 1 Klebsiella quasipneumoniae NA

GCF_002854815 Contig Klebsiella pneumoniae COL-Kpn113 Klebsiella pneumoniae 1 Klebsiella pneumoniae 1 Klebsiella pneumoniae 1 NA

GCF_002854835 Contig Klebsiella pneumoniae COL-Kpn102 Klebsiella pneumoniae 1 Klebsiella pneumoniae 1 Klebsiella pneumoniae 1 NA

GCF_002854855 Contig Klebsiella pneumoniae COL-Kpn115 Klebsiella pneumoniae 1 Klebsiella pneumoniae 1 Klebsiella pneumoniae 1 NA

GCF_002854865 Contig Klebsiella pneumoniae COL-Kpn103 Klebsiella pneumoniae 1 Klebsiella pneumoniae 1 Klebsiella pneumoniae 1 NA

GCF_002854895 Contig Klebsiella pneumoniae COL-Kpn101 Klebsiella pneumoniae 1 Klebsiella pneumoniae 1 Klebsiella pneumoniae 1 NA

GCF_002854915 Contig Klebsiella pneumoniae COL-Kpn99 Klebsiella pneumoniae 1 Klebsiella pneumoniae 1 Klebsiella pneumoniae 1 NA

GCF_002854935 Contig Klebsiella pneumoniae COL-Kpn98 Klebsiella pneumoniae 1 Klebsiella pneumoniae 1 Klebsiella pneumoniae 1 NA

GCF_002854955 Contig Klebsiella pneumoniae COL-Kpn97 Klebsiella pneumoniae 1 Klebsiella pneumoniae 1 Klebsiella pneumoniae 1 NA

GCF_002854975 Contig Klebsiella pneumoniae COL-Kpn96 Klebsiella pneumoniae 1 Klebsiella pneumoniae 1 Klebsiella pneumoniae 1 NA

GCF_002854995 Contig Klebsiella pneumoniae COL-Kpn92 Klebsiella pneumoniae 1 Klebsiella pneumoniae 1 Klebsiella pneumoniae 1 NA

GCF_002855015 Contig Klebsiella pneumoniae COL-Kpn90 Klebsiella pneumoniae 1 Klebsiella pneumoniae 1 Klebsiella pneumoniae 1 NA

GCF_002855035 Contig Klebsiella pneumoniae COL-Kpn91 Klebsiella pneumoniae 1 Klebsiella pneumoniae 1 Klebsiella pneumoniae 1 NA

GCF_002855045 Contig Klebsiella pneumoniae COL-Kpn88 Klebsiella pneumoniae 1 Klebsiella pneumoniae 1 Klebsiella pneumoniae 1 NA

GCF_002855075 Contig Klebsiella pneumoniae COL-Kpn84 Klebsiella pneumoniae 1 Klebsiella pneumoniae 1 Klebsiella pneumoniae 1 NA

GCF_002855095 Contig Klebsiella quasipneumoniae COL-Kpn81 Klebsiella quasipneumoniae 2 Klebsiella quasipneumoniae 2 Klebsiella quasipneumoniae NA

GCF_002855115 Contig Klebsiella pneumoniae COL-Kpn79 Klebsiella pneumoniae 1 Klebsiella pneumoniae 1 Klebsiella pneumoniae 1 NA

GCF_002855125 Contig Klebsiella pneumoniae COL-Kpn78 Klebsiella pneumoniae 1 Klebsiella pneumoniae 1 Klebsiella pneumoniae 1 NA

GCF_002855155 Contig Klebsiella pneumoniae COL-Kpn76 Klebsiella pneumoniae 1 Klebsiella pneumoniae 1 Klebsiella pneumoniae 1 NA

GCF_002855175 Contig Klebsiella pneumoniae COL-Kpn73 Klebsiella pneumoniae 1 Klebsiella pneumoniae 1 Klebsiella pneumoniae 1 NA

GCF_002855195 Contig Klebsiella pneumoniae COL-Kpn72 Klebsiella pneumoniae 1 Klebsiella pneumoniae 1 Klebsiella pneumoniae 1 NA

GCF_002855215 Contig Klebsiella pneumoniae COL-Kpn68 Klebsiella pneumoniae 1 Klebsiella pneumoniae 1 Klebsiella pneumoniae 1 NA

GCF_002855225 Contig Klebsiella quasipneumoniae COL-Kpn67 Klebsiella quasipneumoniae 2 Klebsiella quasipneumoniae 2 Klebsiella quasipneumoniae NA

GCF_002855255 Contig Klebsiella pneumoniae COL-Kpn62 Klebsiella pneumoniae 1 Klebsiella pneumoniae 1 Klebsiella pneumoniae 1 NA

GCF_002855275 Contig Klebsiella pneumoniae COL-Kpn66 Klebsiella pneumoniae 1 Klebsiella pneumoniae 1 Klebsiella pneumoniae 1 NA

GCF_002855295 Contig Klebsiella pneumoniae COL-Kpn59 Klebsiella pneumoniae 1 Klebsiella pneumoniae 1 Klebsiella pneumoniae 1 NA

GCF_002855315 Contig Klebsiella pneumoniae COL-Kpn56 Klebsiella pneumoniae 1 Klebsiella pneumoniae 1 Klebsiella pneumoniae 1 NA

GCF_002855355 Contig Klebsiella quasipneumoniae COL-Kpn49 Klebsiella quasipneumoniae 2 Klebsiella quasipneumoniae 2 Klebsiella quasipneumoniae NA

GCF_002855375 Contig Klebsiella pneumoniae COL-Kpn46 Klebsiella pneumoniae 1 Klebsiella pneumoniae 1 Klebsiella pneumoniae 1 NA

GCF_002855395 Contig Klebsiella pneumoniae COL-Kpn53 Klebsiella pneumoniae 1 Klebsiella pneumoniae 1 Klebsiella pneumoniae 1 NA

GCF_002855425 Contig Klebsiella quasipneumoniae COL-Kpn44 Klebsiella quasipneumoniae 1 Klebsiella quasipneumoniae 1 Klebsiella quasipneumoniae NA

GCF_002855465 Scaffold Klebsiella variicola BDDM165 Klebsiella variicola Klebsiella variicola Klebsiella variicola NA

GCF_002855815 Scaffold Klebsiella pneumoniae D68 Klebsiella pneumoniae 1 Klebsiella pneumoniae 1 Klebsiella pneumoniae 1 NA

GCF_002855825 Scaffold Klebsiella pneumoniae D62 Klebsiella pneumoniae 1 Klebsiella pneumoniae 1 Klebsiella pneumoniae 1 NA

GCF_002855865 Scaffold Klebsiella quasipneumoniae D52 Klebsiella quasipneumoniae 2 Klebsiella quasipneumoniae 2 Klebsiella quasipneumoniae NA

GCF_002855915 Scaffold Klebsiella pneumoniae B14 Klebsiella pneumoniae 1 Klebsiella pneumoniae 1 Klebsiella pneumoniae 1 NA

GCF_002855955 Contig Klebsiella pneumoniae D27 Klebsiella pneumoniae 1 Klebsiella pneumoniae 1 Klebsiella pneumoniae 1 NA

GCF_002855985 Contig Klebsiella pneumoniae C35 Klebsiella pneumoniae 1 Klebsiella pneumoniae 1 Klebsiella pneumoniae 1 NA

GCF_002856015 Contig Klebsiella pneumoniae C29 Klebsiella pneumoniae 1 Klebsiella pneumoniae 1 Klebsiella pneumoniae 1 NA

GCF_002856025 Contig Klebsiella pneumoniae D20 Klebsiella pneumoniae 1 Klebsiella pneumoniae 1 Klebsiella pneumoniae 1 NA

GCF_002856075 Scaffold Klebsiella pneumoniae C23 Klebsiella pneumoniae 1 Klebsiella pneumoniae 1 Klebsiella pneumoniae 1 NA

GCF_002856185 Contig Klebsiella pneumoniae D15 Klebsiella pneumoniae 1 Klebsiella pneumoniae 1 Klebsiella pneumoniae 1 NA

GCF_002856195 Scaffold Klebsiella michiganensis D14 Klebsiella grimontii Klebsiella grimontii Klebsiella grimontii NA

GCF_002856275 Contig Klebsiella quasipneumoniae D65 Klebsiella quasipneumoniae 2 Klebsiella quasipneumoniae 2 Klebsiella quasipneumoniae NA

GCF_002856315 Scaffold Klebsiella pneumoniae C42 Klebsiella pneumoniae 1 Klebsiella pneumoniae 1 Klebsiella pneumoniae 1 NA

GCF_002856325 Contig Klebsiella pneumoniae C41 Klebsiella pneumoniae 1 Klebsiella pneumoniae 1 Klebsiella pneumoniae 1 NA

GCF_002856365 Contig Klebsiella quasipneumoniae C37 Klebsiella quasipneumoniae 2 Klebsiella quasipneumoniae 2 Klebsiella quasipneumoniae NA

GCF_002856385 Scaffold Klebsiella pneumoniae D44 Klebsiella pneumoniae 1 Klebsiella pneumoniae 1 Klebsiella pneumoniae 1 NA

GCF_002856395 Scaffold Klebsiella pneumoniae C36 Klebsiella pneumoniae 1 Klebsiella pneumoniae 1 Klebsiella pneumoniae 1 NA

GCF_002856415 Contig Klebsiella pneumoniae D39 Klebsiella pneumoniae 1 Klebsiella pneumoniae 1 Klebsiella pneumoniae 1 NA

GCF_002856455 Scaffold Klebsiella pneumoniae D36 Klebsiella pneumoniae 1 Klebsiella pneumoniae 1 Klebsiella pneumoniae 1 NA

GCF_002856485 Scaffold Klebsiella quasipneumoniae D32 Klebsiella quasipneumoniae 2 Klebsiella quasipneumoniae 2 Klebsiella quasipneumoniae NA

GCF_002856505 Scaffold Klebsiella quasipneumoniae D30 Klebsiella quasipneumoniae 2 Klebsiella quasipneumoniae 2 Klebsiella quasipneumoniae NA

GCF_002856535 Scaffold Klebsiella pneumoniae C32 Klebsiella pneumoniae 1 Klebsiella pneumoniae 1 Klebsiella pneumoniae 1 NA

GCF_002856565 Scaffold Klebsiella pneumoniae C27 Klebsiella pneumoniae 1 Klebsiella pneumoniae 1 Klebsiella pneumoniae 1 NA

GCF_002856575 Scaffold Klebsiella pneumoniae C26 Klebsiella pneumoniae 1 Klebsiella pneumoniae 1 Klebsiella pneumoniae 1 NA

GCF_002856615 Scaffold Klebsiella pneumoniae C14 Klebsiella pneumoniae 1 Klebsiella pneumoniae 1 Klebsiella pneumoniae 1 NA

GCF_002856665 Contig Klebsiella quasipneumoniae D16 Klebsiella quasipneumoniae 2 Klebsiella quasipneumoniae 2 Klebsiella quasipneumoniae NA

GCF_002856675 Contig Klebsiella pneumoniae C24 Klebsiella pneumoniae 1 Klebsiella pneumoniae 1 Klebsiella pneumoniae 1 NA

GCF_002856765 Contig Klebsiella pneumoniae A3 Klebsiella pneumoniae 1 Klebsiella pneumoniae 1 Klebsiella pneumoniae 1 NA

GCF_002856885 Scaffold Klebsiella pneumoniae C43 Klebsiella pneumoniae 1 Klebsiella pneumoniae 1 Klebsiella pneumoniae 1 NA

GCF_002856915 Scaffold Klebsiella pneumoniae B7 Klebsiella pneumoniae 1 Klebsiella pneumoniae 1 Klebsiella pneumoniae 1 NA

GCF_002856945 Scaffold Klebsiella pneumoniae D42 Klebsiella pneumoniae 1 Klebsiella pneumoniae 1 Klebsiella pneumoniae 1 NA

GCF_002856965 Scaffold Klebsiella michiganensis D40 Klebsiella michiganensis 1 Klebsiella michiganensis 1 Klebsiella michiganensis 1 NA

GCF_002856995 Scaffold Klebsiella pneumoniae A9 Klebsiella pneumoniae 1 Klebsiella pneumoniae 1 Klebsiella pneumoniae 1 NA

GCF_002857005 Scaffold Klebsiella pneumoniae D38 Klebsiella pneumoniae 1 Klebsiella pneumoniae 1 Klebsiella pneumoniae 1 NA

GCF_002857035 Contig Klebsiella pneumoniae D37 Klebsiella pneumoniae 1 Klebsiella pneumoniae 1 Klebsiella pneumoniae 1 NA

GCF_002857065 Scaffold Klebsiella pneumoniae D34 Klebsiella pneumoniae 1 Klebsiella pneumoniae 1 Klebsiella pneumoniae 1 NA

GCF_002857085 Scaffold Klebsiella pneumoniae D26 Klebsiella pneumoniae 1 Klebsiella pneumoniae 1 Klebsiella pneumoniae 1 NA

GCF_002857115 Scaffold Klebsiella pneumoniae C30 Klebsiella pneumoniae 1 Klebsiella pneumoniae 1 Klebsiella pneumoniae 1 NA

GCF_002857125 Scaffold Klebsiella pneumoniae D25 Klebsiella pneumoniae 1 Klebsiella pneumoniae 1 Klebsiella pneumoniae 1 NA

GCF_002857205 Scaffold Klebsiella pneumoniae C21 Klebsiella pneumoniae 1 Klebsiella pneumoniae 1 Klebsiella pneumoniae 1 NA

GCF_002857235 Scaffold Klebsiella pneumoniae C13 Klebsiella pneumoniae 1 Klebsiella pneumoniae 1 Klebsiella pneumoniae 1 NA

GCF_002857265 Contig Klebsiella quasipneumoniae D13 Klebsiella quasipneumoniae 2 Klebsiella quasipneumoniae 2 Klebsiella quasipneumoniae NA

GCF_002857315 Scaffold Klebsiella pneumoniae C6 Klebsiella pneumoniae 1 Klebsiella pneumoniae 1 Klebsiella pneumoniae 1 NA

GCF_002857345 Scaffold Klebsiella pneumoniae D4 Klebsiella pneumoniae 1 Klebsiella pneumoniae 1 Klebsiella pneumoniae 1 NA

GCF_002861655 Scaffold Klebsiella pneumoniae SH1 Klebsiella pneumoniae 1 Klebsiella pneumoniae 1 Klebsiella pneumoniae 1 NA

GCF_002861705 Scaffold Klebsiella pneumoniae HN26 Klebsiella pneumoniae 1 Klebsiella pneumoniae 1 Klebsiella pneumoniae 1 NA

GCF_002861725 Scaffold Klebsiella pneumoniae AH12 Klebsiella pneumoniae 1 Klebsiella pneumoniae 1 Klebsiella pneumoniae 1 NA

GCF_002861745 Scaffold Klebsiella pneumoniae AH11 Klebsiella pneumoniae 1 Klebsiella pneumoniae 1 Klebsiella pneumoniae 1 NA

GCF_002861845 Scaffold Klebsiella pneumoniae UMB0140 Klebsiella pneumoniae 1 Klebsiella pneumoniae 1 Klebsiella pneumoniae 1 NA

GCF_002863955 Contig Klebsiella aerogenes YDC581 Klebsiella aerogenes 1 Klebsiella aerogenes Klebsiella aerogenes NA

GCF_002863975 Contig Klebsiella aerogenes YDC581-4 Klebsiella aerogenes 1 Klebsiella aerogenes Klebsiella aerogenes NA

GCF_002866505 Contig Klebsiella pneumoniae AUH-KIMP152 Klebsiella pneumoniae 1 Klebsiella pneumoniae 1 Klebsiella pneumoniae 1 NA

GCF_002866545 Contig Klebsiella pneumoniae AUH-KIMP181 Klebsiella pneumoniae 1 Klebsiella pneumoniae 1 Klebsiella pneumoniae 1 NA

GCF_002866565 Contig Klebsiella pneumoniae AUH-KIMP180 Klebsiella pneumoniae 1 Klebsiella pneumoniae 1 Klebsiella pneumoniae 1 NA

GCF_002866585 Contig Klebsiella pneumoniae AUH-KIMP170 Klebsiella pneumoniae 1 Klebsiella pneumoniae 1 Klebsiella pneumoniae 1 NA

GCF_002866605 Contig Klebsiella pneumoniae AUH-KIMP148 Klebsiella pneumoniae 1 Klebsiella pneumoniae 1 Klebsiella pneumoniae 1 NA

GCF_002866615 Contig Klebsiella pneumoniae AUH-KIMP182 Klebsiella pneumoniae 1 Klebsiella pneumoniae 1 Klebsiella pneumoniae 1 NA

GCF_002866645 Contig Klebsiella pneumoniae AUH-KIMP158 Klebsiella pneumoniae 1 Klebsiella pneumoniae 1 Klebsiella pneumoniae 1 NA

GCF_002866665 Contig Klebsiella pneumoniae AUH-KIMP159 Klebsiella pneumoniae 1 Klebsiella pneumoniae 1 Klebsiella pneumoniae 1 NA

GCF_002866675 Contig Klebsiella pneumoniae AUH-KIMP177 Klebsiella pneumoniae 1 Klebsiella pneumoniae 1 Klebsiella pneumoniae 1 NA

GCF_002870865 Chromosome Klebsiella pneumoniae LS356 Klebsiella pneumoniae 1 Klebsiella pneumoniae 1 Klebsiella pneumoniae 1 NA

GCF_002870885 Chromosome Klebsiella pneumoniae LS357 Klebsiella pneumoniae 1 Klebsiella pneumoniae 1 Klebsiella pneumoniae 1 NA

GCF_002870905 Chromosome Klebsiella pneumoniae HS09565 Klebsiella pneumoniae 1 Klebsiella pneumoniae 1 Klebsiella pneumoniae 1 NA

GCF_002870925 Chromosome Klebsiella pneumoniae HS102438 Klebsiella pneumoniae 1 Klebsiella pneumoniae 1 Klebsiella pneumoniae 1 NA

GCF_002870945 Chromosome Klebsiella pneumoniae LS355 Klebsiella pneumoniae 1 Klebsiella pneumoniae 1 Klebsiella pneumoniae 1 NA

GCF_002870985 Chromosome Klebsiella pneumoniae LS358 Klebsiella pneumoniae 1 Klebsiella pneumoniae 1 Klebsiella pneumoniae 1 NA

GCF_002871025 Chromosome Klebsiella pneumoniae LS359 Klebsiella pneumoniae 1 Klebsiella pneumoniae 1 Klebsiella pneumoniae 1 NA

GCF_002872205 Contig Klebsiella pneumoniae KPF39 Klebsiella pneumoniae 1 Klebsiella pneumoniae 1 Klebsiella pneumoniae 1 NA

GCF_002880715 Contig Klebsiella sp Kd70 TUC-EEAOC Klebsiella grimontii Klebsiella grimontii Klebsiella grimontii NA

GCF_002886365 Contig Klebsiella pneumoniae YD762-3 Klebsiella pneumoniae 1 Klebsiella pneumoniae 1 Klebsiella pneumoniae 1 NA

GCF_002886375 Contig Klebsiella pneumoniae YD762-2 Klebsiella pneumoniae 1 Klebsiella pneumoniae 1 Klebsiella pneumoniae 1 NA

GCF_002886595 Contig Klebsiella pneumoniae YDC465 Klebsiella pneumoniae 1 Klebsiella pneumoniae 1 Klebsiella pneumoniae 1 NA

GCF_002886665 Contig Klebsiella variicola YD626-2 Klebsiella variicola Klebsiella variicola Klebsiella variicola NA

GCF_002886705 Contig Klebsiella pneumoniae YD648-2 Klebsiella pneumoniae 1 Klebsiella pneumoniae 1 Klebsiella pneumoniae 1 NA

GCF_002886965 Contig Klebsiella pneumoniae YDC121 Klebsiella pneumoniae 1 Klebsiella pneumoniae 1 Klebsiella pneumoniae 1 NA

GCF_002887165 Contig Klebsiella michiganensis YD358 Klebsiella michiganensis 1 Klebsiella michiganensis 1 Klebsiella michiganensis 1 NA

GCF_002887435 Contig Klebsiella pneumoniae Kp81 Klebsiella pneumoniae 1 Klebsiella pneumoniae 1 Klebsiella pneumoniae 1 NA

GCF_002887605 Contig Klebsiella michiganensis YDC736-2 Klebsiella michiganensis 1 Klebsiella michiganensis 1 Klebsiella michiganensis 1 NA

GCF_002887745 Scaffold Klebsiella pneumoniae K7RB Klebsiella pneumoniae 1 Klebsiella pneumoniae 1 Klebsiella pneumoniae 1 NA

GCF_002891215 Contig Klebsiella aerogenes FDAARGOS231 Klebsiella aerogenes 1 Klebsiella aerogenes Klebsiella aerogenes NA

GCF_002893825 Complete Klebsiella pneumoniae pneumoniae GD4 Klebsiella pneumoniae 1 Klebsiella pneumoniae 1 Klebsiella pneumoniae 1 NA

GCF_002894385 Contig Klebsiella pneumoniae pneumoniae WCHKP020039 Klebsiella pneumoniae 1 Klebsiella pneumoniae 1 Klebsiella pneumoniae 1 NA

GCF_002895645 Contig Klebsiella pneumoniae 182604R Klebsiella pneumoniae 1 Klebsiella pneumoniae 1 Klebsiella pneumoniae 1 NA

GCF_002897415 Contig Klebsiella pneumoniae MH17-016D Klebsiella pneumoniae 1 Klebsiella pneumoniae 1 Klebsiella pneumoniae 1 NA

GCF_002900015 Scaffold Klebsiella pneumoniae M057110 Klebsiella pneumoniae 1 Klebsiella pneumoniae 1 Klebsiella pneumoniae 1 NA

GCF_002900035 Scaffold Klebsiella pneumoniae M053972 Klebsiella pneumoniae 1 Klebsiella pneumoniae 1 Klebsiella pneumoniae 1 NA

GCF_002900135 Contig Klebsiella pneumoniae M091528 Klebsiella pneumoniae 1 Klebsiella pneumoniae 1 Klebsiella pneumoniae 1 NA

GCF_002900145 Scaffold Klebsiella pneumoniae M097275 Klebsiella pneumoniae 1 Klebsiella pneumoniae 1 Klebsiella pneumoniae 1 NA

GCF_002901335 Scaffold Klebsiella pneumoniae RUC232 Klebsiella pneumoniae 1 Klebsiella pneumoniae 1 Klebsiella pneumoniae 1 NA

GCF_002903005 Complete Klebsiella pneumoniae KPNIH50 Klebsiella pneumoniae 1 Klebsiella pneumoniae 1 Klebsiella pneumoniae 1 NA

GCF_002903025 Complete Klebsiella pneumoniae KPNIH49 Klebsiella pneumoniae 1 Klebsiella pneumoniae 1 Klebsiella pneumoniae 1 NA

GCF_002903895 Contig Klebsiella pneumoniae LBMM1463 Klebsiella pneumoniae 1 Klebsiella pneumoniae 1 Klebsiella pneumoniae 1 NA

GCF_002903925 Contig Klebsiella pneumoniae LBMM1302 Klebsiella pneumoniae 1 Klebsiella pneumoniae 1 Klebsiella pneumoniae 1 NA

GCF_002903945 Contig Klebsiella pneumoniae LBMM1301 Klebsiella pneumoniae 1 Klebsiella pneumoniae 1 Klebsiella pneumoniae 1 NA

GCF_002903965 Contig Klebsiella pneumoniae LBMM1303 Klebsiella pneumoniae 1 Klebsiella pneumoniae 1 Klebsiella pneumoniae 1 NA

GCF_002903985 Contig Klebsiella pneumoniae LBMM1300 Klebsiella pneumoniae 1 Klebsiella pneumoniae 1 Klebsiella pneumoniae 1 NA

GCF_002906395 Complete Klebsiella oxytoca KONIH4 Klebsiella michiganensis 1 Klebsiella michiganensis 1 Klebsiella michiganensis 1 NA

GCF_002906415 Complete Klebsiella oxytoca KONIH2 Klebsiella michiganensis 1 Klebsiella michiganensis 1 Klebsiella michiganensis 1 NA

GCF_002906435 Complete Klebsiella oxytoca KONIH5 Klebsiella michiganensis 1 Klebsiella michiganensis 1 Klebsiella michiganensis 1 NA

GCF_002909775 Complete Klebsiella pneumoniae BA28434 Klebsiella pneumoniae 1 Klebsiella pneumoniae 1 Klebsiella pneumoniae 1 NA

GCF_002909805 Scaffold Klebsiella pneumoniae BA29256 Klebsiella pneumoniae 1 Klebsiella pneumoniae 1 Klebsiella pneumoniae 1 NA

GCF_002909895 Contig Klebsiella pneumoniae OctC4 3 2 Klebsiella pneumoniae 1 Klebsiella pneumoniae 1 Klebsiella pneumoniae 1 NA

GCF_002909905 Contig Klebsiella pneumoniae OctC4 3 Klebsiella pneumoniae 1 Klebsiella pneumoniae 1 Klebsiella pneumoniae 1 NA

GCF_002909975 Contig Klebsiella pneumoniae OctC4 2 Klebsiella pneumoniae 1 Klebsiella pneumoniae 1 Klebsiella pneumoniae 1 NA

GCF_002909995 Contig Klebsiella pneumoniae OctC4 2 3 Klebsiella pneumoniae 1 Klebsiella pneumoniae 1 Klebsiella pneumoniae 1 NA

GCF_002910015 Contig Klebsiella pneumoniae OctC4 1 3 Klebsiella pneumoniae 1 Klebsiella pneumoniae 1 Klebsiella pneumoniae 1 NA

GCF_002910035 Contig Klebsiella pneumoniae OctC4 1 Klebsiella pneumoniae 1 Klebsiella pneumoniae 1 Klebsiella pneumoniae 1 NA

GCF_002910055 Contig Klebsiella pneumoniae PMB4 Klebsiella pneumoniae 1 Klebsiella pneumoniae 1 Klebsiella pneumoniae 1 NA

GCF_002910075 Contig Klebsiella pneumoniae PMB4 2 Klebsiella pneumoniae 1 Klebsiella pneumoniae 1 Klebsiella pneumoniae 1 NA

GCF_002910085 Contig Klebsiella pneumoniae PMB4 3 Klebsiella pneumoniae 1 Klebsiella pneumoniae 1 Klebsiella pneumoniae 1 NA

GCF_002910115 Contig Klebsiella pneumoniae PMB4 1 Klebsiella pneumoniae 1 Klebsiella pneumoniae 1 Klebsiella pneumoniae 1 NA

GCF_002910125 Contig Klebsiella pneumoniae PMB3 4 Klebsiella pneumoniae 1 Klebsiella pneumoniae 1 Klebsiella pneumoniae 1 NA

GCF_002910155 Contig Klebsiella pneumoniae PMB3 Klebsiella pneumoniae 1 Klebsiella pneumoniae 1 Klebsiella pneumoniae 1 NA

GCF_002910165 Contig Klebsiella pneumoniae PMB3 1 Klebsiella pneumoniae 1 Klebsiella pneumoniae 1 Klebsiella pneumoniae 1 NA

GCF_002910195 Contig Klebsiella pneumoniae PMB2 1 Klebsiella pneumoniae 1 Klebsiella pneumoniae 1 Klebsiella pneumoniae 1 NA

GCF_002910205 Contig Klebsiella pneumoniae PMB1 4 Klebsiella pneumoniae 1 Klebsiella pneumoniae 1 Klebsiella pneumoniae 1 NA

GCF_002910235 Contig Klebsiella pneumoniae PMB2 Klebsiella pneumoniae 1 Klebsiella pneumoniae 1 Klebsiella pneumoniae 1 NA

GCF_002910255 Contig Klebsiella pneumoniae PMB1 3 Klebsiella pneumoniae 1 Klebsiella pneumoniae 1 Klebsiella pneumoniae 1 NA

GCF_002910335 Contig Klebsiella pneumoniae OctC4 Klebsiella pneumoniae 1 Klebsiella pneumoniae 1 Klebsiella pneumoniae 1 NA

GCF_002910375 Contig Klebsiella pneumoniae OctC4 3 1 Klebsiella pneumoniae 1 Klebsiella pneumoniae 1 Klebsiella pneumoniae 1 NA

GCF_002910435 Contig Klebsiella pneumoniae OctC4 2 1 Klebsiella pneumoniae 1 Klebsiella pneumoniae 1 Klebsiella pneumoniae 1 NA

GCF_002910455 Contig Klebsiella pneumoniae OctC4 1 2 Klebsiella pneumoniae 1 Klebsiella pneumoniae 1 Klebsiella pneumoniae 1 NA

GCF_002910495 Contig Klebsiella pneumoniae CST4 2 Klebsiella pneumoniae 1 Klebsiella pneumoniae 1 Klebsiella pneumoniae 1 NA

GCF_002910515 Contig Klebsiella pneumoniae CST4 3 Klebsiella pneumoniae 1 Klebsiella pneumoniae 1 Klebsiella pneumoniae 1 NA

GCF_002910535 Contig Klebsiella pneumoniae CST4 1 Klebsiella pneumoniae 1 Klebsiella pneumoniae 1 Klebsiella pneumoniae 1 NA

GCF_002910555 Contig Klebsiella pneumoniae CST4 Klebsiella pneumoniae 1 Klebsiella pneumoniae 1 Klebsiella pneumoniae 1 NA

GCF_002910565 Contig Klebsiella pneumoniae CST3 4 Klebsiella pneumoniae 1 Klebsiella pneumoniae 1 Klebsiella pneumoniae 1 NA

GCF_002910595 Contig Klebsiella pneumoniae CST3 1 Klebsiella pneumoniae 1 Klebsiella pneumoniae 1 Klebsiella pneumoniae 1 NA

GCF_002910615 Contig Klebsiella pneumoniae CST3 Klebsiella pneumoniae 1 Klebsiella pneumoniae 1 Klebsiella pneumoniae 1 NA

GCF_002910635 Contig Klebsiella pneumoniae CST2 3 Klebsiella pneumoniae 1 Klebsiella pneumoniae 1 Klebsiella pneumoniae 1 NA

GCF_002910655 Contig Klebsiella pneumoniae CST2 1 Klebsiella pneumoniae 1 Klebsiella pneumoniae 1 Klebsiella pneumoniae 1 NA

GCF_002910675 Contig Klebsiella pneumoniae CST1 4 Klebsiella pneumoniae 1 Klebsiella pneumoniae 1 Klebsiella pneumoniae 1 NA

GCF_002910695 Contig Klebsiella pneumoniae CST1 2 Klebsiella pneumoniae 1 Klebsiella pneumoniae 1 Klebsiella pneumoniae 1 NA

GCF_002911035 Contig Klebsiella pneumoniae PMB3 2 Klebsiella pneumoniae 1 Klebsiella pneumoniae 1 Klebsiella pneumoniae 1 NA

GCF_002911055 Contig Klebsiella pneumoniae PMB2 4 Klebsiella pneumoniae 1 Klebsiella pneumoniae 1 Klebsiella pneumoniae 1 NA

GCF_002911065 Contig Klebsiella pneumoniae PMB1 Klebsiella pneumoniae 1 Klebsiella pneumoniae 1 Klebsiella pneumoniae 1 NA

GCF_002911095 Contig Klebsiella pneumoniae PMB2 3 Klebsiella pneumoniae 1 Klebsiella pneumoniae 1 Klebsiella pneumoniae 1 NA

GCF_002911115 Contig Klebsiella pneumoniae CST3 2 Klebsiella pneumoniae 1 Klebsiella pneumoniae 1 Klebsiella pneumoniae 1 NA

GCF_002911135 Contig Klebsiella pneumoniae CST2 4 Klebsiella pneumoniae 1 Klebsiella pneumoniae 1 Klebsiella pneumoniae 1 NA

GCF_002911155 Contig Klebsiella pneumoniae CST1 3 Klebsiella pneumoniae 1 Klebsiella pneumoniae 1 Klebsiella pneumoniae 1 NA

GCF_002911165 Contig Klebsiella pneumoniae CST2 Klebsiella pneumoniae 1 Klebsiella pneumoniae 1 Klebsiella pneumoniae 1 NA

GCF_002918585 Complete Klebsiella pneumoniae KPNIH45 Klebsiella pneumoniae 1 Klebsiella pneumoniae 1 Klebsiella pneumoniae 1 NA

GCF_002918615 Contig Klebsiella pneumoniae KPNIH43 Klebsiella pneumoniae 1 Klebsiella pneumoniae 1 Klebsiella pneumoniae 1 NA

GCF_002918635 Contig Klebsiella oxytoca KONIH8 Klebsiella michiganensis 1 Klebsiella michiganensis 1 Klebsiella michiganensis 1 NA

GCF_002918655 Contig Klebsiella oxytoca KONIH3 Klebsiella michiganensis 1 Klebsiella michiganensis 1 Klebsiella michiganensis 1 NA

GCF_002918665 Contig Klebsiella michiganensis KONIH10 Klebsiella michiganensis 1 Klebsiella michiganensis 1 Klebsiella michiganensis 1 NA

GCF_002918695 Contig Klebsiella oxytoca KONIH6 Klebsiella michiganensis 1 Klebsiella michiganensis 1 Klebsiella michiganensis 1 NA

GCF_002918815 Contig Klebsiella aerogenes EANIH1 Klebsiella aerogenes 1 Klebsiella aerogenes Klebsiella aerogenes NA

GCF_002919565 Contig Klebsiella pneumoniae KPNIH46 Klebsiella pneumoniae 1 Klebsiella pneumoniae 1 Klebsiella pneumoniae 1 NA

GCF_002919575 Contig Klebsiella pneumoniae KPNIH47 Klebsiella pneumoniae 1 Klebsiella pneumoniae 1 Klebsiella pneumoniae 1 NA

GCF_002919605 Contig Klebsiella oxytoca KONIH7 Klebsiella michiganensis 1 Klebsiella michiganensis 1 Klebsiella michiganensis 1 NA

GCF_002919625 Contig Klebsiella oxytoca KONIH9 Klebsiella michiganensis 1 Klebsiella michiganensis 1 Klebsiella michiganensis 1 NA

GCF_002920135 Contig Klebsiella pneumoniae KPNIH26 Klebsiella pneumoniae 1 Klebsiella pneumoniae 1 Klebsiella pneumoniae 1 NA

GCF_002920285 Contig Klebsiella pneumoniae KPNIH42 Klebsiella pneumoniae 1 Klebsiella pneumoniae 1 Klebsiella pneumoniae 1 NA

GCF_002920305 Contig Klebsiella pneumoniae KPNIH51 Klebsiella pneumoniae 1 Klebsiella pneumoniae 1 Klebsiella pneumoniae 1 NA

GCF_002925665 Contig Klebsiella pneumoniae CST1 Klebsiella pneumoniae 1 Klebsiella pneumoniae 1 Klebsiella pneumoniae 1 NA

GCF_002925715 Scaffold Klebsiella pneumoniae Kp3380 Klebsiella pneumoniae 1 Klebsiella pneumoniae 1 Klebsiella pneumoniae 1 NA

GCF_002925745 Scaffold Klebsiella pneumoniae Kp1803 Klebsiella pneumoniae 1 Klebsiella pneumoniae 1 Klebsiella pneumoniae 1 NA

GCF_002925905 Scaffold Klebsiella michiganensis DSM25444 Klebsiella michiganensis 1 Klebsiella michiganensis 1 Klebsiella michiganensis 1 Type strain

GCF_002925975 Contig Klebsiella pneumoniae PMB1 2 Klebsiella pneumoniae 1 Klebsiella pneumoniae 1 Klebsiella pneumoniae 1 NA

GCF_002928635 Scaffold Klebsiella pneumoniae IMT38405 Klebsiella pneumoniae 1 Klebsiella pneumoniae 1 Klebsiella pneumoniae 1 NA

GCF_002928675 Scaffold Klebsiella pneumoniae IMT38444 Klebsiella pneumoniae 1 Klebsiella pneumoniae 1 Klebsiella pneumoniae 1 NA

GCF_002928695 Scaffold Klebsiella pneumoniae IMT38403 Klebsiella pneumoniae 1 Klebsiella pneumoniae 1 Klebsiella pneumoniae 1 NA

GCF_002929175 Contig Klebsiella pneumoniae 14CSI Klebsiella pneumoniae 1 Klebsiella pneumoniae 1 Klebsiella pneumoniae 1 NA

GCF_002934725 Contig Klebsiella pneumoniae SKLX002887 Klebsiella pneumoniae 1 Klebsiella pneumoniae 1 Klebsiella pneumoniae 1 NA

GCF_002934745 Contig Klebsiella pneumoniae SKLX003155 Klebsiella pneumoniae 1 Klebsiella pneumoniae 1 Klebsiella pneumoniae 1 NA

GCF_002934765 Contig Klebsiella pneumoniae SKLX018253 Klebsiella pneumoniae 1 Klebsiella pneumoniae 1 Klebsiella pneumoniae 1 NA

GCF_002934785 Contig Klebsiella pneumoniae HN523E1II Klebsiella pneumoniae 1 Klebsiella pneumoniae 1 Klebsiella pneumoniae 1 NA

GCF_002934815 Scaffold Klebsiella pneumoniae HH510E2I Klebsiella pneumoniae 1 Klebsiella pneumoniae 1 Klebsiella pneumoniae 1 NA

GCF_002934865 Scaffold Klebsiella pneumoniae PR042E3 Klebsiella pneumoniae 1 Klebsiella pneumoniae 1 Klebsiella pneumoniae 1 NA

GCF_002934985 Scaffold Klebsiella pneumoniae PN089E1 Klebsiella pneumoniae 1 Klebsiella pneumoniae 1 Klebsiella pneumoniae 1 NA

GCF_002935005 Contig Klebsiella pneumoniae PN085E1IA Klebsiella pneumoniae 1 Klebsiella pneumoniae 1 Klebsiella pneumoniae 1 NA

GCF_002935085 Complete Klebsiella pneumoniae KPNIH48 Klebsiella pneumoniae 1 Klebsiella pneumoniae 1 Klebsiella pneumoniae 1 NA

GCF_002935125 Contig Klebsiella pneumoniae pneumoniae CRK0349 Klebsiella pneumoniae 1 Klebsiella pneumoniae 1 Klebsiella pneumoniae 1 NA

GCF_002935135 Contig Klebsiella pneumoniae CRK0390 Klebsiella pneumoniae 1 Klebsiella pneumoniae 1 Klebsiella pneumoniae 1 NA

GCF_002935165 Contig Klebsiella pneumoniae pneumoniae CRK0346 Klebsiella pneumoniae 1 Klebsiella pneumoniae 1 Klebsiella pneumoniae 1 NA

GCF_002935175 Contig Klebsiella pneumoniae pneumoniae M1A Klebsiella pneumoniae 1 Klebsiella pneumoniae 1 Klebsiella pneumoniae 1 NA

GCF_002935205 Contig Klebsiella pneumoniae pneumoniae CRK0350 Klebsiella pneumoniae 1 Klebsiella pneumoniae 1 Klebsiella pneumoniae 1 NA

GCF_002935215 Contig Klebsiella pneumoniae pneumoniae CRK0351 Klebsiella pneumoniae 1 Klebsiella pneumoniae 1 Klebsiella pneumoniae 1 NA

GCF_002935245 Contig Klebsiella pneumoniae pneumoniae CRK0352 Klebsiella pneumoniae 1 Klebsiella pneumoniae 1 Klebsiella pneumoniae 1 NA

GCF_002935265 Contig Klebsiella pneumoniae pneumoniae CRK0354 Klebsiella pneumoniae 1 Klebsiella pneumoniae 1 Klebsiella pneumoniae 1 NA

GCF_002935285 Contig Klebsiella pneumoniae pneumoniae CRK0356 Klebsiella pneumoniae 1 Klebsiella pneumoniae 1 Klebsiella pneumoniae 1 NA

GCF_002935305 Contig Klebsiella pneumoniae pneumoniae CRK0359 Klebsiella pneumoniae 1 Klebsiella pneumoniae 1 Klebsiella pneumoniae 1 NA

GCF_002935325 Contig Klebsiella pneumoniae pneumoniae CRK0357 Klebsiella pneumoniae 1 Klebsiella pneumoniae 1 Klebsiella pneumoniae 1 NA

GCF_002935345 Contig Klebsiella pneumoniae pneumoniae CRK0361 Klebsiella pneumoniae 1 Klebsiella pneumoniae 1 Klebsiella pneumoniae 1 NA

GCF_002935365 Contig Klebsiella pneumoniae pneumoniae CRK0362 Klebsiella pneumoniae 1 Klebsiella pneumoniae 1 Klebsiella pneumoniae 1 NA

GCF_002935385 Contig Klebsiella pneumoniae pneumoniae CRK0363 Klebsiella pneumoniae 1 Klebsiella pneumoniae 1 Klebsiella pneumoniae 1 NA

GCF_002935405 Contig Klebsiella pneumoniae pneumoniae CRK0364 Klebsiella pneumoniae 1 Klebsiella pneumoniae 1 Klebsiella pneumoniae 1 NA

GCF_002935425 Contig Klebsiella pneumoniae pneumoniae CRK0365 Klebsiella pneumoniae 1 Klebsiella pneumoniae 1 Klebsiella pneumoniae 1 NA

GCF_002935445 Contig Klebsiella pneumoniae pneumoniae CRK0366 Klebsiella pneumoniae 1 Klebsiella pneumoniae 1 Klebsiella pneumoniae 1 NA

GCF_002935485 Contig Klebsiella pneumoniae pneumoniae CRK0368 Klebsiella pneumoniae 1 Klebsiella pneumoniae 1 Klebsiella pneumoniae 1 NA

GCF_002935505 Contig Klebsiella pneumoniae pneumoniae CRK0371 Klebsiella pneumoniae 1 Klebsiella pneumoniae 1 Klebsiella pneumoniae 1 NA

GCF_002935515 Contig Klebsiella pneumoniae pneumoniae CRK0301 Klebsiella pneumoniae 1 Klebsiella pneumoniae 1 Klebsiella pneumoniae 1 NA

GCF_002935545 Contig Klebsiella pneumoniae pneumoniae CRK0377 Klebsiella pneumoniae 1 Klebsiella pneumoniae 1 Klebsiella pneumoniae 1 NA

GCF_002935555 Contig Klebsiella pneumoniae pneumoniae CRK0374 Klebsiella pneumoniae 1 Klebsiella pneumoniae 1 Klebsiella pneumoniae 1 NA

GCF_002935585 Contig Klebsiella pneumoniae pneumoniae CRK0379 Klebsiella pneumoniae 1 Klebsiella pneumoniae 1 Klebsiella pneumoniae 1 NA

GCF_002935605 Contig Klebsiella pneumoniae pneumoniae CRK0380 Klebsiella pneumoniae 1 Klebsiella pneumoniae 1 Klebsiella pneumoniae 1 NA

GCF_002935625 Contig Klebsiella pneumoniae pneumoniae CRK0382 Klebsiella pneumoniae 1 Klebsiella pneumoniae 1 Klebsiella pneumoniae 1 NA

GCF_002935645 Contig Klebsiella pneumoniae pneumoniae CRK0381 Klebsiella pneumoniae 1 Klebsiella pneumoniae 1 Klebsiella pneumoniae 1 NA

GCF_002935665 Contig Klebsiella pneumoniae pneumoniae CRK0372 Klebsiella pneumoniae 1 Klebsiella pneumoniae 1 Klebsiella pneumoniae 1 NA

GCF_002935685 Contig Klebsiella variicola CRK0383 Klebsiella variicola Klebsiella variicola Klebsiella variicola NA

GCF_002935705 Contig Klebsiella pneumoniae pneumoniae CRK0388 Klebsiella pneumoniae 1 Klebsiella pneumoniae 1 Klebsiella pneumoniae 1 NA

GCF_002935725 Contig Klebsiella pneumoniae pneumoniae CRK0384 Klebsiella pneumoniae 1 Klebsiella pneumoniae 1 Klebsiella pneumoniae 1 NA

GCF_002935745 Contig Klebsiella pneumoniae pneumoniae CRK0387 Klebsiella pneumoniae 1 Klebsiella pneumoniae 1 Klebsiella pneumoniae 1 NA

GCF_002935765 Contig Klebsiella pneumoniae pneumoniae CRK0389 Klebsiella pneumoniae 1 Klebsiella pneumoniae 1 Klebsiella pneumoniae 1 NA

GCF_002935785 Contig Klebsiella pneumoniae pneumoniae CRK0338 Klebsiella pneumoniae 1 Klebsiella pneumoniae 1 Klebsiella pneumoniae 1 NA

GCF_002935795 Contig Klebsiella pneumoniae pneumoniae CRK0339 Klebsiella pneumoniae 1 Klebsiella pneumoniae 1 Klebsiella pneumoniae 1 NA

GCF_002935805 Contig Klebsiella pneumoniae pneumoniae CRK0341 Klebsiella pneumoniae 1 Klebsiella pneumoniae 1 Klebsiella pneumoniae 1 NA

GCF_002935815 Contig Klebsiella pneumoniae pneumoniae CRK0344 Klebsiella pneumoniae 1 Klebsiella pneumoniae 1 Klebsiella pneumoniae 1 NA

GCF_002935865 Contig Klebsiella pneumoniae pneumoniae CRK0303 Klebsiella pneumoniae 1 Klebsiella pneumoniae 1 Klebsiella pneumoniae 1 NA

GCF_002935875 Contig Klebsiella pneumoniae pneumoniae CRK0304 Klebsiella pneumoniae 1 Klebsiella pneumoniae 1 Klebsiella pneumoniae 1 NA

GCF_002935905 Contig Klebsiella pneumoniae pneumoniae CRK0305 Klebsiella pneumoniae 1 Klebsiella pneumoniae 1 Klebsiella pneumoniae 1 NA

GCF_002935915 Contig Klebsiella pneumoniae pneumoniae CRK0307 Klebsiella pneumoniae 1 Klebsiella pneumoniae 1 Klebsiella pneumoniae 1 NA

GCF_002935945 Contig Klebsiella pneumoniae pneumoniae CRK0302 Klebsiella pneumoniae 1 Klebsiella pneumoniae 1 Klebsiella pneumoniae 1 NA

GCF_002935955 Contig Klebsiella pneumoniae pneumoniae CRK0308 Klebsiella pneumoniae 1 Klebsiella pneumoniae 1 Klebsiella pneumoniae 1 NA

GCF_002935985 Contig Klebsiella pneumoniae pneumoniae CRK0310 Klebsiella pneumoniae 1 Klebsiella pneumoniae 1 Klebsiella pneumoniae 1 NA

GCF_002936005 Contig Klebsiella quasipneumoniae quasipneumoniae CRK0311 Klebsiella quasipneumoniae 2 Klebsiella quasipneumoniae 2 Klebsiella quasipneumoniae NA

GCF_002936025 Contig Klebsiella pneumoniae pneumoniae CRK0313 Klebsiella pneumoniae 1 Klebsiella pneumoniae 1 Klebsiella pneumoniae 1 NA

GCF_002936165 Contig Klebsiella pneumoniae pneumoniae CRK0326 Klebsiella pneumoniae 1 Klebsiella pneumoniae 1 Klebsiella pneumoniae 1 NA

GCF_002936185 Contig Klebsiella pneumoniae pneumoniae CRK0330 Klebsiella pneumoniae 1 Klebsiella pneumoniae 1 Klebsiella pneumoniae 1 NA

GCF_002936205 Contig Klebsiella pneumoniae pneumoniae CRK0328 Klebsiella pneumoniae 1 Klebsiella pneumoniae 1 Klebsiella pneumoniae 1 NA

GCF_002936255 Contig Klebsiella pneumoniae pneumoniae CRK0345 Klebsiella pneumoniae 1 Klebsiella pneumoniae 1 Klebsiella pneumoniae 1 NA

GCF_002936265 Contig Klebsiella pneumoniae pneumoniae CRK0331 Klebsiella pneumoniae 1 Klebsiella pneumoniae 1 Klebsiella pneumoniae 1 NA

GCF_002936295 Contig Klebsiella pneumoniae pneumoniae CRK0335 Klebsiella pneumoniae 1 Klebsiella pneumoniae 1 Klebsiella pneumoniae 1 NA

GCF_002936315 Contig Klebsiella pneumoniae pneumoniae CRK0336 Klebsiella pneumoniae 1 Klebsiella pneumoniae 1 Klebsiella pneumoniae 1 NA

GCF_002936335 Contig Klebsiella pneumoniae pneumoniae CRK0347 Klebsiella pneumoniae 1 Klebsiella pneumoniae 1 Klebsiella pneumoniae 1 NA

GCF_002936345 Contig Klebsiella pneumoniae pneumoniae CRK0348 Klebsiella pneumoniae 1 Klebsiella pneumoniae 1 Klebsiella pneumoniae 1 NA

GCF_002936375 Contig Klebsiella pneumoniae pneumoniae CRK0355 Klebsiella pneumoniae 1 Klebsiella pneumoniae 1 Klebsiella pneumoniae 1 NA

GCF_002936395 Contig Klebsiella pneumoniae pneumoniae CRK0353 Klebsiella pneumoniae 1 Klebsiella pneumoniae 1 Klebsiella pneumoniae 1 NA

GCF_002936415 Contig Klebsiella pneumoniae pneumoniae CRK0360 Klebsiella pneumoniae 1 Klebsiella pneumoniae 1 Klebsiella pneumoniae 1 NA

GCF_002936435 Contig Klebsiella pneumoniae pneumoniae CRK0369 Klebsiella pneumoniae 1 Klebsiella pneumoniae 1 Klebsiella pneumoniae 1 NA

GCF_002936455 Contig Klebsiella pneumoniae pneumoniae CRK0358 Klebsiella pneumoniae 1 Klebsiella pneumoniae 1 Klebsiella pneumoniae 1 NA

GCF_002936475 Contig Klebsiella pneumoniae pneumoniae CRK0373 Klebsiella pneumoniae 1 Klebsiella pneumoniae 1 Klebsiella pneumoniae 1 NA

GCF_002936515 Contig Klebsiella pneumoniae pneumoniae CRK0370 Klebsiella pneumoniae 1 Klebsiella pneumoniae 1 Klebsiella pneumoniae 1 NA

GCF_002936535 Contig Klebsiella pneumoniae pneumoniae CRK0385 Klebsiella pneumoniae 1 Klebsiella pneumoniae 1 Klebsiella pneumoniae 1 NA

GCF_002936545 Contig Klebsiella pneumoniae pneumoniae CRK0376 Klebsiella pneumoniae 1 Klebsiella pneumoniae 1 Klebsiella pneumoniae 1 NA

GCF_002936565 Contig Klebsiella pneumoniae pneumoniae CRK0378 Klebsiella pneumoniae 1 Klebsiella pneumoniae 1 Klebsiella pneumoniae 1 NA

GCF_002936595 Contig Klebsiella pneumoniae pneumoniae CRK0386 Klebsiella pneumoniae 1 Klebsiella pneumoniae 1 Klebsiella pneumoniae 1 NA

GCF_002936615 Contig Klebsiella pneumoniae pneumoniae CRK0337 Klebsiella pneumoniae 1 Klebsiella pneumoniae 1 Klebsiella pneumoniae 1 NA

GCF_002936635 Contig Klebsiella pneumoniae pneumoniae CRK0340 Klebsiella pneumoniae 1 Klebsiella pneumoniae 1 Klebsiella pneumoniae 1 NA

GCF_002936645 Contig Klebsiella pneumoniae pneumoniae CRK0342 Klebsiella pneumoniae 1 Klebsiella pneumoniae 1 Klebsiella pneumoniae 1 NA

GCF_002936675 Contig Klebsiella pneumoniae pneumoniae CRK0306 Klebsiella pneumoniae 1 Klebsiella pneumoniae 1 Klebsiella pneumoniae 1 NA

GCF_002936685 Contig Klebsiella pneumoniae pneumoniae CRK0343 Klebsiella pneumoniae 1 Klebsiella pneumoniae 1 Klebsiella pneumoniae 1 NA

GCF_002936705 Contig Klebsiella pneumoniae pneumoniae CRK0309 Klebsiella pneumoniae 1 Klebsiella pneumoniae 1 Klebsiella pneumoniae 1 NA

GCF_002936735 Contig Klebsiella pneumoniae pneumoniae CRK0314 Klebsiella pneumoniae 1 Klebsiella pneumoniae 1 Klebsiella pneumoniae 1 NA

GCF_002936755 Contig Klebsiella pneumoniae pneumoniae CRK0312 Klebsiella pneumoniae 1 Klebsiella pneumoniae 1 Klebsiella pneumoniae 1 NA

GCF_002936875 Contig Klebsiella pneumoniae pneumoniae CRK0327 Klebsiella pneumoniae 1 Klebsiella pneumoniae 1 Klebsiella pneumoniae 1 NA

GCF_002936895 Contig Klebsiella pneumoniae pneumoniae CRK0333 Klebsiella pneumoniae 1 Klebsiella pneumoniae 1 Klebsiella pneumoniae 1 NA

GCF_002936905 Contig Klebsiella pneumoniae pneumoniae CRK0332 Klebsiella pneumoniae 1 Klebsiella pneumoniae 1 Klebsiella pneumoniae 1 NA

GCF_002936935 Contig Klebsiella pneumoniae pneumoniae CRK0334 Klebsiella pneumoniae 1 Klebsiella pneumoniae 1 Klebsiella pneumoniae 1 NA

GCF_002941005 Contig Klebsiella pneumoniae pneumoniae CRK0367 Klebsiella pneumoniae 1 Klebsiella pneumoniae 1 Klebsiella pneumoniae 1 NA

GCF_002941095 Contig Klebsiella pneumoniae YH17175 Klebsiella pneumoniae 1 Klebsiella pneumoniae 1 Klebsiella pneumoniae 1 NA

GCF_002944845 Complete Klebsiella pneumoniae NUHL30457 Klebsiella pneumoniae 1 Klebsiella pneumoniae 1 Klebsiella pneumoniae 1 NA

GCF_002947505 Complete Klebsiella oxytoca AR0028 Klebsiella michiganensis 1 Klebsiella michiganensis 1 Klebsiella michiganensis 1 NA

GCF_002948565 Complete Klebsiella pneumoniae AR0066 Klebsiella pneumoniae 1 Klebsiella pneumoniae 1 Klebsiella pneumoniae 1 NA

GCF_002948835 Complete Klebsiella aerogenes AR0062 Klebsiella aerogenes 1 Klebsiella aerogenes Klebsiella aerogenes NA

GCF_002951555 Complete Klebsiella pneumoniae pneumoniae BR7 Klebsiella pneumoniae 1 Klebsiella pneumoniae 1 Klebsiella pneumoniae 1 NA

GCF_002951595 Complete Klebsiella pneumoniae pneumoniae BR21 Klebsiella pneumoniae 1 Klebsiella pneumoniae 1 Klebsiella pneumoniae 1 NA

GCF_002953295 Complete Klebsiella quasipneumoniae FDAARGOS93 Klebsiella quasipneumoniae 1 Klebsiella quasipneumoniae 1 Klebsiella quasipneumoniae NA

GCF_002966875 Complete Klebsiella pneumoniae HZW25 Klebsiella pneumoniae 1 Klebsiella pneumoniae 1 Klebsiella pneumoniae 1 NA

GCF_002967105 Contig Klebsiella quasipneumoniae YH17268 Klebsiella quasipneumoniae 2 Klebsiella quasipneumoniae 2 Klebsiella quasipneumoniae NA

GCF_002967155 Contig Klebsiella quasipneumoniae YH17269 Klebsiella quasipneumoniae 2 Klebsiella quasipneumoniae 2 Klebsiella quasipneumoniae NA

GCF_002967795 Complete Klebsiella pneumoniae BA2275 Klebsiella pneumoniae 1 Klebsiella pneumoniae 1 Klebsiella pneumoniae 1 NA

GCF_002967835 Scaffold Klebsiella pneumoniae BP737 Klebsiella pneumoniae 1 Klebsiella pneumoniae 1 Klebsiella pneumoniae 1 NA

GCF_002968475 Complete Klebsiella pneumoniae AR0363 Klebsiella pneumoniae 1 Klebsiella pneumoniae 1 Klebsiella pneumoniae 1 NA

GCF_002968495 Complete Klebsiella pneumoniae AR0361 Klebsiella pneumoniae 1 Klebsiella pneumoniae 1 Klebsiella pneumoniae 1 NA

GCF_002969145 Contig Klebsiella pneumoniae SCPM-O-B7852 KpB-2580 Klebsiella pneumoniae 1 Klebsiella pneumoniae 1 Klebsiella pneumoniae 1 NA

GCF_002969255 Contig Klebsiella pneumoniae pneumoniae CRK0195 Klebsiella pneumoniae 1 Klebsiella pneumoniae 1 Klebsiella pneumoniae 1 NA

GCF_002969265 Contig Klebsiella pneumoniae pneumoniae CRK0196 Klebsiella pneumoniae 1 Klebsiella pneumoniae 1 Klebsiella pneumoniae 1 NA

GCF_002969295 Contig Klebsiella pneumoniae pneumoniae CRK0193 Klebsiella pneumoniae 1 Klebsiella pneumoniae 1 Klebsiella pneumoniae 1 NA

GCF_002969315 Contig Klebsiella pneumoniae pneumoniae CRK0194 Klebsiella pneumoniae 1 Klebsiella pneumoniae 1 Klebsiella pneumoniae 1 NA

GCF_002969335 Contig Klebsiella pneumoniae pneumoniae CRK0197 Klebsiella pneumoniae 1 Klebsiella pneumoniae 1 Klebsiella pneumoniae 1 NA

GCF_002969355 Contig Klebsiella pneumoniae pneumoniae CRK0198 Klebsiella pneumoniae 1 Klebsiella pneumoniae 1 Klebsiella pneumoniae 1 NA

GCF_002969375 Contig Klebsiella pneumoniae pneumoniae CRK0199 Klebsiella pneumoniae 1 Klebsiella pneumoniae 1 Klebsiella pneumoniae 1 NA

GCF_002969385 Contig Klebsiella pneumoniae pneumoniae CRK0203 Klebsiella pneumoniae 1 Klebsiella pneumoniae 1 Klebsiella pneumoniae 1 NA

GCF_002969405 Contig Klebsiella pneumoniae pneumoniae CRK0202 Klebsiella pneumoniae 1 Klebsiella pneumoniae 1 Klebsiella pneumoniae 1 NA

GCF_002969435 Contig Klebsiella pneumoniae pneumoniae CRK0206 Klebsiella pneumoniae 1 Klebsiella pneumoniae 1 Klebsiella pneumoniae 1 NA

GCF_002969455 Contig Klebsiella pneumoniae pneumoniae CRK0207 Klebsiella pneumoniae 1 Klebsiella pneumoniae 1 Klebsiella pneumoniae 1 NA

GCF_002969475 Contig Klebsiella pneumoniae pneumoniae CRK0213 Klebsiella pneumoniae 1 Klebsiella pneumoniae 1 Klebsiella pneumoniae 1 NA

GCF_002969495 Contig Klebsiella pneumoniae pneumoniae CRK0209 Klebsiella pneumoniae 1 Klebsiella pneumoniae 1 Klebsiella pneumoniae 1 NA

GCF_002969515 Contig Klebsiella pneumoniae pneumoniae CRK0201 Klebsiella pneumoniae 1 Klebsiella pneumoniae 1 Klebsiella pneumoniae 1 NA

GCF_002969535 Contig Klebsiella pneumoniae pneumoniae CRK0215 Klebsiella pneumoniae 1 Klebsiella pneumoniae 1 Klebsiella pneumoniae 1 NA

GCF_002969555 Contig Klebsiella pneumoniae pneumoniae CRK0192 Klebsiella pneumoniae 1 Klebsiella pneumoniae 1 Klebsiella pneumoniae 1 NA

GCF_002969575 Contig Klebsiella pneumoniae pneumoniae CRK0208 Klebsiella pneumoniae 1 Klebsiella pneumoniae 1 Klebsiella pneumoniae 1 NA

GCF_002969595 Contig Klebsiella pneumoniae pneumoniae CRK0210 Klebsiella pneumoniae 1 Klebsiella pneumoniae 1 Klebsiella pneumoniae 1 NA

GCF_002969615 Contig Klebsiella pneumoniae pneumoniae CRK0214 Klebsiella pneumoniae 1 Klebsiella pneumoniae 1 Klebsiella pneumoniae 1 NA

GCF_002969635 Contig Klebsiella pneumoniae pneumoniae CRK0217 Klebsiella pneumoniae 1 Klebsiella pneumoniae 1 Klebsiella pneumoniae 1 NA

GCF_002969655 Contig Klebsiella pneumoniae pneumoniae CRK0218 Klebsiella pneumoniae 1 Klebsiella pneumoniae 1 Klebsiella pneumoniae 1 NA

GCF_002969675 Contig Klebsiella pneumoniae pneumoniae CRK0216 Klebsiella pneumoniae 1 Klebsiella pneumoniae 1 Klebsiella pneumoniae 1 NA

GCF_002969695 Contig Klebsiella pneumoniae pneumoniae CRK0222 Klebsiella pneumoniae 1 Klebsiella pneumoniae 1 Klebsiella pneumoniae 1 NA

GCF_002969715 Contig Klebsiella pneumoniae pneumoniae CRK0219 Klebsiella pneumoniae 1 Klebsiella pneumoniae 1 Klebsiella pneumoniae 1 NA

GCF_002969735 Contig Klebsiella pneumoniae pneumoniae CRK0237 Klebsiella pneumoniae 1 Klebsiella pneumoniae 1 Klebsiella pneumoniae 1 NA

GCF_002969755 Contig Klebsiella pneumoniae pneumoniae CRK0229 Klebsiella pneumoniae 1 Klebsiella pneumoniae 1 Klebsiella pneumoniae 1 NA

GCF_002969775 Contig Klebsiella pneumoniae pneumoniae CRK0244 Klebsiella pneumoniae 1 Klebsiella pneumoniae 1 Klebsiella pneumoniae 1 NA

GCF_002969795 Contig Klebsiella pneumoniae pneumoniae CRK0241 Klebsiella pneumoniae 1 Klebsiella pneumoniae 1 Klebsiella pneumoniae 1 NA

GCF_002969815 Contig Klebsiella pneumoniae pneumoniae CRK0246 Klebsiella pneumoniae 1 Klebsiella pneumoniae 1 Klebsiella pneumoniae 1 NA

GCF_002969835 Contig Klebsiella pneumoniae pneumoniae CRK0245 Klebsiella pneumoniae 1 Klebsiella pneumoniae 1 Klebsiella pneumoniae 1 NA

GCF_002969855 Contig Klebsiella pneumoniae pneumoniae CRK0257 Klebsiella pneumoniae 1 Klebsiella pneumoniae 1 Klebsiella pneumoniae 1 NA

GCF_002969875 Contig Klebsiella pneumoniae pneumoniae CRK0260 Klebsiella pneumoniae 1 Klebsiella pneumoniae 1 Klebsiella pneumoniae 1 NA

GCF_002969895 Contig Klebsiella pneumoniae pneumoniae CRK0251 Klebsiella pneumoniae 1 Klebsiella pneumoniae 1 Klebsiella pneumoniae 1 NA

GCF_002969915 Contig Klebsiella pneumoniae pneumoniae CRK0262 Klebsiella pneumoniae 1 Klebsiella pneumoniae 1 Klebsiella pneumoniae 1 NA

GCF_002969935 Contig Klebsiella pneumoniae pneumoniae CRK0253 Klebsiella pneumoniae 1 Klebsiella pneumoniae 1 Klebsiella pneumoniae 1 NA

GCF_002969955 Contig Klebsiella pneumoniae pneumoniae CRK0261 Klebsiella pneumoniae 1 Klebsiella pneumoniae 1 Klebsiella pneumoniae 1 NA

GCF_002969965 Contig Klebsiella pneumoniae pneumoniae CRK0268 Klebsiella pneumoniae 1 Klebsiella pneumoniae 1 Klebsiella pneumoniae 1 NA

GCF_002969975 Contig Klebsiella pneumoniae pneumoniae CRK0267 Klebsiella pneumoniae 1 Klebsiella pneumoniae 1 Klebsiella pneumoniae 1 NA

GCF_002970015 Contig Klebsiella pneumoniae pneumoniae CRK0269 Klebsiella pneumoniae 1 Klebsiella pneumoniae 1 Klebsiella pneumoniae 1 NA

GCF_002970035 Contig Klebsiella pneumoniae pneumoniae CRK0272 Klebsiella pneumoniae 1 Klebsiella pneumoniae 1 Klebsiella pneumoniae 1 NA

GCF_002970055 Contig Klebsiella pneumoniae pneumoniae CRK0277 Klebsiella pneumoniae 1 Klebsiella pneumoniae 1 Klebsiella pneumoniae 1 NA

GCF_002970075 Contig Klebsiella pneumoniae pneumoniae CRK0278 Klebsiella pneumoniae 1 Klebsiella pneumoniae 1 Klebsiella pneumoniae 1 NA

GCF_002970085 Contig Klebsiella pneumoniae pneumoniae CRK0279 Klebsiella pneumoniae 1 Klebsiella pneumoniae 1 Klebsiella pneumoniae 1 NA

GCF_002970115 Contig Klebsiella pneumoniae pneumoniae CRK0280 Klebsiella pneumoniae 1 Klebsiella pneumoniae 1 Klebsiella pneumoniae 1 NA

GCF_002970135 Contig Klebsiella pneumoniae pneumoniae CRK0282 Klebsiella pneumoniae 1 Klebsiella pneumoniae 1 Klebsiella pneumoniae 1 NA

GCF_002970155 Contig Klebsiella pneumoniae pneumoniae CRK0283 Klebsiella pneumoniae 1 Klebsiella pneumoniae 1 Klebsiella pneumoniae 1 NA

GCF_002970175 Contig Klebsiella pneumoniae pneumoniae CRK0284 Klebsiella pneumoniae 1 Klebsiella pneumoniae 1 Klebsiella pneumoniae 1 NA

GCF_002970195 Contig Klebsiella pneumoniae pneumoniae CRK0285 Klebsiella pneumoniae 1 Klebsiella pneumoniae 1 Klebsiella pneumoniae 1 NA

GCF_002970215 Contig Klebsiella pneumoniae pneumoniae CRK0286 Klebsiella pneumoniae 1 Klebsiella pneumoniae 1 Klebsiella pneumoniae 1 NA

GCF_002970235 Contig Klebsiella pneumoniae pneumoniae CRK0287 Klebsiella pneumoniae 1 Klebsiella pneumoniae 1 Klebsiella pneumoniae 1 NA

GCF_002970255 Contig Klebsiella pneumoniae pneumoniae CRK0293 Klebsiella pneumoniae 1 Klebsiella pneumoniae 1 Klebsiella pneumoniae 1 NA

GCF_002970275 Contig Klebsiella pneumoniae pneumoniae CRK0289 Klebsiella pneumoniae 1 Klebsiella pneumoniae 1 Klebsiella pneumoniae 1 NA

GCF_002970295 Contig Klebsiella pneumoniae pneumoniae CRK0294 Klebsiella pneumoniae 1 Klebsiella pneumoniae 1 Klebsiella pneumoniae 1 NA

GCF_002970315 Contig Klebsiella pneumoniae pneumoniae CRK0295 Klebsiella pneumoniae 1 Klebsiella pneumoniae 1 Klebsiella pneumoniae 1 NA

GCF_002970335 Contig Klebsiella pneumoniae pneumoniae CRK0296 Klebsiella pneumoniae 1 Klebsiella pneumoniae 1 Klebsiella pneumoniae 1 NA

GCF_002970355 Contig Klebsiella pneumoniae pneumoniae CRK0297 Klebsiella pneumoniae 1 Klebsiella pneumoniae 1 Klebsiella pneumoniae 1 NA

GCF_002970375 Complete Klebsiella pneumoniae pneumoniae CRK0298 Klebsiella pneumoniae 1 Klebsiella pneumoniae 1 Klebsiella pneumoniae 1 NA

GCF_002970395 Contig Klebsiella pneumoniae pneumoniae CRK0299 Klebsiella pneumoniae 1 Klebsiella pneumoniae 1 Klebsiella pneumoniae 1 NA

GCF_002970405 Contig Klebsiella pneumoniae pneumoniae CRK0300 Klebsiella pneumoniae 1 Klebsiella pneumoniae 1 Klebsiella pneumoniae 1 NA

GCF_002970425 Contig Klebsiella pneumoniae pneumoniae CRK0220 Klebsiella pneumoniae 1 Klebsiella pneumoniae 1 Klebsiella pneumoniae 1 NA

GCF_002970455 Contig Klebsiella pneumoniae pneumoniae CRK0223 Klebsiella pneumoniae 1 Klebsiella pneumoniae 1 Klebsiella pneumoniae 1 NA

GCF_002970475 Contig Klebsiella pneumoniae pneumoniae CRK0221 Klebsiella pneumoniae 1 Klebsiella pneumoniae 1 Klebsiella pneumoniae 1 NA

GCF_002970485 Contig Klebsiella pneumoniae pneumoniae CRK0224 Klebsiella pneumoniae 1 Klebsiella pneumoniae 1 Klebsiella pneumoniae 1 NA

GCF_002970515 Contig Klebsiella pneumoniae pneumoniae CRK0228 Klebsiella pneumoniae 1 Klebsiella pneumoniae 1 Klebsiella pneumoniae 1 NA

GCF_002970525 Contig Klebsiella pneumoniae pneumoniae CRK0230 Klebsiella pneumoniae 1 Klebsiella pneumoniae 1 Klebsiella pneumoniae 1 NA

GCF_002970555 Contig Klebsiella pneumoniae pneumoniae CRK0232 Klebsiella pneumoniae 1 Klebsiella pneumoniae 1 Klebsiella pneumoniae 1 NA

GCF_002970575 Contig Klebsiella pneumoniae pneumoniae CRK0233 Klebsiella pneumoniae 1 Klebsiella pneumoniae 1 Klebsiella pneumoniae 1 NA

GCF_002970595 Contig Klebsiella pneumoniae pneumoniae CRK0235 Klebsiella pneumoniae 1 Klebsiella pneumoniae 1 Klebsiella pneumoniae 1 NA

GCF_002970615 Contig Klebsiella pneumoniae pneumoniae CRK0236 Klebsiella pneumoniae 1 Klebsiella pneumoniae 1 Klebsiella pneumoniae 1 NA

GCF_002970625 Contig Klebsiella pneumoniae pneumoniae CRK0238 Klebsiella pneumoniae 1 Klebsiella pneumoniae 1 Klebsiella pneumoniae 1 NA

GCF_002970655 Contig Klebsiella pneumoniae pneumoniae CRK0243 Klebsiella pneumoniae 1 Klebsiella pneumoniae 1 Klebsiella pneumoniae 1 NA

GCF_002970675 Contig Klebsiella pneumoniae pneumoniae CRK0247 Klebsiella pneumoniae 1 Klebsiella pneumoniae 1 Klebsiella pneumoniae 1 NA

GCF_002970695 Contig Klebsiella pneumoniae pneumoniae CRK0252 Klebsiella pneumoniae 1 Klebsiella pneumoniae 1 Klebsiella pneumoniae 1 NA

GCF_002970715 Contig Klebsiella pneumoniae pneumoniae CRK0255 Klebsiella pneumoniae 1 Klebsiella pneumoniae 1 Klebsiella pneumoniae 1 NA

GCF_002970735 Contig Klebsiella pneumoniae pneumoniae CRK0256 Klebsiella pneumoniae 1 Klebsiella pneumoniae 1 Klebsiella pneumoniae 1 NA

GCF_002970745 Contig Klebsiella pneumoniae pneumoniae CRK0258 Klebsiella pneumoniae 1 Klebsiella pneumoniae 1 Klebsiella pneumoniae 1 NA

GCF_002970755 Contig Klebsiella pneumoniae pneumoniae CRK0259 Klebsiella pneumoniae 1 Klebsiella pneumoniae 1 Klebsiella pneumoniae 1 NA

GCF_002970785 Contig Klebsiella pneumoniae pneumoniae CRK0263 Klebsiella pneumoniae 1 Klebsiella pneumoniae 1 Klebsiella pneumoniae 1 NA

GCF_002970815 Contig Klebsiella pneumoniae pneumoniae CRK0264 Klebsiella pneumoniae 1 Klebsiella pneumoniae 1 Klebsiella pneumoniae 1 NA

GCF_002970835 Contig Klebsiella pneumoniae pneumoniae CRK0281 Klebsiella pneumoniae 1 Klebsiella pneumoniae 1 Klebsiella pneumoniae 1 NA

GCF_002970855 Contig Klebsiella pneumoniae pneumoniae CRK0290 Klebsiella pneumoniae 1 Klebsiella pneumoniae 1 Klebsiella pneumoniae 1 NA

GCF_002970875 Contig Klebsiella pneumoniae pneumoniae CRK0275 Klebsiella pneumoniae 1 Klebsiella pneumoniae 1 Klebsiella pneumoniae 1 NA

GCF_002970895 Complete Klebsiella pneumoniae KPHS1249 Klebsiella pneumoniae 1 Klebsiella pneumoniae 1 Klebsiella pneumoniae 1 NA

GCF_002973835 Contig Klebsiella pneumoniae pneumoniae CRK0212 Klebsiella pneumoniae 1 Klebsiella pneumoniae 1 Klebsiella pneumoniae 1 NA

GCF_002973855 Contig Klebsiella pneumoniae pneumoniae CRK0200 Klebsiella pneumoniae 1 Klebsiella pneumoniae 1 Klebsiella pneumoniae 1 NA

GCF_002973875 Contig Klebsiella pneumoniae pneumoniae CRK0211 Klebsiella pneumoniae 1 Klebsiella pneumoniae 1 Klebsiella pneumoniae 1 NA

GCF_002973895 Contig Klebsiella pneumoniae pneumoniae CRK0204 Klebsiella pneumoniae 1 Klebsiella pneumoniae 1 Klebsiella pneumoniae 1 NA

GCF_002973915 Contig Klebsiella pneumoniae pneumoniae CRK0225 Klebsiella pneumoniae 1 Klebsiella pneumoniae 1 Klebsiella pneumoniae 1 NA

GCF_002973925 Contig Klebsiella pneumoniae pneumoniae CRK0205 Klebsiella pneumoniae 1 Klebsiella pneumoniae 1 Klebsiella pneumoniae 1 NA

GCF_002973955 Contig Klebsiella pneumoniae pneumoniae CRK0231 Klebsiella pneumoniae 1 Klebsiella pneumoniae 1 Klebsiella pneumoniae 1 NA

GCF_002973975 Contig Klebsiella pneumoniae pneumoniae CRK0226 Klebsiella pneumoniae 1 Klebsiella pneumoniae 1 Klebsiella pneumoniae 1 NA

GCF_002973995 Contig Klebsiella pneumoniae pneumoniae CRK0234 Klebsiella pneumoniae 1 Klebsiella pneumoniae 1 Klebsiella pneumoniae 1 NA

GCF_002974005 Contig Klebsiella pneumoniae pneumoniae CRK0239 Klebsiella pneumoniae 1 Klebsiella pneumoniae 1 Klebsiella pneumoniae 1 NA

GCF_002974015 Contig Klebsiella pneumoniae pneumoniae CRK0249 Klebsiella pneumoniae 1 Klebsiella pneumoniae 1 Klebsiella pneumoniae 1 NA

GCF_002974055 Contig Klebsiella pneumoniae pneumoniae CRK0242 Klebsiella pneumoniae 1 Klebsiella pneumoniae 1 Klebsiella pneumoniae 1 NA

GCF_002974075 Contig Klebsiella pneumoniae pneumoniae CRK0250 Klebsiella pneumoniae 1 Klebsiella pneumoniae 1 Klebsiella pneumoniae 1 NA

GCF_002974095 Contig Klebsiella pneumoniae pneumoniae CRK0266 Klebsiella pneumoniae 1 Klebsiella pneumoniae 1 Klebsiella pneumoniae 1 NA

GCF_002974115 Contig Klebsiella pneumoniae pneumoniae CRK0265 Klebsiella pneumoniae 1 Klebsiella pneumoniae 1 Klebsiella pneumoniae 1 NA

GCF_002974135 Contig Klebsiella pneumoniae pneumoniae CRK0270 Klebsiella pneumoniae 1 Klebsiella pneumoniae 1 Klebsiella pneumoniae 1 NA

GCF_002974155 Contig Klebsiella pneumoniae pneumoniae CRK0274 Klebsiella pneumoniae 1 Klebsiella pneumoniae 1 Klebsiella pneumoniae 1 NA

GCF_002974165 Contig Klebsiella pneumoniae pneumoniae CRK0271 Klebsiella pneumoniae 1 Klebsiella pneumoniae 1 Klebsiella pneumoniae 1 NA

GCF_002974195 Contig Klebsiella pneumoniae pneumoniae CRK0276 Klebsiella pneumoniae 1 Klebsiella pneumoniae 1 Klebsiella pneumoniae 1 NA

GCF_002974215 Contig Klebsiella pneumoniae pneumoniae CRK0292 Klebsiella pneumoniae 1 Klebsiella pneumoniae 1 Klebsiella pneumoniae 1 NA

GCF_002974235 Contig Klebsiella pneumoniae pneumoniae CRK0288 Klebsiella pneumoniae 1 Klebsiella pneumoniae 1 Klebsiella pneumoniae 1 NA

GCF_002974255 Contig Klebsiella pneumoniae pneumoniae CRK0227 Klebsiella pneumoniae 1 Klebsiella pneumoniae 1 Klebsiella pneumoniae 1 NA

GCF_002974275 Contig Klebsiella pneumoniae pneumoniae CRK0248 Klebsiella pneumoniae 1 Klebsiella pneumoniae 1 Klebsiella pneumoniae 1 NA

GCF_002974285 Contig Klebsiella pneumoniae pneumoniae CRK0240 Klebsiella pneumoniae 1 Klebsiella pneumoniae 1 Klebsiella pneumoniae 1 NA

GCF_002974315 Contig Klebsiella pneumoniae pneumoniae CRK0254 Klebsiella pneumoniae 1 Klebsiella pneumoniae 1 Klebsiella pneumoniae 1 NA

GCF_002974335 Contig Klebsiella pneumoniae pneumoniae CRK0273 Klebsiella pneumoniae 1 Klebsiella pneumoniae 1 Klebsiella pneumoniae 1 NA

GCF_002976515 Contig Klebsiella pneumoniae pneumoniae CRK0291 Klebsiella pneumoniae 1 Klebsiella pneumoniae 1 Klebsiella pneumoniae 1 NA

GCF_002984395 Complete Klebsiella oxytoca FDAARGOS335 Klebsiella oxytoca 1 Klebsiella oxytoca 1 Klebsiella oxytoca 1 NA

GCF_002991725 Contig Klebsiella pneumoniae SC401 Klebsiella pneumoniae 1 Klebsiella pneumoniae 1 Klebsiella pneumoniae 1 NA

GCF_002991945 Contig Klebsiella pneumoniae SC137 Klebsiella pneumoniae 1 Klebsiella pneumoniae 1 Klebsiella pneumoniae 1 NA

GCF_002992025 Contig Klebsiella pneumoniae SC417 Klebsiella pneumoniae 1 Klebsiella pneumoniae 1 Klebsiella pneumoniae 1 NA

GCF_003006175 Complete Klebsiella pneumoniae DA48896 Klebsiella pneumoniae 1 Klebsiella pneumoniae 1 Klebsiella pneumoniae 1 NA

GCF_003006555 Contig Klebsiella pneumoniae K36 Klebsiella pneumoniae 1 Klebsiella pneumoniae 1 Klebsiella pneumoniae 1 NA

GCF_003010735 Complete Klebsiella pneumoniae ozaenae AR0096 Klebsiella pneumoniae 1 Klebsiella pneumoniae 1 Klebsiella pneumoniae 1 NA

GCF_003011775 Scaffold Klebsiella michiganensis 2654 Klebsiella michiganensis 1 Klebsiella michiganensis 1 Klebsiella michiganensis 1 NA

GCF_003012855 Contig Klebsiella pneumoniae B13 Klebsiella pneumoniae 1 Klebsiella pneumoniae 1 Klebsiella pneumoniae 1 NA

GCF_003020825 Complete Klebsiella quasipneumoniae KPC142 Klebsiella quasipneumoniae 1 Klebsiella quasipneumoniae 1 Klebsiella quasipneumoniae NA

GCF_003030145 Complete Klebsiella pneumoniae CFSAN054111 Klebsiella pneumoniae 1 Klebsiella pneumoniae 1 Klebsiella pneumoniae 1 NA

GCF_003031325 Complete Klebsiella pneumoniae NH25 Klebsiella pneumoniae 1 Klebsiella pneumoniae 1 Klebsiella pneumoniae 1 NA

GCF_003031345 Complete Klebsiella pneumoniae NH54 Klebsiella pneumoniae 1 Klebsiella pneumoniae 1 Klebsiella pneumoniae 1 NA

GCF_003031505 Complete Klebsiella pneumoniae BWHC1 Klebsiella pneumoniae 1 Klebsiella pneumoniae 1 Klebsiella pneumoniae 1 NA

GCF_003034385 Contig Klebsiella pneumoniae K4 Klebsiella pneumoniae 1 Klebsiella pneumoniae 1 Klebsiella pneumoniae 1 NA

GCF_003034395 Scaffold Klebsiella pneumoniae K9 Klebsiella pneumoniae 1 Klebsiella pneumoniae 1 Klebsiella pneumoniae 1 NA

GCF_003034425 Scaffold Klebsiella pneumoniae K19 Klebsiella pneumoniae 1 Klebsiella pneumoniae 1 Klebsiella pneumoniae 1 NA

GCF_003034435 Contig Klebsiella pneumoniae K3 Klebsiella pneumoniae 1 Klebsiella pneumoniae 1 Klebsiella pneumoniae 1 NA

GCF_003034445 Scaffold Klebsiella pneumoniae K5 Klebsiella pneumoniae 1 Klebsiella pneumoniae 1 Klebsiella pneumoniae 1 NA

GCF_003034485 Contig Klebsiella pneumoniae K2 Klebsiella pneumoniae 1 Klebsiella pneumoniae 1 Klebsiella pneumoniae 1 NA

GCF_003034505 Scaffold Klebsiella pneumoniae K18 Klebsiella pneumoniae 1 Klebsiella pneumoniae 1 Klebsiella pneumoniae 1 NA

GCF_003034525 Scaffold Klebsiella pneumoniae K13 Klebsiella pneumoniae 1 Klebsiella pneumoniae 1 Klebsiella pneumoniae 1 NA

GCF_003034545 Scaffold Klebsiella pneumoniae K10 Klebsiella pneumoniae 1 Klebsiella pneumoniae 1 Klebsiella pneumoniae 1 NA

GCF_003036445 Contig Klebsiella pneumoniae SCKP040067 Klebsiella pneumoniae 1 Klebsiella pneumoniae 1 Klebsiella pneumoniae 1 NA

GCF_003036465 Scaffold Klebsiella pneumoniae SCKP040072 Klebsiella pneumoniae 1 Klebsiella pneumoniae 1 Klebsiella pneumoniae 1 NA

GCF_003036485 Contig Klebsiella pneumoniae SCKP020055 Klebsiella pneumoniae 1 Klebsiella pneumoniae 1 Klebsiella pneumoniae 1 NA

GCF_003036505 Contig Klebsiella pneumoniae SCKP020052 Klebsiella pneumoniae 1 Klebsiella pneumoniae 1 Klebsiella pneumoniae 1 NA

GCF_003036515 Contig Klebsiella variicola SCKV020069 Klebsiella variicola Klebsiella variicola Klebsiella variicola NA

GCF_003036545 Scaffold Klebsiella pneumoniae SCKP020082 Klebsiella pneumoniae 1 Klebsiella pneumoniae 1 Klebsiella pneumoniae 1 NA

GCF_003036565 Contig Klebsiella pneumoniae WCHKP040051 Klebsiella pneumoniae 1 Klebsiella pneumoniae 1 Klebsiella pneumoniae 1 NA

GCF_003036575 Contig Klebsiella pneumoniae WCHKP085009 Klebsiella pneumoniae 1 Klebsiella pneumoniae 1 Klebsiella pneumoniae 1 NA

GCF_003036605 Scaffold Klebsiella pneumoniae WCHKP040052 Klebsiella pneumoniae 1 Klebsiella pneumoniae 1 Klebsiella pneumoniae 1 NA

GCF_003036615 Contig Klebsiella pneumoniae WCHKP040036 Klebsiella pneumoniae 1 Klebsiella pneumoniae 1 Klebsiella pneumoniae 1 NA

GCF_003036645 Contig Klebsiella pneumoniae WCHKP040009 Klebsiella pneumoniae 1 Klebsiella pneumoniae 1 Klebsiella pneumoniae 1 NA

GCF_003036665 Contig Klebsiella pneumoniae WCHKP040011 Klebsiella pneumoniae 1 Klebsiella pneumoniae 1 Klebsiella pneumoniae 1 NA

GCF_003036685 Contig Klebsiella pneumoniae WCHKP040023 Klebsiella pneumoniae 1 Klebsiella pneumoniae 1 Klebsiella pneumoniae 1 NA

GCF_003036705 Contig Klebsiella pneumoniae WCHKP040016 Klebsiella pneumoniae 1 Klebsiella pneumoniae 1 Klebsiella pneumoniae 1 NA

GCF_003036725 Scaffold Klebsiella pneumoniae WCHKP040024 Klebsiella pneumoniae 1 Klebsiella pneumoniae 1 Klebsiella pneumoniae 1 NA

GCF_003036735 Contig Klebsiella pneumoniae WCHKP040054 Klebsiella pneumoniae 1 Klebsiella pneumoniae 1 Klebsiella pneumoniae 1 NA

GCF_003036765 Scaffold Klebsiella pneumoniae WCHKP015348 Klebsiella pneumoniae 1 Klebsiella pneumoniae 1 Klebsiella pneumoniae 1 NA

GCF_003036785 Contig Klebsiella pneumoniae WCHKP040015 Klebsiella pneumoniae 1 Klebsiella pneumoniae 1 Klebsiella pneumoniae 1 NA

GCF_003036805 Scaffold Klebsiella pneumoniae WCHKP015322 Klebsiella pneumoniae 1 Klebsiella pneumoniae 1 Klebsiella pneumoniae 1 NA

GCF_003036815 Scaffold Klebsiella pneumoniae WCHKP015288 Klebsiella pneumoniae 1 Klebsiella pneumoniae 1 Klebsiella pneumoniae 1 NA

GCF_003036845 Scaffold Klebsiella pneumoniae WCHKP015271 Klebsiella pneumoniae 1 Klebsiella pneumoniae 1 Klebsiella pneumoniae 1 NA

GCF_003036865 Scaffold Klebsiella pneumoniae WCHKP015305 Klebsiella pneumoniae 1 Klebsiella pneumoniae 1 Klebsiella pneumoniae 1 NA

GCF_003036885 Scaffold Klebsiella pneumoniae WCHKP015255 Klebsiella pneumoniae 1 Klebsiella pneumoniae 1 Klebsiella pneumoniae 1 NA

GCF_003036895 Scaffold Klebsiella pneumoniae WCHKP015240 Klebsiella pneumoniae 1 Klebsiella pneumoniae 1 Klebsiella pneumoniae 1 NA

GCF_003036925 Scaffold Klebsiella pneumoniae WCHKP015231 Klebsiella pneumoniae 1 Klebsiella pneumoniae 1 Klebsiella pneumoniae 1 NA

GCF_003036945 Scaffold Klebsiella pneumoniae WCHKP015230 Klebsiella pneumoniae 1 Klebsiella pneumoniae 1 Klebsiella pneumoniae 1 NA

GCF_003036965 Scaffold Klebsiella pneumoniae WCHKP015155 Klebsiella pneumoniae 1 Klebsiella pneumoniae 1 Klebsiella pneumoniae 1 NA

GCF_003036985 Contig Klebsiella pneumoniae WCHKP020107 Klebsiella pneumoniae 1 Klebsiella pneumoniae 1 Klebsiella pneumoniae 1 NA

GCF_003037005 Complete Klebsiella pneumoniae WCHKP020098 Klebsiella pneumoniae 1 Klebsiella pneumoniae 1 Klebsiella pneumoniae 1 NA

GCF_003037025 Contig Klebsiella pneumoniae WCHKP020093 Klebsiella pneumoniae 1 Klebsiella pneumoniae 1 Klebsiella pneumoniae 1 NA

GCF_003037045 Scaffold Klebsiella pneumoniae WCHKP040041 Klebsiella pneumoniae 1 Klebsiella pneumoniae 1 Klebsiella pneumoniae 1 NA

GCF_003037055 Scaffold Klebsiella pneumoniae WCHKP040057 Klebsiella pneumoniae 1 Klebsiella pneumoniae 1 Klebsiella pneumoniae 1 NA

GCF_003037065 Scaffold Klebsiella pneumoniae WCHKP040059 Klebsiella pneumoniae 1 Klebsiella pneumoniae 1 Klebsiella pneumoniae 1 NA

GCF_003037105 Scaffold Klebsiella pneumoniae WCHKP040050 Klebsiella pneumoniae 1 Klebsiella pneumoniae 1 Klebsiella pneumoniae 1 NA

GCF_003037125 Contig Klebsiella pneumoniae WCHKP040020 Klebsiella pneumoniae 1 Klebsiella pneumoniae 1 Klebsiella pneumoniae 1 NA

GCF_003037145 Scaffold Klebsiella pneumoniae WCHKP040021 Klebsiella pneumoniae 1 Klebsiella pneumoniae 1 Klebsiella pneumoniae 1 NA

GCF_003037165 Contig Klebsiella pneumoniae pneumoniae WCHKP020117 Klebsiella pneumoniae 1 Klebsiella pneumoniae 1 Klebsiella pneumoniae 1 NA

GCF_003037185 Contig Klebsiella pneumoniae SCKP020018 Klebsiella pneumoniae 1 Klebsiella pneumoniae 1 Klebsiella pneumoniae 1 NA

GCF_003037205 Scaffold Klebsiella pneumoniae WCHKP040025 Klebsiella pneumoniae 1 Klebsiella pneumoniae 1 Klebsiella pneumoniae 1 NA

GCF_003037225 Contig Klebsiella pneumoniae WCHKP040004 Klebsiella pneumoniae 1 Klebsiella pneumoniae 1 Klebsiella pneumoniae 1 NA

GCF_003037245 Scaffold Klebsiella pneumoniae WCHKP040028 Klebsiella pneumoniae 1 Klebsiella pneumoniae 1 Klebsiella pneumoniae 1 NA

GCF_003037265 Contig Klebsiella pneumoniae WCHKP040002 Klebsiella pneumoniae 1 Klebsiella pneumoniae 1 Klebsiella pneumoniae 1 NA

GCF_003037275 Contig Klebsiella pneumoniae WCHKP040010 Klebsiella pneumoniae 1 Klebsiella pneumoniae 1 Klebsiella pneumoniae 1 NA

GCF_003037305 Contig Klebsiella pneumoniae WCHKP040055 Klebsiella pneumoniae 1 Klebsiella pneumoniae 1 Klebsiella pneumoniae 1 NA

GCF_003037325 Complete Klebsiella pneumoniae WCHKP040035 Klebsiella pneumoniae 1 Klebsiella pneumoniae 1 Klebsiella pneumoniae 1 NA

GCF_003037335 Contig Klebsiella pneumoniae WCHKP040049 Klebsiella pneumoniae 1 Klebsiella pneumoniae 1 Klebsiella pneumoniae 1 NA

GCF_003037365 Scaffold Klebsiella pneumoniae WCHKP015336 Klebsiella pneumoniae 1 Klebsiella pneumoniae 1 Klebsiella pneumoniae 1 NA

GCF_003037385 Scaffold Klebsiella pneumoniae WCHKP015314 Klebsiella pneumoniae 1 Klebsiella pneumoniae 1 Klebsiella pneumoniae 1 NA

GCF_003037395 Scaffold Klebsiella pneumoniae WCHKP015256 Klebsiella pneumoniae 1 Klebsiella pneumoniae 1 Klebsiella pneumoniae 1 NA

GCF_003037435 Scaffold Klebsiella pneumoniae WCHKP015235 Klebsiella pneumoniae 1 Klebsiella pneumoniae 1 Klebsiella pneumoniae 1 NA

GCF_003037455 Contig Klebsiella pneumoniae WCHKP020094 Klebsiella pneumoniae 1 Klebsiella pneumoniae 1 Klebsiella pneumoniae 1 NA

GCF_003037475 Scaffold Klebsiella pneumoniae WCHKP015229 Klebsiella pneumoniae 1 Klebsiella pneumoniae 1 Klebsiella pneumoniae 1 NA

GCF_003037485 Contig Klebsiella pneumoniae WCHKP020027 Klebsiella pneumoniae 1 Klebsiella pneumoniae 1 Klebsiella pneumoniae 1 NA

GCF_003037515 Scaffold Klebsiella pneumoniae WCHKP020112 Klebsiella pneumoniae 1 Klebsiella pneumoniae 1 Klebsiella pneumoniae 1 NA

GCF_003037535 Scaffold Klebsiella pneumoniae WCHKP040058 Klebsiella pneumoniae 1 Klebsiella pneumoniae 1 Klebsiella pneumoniae 1 NA

GCF_003037555 Contig Klebsiella pneumoniae WCHKP040017 Klebsiella pneumoniae 1 Klebsiella pneumoniae 1 Klebsiella pneumoniae 1 NA

GCF_003037565 Contig Klebsiella pneumoniae WCHKP040013 Klebsiella pneumoniae 1 Klebsiella pneumoniae 1 Klebsiella pneumoniae 1 NA

GCF_003037585 Scaffold Klebsiella pneumoniae WCHKP040046 Klebsiella pneumoniae 1 Klebsiella pneumoniae 1 Klebsiella pneumoniae 1 NA

GCF_003037615 Complete Klebsiella pneumoniae pneumoniae SCKP040074 Klebsiella pneumoniae 1 Klebsiella pneumoniae 1 Klebsiella pneumoniae 1 NA

GCF_003037655 Contig Klebsiella pneumoniae SCKP040075 Klebsiella pneumoniae 1 Klebsiella pneumoniae 1 Klebsiella pneumoniae 1 NA

GCF_003037665 Contig Klebsiella pneumoniae SCKP040073 Klebsiella pneumoniae 1 Klebsiella pneumoniae 1 Klebsiella pneumoniae 1 NA

GCF_003037695 Contig Klebsiella pneumoniae SCKP020053 Klebsiella pneumoniae 1 Klebsiella pneumoniae 1 Klebsiella pneumoniae 1 NA
[truncated: 1,371,728 more chars]
